# Supplementary material for: Arylcarboxylation of unactivated alkenes with CO2 via visible-light photoredox catalysis
Source: Nat Commun. 2023 Jun 14;14:3529. doi: 10.1038/s41467-023-39240-8 (PMC10267151; doi:10.1038/s41467-023-39240-8)
Supplement: Supplementary file 1 — Supplementary Information [file 41467_2023_39240_MOESM1_ESM.pdf]

Supplementary Information

for

Arylcarboxylation of unactivated alkenes with CO_2 via visible-light photoredox catalysis

Wei Zhang,[1,2] Zhen Chen,[1] Yuan-Xu Jiang,[1] Li-Li Liao,[3] Wei Wang,[1] Jian-Heng Ye,[1]* and Da-Gang Yu[1,4]*

[1]Key Laboratory of Green Chemistry & Technology of Ministry of Education, College of Chemistry, Sichuan University, Chengdu 610064

[2]West China School of Public Health and West China Fourth Hospital, Sichuan University, Chengdu 610064

[3]School of Chemistry and Chemical Engineering, Chongqing University, Chongqing 400030, P. R. China

[4]State Key Laboratory of Elemento-Organic Chemistry, Nankai University Tianjin 300071, P. R. China

*Correspondence and requests for materials should be addressed to J.-H. Ye (email: jhye@scu.edu.cn) or D.-G. Yu (email: dgyu@scu.edu.cn)

*Correspondence: jhye@scu.edu.cn; dgyu@scu.edu.cn

Table of contents

1 Supplementary Methods 3

1.1 General considerations 3

2 Supplementary Discussion 4

2.1 Preparation of substrates 4

2.2 Additional reaction optimization 8

Screening reaction parameters for arylcarboxylation 8

2.3 Experimental procedures 11

2.4 Gram scale of 1a 31

2.5 Product derivations 32

3 Supplementry Notes 40

3.1 Mechanistic investigations 40

3.1.1 Trapping experiments 40

3.1.2 Reduction of unactivated alkenes 1a 41

3.1.3 Detection of the formate and oxalate 42

3.1.4 Luminescence quenching experiments 52

3.1.5 Evidence of possible HAT process 54

3.1.6 NMR spectroscopic evidence of possible ConPET process 55

3.1.7 Density Functional Theory (DFT) calculations 56

3.1.8 Proposed mechanism in the absence of silane 59

3.1.9 Proposed mechanism in the presence of silane 62

3.2 NMR spectra 64

4 Supplementary References 121

1 Supplementary Methods

1.1 General considerations

All reactions were set up using standard Schlenk techniques and carried out under a CO_2 atmosphere with dry solvents. Commercially available chemicals were obtained from Adamas-beta, Energy Chemical, Bidepharm, TCI or J&K Scientific and used as received unless otherwise stated. *fac*-Ir(ppy)$_3$ was prepared according to the literature procedure[1] or purchased from Bidepharm. Anhydrous dimethyl sulfoxide (DMSO) was purchased from J&K Scientific. Cesium carbonate (Cs_2CO_3) was purchased from Accela ChemBio. Dimethylphenylsilane ($PhMe_2SiH$) was purchased from J&K Scientific. 4-*tert*-butylthiophenol (HAT catalyst) was purchased from TCI. Reactions were monitored by thin-layer chromatography (TLC) carried out on 0.2 ±0.03 mm using UV light as a visualizing agent and bromocresol green in EtOH or phosphomolybdic acid in ethanol as developing agents.

^{1}H, ^{19}F, and ^{13}C NMR spectra were recorded on a Brüker Advance 400 spectrometer (^{1}H: 400 MHz, ^{19}F: 376 MHz, ^{13}C: 101 MHz). Chemical shifts (δ) for ^{1}H and ^{13}C NMR spectra are given in ppm relative to TMS. The residual solvent signals were used as references for ^{1}H and ^{13}C NMR spectra and the chemical shifts converted to the TMS scale ($CDCl_3$: δH = 7.26 ppm, δC = 77.16 ppm, CD_3CN-*d*$_3$: δH = 1.32 ppm, δC = 1.32, 118.26 ppm, CD_3OD: δH = 3.31 ppm, δC = 49.00 ppm). The following abbreviations were used to explain the multiplicities: s = singlet, d = doublet, t = triplet, q = quartet, m = multiplet, br = broad.

LRMS was obtained using Thermo-Fisher LTQ-ESI-MS. Exact ESI mass spectra were recorded on a SHIMADZU LCMS-IT-TOF. ESI-MS were obtained on a Thermo-LTQ. TLC was performed using commercially prepared 100-400 mesh silica gel plates (GF254), and visualization was effected at 254 nm. Visible light irradiation was performed with a 30 W LED lamp at λ_{ir} = 450 ± 10 nm) for photocatalytic reactions.

2 Supplementary Discussion

2.1 Preparation of substrates

Substrates **3a-3i and 5a-5d** were prepared according to the previously reported literature procedures.[1-3]

General synthetic methods for substrate 1a-1h, 1l-1t, 1v-1ad

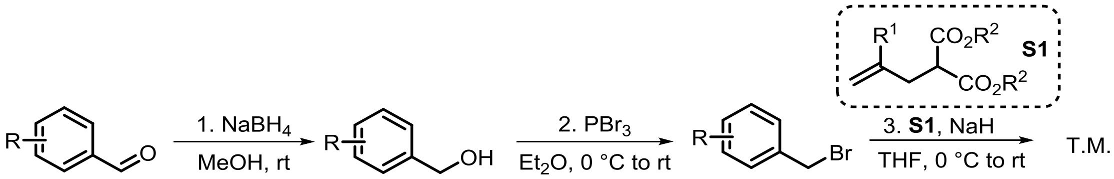

Step 1. To a solution of aromatic aldehyde (10 mmol, 1.0 equiv) in MeOH (50 mL) was added $NaBH_4$ (15 mmol, 1.5 equiv) slowly at 0 ℃. The resulting mixture was stirred at room temperature for 2 hours. After the reaction was completed, the reaction mixture was quenched with water (40 mL) and extracted with ethyl acetate three times, dried over Na_2SO_4. The combined filtrate was concentrated and the residue was purified by a silica gel column chromatography to give the pure benzyl alcohol.

Step 2. A dried Schlenk flask was charged with benzyl alcohol (10 mmol, 1.0 equiv). The flask was evacuated and backfilled with N_2 three times. After the addition of dry Et_2O (30 mL), PBr_3 (15 mmol, 1.5 equiv) was added dropwise at 0 ℃. The resulting mixture was stirred at 0 ℃ for 1 hour and stirred at room temperature for 5 hours. After the reaction was completed, the reaction mixture was then cooled to 0 ℃, quenched with saturated $NaHCO_3$ and extracted with ethyl acetate three times, washed with brine, and dried over Na_2SO_4. The combined organic phase was concentrated *in vacuo* and the resulting crude product was used for the next step without further purification.

Step 3. To a solution of NaH (30 mmol, 1.5 equiv) in dry THF (50 mL) was added **S1** (20 mmol, 1.0 equiv) dropwise at 0 ℃. The resulting mixture was stirred for 30 min. Benzyl bromide (24 mmol, 1.2 equiv) was added slowly at 0 ℃, the reaction was warmed up to room temperature and TLC monitored the complete conversion of di-

tert-butyl 2-allylmalonate, the reaction mixture was quenched with water and extracted with ethyl acetate three times, dried by Na_2SO_4 and concentrated. The crude product was purified by flash chromatography on silica gel to give the target product.

General synthetic methods for substrate 1i-1k

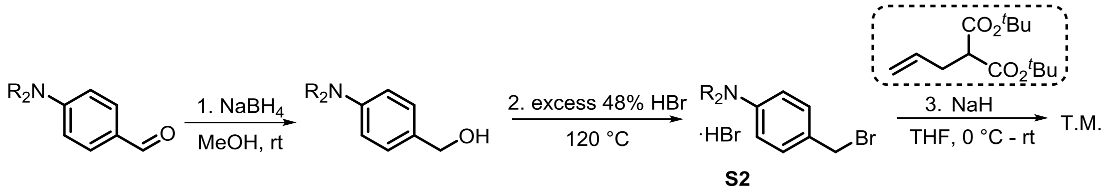

Step 1. To a solution of aromatic aldehyde (10 mmol, 1.0 equiv) in MeOH (50 mL) was added $NaBH_4$ (15 mmol, 1.5 equiv) slowly at 0 ℃. The resulting mixture was stirred at room temperature for 2 hours. After the reaction was completed, the reaction mixture was quenched with water (40 mL) and extracted with ethyl acetate three times, dried over Na_2SO_4. The combined filtrate was concentrated and the residue was purified by a silica gel column chromatography to give the pure benzyl alcohol.

Step 2. A dried Schlenk flask was charged with benzyl alcohol (10 mmol, 1.0 equiv). The flask was evacuated and backfilled with N_2 three times and 48% HBr was added dropwise at room temperature. The reaction was heated to 120 ℃ and stirred for 2 hours. After the reaction was completed, it was allowed to cool to room temperature, and water was then removed *in vacuo*. The resulting crude product was used for the next step without further purification.

Step 3. To a solution of NaH (30 mmol, 1.5 equiv) in dry THF (50 mL) was added di-*tert*-butyl 2-allylmalonate (20 mmol, 1.0 equiv) slowly at 0 ℃. The resulting mixture was stirred for 30 min. **S2** (24 mmol, 1.2 equiv) was added dropwise at 0 ℃, the reaction was warmed up to room temperature and TLC monitored the complete conversion of di-*tert*-butyl 2-allylmalonate. The reaction mixture was quenched with water and extracted with ethyl acetate three times, dried by Na_2SO_4 and concentrated. The crude product was purified by flash chromatography on silica gel to give the target product.

General synthetic methods for substrates 1x-1z

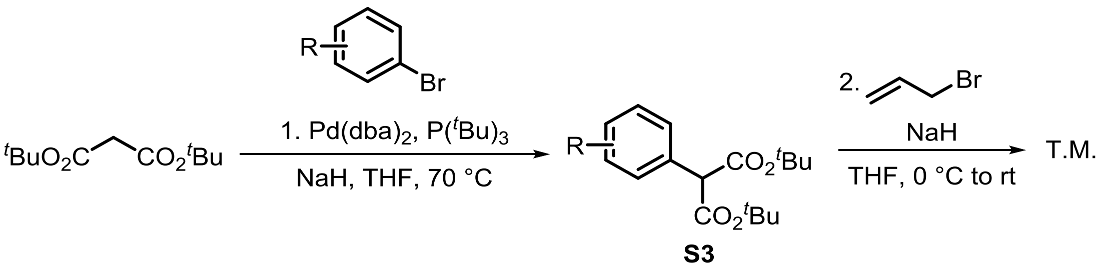

Step 1. An oven-dried 25 mL Schlenk flask equipped with a magnetic stir bar was evacuated and backfilled with N_2 three times. Di-*tert*-butyl malonate (2.2 mmol, 1.1 equiv) and dry THF (2 mL) were added, followed by NaH (2.4 mmol, 1.2 equiv). After the evolution of hydrogen was complete (ca. 2 min), aryl bromide (1.0 mmol, 1.0 equiv), phosphine (0.040 mmol, 0.04 equiv), Pd(dba)$_2$ (0.020 mmol, 0.02 equiv), and additional THF (4.0 mL) were added. The vial was sealed with a cap containing a PTFE septum and removed from the drybox. The homogeneous reaction mixture was stirred at 70 ℃ and monitored by GC-MS. After the complete conversion of the aryl bromide, the crude reaction was filtered through a plug of celite and concentrated in vacuo. The residue was purified by flash chromatography on silica gel to give the **S3**.

Step 2. To a solution of NaH (2.8 mmol, 1.4 equiv) in dry THF (5 mL) was added **S3** (2 mmol, 1.0 equiv) dropwise at 0 ℃. The resulting mixture was stirred for 30 min. Allyl bromide (2.8 mmol, 1.4 equiv) was added slowly at 0 ℃, the reaction was warmed up to room temperature and TLC monitored the complete conversion of **S3**, the reaction mixture was quenched with water and extracted with ethyl acetate three times, dried by Na_2SO_4 and concentrated. The crude product was purified by flash chromatography on silica gel to give the target product.

Synthesis of substrate 1u

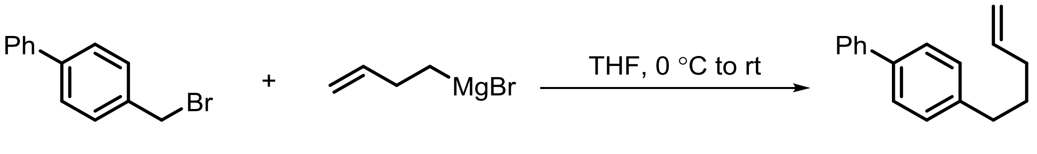

To a stirred solution of benzyl bromide (1.0 equiv, 20 mmol) in dry THF (20 mL) was added 3-en-1-ylmagnesium bromide (2 mmol, 1.0 equiv) dropwise at 0 ℃. The resulting mixture was stirred at room temperature for 10 hours. The reaction mixture was quenched with saturated NH_4Cl and extracted with diethyl ether three times, dried by Na_2SO_4. The combined organic phase was concentrated *in vacuo* and the residue was purified by flash chromatography on silica gel to give the **1u**.

2.2 Additional reaction optimization

Screening reaction parameters for arylcarboxylation

Supplementary Table 1: Screening of photocatalyst[a]

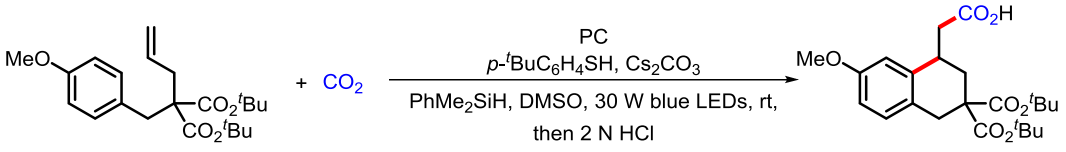

1a (1 atm) **2a**

Entry	PC	Yield[b]
1	$Ir[(ppy)_2(dtbbpy)]PF_6$	60%
2	*fac*-$Ir(ppy)_3$	86%
3	3DPA2FBN	N.D.
4	4-CzIPN	N.D.

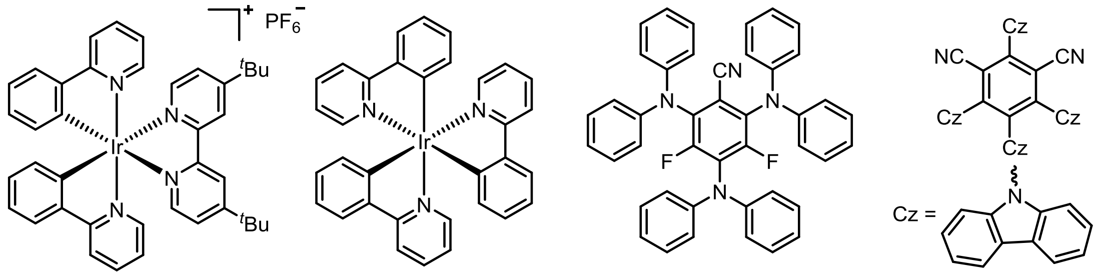

$Ir(ppy)_2(dtbbpy)PF_6$ *fac*-$Ir(ppy)_3$ 3DPA2FBN 4CzIPN

[a]Reaction conditions: **2a** (0.2 mmol, 1.0 equiv), PC (1 mol%), p-^{t}BuC$_6$H$_4$SH (20 mol%), Cs_2CO_3 (3.0 equiv), PhMe$_2$SiH (1.0 equiv), DMSO (2 mL), irradiation by blue LEDs at room temperature under CO_2 (1 atm) for 24 h, 25 mL Schlenk tube, acidification by HCl (2 N), [b]^{1}H NMR yields with 1,3,5-trimethoxybenzene as an internal standard.

Supplementary Table 2: Screening of solvent[a]

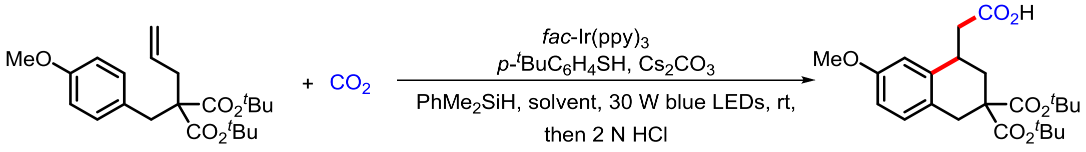

1a (1 atm) **2a**

Entry	Solvent	Yield[b]
1	DMSO	86%
2	DMF	55%
3	DMAc	58%
4	NMP	44%

[a]Reaction conditions: **2a** (0.2 mmol, 1.0 equiv), *fac*-Ir(ppy)$_3$ (1 mol%), *p*-^{t}BuC$_6$H$_4$SH (20 mol%), Cs_2CO_3 (3.0 equiv), $PhMe_2SiH$ (1.0 equiv), solvent (2 mL), irradiation by blue LEDs at room temperature under CO_2 (1 atm) for 24 h, 25 mL Schlenk tube, acidification by HCl (2 N), [b]^{1}H NMR yields with 1,3,5-trimethoxybenzene as an internal standard.

Supplementary Table 3: Screening of silane[a]

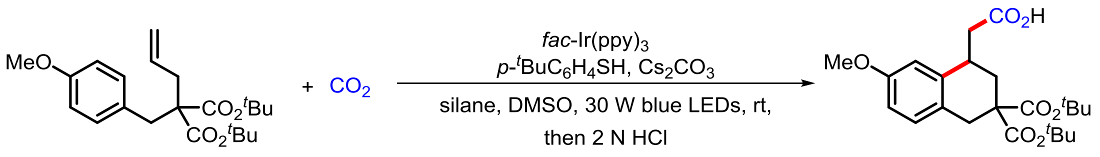

1a (1 atm) **2a**

Entry	Silane	Yield[b]
1	$PhMe_2SiH$	86%
2	Ph_3SiH	82%
3	Et_3SiH	85%
4	PMHS	82%

[a]Reaction conditions: **2a** (0.2 mmol, 1.0 equiv), *fac*-Ir(ppy)$_3$ (1 mol%), *p*-^{t}BuC$_6$H$_4$SH (20 mol%), Cs_2CO_3 (3.0 equiv), silane (1.0 equiv), DMSO (2 mL), irradiation by blue LEDs at room temperature under CO_2 (1 atm) for 24 h, 25 mL Schlenk tube, acidification by HCl (2 N), [b]^{1}H NMR yields with 1,3,5-trimethoxybenzene as an internal standard.

Supplementary Table 4: Screening of HAT catalyst[a]

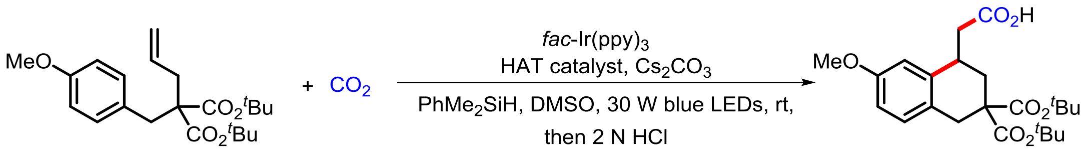

1a (1 atm) **2a**

Entry	HAT catalyst	Yield[b]
1	*p*-^{t}BuC$_6$H$_4$SH	86%
2	*p*-^{t}BuSH	82%
3	Ph_3SiOH	trace
4	2,4,6-^{t}Bu$_3$C$_6$H$_2$SH	76%
5[c]	*p*-^{t}BuC$_6$H$_4$SH	66%
6[c]	*p*-^{t}BuC$_6$H$_4$SK	62%

[a]Reaction conditions: **2a** (0.2 mmol, 1.0 equiv), *fac*-Ir(ppy)$_3$ (1 mol%), HAT catalyst (20 mol%), Cs_2CO_3 (3.0 equiv), $PhMe_2SiH$ (1.0 equiv), DMSO (2 mL), irradiation by blue LEDs at room temperature under CO_2 (1 atm) for 24 h, 25 mL Schlenk tube, acidification by HCl (2 N), [b]^{1}H NMR yields with 1,3,5-trimethoxybenzene as an internal standard. [c]w/o $PhMe_2SiH$.

Supplementary Table 5: Screening of base[a]

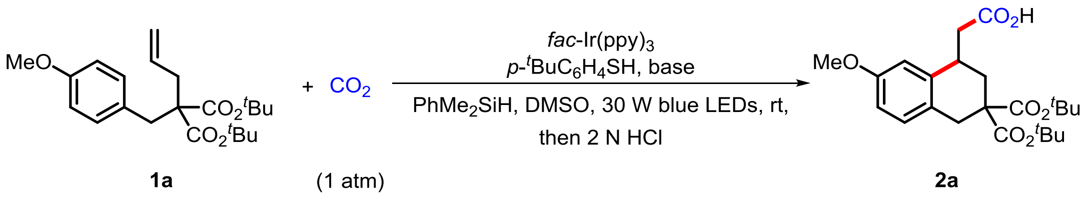

Entry	Base	Yield[b]
1	Cs_2CO_3	86%
2	K_2CO_3	68%
3	Na_2CO_3	6%
4	$CsHCO_3$	10%

[a]Reaction conditions: **2a** (0.2 mmol, 1.0 equiv), *fac*-Ir(ppy)$_3$ (1 mol%), *p*-^{t}BuC$_6$H$_4$SH (20 mol%), base (3.0 equiv), PhMe$_2$SiH (1.0 equiv), DMSO (2 mL), irradiation by blue LEDs at room temperature under CO_2 (1 atm) for 24 h, 25 mL Schlenk tube, acidification by HCl (2 N), [b]^{1}H NMR yields with 1,3,5-trimethoxybenzene as an internal standard.

2.3 Experimental procedures

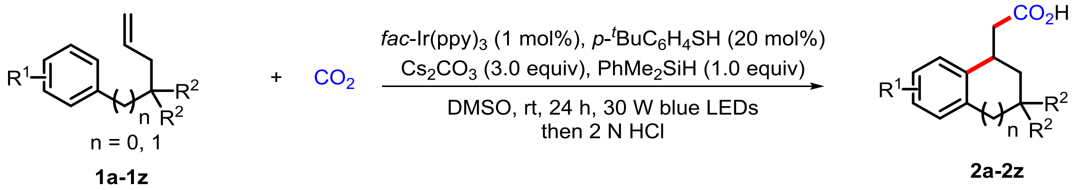

Procedure: To an oven-dried Schlenk tube (25 mL) equipped with a magnetic stir bar were added the unactivated alkenes (0.2 mmol, 1.0 equiv for solid substrates) and *fac*-Ir(ppy)$_3$ (1 mol%). The tube was moved into the glovebox where was added the Cs_2CO_3 (0.6 mmol, 195.5 mg, 3.0 equiv). The tube was sealed and removed from the glovebox, then evacuated and back-filled with CO_2 atmosphere three times. liquid alkenes were added under CO_2 atmosphere followed by anhydrous DMSO (2 mL), $PhMe_2SiH$ (0.2 mmol, 27.3 mg, 31 μL, 1.0 equiv), 4-*tert*-butylthiophenol (0.04 mol, 6.7 mg, 7.0 μL, 20 mol%), and the tube was sealed at atmospheric pressure of CO_2 (1 atm). The reaction was stirred and irradiated with a 30 W blue LED lamp (1 cm away, with a cooling fan to keep the reaction temperature at 25-30 ℃ and keeping the reaction region located in the center of LEDs lamp) for 24 hours. Upon completion of the reaction, the reaction mixture was diluted with 3 mL EA and quenched by 3 mL 2 N HCl. After adding 10 mL of H_2O, the mixture was extracted with EA five times and the combined organic phases were concentrated *in vacuo*. The residue was purified by a silica gel flash column chromatography (PE/EA/AcOH 10/1/ ~ 5/1~/5/10.2%) to give the pure desired product.

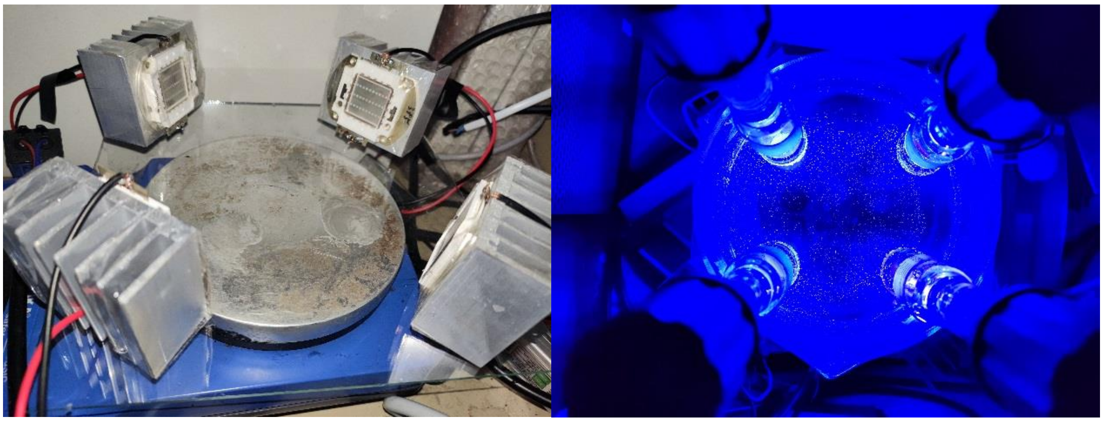

Supplementary Figure 1. Blue LEDs photoreactor for arylcarboxylation.

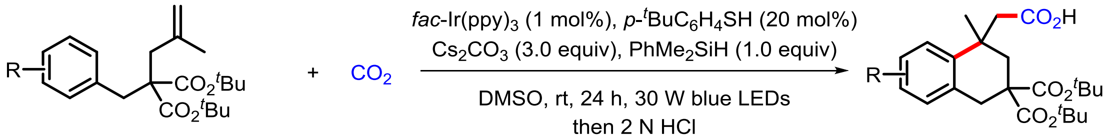

1aa-1ad **2aa-2ad**

Procedure: To an oven-dried Schlenk tube (25 mL) equipped with a magnetic stir bar were added the unactivated alkenes (0.2 mmol, 1.0 equiv for solid substrates) and *fac*-Ir(ppy)$_3$ (1 mol%). The tube was moved into the glovebox where was added the Cs_2CO_3 (0.6 mmol, 195.5 mg, 3.0 equiv). The tube was sealed and removed from the glovebox, then evacuated and back-filled with CO_2 atmosphere three times. liquid alkenes were added under CO_2 atmosphere followed by anhydrous DMSO (2 mL), $PhMe_2SiH$ (0.2 mmol, 27.3 mg, 31 μL, 1.0 equiv), 4-*tert*-butylthiophenol (0.04 mol, 6.7 mg, 7.0 μL, 20 mol%), and the tube was sealed at atmospheric pressure of CO_2 (1 atm). The reaction was stirred and irradiated with a 30 W blue LED lamp (1 cm away, with a cooling fan to keep the reaction temperature at 25-30 ℃ and keeping the reaction region located in the center of LEDs lamp) for 24 hours. Upon completion of the reaction, the reaction mixture was diluted with 3 mL EA and quenched by 3 mL 2 N HCl. After adding 10 mL of H_2O, the mixture was extracted with EA five times and the combined organic phases were concentrated *in vacuo*. The residue was purified by a silica gel flash column chromatography (PE/EA/AcOH 10/1/ ~ 5/1~/5/10.2%) to give the pure desired product.

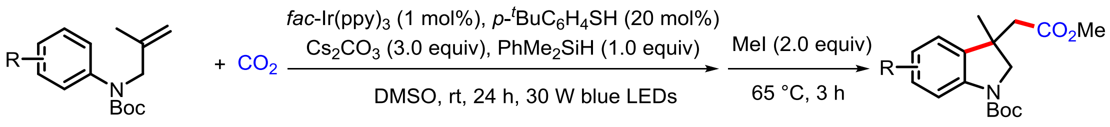

3 **4**

Procedure: To an oven-dried Schlenk tube (25 mL) equipped with a magnetic stir bar were added the unactivated alkenes (0.2 mmol, 1.0 equiv for solid substrates) and *fac*-Ir(ppy)$_3$ (1 mol%). The tube was moved into the glovebox where was added the Cs_2CO_3 (0.6 mmol, 195.5 mg, 3.0 equiv). The tube was sealed and removed from the glovebox, then evacuated and back-filled with CO_2 atmosphere three times. liquid alkenes were added under CO_2 atmosphere followed by anhydrous DMSO (2 mL), $PhMe_2SiH$ (0.2 mmol, 27.3 mg, 31 μL, 1.0 equiv), 4-*tert*-butylthiophenol (0.04 mol, 6.7 mg, 7.0 μL, 20 mol%), and the tube was sealed at atmospheric pressure of CO_2 (1 atm). The reaction was stirred and irradiated with a 30 W blue LED lamp (1 cm away, with a cooling fan to keep the reaction temperature at 25-30 ℃ and keeping the reaction region located in the center of LEDs lamp) for 24 hours. Upon completion of the reaction, MeI (0.4 mmol, 25 μL, 2.0 equiv) was added, the mixture was stirred at 65 ^{o}C for 3 h and then cooled to room temperature. The crude reaction mixture was diluted with 3 mL EA. After adding 10 mL of H_2O, the mixture was extracted with EA five times and the combined organic phases were concentrated *in vacuo*. The residue was purified by a silica gel flash column chromatography (PE/EA 60/1/ ~ 20/1) to give the pure desired product.

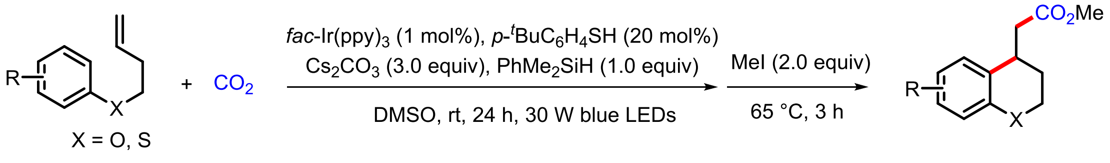

5

6

Procedure: To an oven-dried Schlenk tube (25 mL) equipped with a magnetic stir bar were added the unactivated alkenes (0.2 mmol, 1.0 equiv for solid substrates) and *fac*-Ir(ppy)$_3$ (1 mol%). The tube was moved into the glovebox where was added the Cs_2CO_3 (0.6 mmol, 195.5 mg, 3.0 equiv). The tube was sealed and removed from the glovebox, then evacuated and back-filled with CO_2 atmosphere three times. liquid alkenes were added under CO_2 atmosphere followed by anhydrous DMSO (2 mL), $PhMe_2SiH$ (0.2 mmol, 27.3 mg, 31 μL, 1.0 equiv), 4-*tert*-butylthiophenol (0.04 mol, 6.7 mg, 7.0 μL, 20 mol%), and the tube was sealed at atmospheric pressure of CO_2 (1 atm). The reaction was stirred and irradiated with a 30 W blue LED lamp (1 cm away, with a cooling fan to keep the reaction temperature at 25-30 ℃ and keeping the reaction region located in the center of LEDs lamp) for 24 hours. Upon completion of the reaction, MeI (0.4 mmol, 25 μL, 2.0 equiv) was added, and the mixture was stirred at 65 °C for 3 h and then cooled to room temperature. The crude reaction mixture was diluted with 3 mL EA. After adding 10 mL of H_2O, the mixture was extracted with EA five times and the combined organic phases were concentrated *in vacuo*. The residue was first purified by a silica gel flash column chromatography (PE/EA 150/1/ ~ 60/1) to give the mixture and the yields were determined with CH_2Br_2 as an internal standard. The desired arylcarboxylation products were further purified by preparative HPLC.

2-(3,3-bis(tert-butoxycarbonyl)-7-methoxy-1,2,3,4-tetrahydronaphthalen-1-yl) acetic acid (2a)

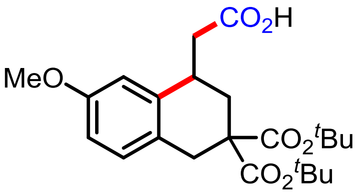

70.0 mg, 83% yield, yellow oil;

R_f (PE/EA = 1:1) = 0.6 - 0.7;

^{1}H NMR (400 MHz, $CDCl_3$) δ 7.06 – 7.01 (m, 1H), 6.73 – 6.68 (m, 2H), 3.75 (s, 3H), 3.41 – 3.29 (m, 1H), 3.20 (dd, J = 15.8, 1.8 Hz, 1H), 3.04 – 2.90 (m, 2H), 2.68 – 2.49 (m, 2H), 1.85 (dd, J = 13.4, 10.4 Hz, 1H), 1.46 (s, 9H), 1.35 (s, 9H); **^{13}C NMR** (101 MHz, $CDCl_3$) δ 178.4, 171.1, 169.9, 158.2, 138.3, 129.9, 126.7, 112.3, 111.6, 81.7, 81.2, 55.2, 54.9, 40.7, 34.6, 34.5, 32.6, 27.9, 27.7; **HRMS (ESI-)**: calculated for $C_{23}H_{31}O_7^-$ $[M-H]^-$ 419.2075, found 419.2077.

2-(7-(benzyloxy)-3,3-bis(tert-butoxycarbonyl)-1,2,3,4-tetrahydronaphthalen-1-yl) acetic acid (2b)

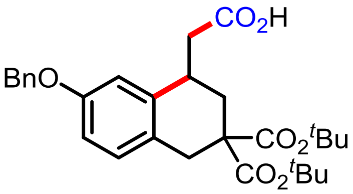

67.3 mg, 68% yield, pale yellow solid;

R_f (PE/EA = 1:1) = 0.4 - 0.5;

Mp 108-111 ℃

^{1}H NMR (400 MHz, $CDCl_3$) δ 7.45 – 7.35 (m, 4H), 7.35 – 7.28 (m, 1H), 7.08 – 7.02 (m, 1H), 6.82 – 6.76 (m, 2H), 5.02 (s, 2H), 3.36 (m, 1H), 3.29 – 3.16 (m, 1H), 3.07 – 2.89 (m, 2H), 2.53 (dd, J = 16.2, 9.2 Hz, 2H), 1.87 (dd, J = 13.5, 10.5 Hz, 1H), 1.47 (s, 9H), 1.37 (s, 9H); **^{13}C NMR** (101 MHz, $CDCl_3$) δ 178.4, 171.1, 169.9, 157.4, 138.3, 137.1, 129. 9, 128.6, 127.9, 127.5, 127.1, 113.2, 112.7, 81.7, 81.3, 70.1, 54.9, 40.7, 34.6, 34.5, 32.5, 27.9, 27.7; **HRMS (ESI-):** calculated for $C_{29}H_{35}O_7^-$ $[M-H]^-$ 495.2388, found 495.2391.

2-(3,3-bis(tert-butoxycarbonyl)-7-phenoxy-1,2,3,4-tetrahydronaphthalen-1-yl) acetic acid (2c)

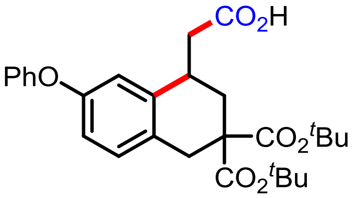

66.9 mg, 69% yield, white solid;

R_f (PE/EA = 2:1) = 0.5 - 0.6;

Mp 139-142 ℃

^{1}H NMR (400 MHz, $CDCl_3$) δ 7.34 – 7.28 (m, 2H), 7.11 – 7.05 (m, 2H), 6.97-6.95 (m, 2H), 6.86 – 6.77 (m, 2H), 3.36-3.32 (m, 1H), 3.30 – 3.20 (d, J = 15.7 Hz, 1H), 3.01 (d, J = 15.7 Hz, 1H), 2.89 (dd, J = 16.3, 4.4 Hz, 1H), 2.61

(ddd, J = 13.5, 6.2, 1.9 Hz, 1H), 2.52 (dd, J = 16.3, 9.2 Hz, 1H), 1.87 (dd, J = 13.5, 10.5 Hz, 1H), 1.47 (s, 9H), 1.37 (s, 9H); **^{13}C NMR** (101 MHz, $CDCl_3$) δ 178.0, 171.0, 169.8, 157.4, 155. 6, 138.8, 130.2, 129.70, 129.65, 123.0, 118.5, 117.3, 116.8, 81.8, 81.4, 54.9 40.5, 34.7, 34.3, 32.4, 27.9, 27.7; **HRMS (ESI-):** calculated for $C_{28}H_{33}O_7^-$ [M-H]$^-$ 481.2232, found 481.2233.

2-(3,3-bis(tert-butoxycarbonyl)-7-(trifluoromethoxy)-1,2,3,4-tetrahydronaphthalen-1-yl) acetic acid (2d)

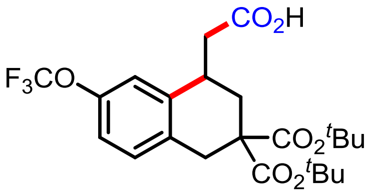

46.0 mg, 49% yield, white solid;

R_f (PE/EA = 1:1) = 0.6 - 0.7;

Mp 79-82 ℃

^{1}H NMR (400 MHz, $CDCl_3$) δ 7.15 (d, J = 8.1 Hz, 1H), 7.05 – 6.96 (m, 2H), 3.41 – 3.31 (m, 1H), 3.26 (dd, J = 16.1, 1.8 Hz, 1H), 3.02 (d, J = 16.2 Hz, 1H), 2.93 (dd, J = 16.3, 4.6 Hz, 1H), 2.68 – 2.53 (m, 2H), 1.88 (dd, J = 13.5, 10.5 Hz, 1H), 1.46 (s, 9H), 1.35 (s, 9H); **^{13}C NMR** (101 MHz, $CDCl_3$) δ 177.8, 170.7, 169.6, 147.8, 139.1, 133.5, 130.2, 120.5 (q, J = 257.7 Hz), 119.1, 118.8, 82.0, 81.6, 54.7, 40.3, 34.8, 34.1, 32.4, 27.8, 27.6; **^{19}F NMR** (376 MHz, $CDCl_3$) δ -57.87; **HRMS (ESI-):** calculated for $C_{23}H_{28}F_3O_7^-$ [M-H]$^-$ 473.1793, found 473.1793.

2-(3,3-bis(tert-butoxycarbonyl)-7-(tert-butyl)-1,2,3,4-tetrahydronaphthalen-1-yl)acetic acid (2e)

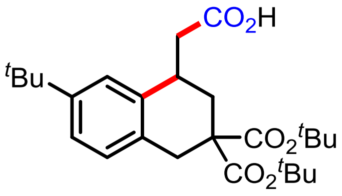

63.4 mg, 71% yield, pale yellow solid;

R_f (PE/EA = 2:1) = 0.5 - 0.6;

Mp 95-98 ℃

^{1}H NMR (400 MHz, $CDCl_3$) δ 7.19 – 7.15 (m, 2H), 7.06 (d, J = 8.8 Hz, 1H), 3.45-3.33 (m, 1H), 3.24 (dd, J = 15.9, 1.8 Hz, 1H), 3.05 – 2.95 (m, 2H), 2.63 (ddd, J = 13.5, 6.1, 1.9 Hz, 1H), 2.55 (dd, J = 16.1, 9.3 Hz, 1H), 1.88 (dd, J = 13.5, 10.2 Hz, 1H), 1.47 (s, 9H), 1.35 (s, 9H), 1.28 (s, 9H); **^{13}C NMR** (101 MHz, $CDCl_3$) δ 178.6, 171.2, 169.9, 149.2, 136.4, 131.5, 128.7, 123.6, 123.0, 81.7, 81.2, 54.8, 41.0, 34.9, 34.7, 34.5, 32.6, 31.4, 27.9, 27.7; **HRMS (ESI-):** calculated for $C_{26}H_{37}O_6^-$ [M-H]$^-$ 445.2596, found 445.2598.

2-(3,3-bis(tert-butoxycarbonyl)-7-isopropyl-1,2,3,4-tetrahydronaphthalen-1-yl)acetic acid (2f)

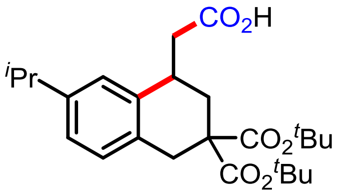

53.6 mg, 62% yield, colorless oil;

R_f (PE/EA = 2:1) = 0.5 - 0.6;

^{1}H NMR (400 MHz, $CDCl_3$) δ 7.07 – 6.98 (m, 3H), 3.48-3.31 (m, 1H), 3.24 (dd, *J* = 15.9, 1.8 Hz, 1H), 3.02 (dd, *J* = 16.0, 5.0 Hz, 2H), 2.84 (p, *J* = 6.9 Hz, 1H), 2.63 (ddd, *J* = 13.4, 6.0, 1.9 Hz, 1H), 2.53 (dd, *J* = 16.1, 9.4 Hz, 1H), 1.87 (dd, *J* = 13.4, 10.3 Hz, 1H), 1.47 (s, 9H), 1.35 (s, 9H), 1.21 (d, *J* = 6.7 Hz, 6H); **^{13}C NMR** (101 MHz, $CDCl_3$) δ 178.6, 171.2, 169.9, 147.0, 136.8, 131.9, 128.9, 124.6, 124.2, 81.7, 81.2, 54.9, 40.9, 35.0, 34.6, 33.9, 32.5, 27.9, 27.7, 24.1, 24.0; **HRMS (ESI-):** calculated for $C_{25}H_{36}O_6^-$ [M-H]$^-$ 431.2439, found 431.2434.

2-(3,3-bis(tert-butoxycarbonyl)-7-fluoro-1,2,3,4-tetrahydronaphthalen-1-yl)acetic acid (2g)

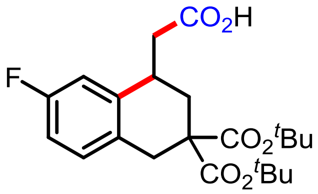

51.6 mg, 63% yield, pale yellow solid;

R_f (PE/EA = 1:1) = 0.6 - 0.7;

Mp 108-111 ℃

^{1}H NMR (400 MHz, $CDCl_3$) δ 7.08 (dd, *J* = 8.4, 5.8 Hz, 1H), 6.92 – 6.79 (m, 2H), 3.40 – 3.30 (m, 1H), 3.23 (d, *J* = 15.2 Hz, 1H), 3.04 – 2.88 (m, 2H), 2.64 – 2.50 (m, 2H), 1.86 (dd, *J* = 13.5, 10.5 Hz, 1H), 1.46 (s, 9H), 1.36 (s, 9H); **^{13}C NMR** (101 MHz, $CDCl_3$) δ 178.0, 170.9, 169.7, 161.5 (d, *J* = 243.6 Hz), 139.2 (d, *J* = 6.6 Hz), 130.3 (d, *J* = 10.2 Hz), 130.2, 113.5 (d, *J* = 21.5 Hz), 112.8 (d, *J* = 21.8 Hz), 81.9, 81.5, 54.8, 40.3, 34.6, 34.2, 32.4, 27.8, 27.7; **^{19}F NMR** (376 MHz, $CDCl_3$) δ -116.22; **HRMS (ESI-):** calculated for $C_{22}H_{28}FO_6^-$ [M-H]$^-$ 407.1875, found 407.1878.

2-(3,3-bis(tert-butoxycarbonyl)-7-chloro-1,2,3,4-tetrahydronaphthalen-1-yl) acetic acid (2h)

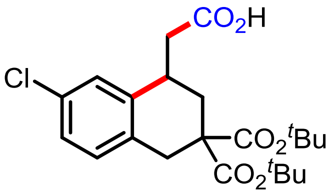

61.5 mg, 73% yield, yellow oil;

R_f (PE/EA = 1:1) = 0.6 - 0.7;

^{1}H NMR (400 MHz, $CDCl_3$) δ 7.18 – 7.14 (m, 1H), 7.13 – 7.04 (m, 2H), 3.38 – 3.28 (m, 1H), 3.22 (d, *J* = 18.0 Hz, 1H),

3.02 – 2.92 (m, 2H), 2.64 – 2.51 (m, 2H), 1.86 (dd, *J* = 13.5, 10.6 Hz, 1H), 1.46 (s, 9H), 1.36 (s, 9H); **^{13}C NMR** (101 MHz, $CDCl_3$) δ 177.9, 170.7, 169.6, 138.9, 133.2, 132.0, 130.2, 126.6, 126.2, 81.9, 81.5, 54.6, 40.2, 34.7, 34.1, 32.2, 27.8, 27.7; **HRMS (ESI-):** calculated for $C_{22}H_{28}ClO_6^-$ $[M-H]^-$ 423.1580, found 423.1579.

2-(3,3-bis(tert-butoxycarbonyl)-7-(diphenylamino)-1,2,3,4-tetrahydronaphthalen-1-yl) acetic acid (2i)

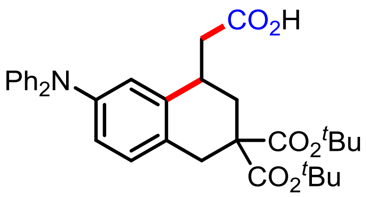

69.0 mg, 62% yield, pale yellow oil;
R_f (PE/EA = 1:1) = 0.5 - 0.6;
^{1}H NMR (400 MHz, $CDCl_3$) δ 7.22 (t, *J* = 7.7 Hz, 4H), 7.08 – 7.02 (m, 4H), 7.00 – 6.86 (m, 5H), 3.38-3.26 (m, 1H), 3.24 (d, *J* = 15.9 Hz, 1H), 3.02 (d, *J* = 15.9 Hz, 1H), 2.78 (dd, *J* = 16.3, 4.3 Hz, 1H), 2.62 (dd, *J* = 13.4, 6.3 Hz, 1H), 2.46 (dd, *J* = 16.4, 9.6 Hz, 1H), 1.88 (dd, *J* = 13.5, 10.2 Hz, 1H), 1.48 (s, 9H), 1.39 (s, 9H); **^{13}C NMR** (101 MHz, $CDCl_3$) δ 178.2, 171.1, 169.9, 147.8, 146.2, 138.1, 129.9, 129.3, 129.1, 123.8, 122.9, 122.5, 122.3, 81.7, 81.3, 55.0, 40.8, 34.9, 34.4, 32.4, 27.9, 27.7; **HRMS (ESI-):** calculated for $C_{34}H_{38}NO_6^-$ $[M-H]^-$ 556.2705, found 556.2702.

Di-tert-butyl-6-(dimethylamino)-4-(2-methoxy-2-oxoethyl)-3,4 dihydronaphthalene-2,2(1H)-dicarboxylate (2j)

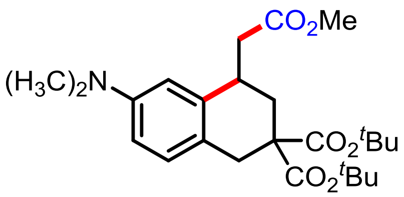

47.8 mg, 54% yield, pale yellow oil;
R_f (PE/EA = 10:1) = 0.2 - 0.3;
^{1}H NMR (400 MHz, $CDCl_3$) δ 6.98 (d, *J* = 8.4 Hz, 1H), 6.60 – 6.56 (m, 1H), 6.50 (d, *J* = 2.4 Hz, 1H), 3.71 (s, 3H), 3.42 – 3.30 (m, 1H), 3.14 (dd, *J* = 15.6, 1.6 Hz, 1H), 2.97 – 2.90 (m, 2H), 2.88 (s, 6H), 2.58 – 2.43 (m, 2H), 1.81 (dd, *J* = 13.5, 10.2 Hz, 1H), 1.45 (s, 9H), 1.36 (s, 9H); **^{13}C NMR** (101 MHz, $CDCl_3$) δ 173.0, 171.3, 170.0, 149.5, 137.8, 129.5, 122.9, 111.8, 110.6, 81.4, 81.0, 55.0, 51.6, 41.3, 40.9, 34.8, 34.5, 33.0, 27.9, 27.7; **HRMS (ESI+):** calculated for $C_{25}H_{38}NO_6^+$ $[M+H]^+$ 448.2694, found 448.2691.

Di-tert-butyl-4-(2-methoxy-2-oxoethyl)-6-morpholino-3,4-dihydronaphthalene-2,2(1H)-dicarboxylate (2k)

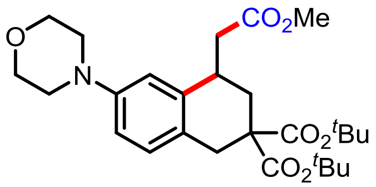

62.6 mg, 64% yield, pale yellow solid; R_f (PE/EA = 10:1) = 0.2 - 0.3; Mp 82-84 ℃ **^{1}H NMR** (400 MHz, $CDCl_3$) δ 7.02 (d, *J* = 8.0 Hz, 1H), 6.75 – 6.66 (m, 2H), 3.85 – 3.81 (m, 4H), 3.70 (s, 3H), 3.41 – 3.28 (m, 1H), 3.15 (dd, *J* = 16.0, 1.2 Hz, 1H), 3.11 – 3.04 (m, 4H), 2.96 (dd, *J* = 15.6 Hz, 1H), 2.87 (dd, *J* = 15.6, 4.8 Hz, 1H), 2.57 – 2.45 (m, 2H), 1.82 (dd, *J* = 13.5, 10.2 Hz, 1H), 1.44 (s, 9H), 1.35 (s, 9H); **^{13}C NMR** (101 MHz, $CDCl_3$) δ 172.9, 171.2, 169.9, 150.0, 138.1, 129.6, 126.4, 114.6, 113.8, 81.5, 81.1, 67.0, 54.9, 51.7, 49.8, 41.1, 34.7, 34.6, 33.0, 27.9, 27.7; **HRMS (ESI+):** calculated for $C_{27}H_{39}NO_7^+$ $[M+H]^+$ 490.2799, found 490.2802.

2-(3,3-bis(tert-butoxycarbonyl)-7-(methylthio)-1,2,3,4-tetrahydronaphthalen-1-yl)acetic acid (2l)

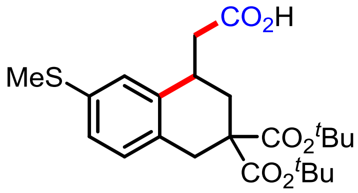

56.0 mg, 64% yield, pale yellow solid; R_f (PE/EA = 2:1) = 0.5 - 0.6; Mp 107-110 ℃ **^{1}H NMR** (400 MHz, $CDCl_3$) δ 7.08 (s, 1H), 7.06 (d, *J* = 0.8 Hz, 2H), 3.39 – 3.29 (m, 1H), 3.22 (dd, *J* = 16.0, 1.2 Hz, 1H), 3.03 – 2.93 (m, 2H), 2.65 – 2.50 (m, 2H), 2.44 (s, 3H), 1.87 (dd, *J* = 13.5, 10.5 Hz, 1H), 1.46 (s, 9H), 1.36 (s, 9H); **^{13}C NMR** (101 MHz, $CDCl_3$) δ 178.3, 171.0, 169.8, 137.8, 136.0, 131.9, 129.5, 125.4, 124.9, 81.8, 81.4, 54.7, 40.5, 34.9, 34.4, 32.3, 27.9, 27.7, 16.3; **HRMS (ESI-):** calculated for $C_{23}H_{32}SO_6^-$ $[M-H]^-$ 435.1847, found 435.1846.

2-(3,3-bis(tert-butoxycarbonyl)-7-(diethylcarbamoyl)-1,2,3,4-tetrahydronaphthalen-1-yl)acetic acid (2m)

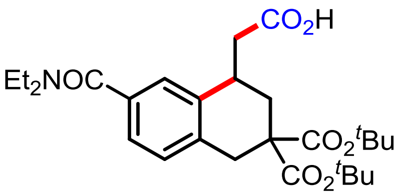

51.2 mg, 52% yield, brown oil; R_f (PE/EA = 1:1) = 0.1 - 0.2; **^{1}H NMR** (400 MHz, $CDCl_3$) δ 7.19 (s, 1H), 7.15 – 7.08 (m, 2H), 3.51 (s, 2H), 3.41 – 3.30 (m, 1H), 3.28 – 3.13

(m, 3H), 3.04 (d, J = 15.6 Hz, 1H), 2.93 (dd, J = 16.4, 4.4 Hz, 1H), 2.65 – 2.48 (m, 2H), 1.86 (dd, J = 13.5, 10.4 Hz, 1H), 1.45 (s, 9H), 1.34 (s, 9H), 1.24 – 1.03 (m, 6H); **^{13}C NMR** (101 MHz, $CDCl_3$) δ 176.6, 171.6, 170.8, 169.7, 137.8, 136.2, 135.0, 128.9, 124.5, 124.3, 81.8, 81.4, 54.8, 43.4, 40.3, 39.5, 35.2, 34.4, 32.3, 27.8, 27.7, 14.2, 12.9; **HRMS (ESI-):** calculated for $C_{27}H_{38}NO_7^-$ [M-H]$^-$ 488.2654, found 488.2654.

2-(3,3-bis(tert-butoxycarbonyl)-7-phenyl-1,2,3,4-tetrahydronaphthalen-1-yl)acetic acid (2n)

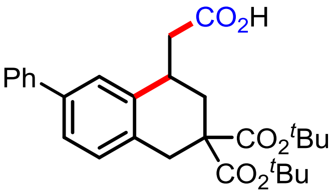

56.9 mg, 61% yield, pale yellow solid;

R_f (PE/EA = 1:1) = 0.6 - 0.7;

Mp 118-121 ℃

^{1}H NMR (400 MHz, $CDCl_3$) δ 7.62 – 7.56 (m, 2H), 7.49 – 7.40 (m, 4H), 7.38 – 7.32 (m, 1H), 7.26 (t, J = 8.2 Hz, 1H), 3.54 – 3.43 (m, 1H), 3.36 (dd, J = 16.3, 1.8 Hz, 1H), 3.13 (dd, J = 16.5, 4.0 Hz, 2H), 2.72 (ddd, J = 13.4, 6.0, 1.9 Hz, 1H), 2.64 (dd, J = 16.2, 9.4 Hz, 1H), 1.98 (dd, J = 13.5, 10.4 Hz, 1H), 1.52 (s, 9H), 1.42 (s, 9H); **^{13}C NMR** (101 MHz, $CDCl_3$) δ 178.6, 171.1, 169.9, 141.1, 139.5, 137.4, 133.9, 129.5, 128.7, 127.15, 127.07, 125.4, 125.0, 81.8, 81.4, 54.8, 40.7, 35.1, 34.6, 32.5, 27.9, 27.7; **HRMS (ESI-):** calculated for $C_{28}H_{33}O_6^-$ [M-H]$^-$ 465.2283, found 465.2282.

2-(3,3-bis(tert-butoxycarbonyl)-5-phenyl-1,2,3,4-tetrahydronaphthalen-1-yl)acetic acid (2o)

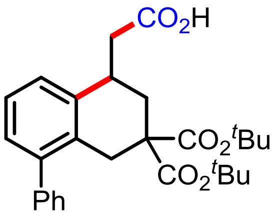

57.0 mg, 61% yield, pale yellow solid;

R_f (PE/EA = 2:1) = 0.3 - 0.4;

Mp 129-132 ℃

^{1}H NMR (400 MHz, $CDCl_3$) δ 7.44 – 7.38 (m, 2H), 7.39 – 7.30 (m, 3H), 7.25 – 7.16 (m, 2H), 7.11 (dd, J = 6.8, 1.6 Hz, 1H), 3.60 – 3.49 (m, 1H), 3.18 (dd, J = 16.4, 1.6 Hz, 1H), 3.06 – 2.96 (m, 2H), 2.69 – 2.57 (m, 2H), 1.90 (dd, J = 13.5, 10.0 Hz, 1H), 1.36 (s, 9H), 1.27 (s, 9H); **^{13}C NMR** (101 MHz, $CDCl_3$) δ 178.3, 171.2, 169.9, 142.1, 141.3, 138.3, 132.1, 129.4, 128.1, 128.0, 126.9, 126.2, 125.6, 81.7, 81.1, 55.4, 41.5, 34.8, 33.2, 33.0, 27.8, 27.6; **HRMS (ESI-):** calculated for $C_{32}H_{42}NaO_8^-$ [M-H]$^-$ 465.2283, found 465.2283.

2-(3,3-bis(tert-butoxycarbonyl)-5-(trifluoromethoxy)-1,2,3,4-tetrahydronaphthalen-1-yl)acetic acid (2p)

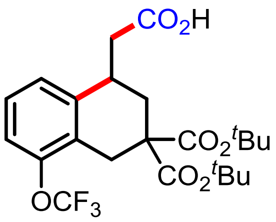

64.5 mg, 68% yield, pale yellow oil;

R_f (PE/EA = 3:1) = 0.2 - 0.3;

^{1}H NMR (400 MHz, $CDCl_3$) δ 7.22 – 7.16 (m, 1H), 7.16 – 7.06 (m, 2H), 3.48 – 3.36 (m, 2H), 2.96 (dd, *J* = 16.4, 4.4 Hz, 1H), 2.89 (d, *J* = 16.8 Hz, 1H), 2.64 – 2.55 (m, 2H), 1.89 (dd, *J* = 13.5, 10.7 Hz, 1H), 1.47 (s, 9H), 1.35 (s, 9H); **^{13}C NMR** (101 MHz, $CDCl_3$) δ 178.1, 170.8, 169.5, 147.3, 139.9, 128.0, 127.0, 124.6, 120.7 (q, *J* = 258.3 Hz), 118.3, 82.1, 81.6, 54.2, 40.6, 34.1, 32.2, 28.9, 27.8, 27.6; **HRMS (ESI-):** calculated for $C_{23}H_{28}F_3O_7^-$ $[M-H]^-$ 473.1793, found 473.1795.

2-(3,3-bis(ethoxycarbonyl)-7-methoxy-1,2,3,4-tetrahydronaphthalen-1-yl)acetic acid (2q)

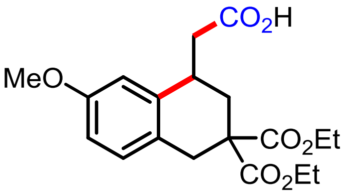

54.0 mg, 74% yield, pale yellow solid;

R_f (PE/EA = 1:1) = 0.5 - 0.6;

Mp 107-110 ℃

^{1}H NMR (400 MHz, $CDCl_3$) δ 7.05 (d, *J* = 8.7 Hz, 1H), 6.74 – 6.68 (m, 2H), 4.24 – 4.16 (m, 2H), 4.16 – 4.06 (m, 2H), 3.76 (s, 3H), 3.47 – 3.36 (m, 1H), 3.27 (dd, *J* = 16.0, 1.6 Hz, 1H), 3.12 (d, *J* = 16.0 Hz, 1H), 2.93 (dd, *J* = 16.4, 5.6 Hz, 1H), 2.71 (ddd, *J* = 13.6, 6.4, 1.9 Hz, 1H), 2.56 (dd, *J* = 16.2, 9.0 Hz, 1H), 1.97 (dd, *J* = 13.6, 10.1 Hz, 1H), 1.26 (t, *J* = 7.2 Hz, 3H), 1.16 (t, *J* = 7.2 Hz, 3H); **^{13}C NMR** (101 MHz, $CDCl_3$) δ 177.6, 171.7, 170.7, 158.3, 138.1, 123.0, 126.2, 112.4, 111.8, 61.7, 61.4, 55.2, 53.8, 40.7, 34.5, 34.4, 32.5, 14.0, 14.0; **HRMS (ESI-):** calculated for $C_{19}H_{23}O_7^-$ $[M-H]^-$ 363.1449, found 363.1448.

2-(7-methoxy-3,3-bis(methoxycarbonyl)-1,2,3,4-tetrahydronaphthalen-1-yl)acetic acid (2r)

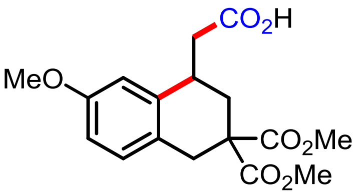

54.4 mg, 81% yield, pale yellow solid;

R_f (PE/EA = 1:1) = 0.4 - 0.5;

Mp 110-113 ℃

^{1}**H NMR** (400 MHz, $CDCl_3$) δ 7.04 (d, *J* = 8.4 Hz, 1H), 6.74 – 6.67 (m, 2H), 3.76 (s, 3H), 3.74 (s, 3H), 3.66 (s, 3H), 3.45 – 3.37 (m, 1H), 3.27 (dd, *J* = 12.0, 1..2 Hz, 1H), 3.15 (d, *J* = 16.0 Hz, 1H), 2.92 (dd, *J* = 16.4, 4.4 Hz, 1H), 2.72 (ddd, *J* = 13.6, 6.4, 1.9 Hz, 1H), 2.56 (dd, *J* = 16.2, 8.9 Hz, 1H), 1.99 (dd, *J* = 13.6, 10.1 Hz, 1H); ^{13}**C NMR** (101 MHz, $CDCl_3$) δ 178.1, 172.1, 171.2, 158.4, 137.9, 130.0, 125.9, 112.5, 111.8, 55.2, 53.8, 52.9, 52.8, 40.8, 34.50, 34.45, 32.5; **HRMS (ESI-):** calculated for $C_{17}H_{19}O_7^-$ [M-H]$^-$ 335.1136, found 335.1135.

2-(3,3-bis(ethoxycarbonyl)-7-phenyl-1,2,3,4-tetrahydronaphthalen-1-yl)acetic acid (2s)

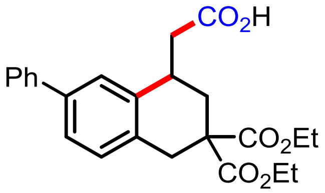

51.0 mg, 62% yield, pale yellow solid;

R_f (PE/EA = 1:1) = 0.3 - 0.4;

Mp 125-128 ℃

^{1}**H NMR** (400 MHz, $CDCl_3$) δ 7.59 – 7.54 (m, 2H), 7.46 – 7.36 (m, 4H), 7.36 – 7.28 (m, 1H), 7.22 (d, *J* = 8.4 Hz, 1H), 4.28 – 4.17 (m, 2H), 4.18 – 4.07 (m, 2H), 3.57 – 3.44 (m, 1H), 3.39 (dd, *J* = 16.1, 2.0 Hz, 1H), 3.24 (d, *J* = 16.0 Hz, 1H), 3.04 (dd, *J* = 16.2, 4.5 Hz, 1H), 2.77 (ddd, *J* = 13.6, 6.3, 1.9 Hz, 1H), 2.62 (dd, *J* = 16.2, 9.2 Hz, 1H), 2.05 (dd, *J* = 13.6, 10.1 Hz, 1H), 1.28 (t, *J* = 7.1 Hz, 3H), 1.18 (t, *J* = 7.1 Hz, 3H); ^{13}**C NMR** (101 MHz, $CDCl_3$) δ 177.7, 171.7, 170.6, 141.0, 139.7, 137.2, 133.2, 129.6, 128.8, 127.2, 127.0, 125.5, 125.2, 61.8, 61.5, 53.7, 40.7, 35.0, 34.4, 32.4, 14.04, 13.96; **HRMS (ESI-):** calculated for $C_{24}H_{25}O_6^-$ [M-H]$^-$ 409.1657, found 409.1657.

2-(3,3-bis(isopropoxycarbonyl)-7-phenyl-1,2,3,4-tetrahydronaphthalen-1-yl)acetic acid (2t)

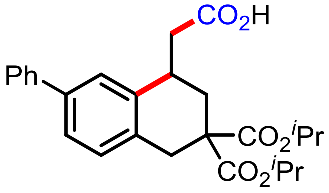

46.3 mg, 53% yield, pale yellow solid;

R_f (PE/EA = 1:1) = 0.5 - 0.6;

Mp 119-122 ℃

^{1}**H NMR** (400 MHz, $CDCl_3$) δ 7.56 – 7.51 (m, 2H), 7.45 – 7.35 (m, 4H), 7.36 – 7.28 (m, 1H), 7.21 (d, *J* = 7.8 Hz, 1H), 5.14 – 5.01 (m, 1H), 5.04 – 4.92 (m, 1H), 3.55 – 3.41 (m, 1H), 3.38 (dd, *J* = 16.0, 2.1 Hz, 1H), 3.17 (d, *J* = 16.0 Hz, 1H), 3.06 (dd, *J* = 16.2, 4.4 Hz, 1H), 2.75 (ddd, *J* = 13.6, 6.2, 2.0 Hz, 1H), 2.62 (dd, *J* = 16.2, 9.2 Hz, 1H), 2.00 (dd, *J* = 13.5, 10.4 Hz, 1H), 1.27 (d, *J* = 1.8 Hz, 3H), 1.25

(d, J = 1.8 Hz, 3H), 1.20 (d, J = 6.3 Hz, 3H), 1.13 (d, J = 6.2 Hz, 3H); **^{13}C NMR** (101 MHz, $CDCl_3$) δ 177.9, 171.3, 170.1, 141.1, 139.7, 137.3, 133.5, 129.5, 128.7, 127.2, 127.1, 125.4, 125.1, 69.2, 68.9, 53.7, 40.7, 35.0, 34.5, 32.4, 21.6, 21.59, 21.54, 21.4; **HRMS (ESI-):** calculated for $C_{26}H_{29}O_6^-$ [M-H]$^-$ 437.1970, found 437.1970.

2-(7-phenyl-1,2,3,4-tetrahydronaphthalen-1-yl)acetic acid (2u)

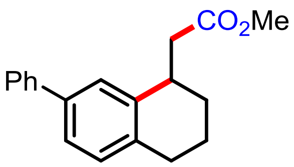

20.7 mg, 37% yield, colorless oil;

R_f (PE/EA = 10:1) = 0.5 - 0.6;

^{1}H NMR (400 MHz, $CDCl_3$) δ 7.59 – 7.53 (m, 2H), 7.47 – 7.30 (m, 5H), 7.16 (d, J = 7.8 Hz, 1H), 3.73 (s, 3H), 3.51 – 3.36 (m, 1H), 2.87 – 2.75 (m, 3H), 2.61 (dd, J = 15.3, 10.0 Hz, 1H), 2.04 – 1.70 (m, 4H); **^{13}C NMR** (101 MHz, $CDCl_3$) δ 173.3, 141.2, 139.6, 138.9, 136.3, 129.8, 128.7, 127.1, 127.1, 127.0, 124.9, 51.6, 41.9, 34.8, 29.3, 28.1, 19.5; **HRMS (ESI+):** calculated for $C_{19}H_{20}O_2Na^+$ [M+Na]$^+$ 303.1365, found 303.1359.

2-(3,3-bis(tert-butoxycarbonyl)-7-(tert-butyl)-1,2,3,4-tetrahydronaphthalen-1-yl)acetic acid (2v)

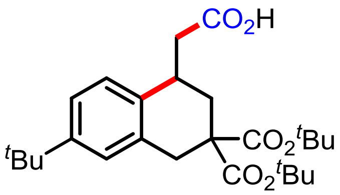

65.0 mg, 73% yield, pale yellow oil;

R_f (PE/EA = 1:1) = 0.7 - 0.8;

^{1}H NMR (400 MHz, $CDCl_3$) δ 7.22 – 7.16 (m, 1H), 7.15 – 7.08 (m, 2H), 3.38 – 3.25 (m, 2H), 3.08 – 2.96 (m, 2H), 2.61 (ddd, J = 13.4, 5.9, 1.9 Hz, 1H), 2.52 (dd, J = 16.2, 9.2 Hz, 1H), 1.88 (dd, J = 13.4, 10.7 Hz, 1H), 1.47 (s, 9H), 1.36 (s, 9H), 1.30 (s, 9H); **^{13}C NMR** (101 MHz, $CDCl_3$) δ 178.5, 171.1, 169.9, 149.1, 134.1, 134.0, 125.8, 125.7, 123.6, 81.6, 81.2, 54.9, 40.6, 35.6, 34.6, 34.3, 31.9, 31.3, 27.9, 27.7; **HRMS (ESI-):** calculated for $C_{26}H_{38}O_6^-$ [M-H]$^-$ 445.2596, found 445.2596.

2-(3,3-bis(tert-butoxycarbonyl)-6,8-dimethoxy-1,2,3,4-tetrahydronaphthalen-1-yl)acetic acid (2w)

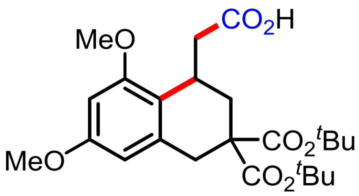

69.2 mg, 77% yield, pale yellow solid;

R_f (PE/EA = 1:1) = 0.6 - 0.7;

Mp 117-120 ℃

^{1}H NMR (400 MHz, $CDCl_3$) δ 6.28 (d, *J* = 2.5 Hz, 1H), 6.26 (d, *J* = 2.4 Hz, 1H), 3.76 (s, 3H), 3.75 (s, 3H), 3.65 – 3.55 (m, 1H), 3.12 (d, *J* = 15.7 Hz, 1H), 3.04 – 3.00 (m, 1H), 2.99 – 2.94 (m, 1H), 2.64 (ddd, *J* = 13.9, 7.8, 1.9 Hz, 1H), 2.33 (dd, *J* = 16.3, 9.5 Hz, 1H), 1.97 (dd, *J* = 13.9, 6.9 Hz, 1H), 1.47 (s, 9H), 1.30 (s, 9H); **^{13}C NMR** (101 MHz, $CDCl_3$) δ 179.1, 171.3, 169.8, 158.9, 158.1, 136.8, 118.3, 104.5, 97.0, 81.9, 81.0, 55.2, 55.1, 54.8, 40.1, 36.3, 34.4, 28.4, 27.8, 27.7; **HRMS (ESI-):** calculated for $C_{24}H_{33}O_8^-$ $[M-H]^-$ 449.2181, found 449.2179.

2-(3,3-bis(tert-butoxycarbonyl)-6-methoxy-2,3-dihydro-1H-inden-1-yl)acetic acid (2x)

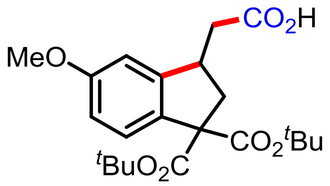

53.1 mg, 65% yield, pale yellow oil;

R_f (PE/EA = 1:1) = 0.1 - 0.2;

^{1}H NMR (400 MHz, $CDCl_3$) δ 7.46 (d, *J* = 8.4 Hz, 1H), 6.83 (dd, *J* = 8.8, 2.4 Hz, 1H), 6.73 (d, *J* = 2.0 Hz, 1H), 3.79 (s, 3H), 3.74 – 3.64 (m, 1H), 2.96 (dd, *J* = 13.6, 8.0 Hz, 1H), 2.84 (dd, *J* = 16.0, 5.2 Hz, 1H), 2.58 (dd, *J* = 16.4, 9.6 Hz, 1H), 2.36 (dd, *J* = 13.6, 6.4 Hz, 1H), 1.48 (s, 9H), 1.45 (s, 9H); **^{13}C NMR** (101 MHz, $CDCl_3$) δ 178.0, 170.1, 169.8, 160.4, 147.4, 131.6, 127.6, 113.6, 108.6, 81.7, 81.6, 65.4, 55.4, 40.3, 40.0, 39.1, 27.88, 27.85; **HRMS (ESI-):** calculated for $C_{22}H_{29}O_7^-$ $[M-H]^-$ 405.1919, found 405.1921.

Di-tert-butyl 5-(dimethylamino)-3-(2-methoxy-2-oxoethyl)-2,3-dihydro-1H-indene-1,1-dicarboxylate (2y)

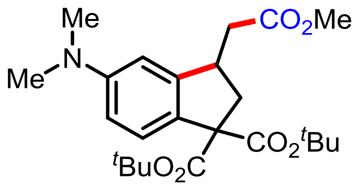

43.0 mg, 50% yield, pale yellow oil;

R_f (PE/EA = 5:1) = 0.4 - 0.5;

^{1}H NMR (400 MHz, $CDCl_3$) δ 7.39 (d, *J* = 8.0 Hz, 1H), 6.72 – 6.64 (m, 1H), 6.50 (s, 1H), 3.72 (s, 3H), 3.71 – 3.58 (m, 1H), 2.93 (s, 6H), 2.88 (dd, *J* = 13.7, 8.2 Hz, 1H), 2.79 (dd, *J* = 15.6, 5.2 Hz, 1H), 2.52 (dd, *J* = 15.6, 9.6 Hz, 1H), 2.32 (dd, *J* = 13.7, 6.2 Hz, 1H), 1.47 (s, 9H), 1.44 (s, 9H); **^{13}C NMR** (101 MHz, $CDCl_3$) δ 173.2, 170.4, 170.2, 151.2, 147.2, 129.1, 127.0, 112.3, 107.1, 81.33, 81.30, 65.3, 51.6, 40.8, 40.4, 40.2, 39.5, 27.90, 27.88; **HRMS (ESI+):** calculated for $C_{24}H_{36}NO_6^+$ $[M+H]^+$ 434.2537, found 434.2532.

2-(3,3-bis(tert-butoxycarbonyl)-6-fluoro-2,3-dihydro-1H-inden-1-yl)acetic acid (2z)

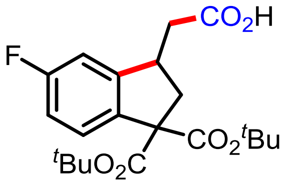

49.6 mg, 63% yield, colorless oil;

R_f (PE/EA = 1:1) = 0.4 - 0.5;

^{1}H NMR (400 MHz, $CDCl_3$) δ 7.52 (dd, *J* = 8.8, 5.6 Hz, 1H), 6.98 (td, *J* = 8.6, 2.2 Hz, 1H), 6.90 (dd, *J* = 8.9, 2.6 Hz, 1H), 3.72 (p, *J* = 7.8 Hz, 1H), 2.99 (dd, *J* = 13.6, 8.0 Hz, 1H), 2.81 (dd, *J* = 16.4, 5.6 Hz, 1H), 2.60 (dd, *J* = 16.4, 9.2 Hz, 1H), 2.37 (dd, *J* = 13.6, 6.4 Hz, 1H), 1.48 (s, 9H), 1.44 (s, 9H); **^{13}C NMR** (101 MHz, $CDCl_3$) δ 177.8, 169.6, 169.3, 163.4 (d, *J* = 246.6 Hz), 148.1 (d, *J* = 8.0 Hz), 135.0 (d, *J* = 2.4 Hz), 128.2 (d, *J* = 9.0 Hz), 114.6 (d, *J* = 22.9 Hz), 110.6 (d, *J* = 22.6 Hz), 82.05, 81.97, 65.4, 40.4, 39.7, 38.9, 27.9, 27.8; **^{19}F NMR** (376 MHz, $CDCl_3$) δ -113.62; **HRMS (ESI-):** calculated for $C_{21}H_{26}FO_6^-$ $[M-H]^-$ 393.1719, found 393.1716.

2-(7-(benzyloxy)-3,3-bis(tert-butoxycarbonyl)-1-methyl-1,2,3,4-tetrahydronaphthalen-1-yl)acetic acid (2aa)

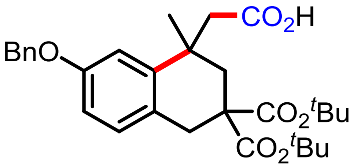

51.2 mg, 50% yield, pale yellow oil;

R_f (PE/EA = 1:1) = 0.7 - 0.8;

Mp 120-123 ℃

^{1}H NMR (400 MHz, $CDCl_3$) δ 7.86 (d, *J* = 7.7 Hz, 2H), 7.59 – 7.49 (m, 1H), 7.50 – 7.42 (m, 2H), 7.20 (d, *J* = 8.5 Hz, 1H), 7.03 (d, *J* = 2.7 Hz, 1H), 6.88 (dd, *J* = 8.5, 2.8 Hz, 1H), 5.96 (s, 2H), 3.79 (s, 3H), 2.83 (t, *J* = 7.8 Hz, 2H), 2.38 (t, *J* = 7.3 Hz, 2H), 2.09 – 1.91 (m, 2H); **^{13}C NMR** (101 MHz, $CDCl_3$) δ 176.7, 171.3, 170.7, 157.6, 142.8, 137.1, 129.7, 128.6, 127.9, 127.6, 126.5, 112.9, 112.6, 81.8, 81.5, 70.1, 54.1, 39.1, 36.2, 34.7, 30.3, 27.8; **HRMS (ESI-):** calculated for $C_{30}H_{37}O_7^-$ $[M-H]^-$ 509.2545, found 509.2545.

2-(3,3-bis(tert-butoxycarbonyl)-7-(tert-butyl)-1-methyl-1,2,3,4-tetrahydronaphthalen-1-yl)acetic acid (2ab)

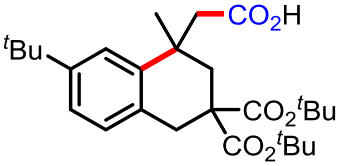

51.5 mg, 56% yield, pale yellow oil;

R_f (PE/EA = 5:1) = 0.2 - 0.3;

Mp 106-109 ℃

^{1}H NMR (400 MHz, $CDCl_3$) δ 7.24 (d, *J* = 1.6 Hz, 1H), 7.16 (dd, *J* = 8.0, 2.0 Hz, 1H), 7.05 (d, *J* = 8.4 Hz, 1H), 3.16 (d, *J* = 15.8 Hz, 1H), 3.03 (d, *J* = 16.0 Hz, 1H), 2.72 – 2.57 (m, 3H), 2.25 (d, *J* = 14.8 Hz, 1H), 1.44 (s, 12H), 1.40 (s, 9H), 1.28 (s, 9H); **^{13}C NMR** (101 MHz, $CDCl_3$) δ 176.9, 171.4, 170.8, 149.2, 140.8, 130.6, 128.4, 123.5, 122.5, 81.8, 81.5, 53.9, 47.6, 39.5, 36.3, 35.0, 34.5, 31.4, 30.1, 27.8; **HRMS (ESI-):** calculated for $C_{27}H_{39}O_6^-$ [M-H]$^-$ 459.2752, found 459.2752.

2-(3,3-bis(tert-butoxycarbonyl)-1-methyl-7-phenyl-1,2,3,4-tetrahydronaphthalen-1-yl)acetic acid (2ac)

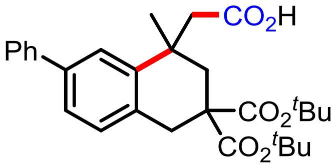

46.4 mg, 48% yield, pale yellow solid;

R_f (PE/EA = 1:1) = 0.5 - 0.6;

Mp 143-146 ℃

^{1}H NMR (400 MHz, $CDCl_3$) δ 7.58 – 7.52 (m, 2H), 7.47 – 7.39 (m, 3H), 7.39 – 7.29 (m, 2H), 7.21 (d, *J* = 7.6 Hz, 1H), 3.25 (d, *J* = 16.0 Hz, 1H), 3.10 (d, *J* = 15.6 Hz, 1H), 2.79 – 2.69 (m, 3H), 2.30 (d, *J* = 14.8 Hz, 1H), 1.48 (s, 3H), 1.46 (s, 9H), 1.42 (s, 9H); **^{13}C NMR** (101 MHz, $CDCl_3$) δ 176.6, 171.2, 170.7, 141.8, 141.3, 139.6, 133.1, 129.3, 128.7, 127.09, 127.07, 125.4, 124.7, 81.9, 81.6, 53.9, 47.5, 39.3, 36.2, 35.3, 30.5, 27.8; **HRMS (ESI-):** calculated for $C_{29}H_{35}O_6^-$ [M-H]$^-$ 479.2439, found 479.2438.

2-(3,3-bis(tert-butoxycarbonyl)-6,8-dimethoxy-1-methyl-1,2,3,4-tetrahydronaphthalen-1-yl)acetic acid (2ad)

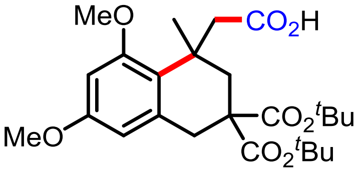

58.1 mg, 63% yield, pale yellow solid;

R_f (PE/EA = 1:1) = 0.4 - 0.5;

Mp 140-143 ℃

^{1}H NMR (400 MHz, $CDCl_3$) **^{1}H NMR** (400 MHz, $CDCl_3$) δ 6.28 (s, 2H), 3.76 (s, 3H), 3.74 (s, 3H), 3.16 (dd, *J* = 16.0, 2.0 Hz, 1H), 3.01 – 2.81 (m, 3H), 2.68 (d, *J* = 14.4 Hz, 1H), 2.18 (dd, *J* = 14.3, 2.0 Hz, 1H), 1.46 (s, 9H), 1.41 (s, 9H), 1.40 (s, 3H); **^{13}C NMR** (101 MHz, $CDCl_3$) δ 177.4, 171.1, 170.8, 159.0, 158.5, 136.8, 121.2, 104.5, 98.0, 81.6, 81.5, 55.03, 54.99, 53.2, 44.7, 41.6, 36.8, 35.7, 27.8, 27.5; **HRMS (ESI-):** calculated for $C_{25}H_{35}O_8^-$ [M-H]$^-$ 463.2337, found 463.2338.

Tert-butyl 3-(2-methoxy-2-oxoethyl)-3-methyl-5-phenylindoline-1-carboxylate (4a)

50.5 mg, 66% yield, pale yellow solid;

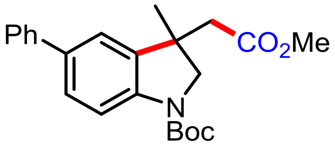

R_f (PE/EA = 10:1) = 0.6 - 0.7;

Mp 100-103 ℃

^{1}H NMR (400 MHz, CD_3CN) δ 7.78 (s, 1H), 7.63 – 7.55 (m, 2H), 7.50 – 7.39 (m, 4H), 7.33 – 7.28 (m, 1H), 4.12 (d, *J* = 11.6 Hz, 1H), 3.76 (d, *J* = 11.2 Hz, 1H), 3.57 (s, 3H), 2.79 – 2.63 (m, 2H), 1.55 (s, 9H), 1.40 (s, 3H); **^{13}C NMR** (101 MHz, CD_3CN) δ 172.1, 152.9, 142.3, 141.4, 139.9, 135.8, 129.5, 127.6, 127.4, 127.2, 122.0, 115.4, 81.3, 60.7, 51.7, 44.7, 41.9, 28.3, 26.9; **HRMS (ESI+):** calculated for $C_{23}H_{27}NO_4Na^+$ $[M+Na]^+$ 404.1832, found 404.1827.

.

Tert-butyl 5-(tert-butyl)-3-(2-methoxy-2-oxoethyl)-3-methylindoline -1-carboxylate (4b)

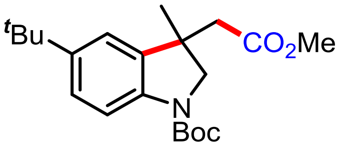

42.0 mg, 58% yield, pale yellow solid;

R_f (PE/EA = 10:1) = 0.4 - 0.5;

Mp 59-62 ℃

^{1}H NMR (400 MHz, CD_3CN) δ 7.59 (s, 1H), 7.23 – 7.19 (m, 2H), 4.06 (d, *J* = 11.2 Hz, 1H), 3.69 (d, *J* = 11.2 Hz, 1H), 3.56 (s, 3H), 2.73 – 2.55 (m, 2H), 1.53 (s, 9H), 1.36 (s, 3H), 1.28 (s, 9H); **^{13}C NMR** (101 MHz, CD_3CN) δ 172.1, 153.0, 146.1, 140.4, 138.70, 125.4, 120.5, 114.5, 81.3, 60.7, 51.7, 44.8, 42.1, 34.8, 31.6, 28.4, 26.7; **HRMS (ESI+):** calculated for $C_{21}H_{31}NO_4Na^+$ $[M+Na]^+$ 384.2145, found 384.2137.

Tert-butyl 3-(2-methoxy-2-oxoethyl)-3-methylindoline-1-carboxylate (4c)

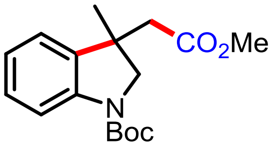

26.1 mg, 43% yield, colorless oil;

R_f (PE/EA = 10:1) = 0.4 - 0.5;

^{1}H NMR (400 MHz, CD_3CN) δ 7.72 (s, 1H), 7.20 – 7.14 (m, 2H), 6.96 (td, *J* = 7.5, 1.1 Hz, 1H), 4.08 (d, *J* = 11.6 Hz, 1H), 3.71 (d, *J* = 11.2 Hz, 1H), 3.56 (s, 3H), 2.65 (q, *J* = 14.8 Hz, 2H), 1.53 (s, 9H), 1.35 (s, 3H); **^{13}C NMR** (101 MHz, CD_3CN) δ 171.9, 152.8, 142.3, 138.8, 128.5, 123.2, 122.8, 114.9, 100.5, 81.0, 60.3, 51.5, 44.6, 41.6, 28.1, 26.7; **HRMS (ESI+):** calculated for $C_{17}H_{23}NO_4Na^+$ $[M+Na]^+$ 328.1519 found 328.1505

Tert-butyl 5-chloro-3-(2-methoxy-2-oxoethyl)-3-methylindoline-1-carboxylate (4d)

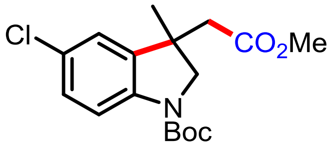

47.3 mg, 70% yield, pale yellow oil;

R_f (PE/EA = 10:1) = 0.2 - 0.3;

^{1}H NMR (400 MHz, CD_3CN) δ 7.68 (s, 1H), 7.20 – 7.13 (m, 2H), 4.08 (d, *J* = 11.6 Hz, 1H), 3.73 (d, *J* = 11.6 Hz, 1H), 3.57 (s, 3H), 2.72 – 2.59 (m, 2H), 1.53 (s, 9H), 1.35 (s, 3H); **^{13}C NMR** (101 MHz, CD_3CN) δ 171.7, 152.6, 141.2, 128.5, 128.2, 127.0, 123.6, 116.0, 81.3, 60.5, 51.6, 44.4, 41.8, 28.1, 26.7; **HRMS (ESI+):** calculated for $C_{17}H_{22}ClNO_4Na^+$ $[M+Na]^+$ 362.1130, found 362.1127.

Tert-butyl 5-fluoro-3-(2-methoxy-2-oxoethyl)-3-methylindoline-1-carboxylate (4e)

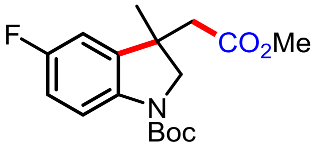

34.9 mg, 54% yield, pale yellow oil;

R_f (PE/EA = 10:1) = 0.3 - 0.4;

^{1}H NMR (400 MHz, CD_3CN) δ 7.70 (s, 1H), 6.96 (dd, *J* = 8.8, 2.8 Hz, 1H), 6.90 (td, *J* = 9.0, 2.8 Hz, 1H), 4.09 (d, *J* = 11.6 Hz, 1H), 3.73 (d, *J* = 11.6 Hz, 1H), 3.57 (s, 3H), 2.72 – 2.59 (m, 2H), 1.53 (s, 9H), 1.36 (s, 3H); **^{13}C NMR** (101 MHz, CD_3CN) δ 171.9, 159.3 (d, *J* = 238.1 Hz), 152.9, 141.1, 115.9, 114.6 (d, *J* = 23.2 Hz), 110.7 (d, *J* = 24.4 Hz), 81.1, 60.6, 51.7, 44.5, 41.8, 28.3, 26.7; **^{19}F NMR** (376 MHz, CD_3CN) δ -123.08; **HRMS (ESI+):** calculated for $C_{19}H_{20}NO_3Na^+$ $[M+Na]^+$ 346.1425, found 346.1421.

4-(2-(benzamido(carboxy)methyl)-4,5-dimethoxyphenyl)butanoic acid (4f)

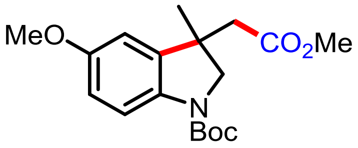

39.9 mg, 60% yield, pale yellow solid;

R_f (PE/EA = 10:1) = 0.2 - 0.3;

Mp 81-84 ℃

^{1}H NMR (400 MHz, CD_3CN) δ 7.64 (s, 1H), 6.77 (d, *J* = 2.8 Hz, 1H), 6.72 (dd, *J* = 8.78 2.8 Hz, 1H), 4.06 (d, *J* = 12.0 Hz, 1H), 3.73 (s, 3H), 3.69 (d, *J* = 11.2 Hz, 1H), 3.57 (s, 3H), 2.72 – 2.56 (m, 2H), 1.52 (s, 9H), 1.35 (s, 3H); **^{13}C NMR** (101 MHz, CD_3CN) δ 171.7, 156.0, 152.6, 140.2, 135.7, 115.3, 112.9, 109.4, 79.6, 60.2, 55.6, 51.4, 44.2, 41.7, 28.0, 26.3; **HRMS (ESI+):** calculated for $C_{18}H_{25}NO_5Na^+$ $[M+Na]^+$ 358.1625, found 358.1624.

Tert-butyl 3-(2-methoxy-2-oxoethyl)-3-methyl-5-(methylthio)indolines -1-carboxylate (4g)

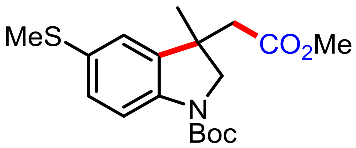

42.8 mg, 58% yield, pale yellow solid;

R_f (PE/EA = 10:1) = 0.2 - 0.3;

Mp 74-77 ℃

^{1}H NMR (400 MHz, CD_3CN) δ δ 7.65 (s, 1H), 7.15 – 7.07 (m, 2H), 4.07 (d, *J* = 11.6 Hz, 1H), 3.71 (d, *J* = 11.6 Hz, 1H), 3.57 (s, 3H), 2.73 – 2.57 (m, 2H), 2.44 (s, 3H), 1.53 (s, 9H), 1.35 (s, 3H); **^{13}C NMR** (101 MHz, CD_3CN) δ 171.9, 152.7, 140.7, 140.0, 131.6, 127.8, 122.8, 115.4, 81.1, 60.4, 51.6, 44.5, 41.6, 28.2, 26.6, 16.8; **HRMS (ESI+):** calculated for $C_{18}H_{25}NO_4SNa^+$ $[M+Na]^+$ 374.1397, found 374.1389.

Tert-butyl 4,6-dimethoxy-3-(2-methoxy-2-oxoethyl)-3-methylindoline -1-carboxylate (4h)

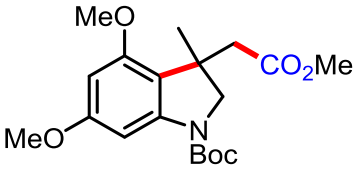

42.2 mg, 58% yield, pale yellow oil;

R_f (PE/EA = 10:1) = 0.3 - 0.4;

^{1}H NMR (400 MHz, CD_3CN) **^{1}H NMR** (400 MHz, CD_3CN) δ 7.02 (s, 1H), 6.15 (d, *J* = 2.4 Hz, 1H), 4.07 (d, *J* = 11.2 Hz, 1H), 3.77 (s, 3H), 3.75 (s, 3H), 3.67 (d, *J* = 11.2 Hz, 1H), 3.54 (s, 3H), 2.88 – 2.65 (m, 2H), 1.52 (s, 9H), 1.37 (s, 3H); **^{13}C NMR** (101 MHz, CD_3CN) δ 172.2, 161.7, 157.3, 152.5, 144.3, 116.1, 115.0, 93.2, 80.8, 60.9, 55.4, 51.2, 42.7, 41.4, 28.0, 25.2; **HRMS (ESI+):** calculated for $C_{19}H_{27}NO_6Na^+$ $[M+Na]^+$ 388.1731, found 388.1726.

Tert-butyl 4,5,6-trimethoxy-3-(2-methoxy-2-oxoethyl)-3-methylindoline -1-carboxylate (4i)

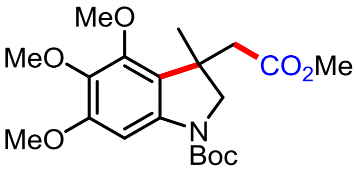

41.1 mg, 52% yield, pale yellow oil;

R_f (PE/EA = 5:1) = 0.4 - 0.5;

^{1}H NMR (400 MHz, CD_3CN) δ 7.29 (s, 1H), 4.06 (d, *J* = 11.2 Hz, 1H), 3.90 (s, 3H), 3.79 (s, 3H), 3.72 (s, 3H),

3.65 (d, J = 11.6 Hz, 1H), 3.53 (s, 3H), 2.86 – 2.65 (m, 2H), 1.53 (s, 9H), 1.39 (s, 3H); **^{13}C NMR** (101 MHz, CD_3CN) δ 172.2, 154.3, 152.6, 150.7, 137.6, 121.1, 105.0, 95.7, 81.0, 61.0, 60.71, 60.66, 56.1, 51.4, 43.6, 41.9, 28.2, 26.4; **HRMS (ESI+):** calculated for $C_{20}H_{29}NO_7Na^+$ [M+Na]$^+$ 418.1836, found 418.1835.

Methyl 2-(6-methoxychroman-4-yl)acetate (6a)

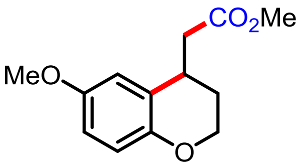

16.1 mg, 34% yield, colorless oil;

R_f (PE/EA = 20:1) = 0.2 - 0.3;

^{1}H NMR (600 MHz, $CDCl_3$) δ 6.74 (d, J = 8.9 Hz, 1H), 6.70 (dd, J = 8.9, 2.9 Hz, 1H), 6.65 (d, J = 2.9 Hz, 1H), 4.17 – 4.10 (m, 2H), 3.74 (s, 3H), 3.73 (s, 3H), 3.37 – 3.20 (m, 1H), 2.80 (dd, J = 15.7, 4.7 Hz, 1H), 2.54 (dd, J = 15.7, 10.1 Hz, 1H), 2.18 – 2.10 (m, 1H), 1.85 – 1.78 (m, 1H); **^{13}C NMR** (150 MHz, $CDCl_3$) δ 172.6, 153.4, 148.6, 125.0, 117.7, 114.1, 113.3, 63.1, 55.7, 51.8, 41.2, 30.8, 27.5; **HRMS (ESI+):** calculated for $C_{13}H_{16}O_4Na^+$ [M+Na]$^+$ 259.0941, found 259.0940.

Methyl 2-(6-(tert-butyl)chroman-4-yl)acetate (6b)

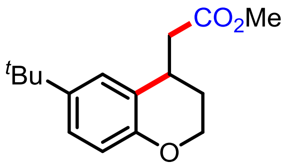

11.0 mg, 21% yield, colorless oil;

R_f (PE/EA = 20:1) = 0.3 - 0.4;

^{1}H NMR (600 MHz, $CDCl_3$) δ 7.17 (dd, J = 8.6, 2.4 Hz, 1H), 7.13 (dd, J = 2.4, 0.8 Hz, 1H), 6.77 (d, J = 8.6 Hz, 1H), 4.23 – 4.19 (m, 1H), 4.18 – 4.13 (m, 1H), 3.76 (s, 3H), 3.41 – 3.35 (m, 1H), 2.83 (dd, J = 15.6, 4.8 Hz, 1H), 2.59 (dd, J = 15.5, 10.1 Hz, 1H), 2.26 – 2.11 (m, 1H), 1.88 – 1.82 (m, 1H), 1.30 (s, 9H); **^{13}C NMR** (150 MHz, $CDCl_3$) δ 172.7, 152.2, 143.1, 125.4, 125.0, 123.6, 116.5, 63.0, 51.7, 41.4, 34.1, 31.5, 30.7, 27.5; **HRMS (ESI+):** calculated for $C_{16}H_{22}O_3Na^+$ [M+Na]$^+$ 285.1461, found 285.1458.

Methyl 2-(6-methoxythiochroman-4-yl)acetate (6c)

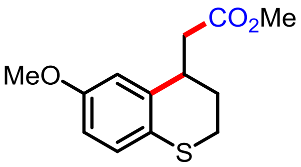

32.8 mg, 65% yield, colorless oil;

R_f (PE/EA = 20:1) = 0.4 - 0.5;

^{1}H NMR (400 MHz, $CDCl_3$) δ 7.06 – 6.98 (m, 1H), 6.75 – 6.66 (m, 2H), 3.75 (s, 3H), 3.70 (s, 3H), 3.43 – 3.36 (m, 1H),

3.14 – 3.05 (m, 1H), 2.92 (dt, J = 12.4, 4.8 Hz, 1H), 2.72 – 2.56 (m, 2H), 2.18 – 1.99 (m, 2H); **^{13}C NMR** (101 MHz, $CDCl_3$) δ 172.6, 156.8, 137.3, 127.8, 123.3, 114.8, 113.5, 55.4, 51.8, 39.2, 34.9, 26.7, 22.9; **HRMS (ESI+):** calculated for $C_{13}H_{16}O_3SNa^+$ $[M+Na]^+$ 275.0712, found 275.0710.

Methyl 2-(6-(tert-butyl)thiochroman-4-yl)acetate (6d)

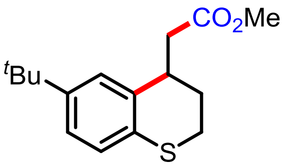

29.0 mg, 52% yield, colorless oil;

R_f (PE/EA = 20:1) = 0.4 - 0.5;

^{1}H NMR (600 MHz, $CDCl_3$) δ 7.14 – 7.10 (m, 2H), 7.03 (d, J = 8.0 Hz, 1H), 3.70 (s, 3H), 3.47 – 3.39 (m, 1H), 3.17 – 3.09 (m, 1H), 2.92 (dt, J = 12.5, 4.5 Hz, 1H), 2.69 – 2.57 (m, 2H), 2.24 – 2.13 (m, 1H), 2.10 – 2.01 (m, 1H), 1.27 (s, 9H); **^{13}C NMR** (150 MHz, $CDCl_3$) δ 172.7, 158.3, 135.2, 128.9, 126.5, 126.3, 124.3, 51.7, 39.7, 34.9, 34.3, 31.3, 26.6, 22.7; **HRMS (ESI+):** calculated for $C_{16}H_{22}O_2SNa^+$ $[M+Na]^+$ 301.1233, found 301.1235.

2.4 Gram scale of 1a

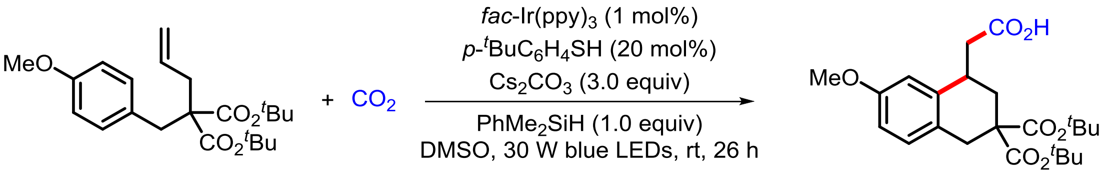

1a, 4 mmol **2a**, 1.40 g, 84%

Procedure: To an oven-dried Schlenk tube (500 mL) equipped with a magnetic stir bar was added **1a** (4 mmol, 1.50 g), *fac*-Ir(ppy)$_3$ (0.04 mmol, 26.2 mg, 1 mol%). Then moved the tube into the glovebox and added the Cs_2CO_3 (12 mmol, 3.91 g, 3.0 equiv). The tube was sealed and removed from the glovebox, then evacuated and back-filled with CO_2 atmosphere three times, anhydrous DMSO (40 mL) was added under CO_2 atmosphere followed by $PhMe_2SiH$ (4 mmol, 546 mg, 630 μL, 1.0 equiv), 4-*tert*-butylthiophenol (0.8 mmol, 134 mg, 140 μL, 20 mol%) and the tube was sealed at atmospheric pressure of CO_2 (1 atm). The reaction was stirred and irradiated with a 30 W blue LEDs lamp (1 cm away, with a cooling fan to keep the reaction temperature at 25-30 ℃ and keep the reaction region located in the center of LEDs lamp) for 26 hours. The reaction mixture was diluted with 60 mL EA and quenched by 60 mL 2 N HCl. The mixture was extracted with EA ten times and the combined organic phases were

concentrated *in vacuo*. The residue was purified by a silica gel flash column chromatography to give the pure desired product **2a** (1.40 g, 84%).

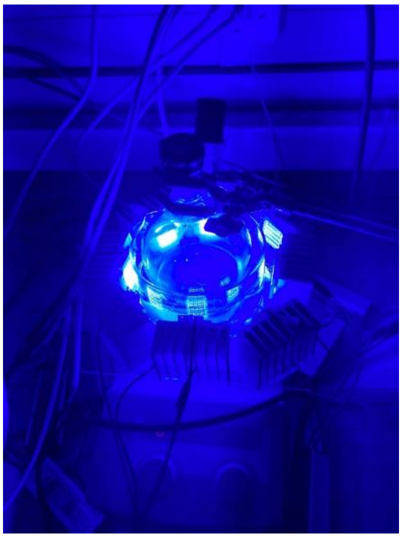

Supplementary Figure 2. Blue LEDs photoreactor for gram scale arylcarboxylation reaction.

2.5 Product derivations

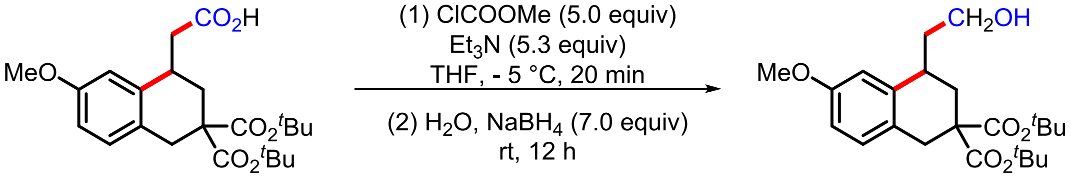

An oven-dried two-necked flask (25 mL) was equipped with a magnetic stir bar and the carboxylic acid **2a** (0.2 mmol, 84 mg, 1.0 equiv). The flask was evacuated and backfilled with N_2 three times. After the addition of dry THF (6 mL), Et_3N (1.1 mmol, 150 μL, 5.3 equiv) was added at -5 ℃, followed by the dropwise addition of methyl chloroformate (1.0 mmol, 65 μL, 5.0 equiv) and stirred at -5 ℃ for 20 min. The heterogeneous mixture was filtered and washed by THF twice, water (0.25 mL) and $NaBH_4$ (1.4 mmol, 53 mg, 7.0 equiv) were added sequentially to the filtrate and the mixture was stirred at room temperature for another 12 hours. After the reaction was finished, the reaction mixture was concentrated *in vacuo* and the residue was purified

by a silica gel flash column chromatography to give the pure desired product **7** (74.6 mg, 92%) as a colorless oil.

Di-tert-butyl 4-(2-hydroxyethyl)-6-methoxy-3,4-dihydronaphthalene-2,2(1H)-dicarboxylate (7)

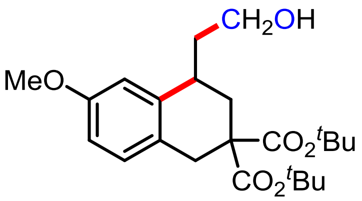

74.6 mg, 92% yield, colorless oil;

R_f (PE/EA = 2:1) = 0.4 - 0.5;

^{1}H NMR (400 MHz, $CDCl_3$) δ 7.02 (d, *J* = 8.3 Hz, 1H), 6.78 – 6.75 (m, 1H), 6.69 (ddd, *J* = 8.4, 2.7, 0.7 Hz, 1H), 3.81 (dd, *J* = 7.6, 6.0 Hz, 2H), 3.77 (s, 3H), 3.14 (dd, *J* = 15.5, 2.0 Hz, 1H), 3.09 – 2.95 (m, 2H), 2.55 (ddd, *J* = 13.5, 6.5, 2.0 Hz, 1H), 2.25 – 2.17 (m, 1H), 1.83 – 1.69 (m, 2H), 1.46 (s, 9H), 1.32 (s, 9H); **^{13}C NMR** (101 MHz, $CDCl_3$) δ 171.4, 170.1, 158.2, 140.1, 129.7, 126.7, 112.0, 111.8, 81.5, 81.0, 60.6, 55.2, 55.1, 38.8, 34.7, 34.3, 32.6, 27.9, 27.7; **HRMS (ESI+)**: calculated for $C_{23}H_{34}O_6Na^+$ $[M+Na]^+$: 429.2248, found 429.2248.

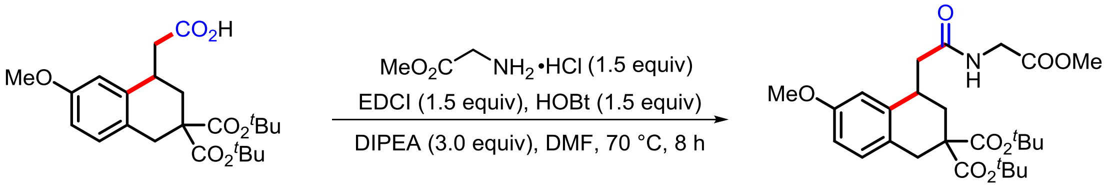

An oven-dried Schlenk tube (10 mL) was equipped with a magnetic stir bar, the carboxylic acid **2a** (0.1 mmol, 42 mg, 1.0 equiv), glycine methyl ester hydrochloride (0.15 mmol, 19 mg, 1.5 equiv), EDCI (0.15 mmol, 29 mg, 1.5 equiv) and HOBt (0.15 mmol, 20 mg, 1.5 equiv). The flask was evacuated and backfilled with N_2 three times., DMF (2 mL) and DIPEA (0.3 mmol, 52 μL, 3.0 equiv) were added and the mixture was stirred at 70 ℃ for 8 hours. After the reaction was finished, the reaction mixture was quenched with water and extracted with ethyl acetate three times, dried with anhydrous Na_2SO_4. The combined organic phase was concentrated *in vacuo* and the residue was purified by a silica gel flash column chromatography to give the pure desired product **8** (45.9 mg, 93%) as a white solid.

Di-tert-butyl 6-methoxy-4-(2-((2-methoxy-2-oxoethyl)amino)-2-oxoethyl)-3,4-dihydronaphthalene-2,2(1H)-dicarboxylate (8)

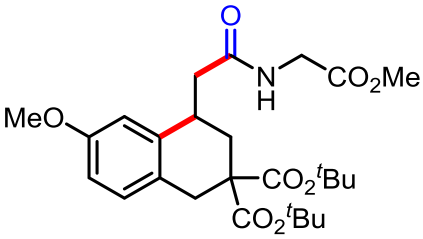

45.9 mg, 93% yield, white solid;

R_f (PE/EA = 1:1) = 0.4 - 0.5;

Mp 126-129 ℃

^{1}H NMR (400 MHz, $CDCl_3$) δ 7.06 (d, *J* = 8.3 Hz, 1H), 6.77 – 6.70 (m, 2H), 6.15 – 6.09 (m, 1H), 4.09 (d, *J* = 5.2 Hz, 2H), 3.78 (d, *J* = 1.2 Hz, 6H), 3.50 – 3.41 (m, 1H), 3.19 (d, *J* = 15.5 Hz, 1H), 3.00 (d, *J* = 15.6 Hz, 1H), 2.83 (dd, *J* = 14.6, 5.0 Hz, 1H), 2.56 (ddd, *J* = 13.6, 6.5, 1.9 Hz, 1H), 2.43 (dd, *J* = 14.6, 8.8 Hz, 1H), 1.87 (dd, *J* = 13.6, 9.7 Hz, 1H), 1.47 (s, 9H), 1.37 (s, 9H); **^{13}C NMR** (101 MHz, $CDCl_3$) δ 171.6, 171.3, 170.2, 169.9, 158.2, 138.8, 129. 8, 126.7, 112.3, 111.6, 81.6, 81.2, 55.2, 54.9, 52.3, 43.0, 41.3, 34.6, 34.1, 33.1, 27.8, 27.7; **HRMS (ESI+)**: calculated for $C_{26}H_{38}NO_8^+$ $[M+H]^+$: 492.2592, found 492.2597.

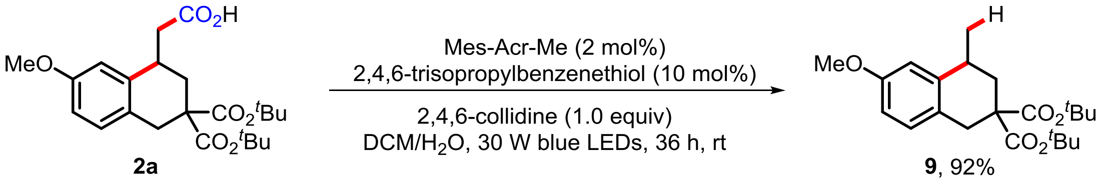

An oven-dried Schlenk tube (10 mL) was equipped with a magnetic stir bar, the carboxylic acid **2a** (0.2 mmol, 84 mg, 1.0 equiv), 9-Mesityl-10-methyl acridinium perchlorate (Mes-Acr-Me) (0.004 mmol, 1.6 mg, 2 mol%). The flask was evacuated and backfilled with N_2 three times. DCM (1.6 mL), water (0.4 mL), 2,4,6-triisopropylbenzenethiol (0.02 mmol, 4.8 mg, 10 mol%) and 2,4,6-collidine (0.2 mmol, 24 mg, 1.0 equiv) were added successively with a syringe under N_2. The tube was then sealed and stirred under the irradiation of 30 W blue LEDs at room temperature for 36 hours. After the reaction was finished, the reaction mixture was extracted by ethyl acetate three times, dried by anhydrous Na_2SO_4. The combined organic phase was concentrated *in vacuo* and the residue was purified by a silica gel flash chromatography to give the pure desired product **9** (69.3 mg, 92%) as a colorless oil.

Di-tert-butyl 6-methoxy-4-methyl-3,4-dihydronaphthalene-2,2(1H)-dicarboxylate (9)

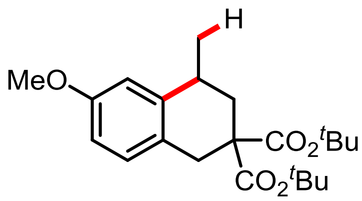

69.3 mg, 92% yield, colorless oil;

R_f (PE/EA = 20:1) = 0.4 - 0.5;

^{1}H NMR (400 MHz, $CDCl_3$) δ 7.04 (d, *J* = 8.4 Hz, 1H), 6.79 (dd, *J* = 2.7, 1.0 Hz, 1H), 6.74 – 6.68 (m, 1H), 3.79 (s, 3H), 3.21 (dd, *J* = 15.7, 2.0 Hz, 1H), 2.98 (dt, *J* = 15.5, 1.1 Hz, 1H), 2.92-2.84 (m, 1H), 2.48 (ddd, *J* = 13.5, 6.0, 2.1 Hz, 1H), 1.73 (dd, *J* = 13.5, 11.2 Hz, 1H), 1.48 (s, 9H), 1.34 (s, 9H), 1.31 (s, 3H); **^{13}C NMR** (101 MHz, $CDCl_3$) δ 171.4, 170.2, 158.0, 141.4, 129.3, 126.3, 111.8, 111.5, 81.3, 80.9, 55.1, 55.0, 37.2, 34.7, 30.3, 27.8, 27.7, 21.4. **HRMS (ESI+)**: calculated for $C_{22}H_{32}O_5Na^+$ $[M+Na]^+$: 399.2142, found 399.2137.

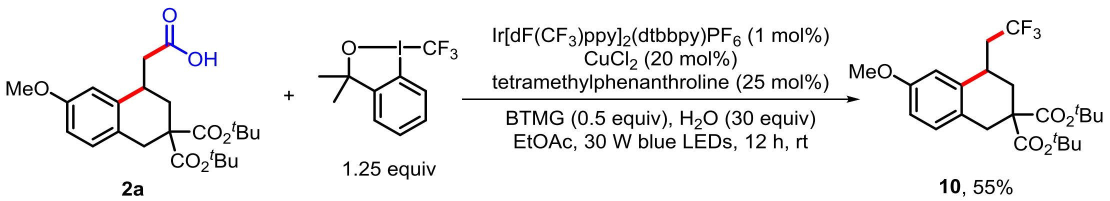

An oven-dried Schlenk tube (25 mL) was equipped with a magnetic stir bar, the carboxylic acid **2a** (0.2 mmol, 84 mg, 1.0 equiv), Ir[dF(CF_3)ppy]$_2$(dtbbpy)PF_6 (0.002 mmol, 2.2 mg, 1 mol%), $CuCl_2$ (0.04 mmol, 5.4 mg, 20 mol%), tetramethylphenanthroline (0.05 mmol, 11.8 mg, 25 mol%) and Togni's reagent I (0.25 mmol, 82.6 mg, 1.25 equiv). The flask was evacuated and backfilled with N_2 three times. EtOAc was added (8 mL) followed by addition of BTMG (0.1 mmol, 17.2 mg, 0.5 equiv) and water (6 mmol, 108 μL, 30 equiv). The tube was then sealed and stirred under the irradiation of 30 W blue LEDs at room temperature for 12 hours. After the reaction was finished, the reaction mixture was extracted by ethyl acetate three times and dried with anhydrous Na_2SO_4. The combined organic phase was concentrated *in vacuo* and the residue was purified by a silica gel flash chromatography to give the pure desired product **10** (48.9 mg, 55%) as a colorless oil.

Di-tert-butyl 6-methoxy-4-methyl-3,4-dihydronaphthalene-2,2(1H)-dicarboxylate (10)

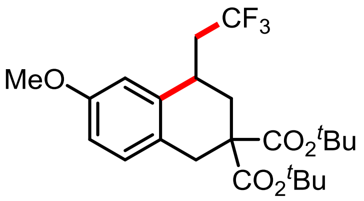

48.9 mg, 55% yield, colorless oil;

R_f (PE/EA = 50:1) = 0.4 - 0.5;

^{1}H NMR (400 MHz, $CDCl_3$) δ 7.02 (d, J = 8.4 Hz, 1H), 6.71 (dd, J = 8.4, 2.5 Hz, 1H), 6.66 (d, J = 2.5 Hz, 1H), 3.75 (s, 3H), 3.28 – 3.13 (m, 2H), 2.95 (d, J = 15.7 Hz, 1H), 2.79 – 2.64 (m, 2H), 2.34 – 2.16 (m, 1H), 1.84 (dd, J = 13.7, 10.1 Hz, 1H), 1.44 (s, 9H), 1.31 (s, 9H); **^{13}C NMR** (101 MHz, $CDCl_3$) δ 171.0, 169.6, 158.3, 137.6, 130.1, 127.0 (q, J = 28.3 Hz), 126.7, 112.4, 112.2, 81.8, 81.3, 55.3, 54.8, 40.8 (q, J = 28.3 Hz), 34.8, 34.5, 30.8 (q, J = 2.2 Hz), 27.8, 27.7; **^{19}F NMR** (376 MHz, $CDCl_3$) δ -63.57. **HRMS (ESI+)**: calculated for $C_{23}H_{31}F_3O_5Na^+$ $[M+Na]^+$: 467.2016, found 467.2014.

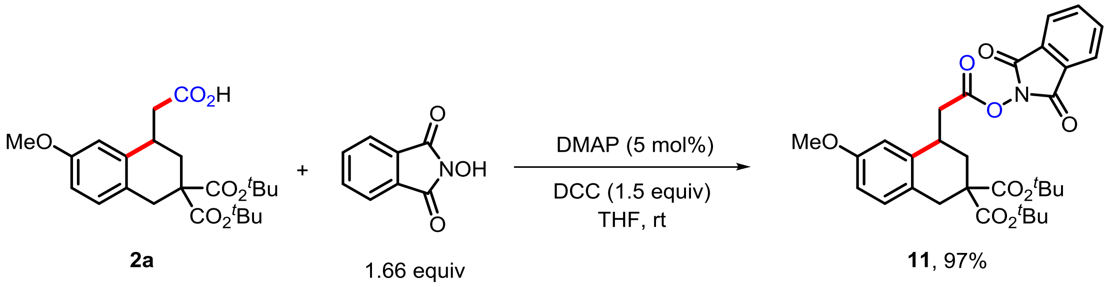

An oven-dried two-necked flask (25 mL) was equipped with a magnetic stir bar, carboxylic acid **2a** (0.2 mmol, 84 mg, 1.0 equiv) and was evacuated and backfilled with N_2 three times. After sequential addition of dry THF (1.2 mL), *N*-hydroxyphthalimide (0.33 mmol, 54 mg, 1.66 equiv), DMAP (0.01 mmol, 1.2 mg, 5.0 mol%), and DCC (0.3 mmol, 62 mg, 1.50 equiv), the reaction mixture was stirred at room temperature for 10 hours. After the reaction was completed, the heterogeneous mixture was filtered and the filtrate was concentrated *in vacuo*. The crude product was purified by a silica gel flash column chromatography to give the pure desired product **10** (117.5 mg, 97%) as a white solid.

Di-tert-butyl 6-methoxy-4-methyl-3,4-dihydronaphthalene-2,2(1H)-dicarboxylate (11)

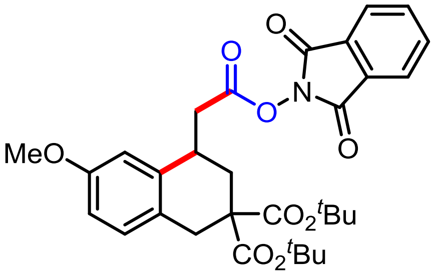

117.5 mg, 97% yield, white solid; R_f (PE/EA = 5:1) = 0.3 - 0.4; **^{1}H NMR** (400 MHz, $CDCl_3$) δ 7.91 – 7.83 (m, 2H), 7.80 – 7.74 (m, 2H), 7.03 (d, J = 8.2 Hz, 1H), 6.76 – 6.69 (m, 2H), 3.79 (s, 3H), 3.51 (s, 1H), 3.25 – 3.14 (m, 2H), 3.00 (d, J = 15.6 Hz, 1H), 2.87 (dd, J = 16.4, 8.6 Hz, 1H), 2.72 (dd, J = 13.6, 6.4 Hz, 1H), 1.97 – 1.89 (m, 1H), 1.45 (s, 9H), 1.32 (s, 9H); **^{13}C NMR** (101 MHz, $CDCl_3$) δ 170.8, 169.7, 168.4, 161.8, 158.3, 137.4, 134.7, 130.0, 128.9, 126.6, 124.0, 113.0, 111.3, 81.7, 81.2, 55.3, 54.9, 37.8, 34.5, 34.4, 32.8, 27.8, 27.7; **HRMS (ESI+)**: calculated for $C_{31}H_{35}NO_9Na^+$ $[M+Na]^+$: 588.2204, found 588.2204.

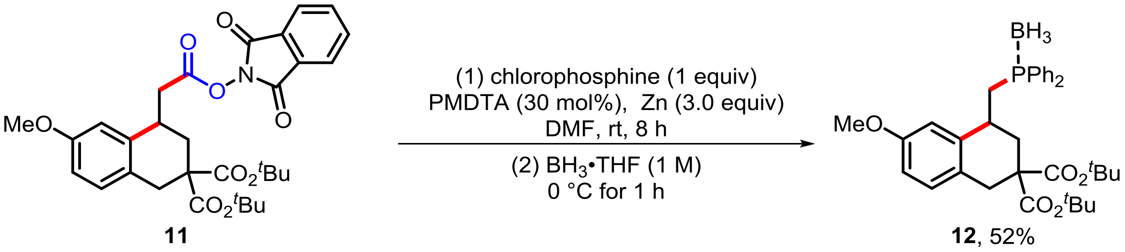

An oven-dried Schlenk tube (10 mL) was equipped with a magnetic stir bar, NHP ester **11** (0.15 mmol, 84.8 mg, 1.5 equiv) and was evacuated and backfilled with N_2 three times. Then chlorophosphine (0.1 mmol, 18 μL, 1.0 equiv), PMDTA (0.03 mmol, 6.3 μL, 30 mol%), and DMF (0.6 mL) was added sequentially. After stirring for 2 min, the activated zinc powder (0.3 mmol, 16.9 mg, 3.0 equiv) was added. The reaction was stirred at room temperature for 6 hours. Borane-tetrahydrofuran complex (0.6 mmol, 0.6 mL, 3.0 equiv, 1 M solution in THF) was added to the reaction mixture under N_2 at 0 ℃ and stirred for 1 hour at 0 ℃. The mixture was quenched with MeOH (0.5 mL) and extracted with ethyl acetate three times, dried with anhydrous Na_2SO_4. The combined organic phase was concentrated *in vacuo* and the residue was purified by a silica gel flash column chromatography to give the pure desired product **12** (35.7 mg, 52%) as a white solid.

Di-tert-butyl 4-(((l4-boranyl) diphenyl-l5-phosphanyl)methyl)-6-methoxy-3,4-dihydronaphthalene-2,2(1H)-dicarboxylate (12)

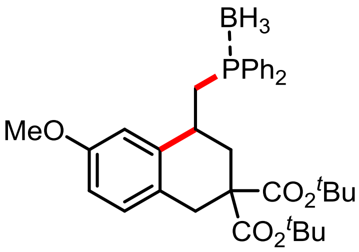

35.7 mg, 52% yield, white solid;

R_f (PE/EA = 5:1) = 0.4 - 0.5;

Mp 115-118 ℃

^{1}H NMR (400 MHz, $CDCl_3$) δ 7.84 – 7.70 (m, 4H), 7.54 – 7.42 (m, 6H), 7.00 (d, *J* = 8.4 Hz, 1H), 6.69 (d, *J* = 7.1 Hz, 2H), 3.73 (s, 3H), 3.51 – 3.33 (m, 1H), 3.13 (dd, *J* = 15.6, 2.1 Hz, 1H), 2.93 (d, *J* = 15.5 Hz, 1H), 2.83 (ddd, *J* = 14.8, 11.8, 3.0 Hz, 1H), 2.56 (ddd, *J* = 14.7, 11.6, 9.4 Hz, 1H), 2.47 (ddd, *J* = 13.8, 7.0, 2.1 Hz, 1H), 1.61 (d, *J* = 8.4 Hz, 1H), 1.40 (s, 9H), 1.29 (s, 9H); **^{13}C NMR** (101 MHz, $CDCl_3$) δ 171.0, 169.4, 158.2, 139.9 (d, *J* = 11.3 Hz), 132.5 (d, *J* = 9.2 Hz), 132.0 (d, *J* = 8.9 Hz), 131.3 (d, *J* = 2.2 Hz), 131.1 (d, *J* = 2.4 Hz), 129.7, 128.9 (d, *J* = 4.8 Hz), 128.8 (d, *J* = 4.9 Hz), 112.4 (d, *J* = 15.3 Hz), 81.4, 81.2, 55.2 (d, *J* = 15.6 Hz), 36.4 (d, *J* = 2.4 Hz), 35.0, 34.8, 34.6, 31.9 (d, *J* = 2.0 Hz), 27.8, 27.6; **^{31}P NMR** (162 MHz, $CDCl_3$) δ 14.87. **HRMS (ESI+)**: calculated for $C_{34}H_{44}BO_5PNa^+$ $[M+Na]^+$: 597.2912, found 597.2914.

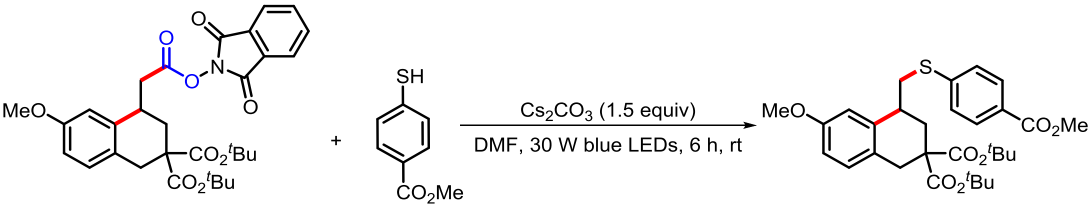

11 **13**, 56%

An oven-dried Schlenk tube (10 mL) was equipped with a magnetic stir bar, NHP ester **11** (0.1 mmol, 1.0 equiv), methyl 4-mercaptobenzoate (0.12 mmol, 1.2 equiv) and Cs_2CO_3 (0.15 mmol, 1.5 equiv). The flask was evacuated and backfilled with N_2 three times, followed by the addition of DMF (1 mL). The mixture was irradiated with 30 W blue LEDs for 6 hours. After the reaction was completed, the reaction mixture was extracted with ethyl acetate three times, dried with anhydrous Na_2SO_4. The combined organic phase was concentrated *in vacuo* and the residue was purified by a silica gel flash column chromatography to give the pure desired product **13** (30.4 mg, 56%) as a colorless oil.

Di-tert-butyl 6-methoxy-4-(((4-(methoxycarbonyl)phenyl) thio)methyl)-3,4-dihydronaphthalene-2,2(1H)-dicarboxylate (13)

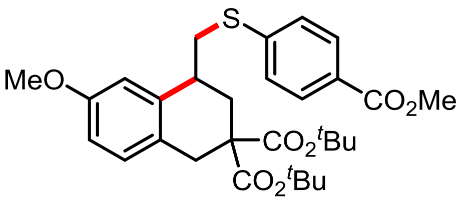

30.4 mg, 56% yield, colorless oil; R_f (PE/EA = 5:1) = 0.4 - 0.5; **^{1}H NMR** (400 MHz, $CDCl_3$) δ 7.92 (dd, *J* = 8.5, 1.6 Hz, 2H), 7.35 (dd, *J* = 8.5, 1.6 Hz, 2H), 7.07 (d, *J* = 8.4 Hz, 1H), 6.82 (d, *J* = 2.6 Hz, 1H), 6.74 (dd, *J* = 8.3, 2.6 Hz, 1H), 3.92 (s, 3H), 3.79 (s, 3H), 3.56 (dd, *J* = 12.5, 3.9 Hz, 1H), 3.27 (dd, *J* = 11.3, 7.6 Hz, 1H), 3.23 – 3.13 (m, 2H), 3.03 (d, *J* = 15.6 Hz, 1H), 2.73 (ddd, *J* = 13.7, 6.7, 2.1 Hz, 1H), 1.96 (ddd, *J* = 13.7, 9.6, 1.5 Hz, 1H), 1.46 (s, 9H), 1.26 (s, 9H).; **^{13}C NMR** (101 MHz, $CDCl_3$) δ 171.1, 169.8, 166.8, 158.2, 144.0, 137.9, 130.0, 129.93, 126.90, 126.9, 112.6, 112.2, 81.7, 81.1, 55.2, 55.1, 52.1, 39.5, 35.4, 34.8, 34.1, 27.9, 27.6; **HRMS (ESI+)**: calculated for $C_{30}H_{38}O_7SNa^+$ $[M+Na]^+$: 565.6762, found 565.6767.

3 Supplementry Notes

3.1 Mechanistic investigations

3.1.1 Trapping experiments

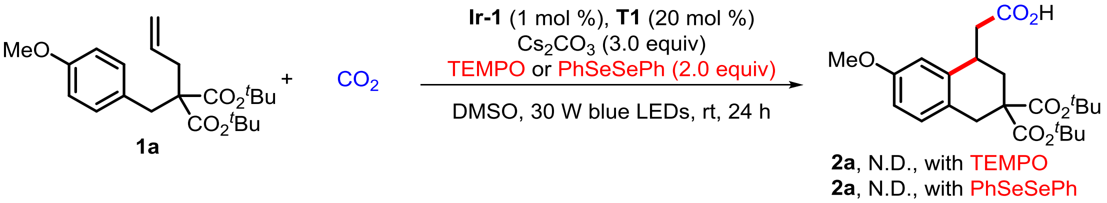

Procedure: To an oven-dried Schlenk tube (25 mL) equipped with a magnetic stir bar was added **1a** (0.2 mmol, 75.2 mg), *fac*-Ir(ppy)$_3$ (1 mol%, 1.3 mg), TEMPO (0.4 mmol, 62.5 mg, 2.0 equiv) or PhSeSePh (0.4 mmol, 125 mg, 2.0 equiv). The tube was moved into the glovebox where was added the Cs_2CO_3 (0.6 mmol, 195.5 mg, 3.0 equiv). The tube was sealed and removed from the glovebox, then evacuated and back-filled with CO_2 atmosphere three times. Anhydrous DMSO (2 mL) was added under CO_2 atmosphere followed by 4-*tert*-butylthiophenol (0.04 mmol, 6.7 mg, 7 μL, 20 mol%), and the tube was sealed at atmospheric pressure of CO_2 (1 atm). The reaction was stirred and irradiated with a 30 W blue LEDs lamp (1 cm away, with a cooling fan to keep the reaction temperature at 25-30 ℃ and keep the reaction region located in the center of LEDs lamp) for 24 hours. The reaction mixture was diluted with 3 mL EA and quenched by 3 mL 2 N HCl. Then the residue was analyzed by ^{1}H NMR with 1,3,5-trimethoxybenzene (0.1 mmol) as the internal standard and the sample was subjected to HRMS analysis.

3.1.2 Reduction of unactivated alkenes 1a

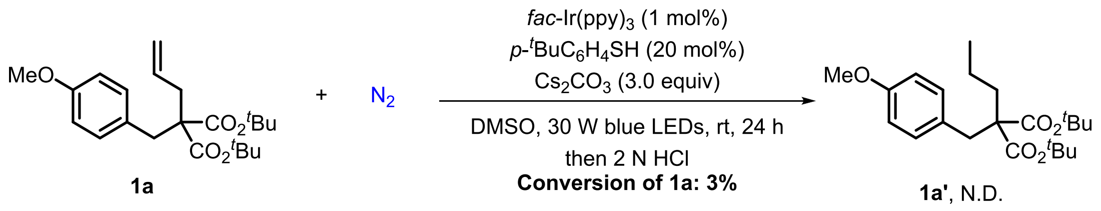

Procedure: To an oven-dried Schlenk tube (25 mL) equipped with a magnetic stir bar was added **1a** (0.2 mmol, 75.2 mg), *fac*-Ir(ppy)$_3$ (1 mol%, 1.3 mg). The tube was moved into the glovebox where was added the Cs_2CO_3 (0.6 mmol, 195.5 mg, 3.0 equiv). The tube was sealed and removed from the glovebox, then evacuated and back-filled with N_2 atmosphere three times. Anhydrous DMSO (2 mL) was added under N_2 atmosphere followed by 4-*tert*-butylthiophenol (0.04 mmol, 6.7 mg, 7 μL, 20 mol%), and the tube was sealed at atmospheric pressure of N_2. The reaction was stirred and irradiated with a 30 W blue LEDs lamp (1 cm away, with a cooling fan to keep the reaction temperature at 25-30 ℃ and keep the reaction region located in the center of LEDs lamp) for 24 hours. The reaction mixture was diluted with 3 mL EA and quenched by 3 mL H_2O. Then the residue was analyzed by ESI-MS and the product **1a'** was not detected by ESI-MS, indicating that **1a** could not be reduced in the reaction.

3.1.3 Detection of the formate and oxalate

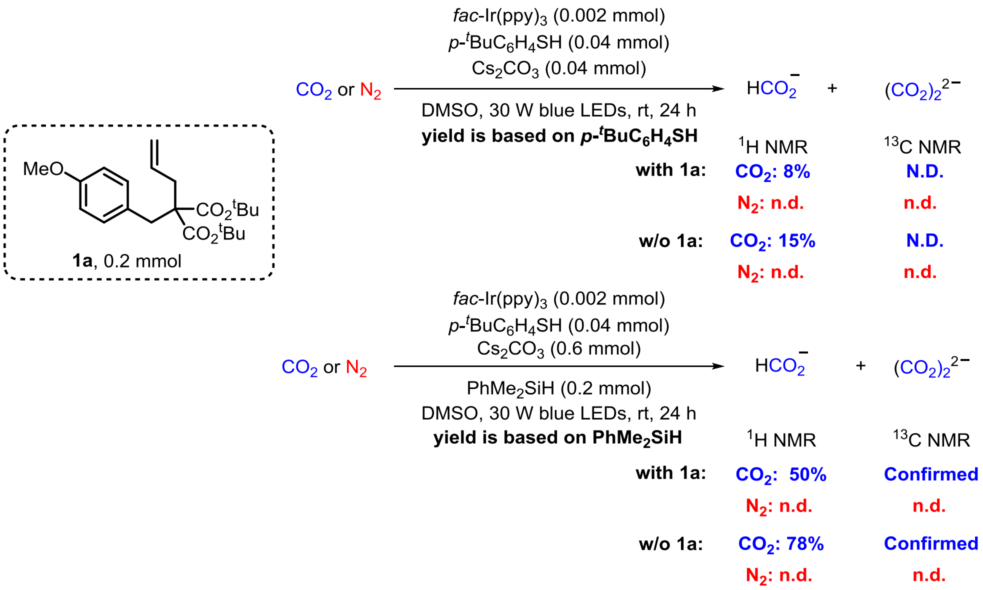

Procedure w/o $PhMe_2SiH$: An oven-dried Schlenk tube (25 mL) equipped with a magnetic stir bar was added **1a** (0.2 mmol, 75.2 mg) or not, *fac*-Ir(ppy)$_3$ (1 mol%, 1.3 mg). The tube was moved into the glovebox where was added the Cs_2CO_3 (0.04 mmol, 13.0 mg). The tube was sealed and removed from the glovebox, then evacuated and back-filled with CO_2 or N_2 atmosphere three times. Anhydrous DMSO (2 mL) was added under CO_2 or N_2 atmosphere followed by 4-*tert*-butylthiophenol (0.04 mmol, 6.7 mg, 7 μL), and the tube was sealed at atmospheric pressure of CO_2 (1 atm). The reaction was stirred and irradiated with a 30 W blue LEDs lamp (1 cm away, with a cooling fan to keep the reaction temperature at 25-30 ℃ and keep the reaction region located in the center of LEDs lamp) for 24 hours. The resulting mixture was concentrated *in vacuo* carefully, and the residue was quenched by 2 mL aqueous sodium *p*-methylbenzene sulfonate (0.05 M), washed with EA and water and transferred to a 25 mL round bottom flask. After the mixture was concentrated *in vacuo*, 1.5 mL D_2O and 5 mL DCM was added to make the mixture dissolve sufficiently. The aqueous phase was analyzed by crude ^{1}H NMR with sodium *p*-methylbenzene sulfonate as the internal standard.

The reaction of substrate 1a under CO_2 atmosphere

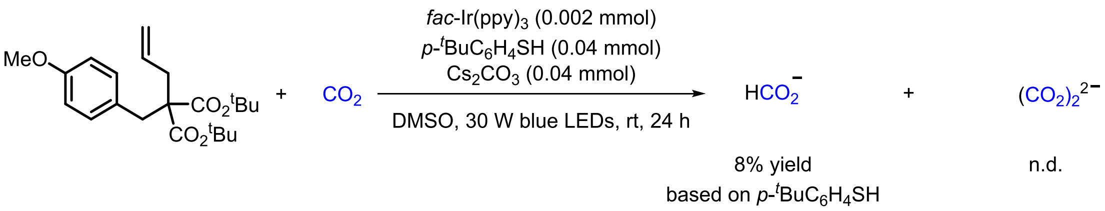

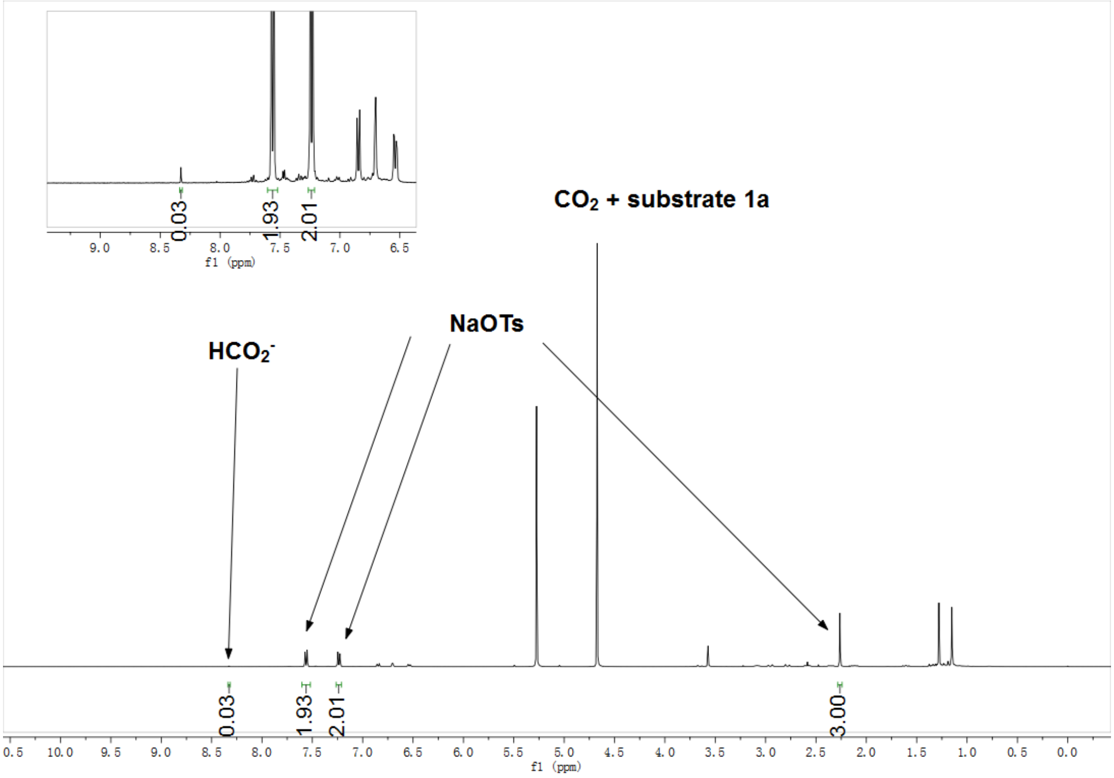

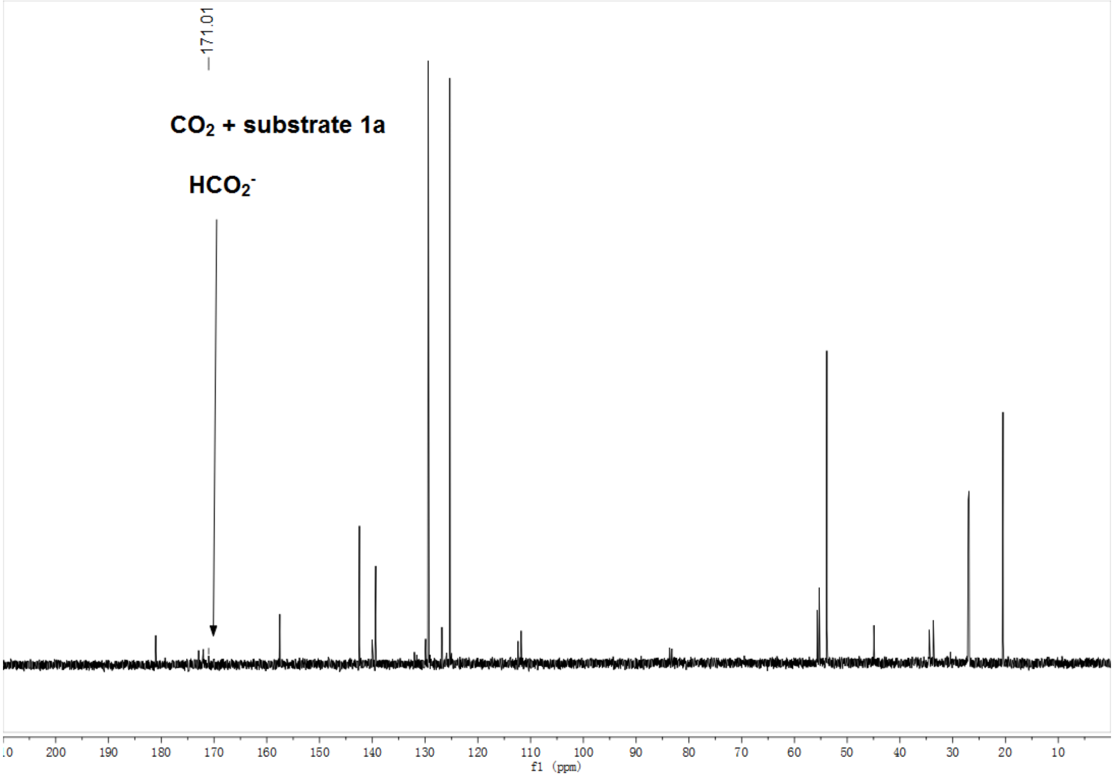

Supplementary Figure 3. Results of the reaction of substrate 1a under CO_2 atmosphere

The reaction w/o substrate 1a under CO_2 atmosphere

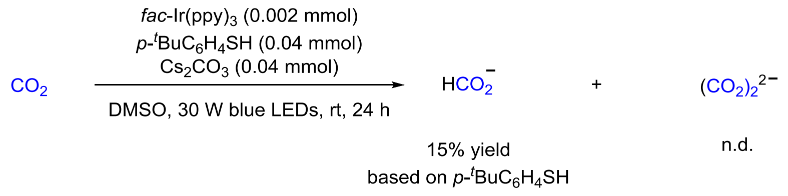

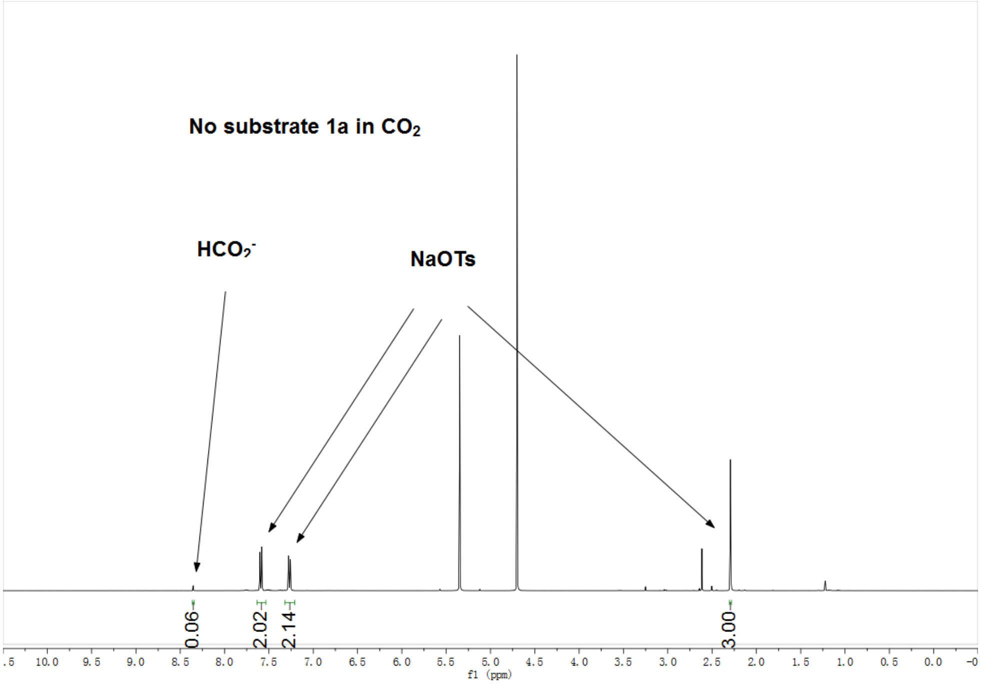

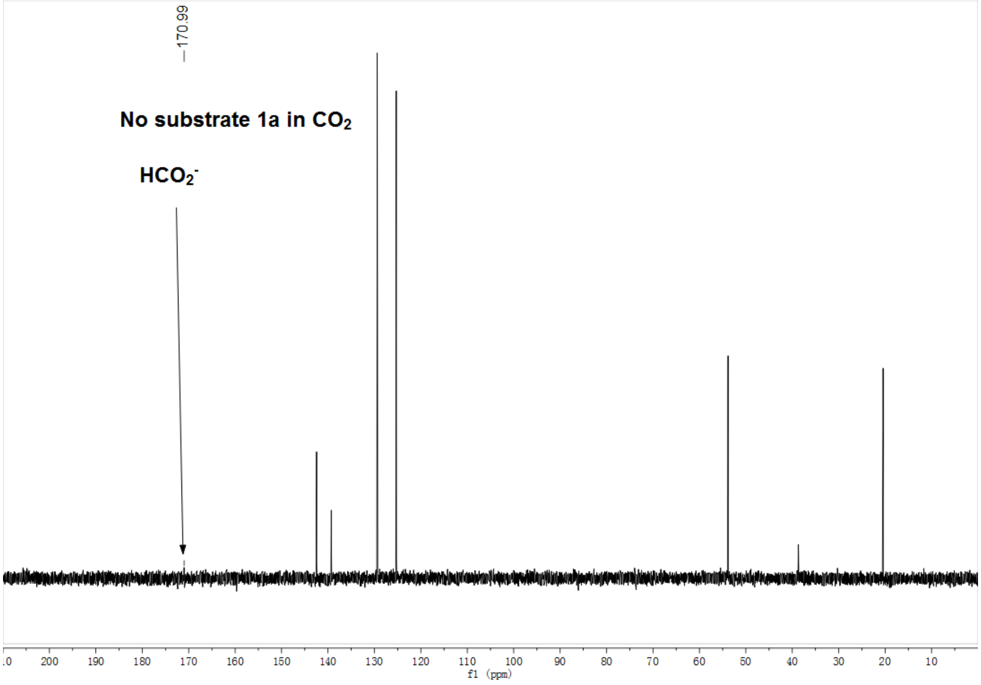

Supplementary Figure 4. Results of the reaction w/o substrate 1a under CO_2 atmosphere

The reaction of substrate 1a under N_2 atmosphere

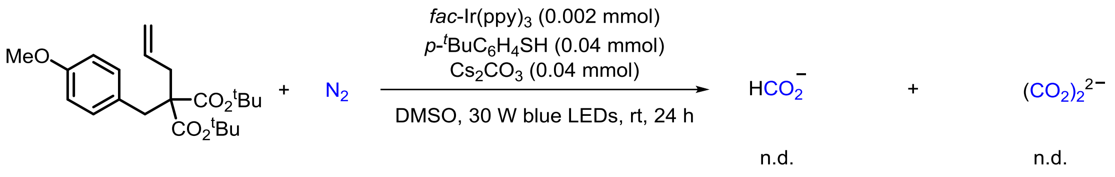

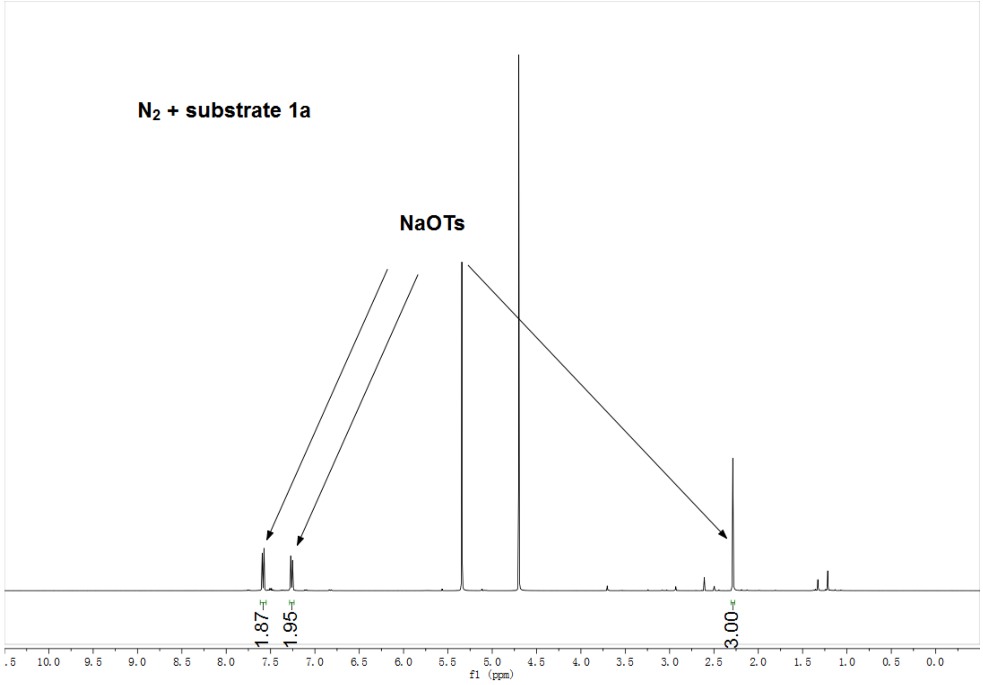

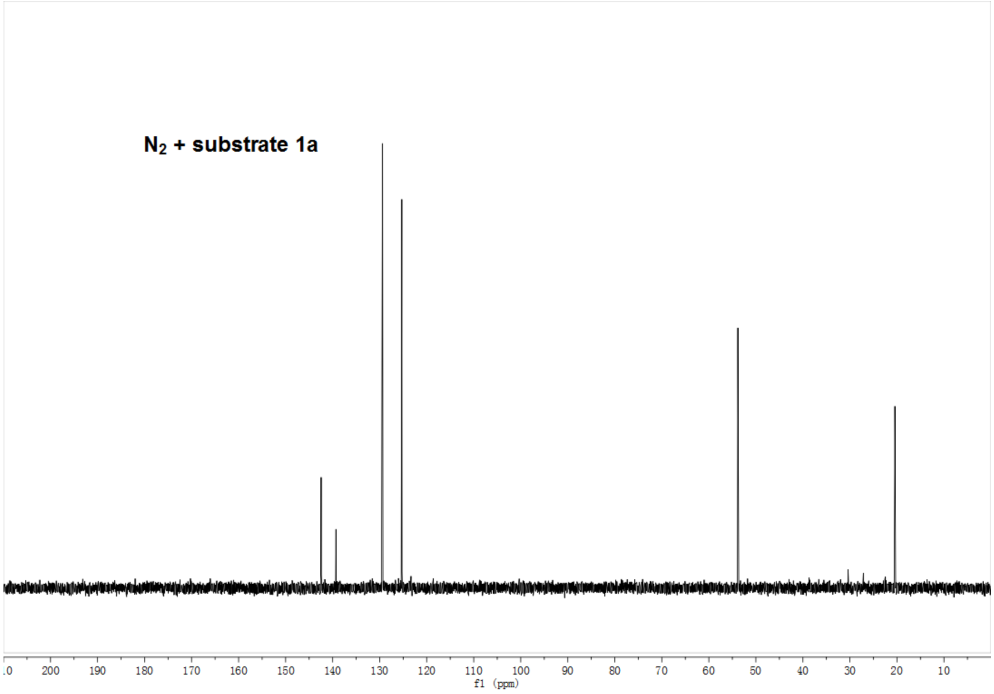

Supplementary Figure 5. Results of the reaction of substrate 1a under N_2 atmosphere

The reaction w/o substrate 1a under N_2 atmosphere

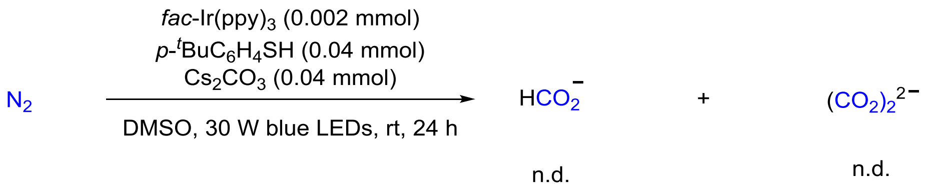

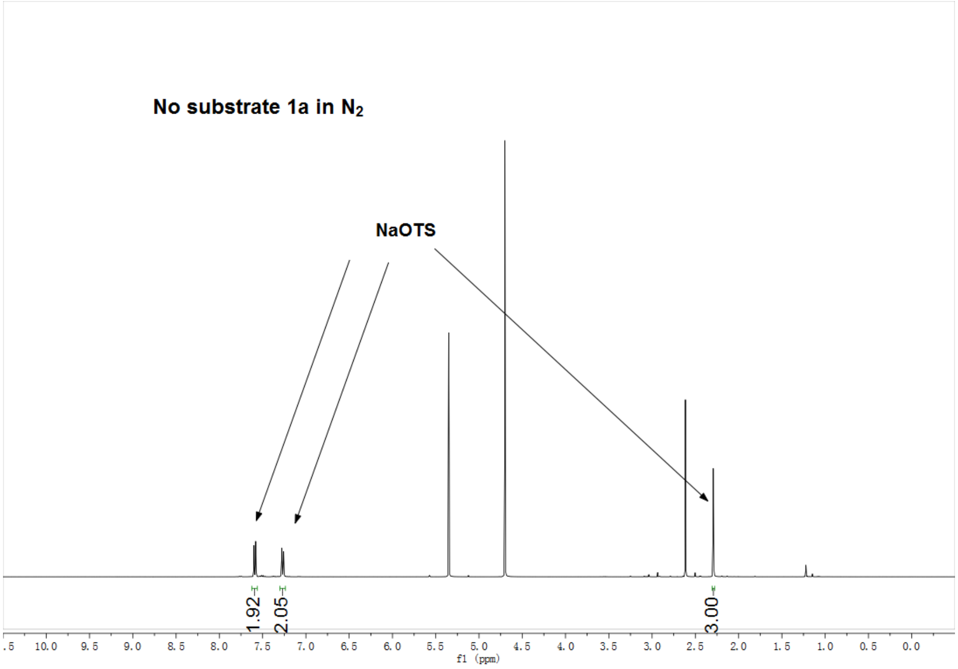

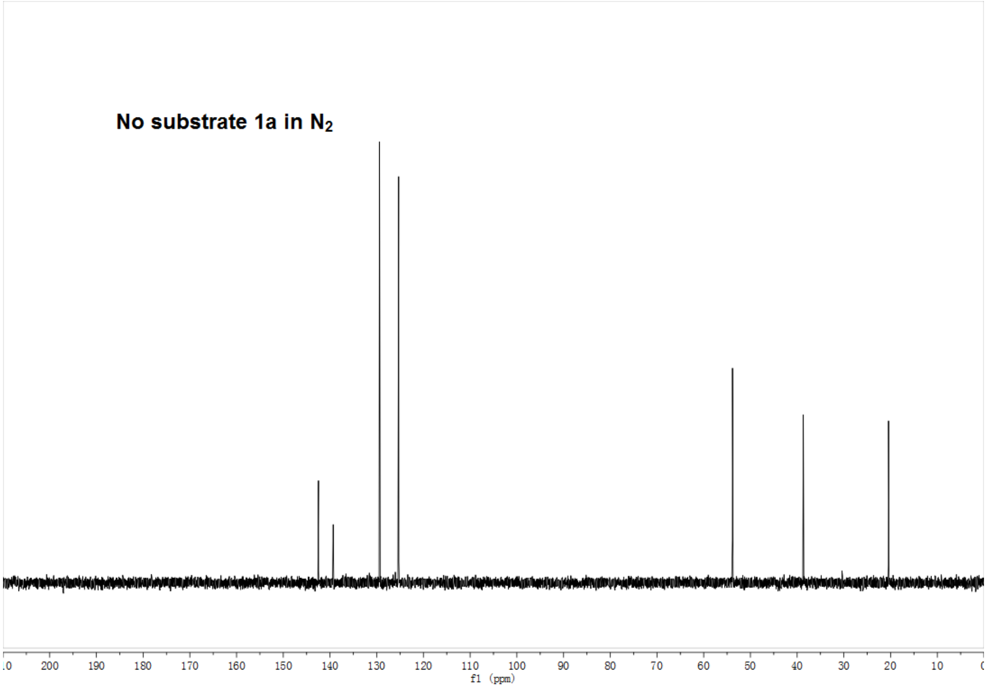

Supplementary Figure 6. Results of the reaction w/o substrate 1a under N_2 atmosphere

Procedure with $PhMe_2SiH$: An oven-dried Schlenk tube (25 mL) equipped with a magnetic stir bar was added **1a** (0.2 mmol, 75.2 mg) or not, *fac*-Ir(ppy)$_3$ (1 mol%, 1.3 mg). The tube was moved into the glovebox where was added the Cs_2CO_3 (0.6 mmol, 195.5 mg, 3.0 equiv). The tube was sealed and removed from the glovebox, then evacuated and back-filled with CO_2 or N_2 atmosphere three times. Anhydrous DMSO (2 mL) was added under CO_2 or N_2 atmosphere followed by $PhMe_2SiH$ (0.2 mmol, 27.3 mg, 31 μL, 1.0 equiv), 4-*tert*-butylthiophenol (0.04 mmol, 6.7 mg, 7 μL, 20 mol%), and the tube was sealed at atmospheric pressure of CO_2 (1 atm). The reaction was stirred and irradiated with a 30 W blue LEDs lamp (1 cm away, with a cooling fan to keep the reaction temperature at 25-30 ℃ and keep the reaction region located in the center of LEDs lamp) for 24 hours. The resulting mixture was concentrated *in vacuo* carefully, and the residue was quenched by 2 mL aqueous sodium *p*-methylbenzene sulfonate (0.05 M), washed with EA and water and transferred to a 25 mL round bottom flask. After the mixture was concentrated *in vacuo*, 1.5 mL D_2O and 5 mL DCM was added to make the mixture dissolve sufficiently. The aqueous phase was analyzed by crude ^1H NMR with sodium *p*-methylbenzene sulfonate as the internal standard.

The reaction of substrate 1a under CO_2 atmosphere with silane

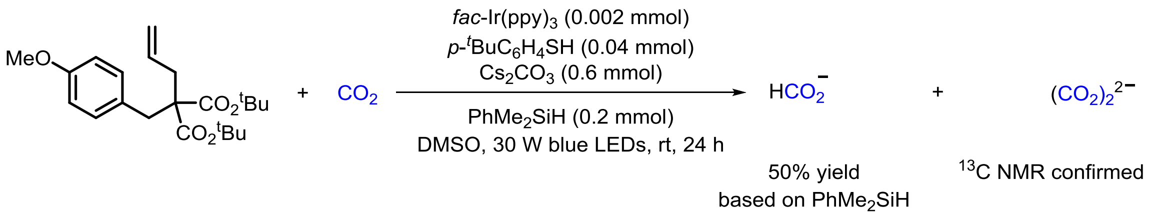

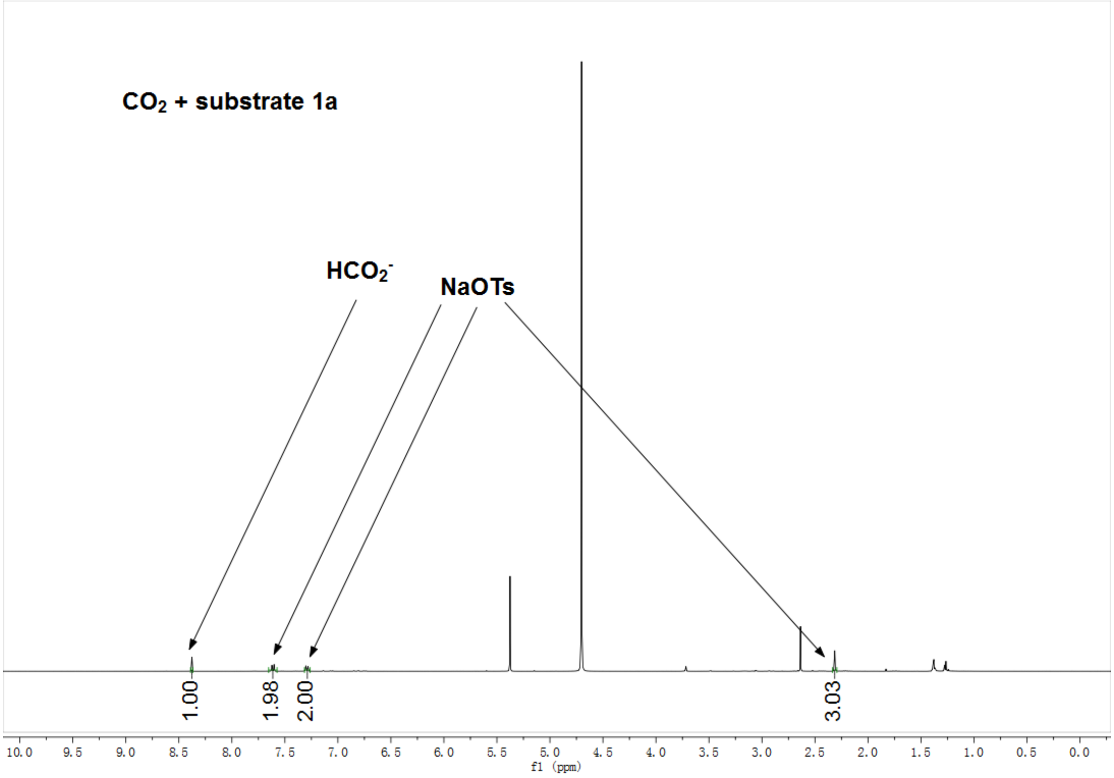

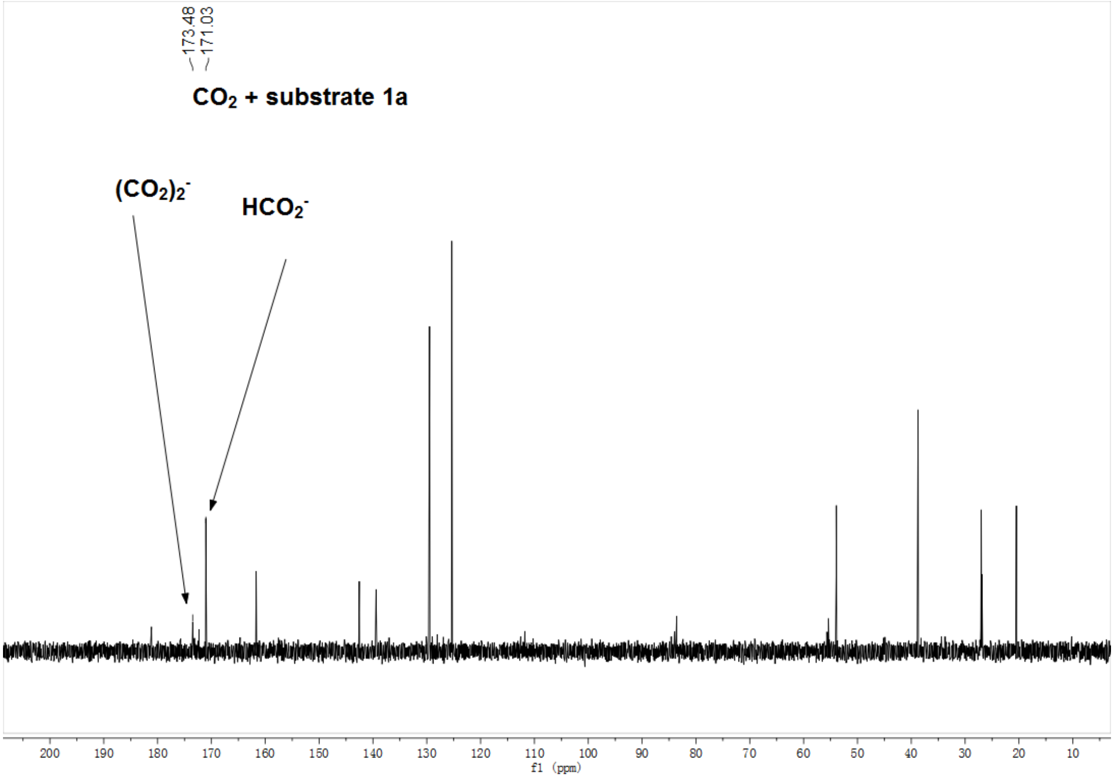

Supplementary Figure 7. Results of reaction in the presence of 1a, PhMe$_2$SiH and CO$_2$.

The reaction w/o substrate 1a under CO_2 atmosphere with silane

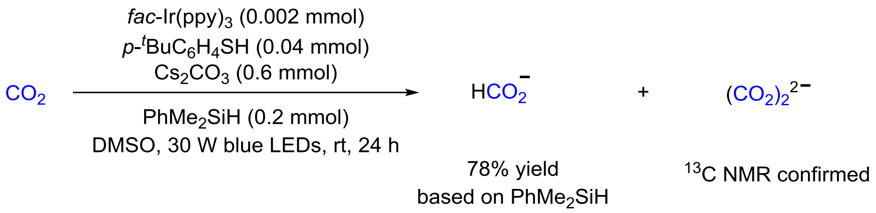

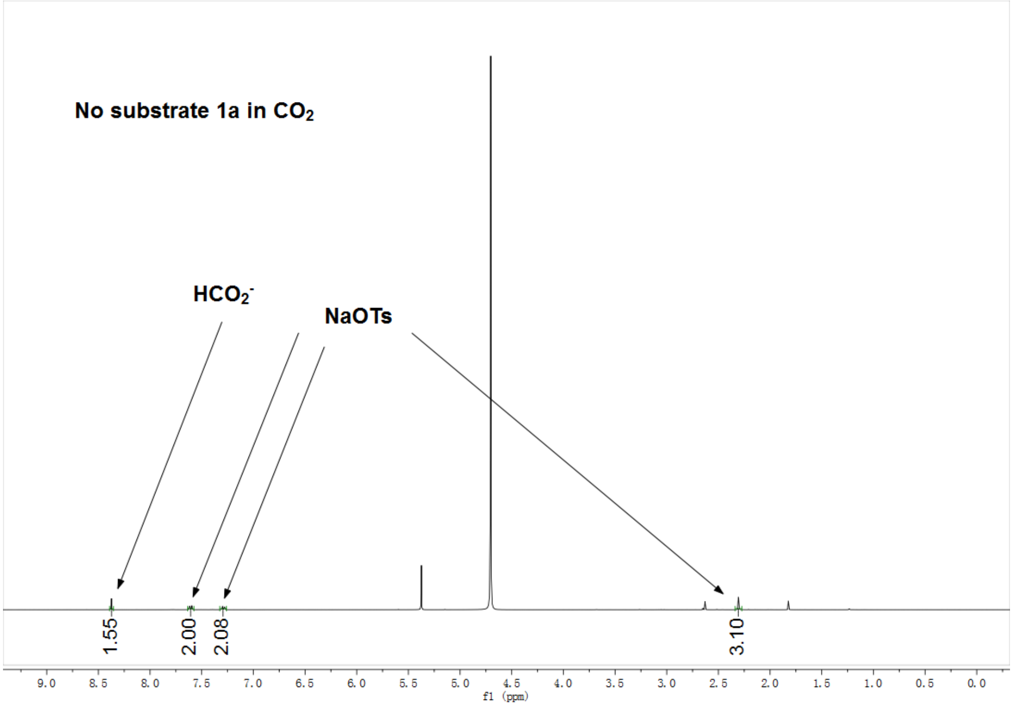

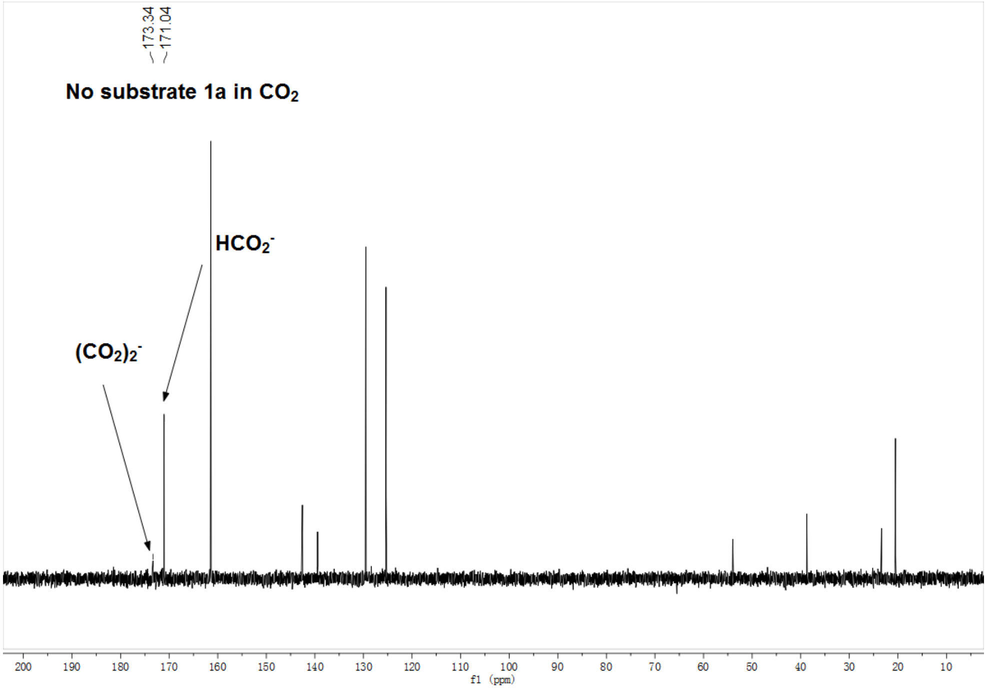

Supplementary Figure 8. Results in the presence of $PhMe_2SiH$ and CO_2.

The reaction of substrate 1a under N_2 atmosphere with silane

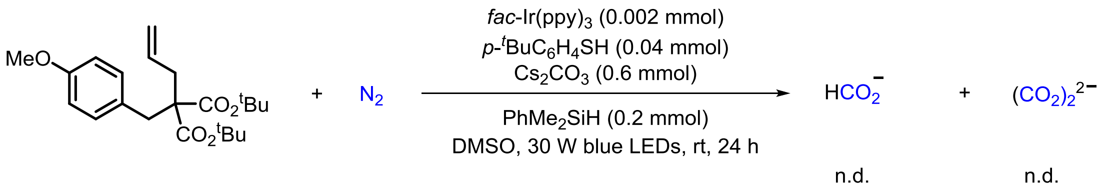

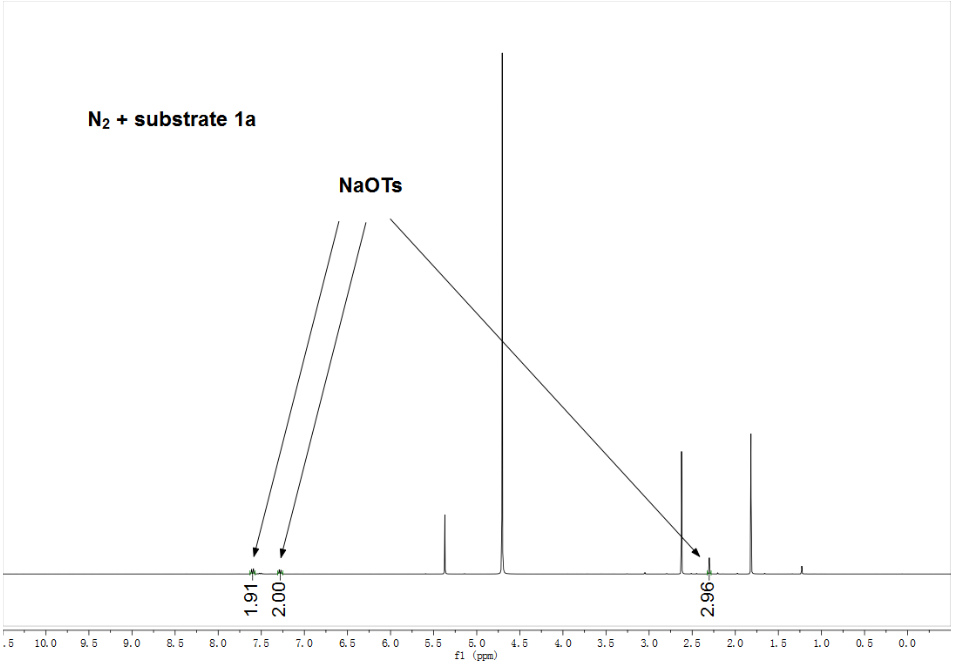

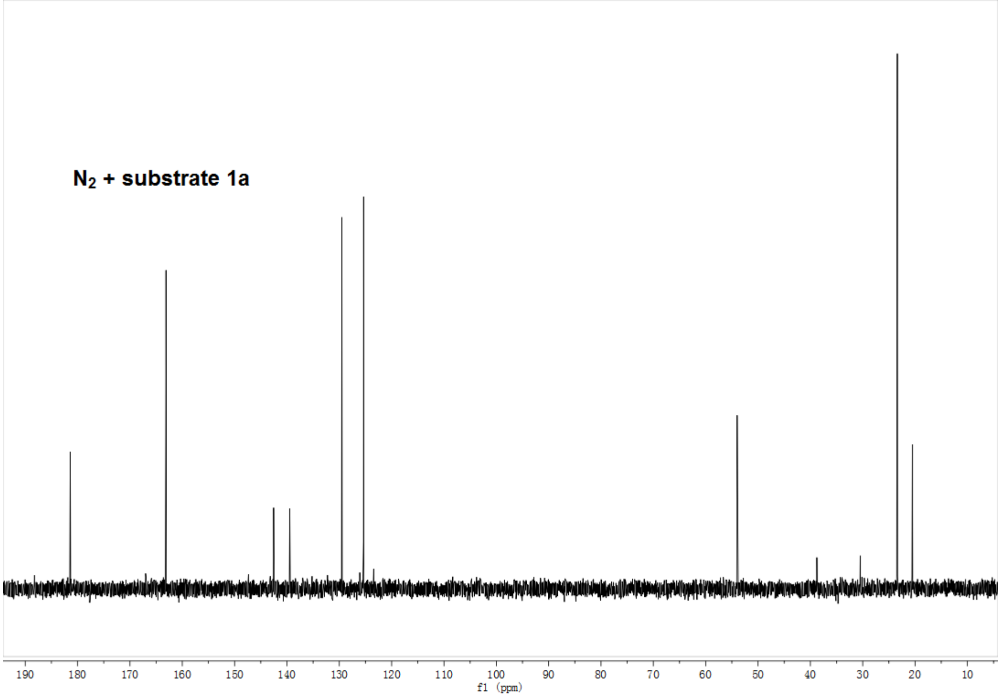

Supplementary Figure 9. Results in the presence of 1a, $PhMe_2SiH$ and N_2.

The reaction w/o substrate 1a under N_2 atmosphere with silane

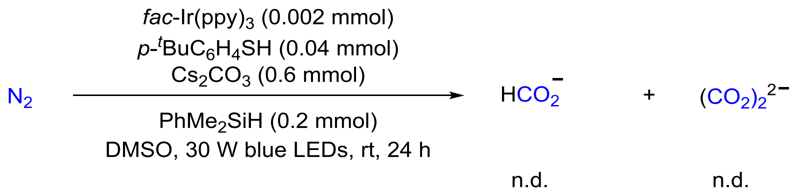

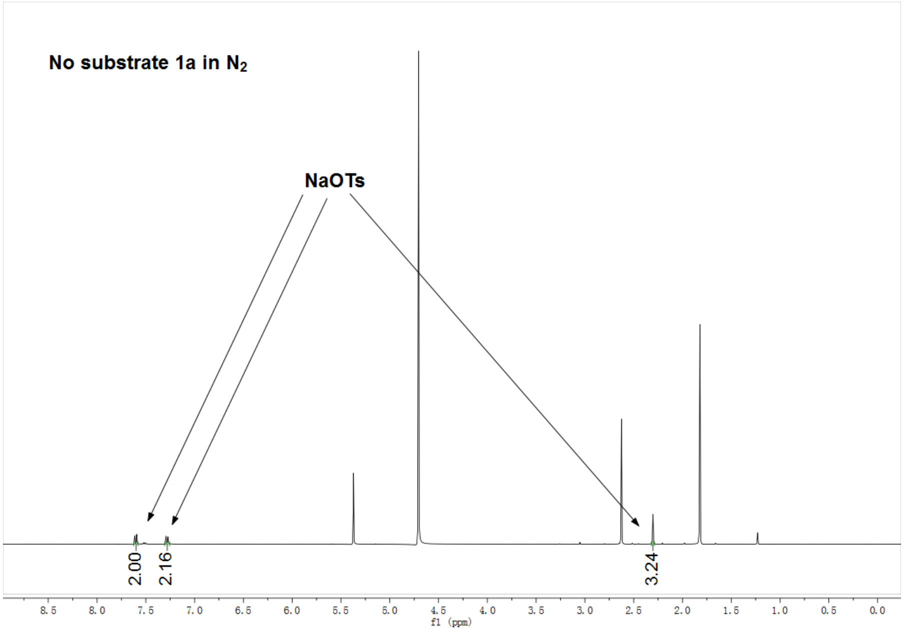

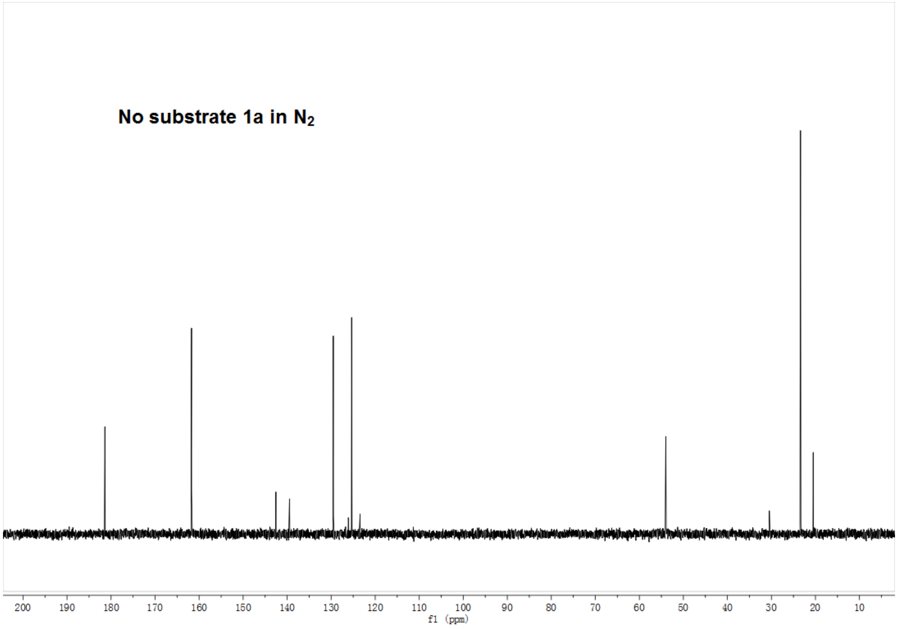

Supplementary Figure 10. Results in the presence of $PhMe_2SiH$ and N_2.

3.1.4 Luminescence quenching experiments

All fluorescence quenching experiments were measured by using RF-5301PC Spectrofluorophotometer. Anhydrous DMSO was degassed by N_2 bubbling for 30 min before use. The photocatalyst *fac*-Ir(ppy)$_3$ was excited at 395 nm and the emission spectrum λ_{max} = 518 nm was recorded. In a typical experiment, the amount of unactivated alkene **1a**, *p*-^{t}BuC$_6$H$_4$SH or *p*-^{t}BuC$_6$H$_4$SK was added to a 3 mL (1.0×10^{-4} M) solution of *fac*-Ir(ppy)$_3$ in DMSO in the 3.5 mL quartz cuvette (d = 1 cm) and covered with Teflon cap in the glovebox. Then the emission spectrum of the solution was collected at each addition.

The concentrate of **1a**, *p*-^{t}BuC$_6$H$_4$SH and *p*-^{t}BuC$_6$H$_4$SK are 0.2 M, 0.2 M, and 0.05 M, respectively.

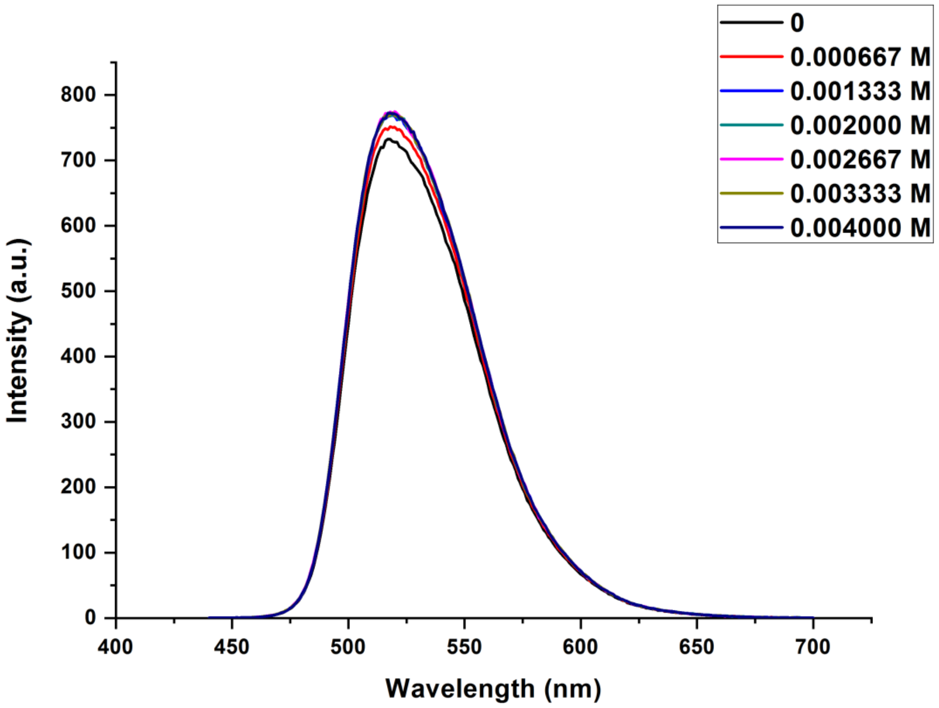

(a) *fac*-Ir(ppy)$_3$ (1.0×10^{-4} M) with **1a** (0-0.004 M) in DMSO

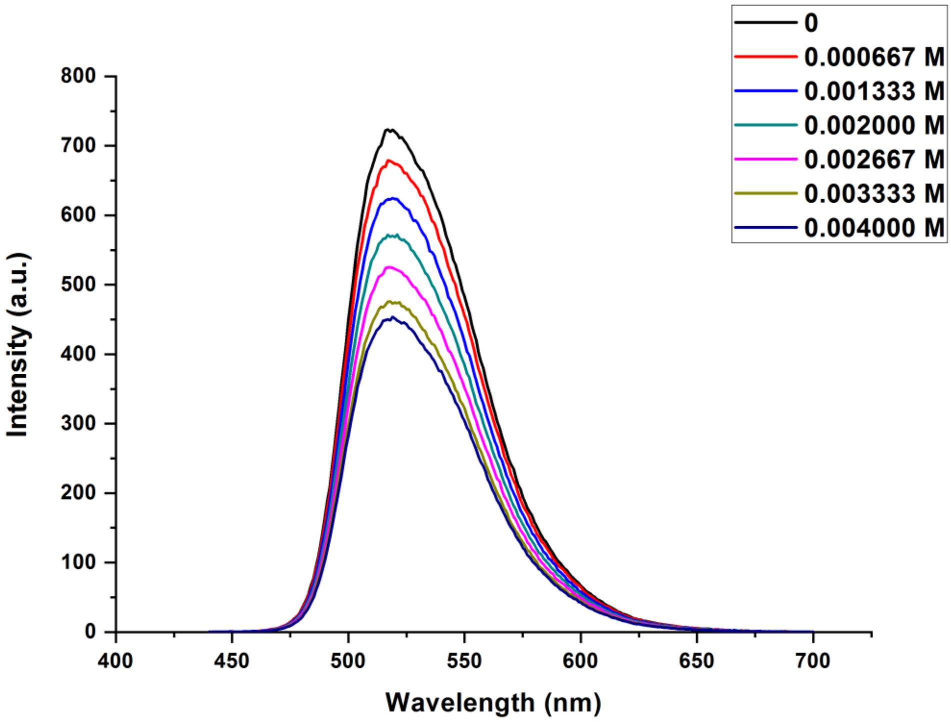

(b) *fac*-Ir(ppy)$_3$ (1.0 × 10^{-4} M) with *p*-^{t}BuC_6H_4SH (0-0.004 M) in DMSO

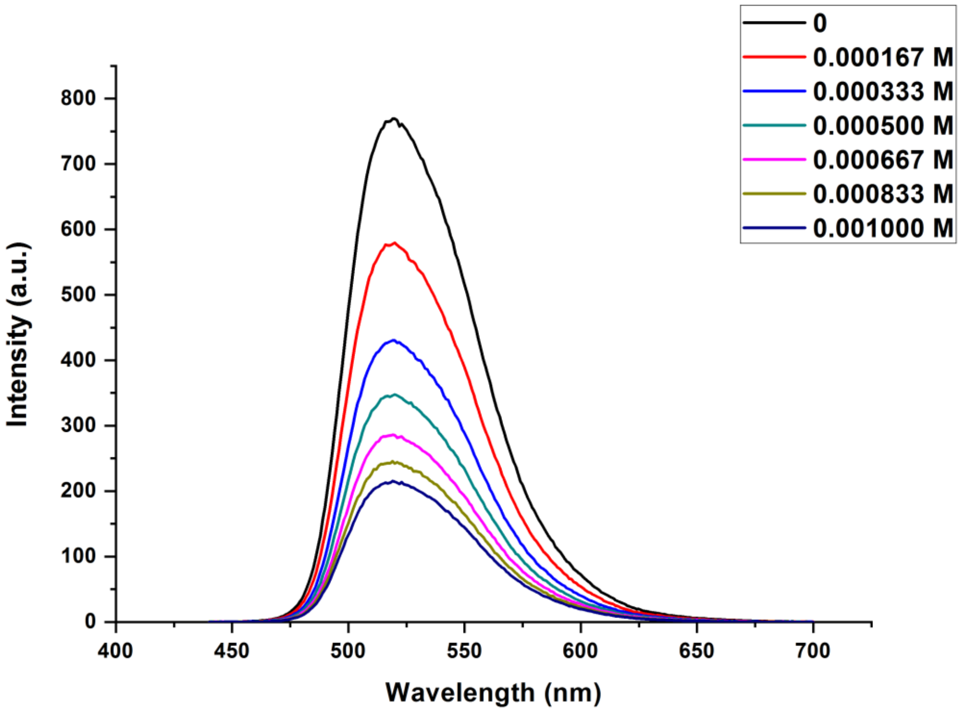

(c) *fac*-Ir(ppy)$_3$ (1.0 × 10^{-4} M) with *p*-^{t}BuC_6H_4SK (0-0.001 M) in DMSO

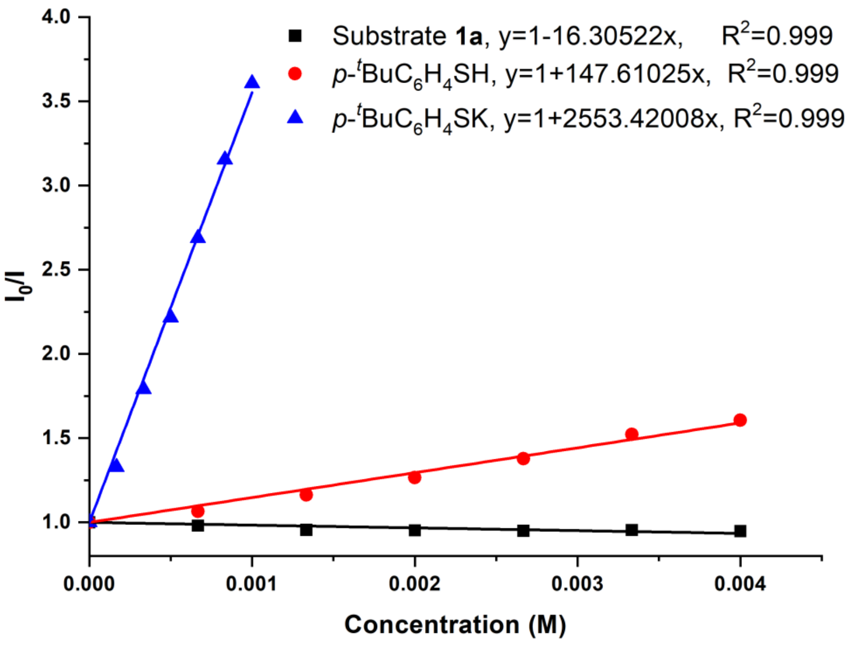

(d) Stern-Volmer quenching plots

Supplementary Figure 11. **Luminescence quenching experiments**: (a) *fac*-Ir(ppy)3 (1.0 × 10^{-4} M) with **1a** (0-0.004 M) in DMSO. (b) *fac*-Ir(ppy)3 (1.0 × 10^{-4} M) with *p*-^{t}BuC6H4SH (0-0.004 M) in DMSO. (c) *fac*-Ir(ppy)3 (1.0 × 10^{-4} M) with *p*-^{t}BuC6H4SK (0-0.001 M) in DMSO. (d) Stern-Volmer quenching plots of the above quenching experiments.

3.1.5 Evidence of possible HAT process

Procedure: To an oven-dried Schlenk tube (25 mL) equipped with a magnetic stir bar were added the **1a** (0.2 mmol, 75.2 mg) and *fac*-Ir(ppy)3 (1 mol%, 1.3 mg, 1 mol%). The tube was moved into the glovebox where was added the Cs_2CO_3 (0.6 mmol, 195.5 mg, 3.0 equiv). The tube was sealed and removed from the glovebox, then evacuated and back-filled with CO_2 atmosphere three times. Anhydrous DMSO (2 mL) and 4-*tert*-butylthiophenol (0.04 mol, 6.7 mg, 7.0 μL, 20 mol%) were added under CO_2 atmosphere, and the tube was sealed at atmospheric pressure of CO_2 (1 atm). The reaction was stirred and irradiated with a 30 W blue LED lamp (1 cm away, with a cooling fan to keep the reaction temperature at 25-30 ℃ and keeping the reaction region located in the center of LEDs lamp) for 24 hours. Upon completion of the reaction, MeI (0.4 mmol, 25 μL, 2.0 equiv) was added under air or N_2 atmosphere, respectively. After

stirring at 65 °C for 3 hours, the resulting mixture was concentrated *in vacuo* under air or N_2 atmosphere, respectively. The residue was analyzed by ^{1}H NMR with 1,3,5-trimethoxybenzene (0.1 mmol) as the internal standard. The results showed that working up in the air or nitrogen atmosphere did not affect product yields, demonstrating a possible HAT process, rather than oxidative aromatization by oxygen process in the final step.

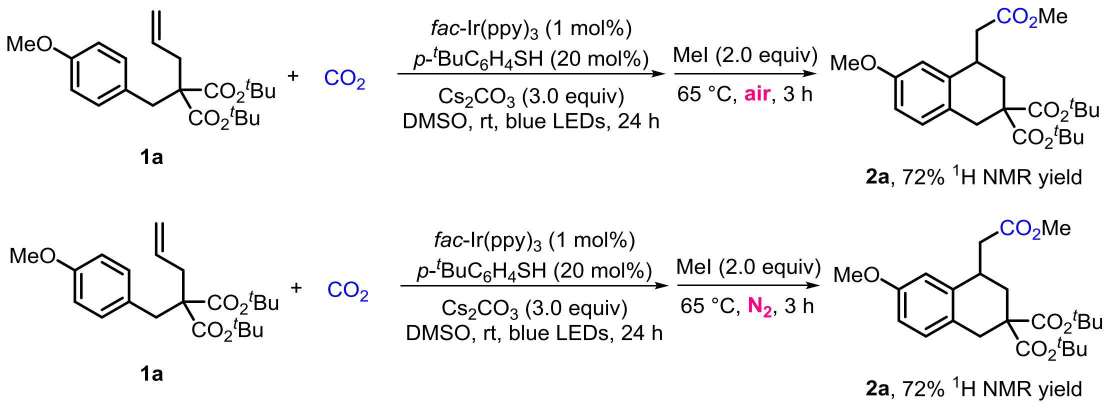

Supplementary Figure 12. Possibility of HAT process in the absence of silanes

3.1.6 NMR spectroscopic evidence of possible ConPET process

To a J-young tube, *fac*-Ir(ppy)$_3$ (1.3 mg, 0.002 mmol) was added. Then, the J-young tube was transferred to the glovebox in which DMSO-d_6 (0.5 mL) and *p*-^{t}BuC_6H_4SK (8.2 mg, 0.004 mmol) were subsequently added.

Step 1: The ^{1}H spectrum was recorded.

Step 2: The ^{1}H spectrum was recorded after the solution was irradiated with 30 W blue LED lamps (1 cm away) for 5 min.

Step 3: Then, the CO_2 was added to the solution in the dark. The ^{1}H spectrum was recorded after the solution was kept in dark for 10 min.

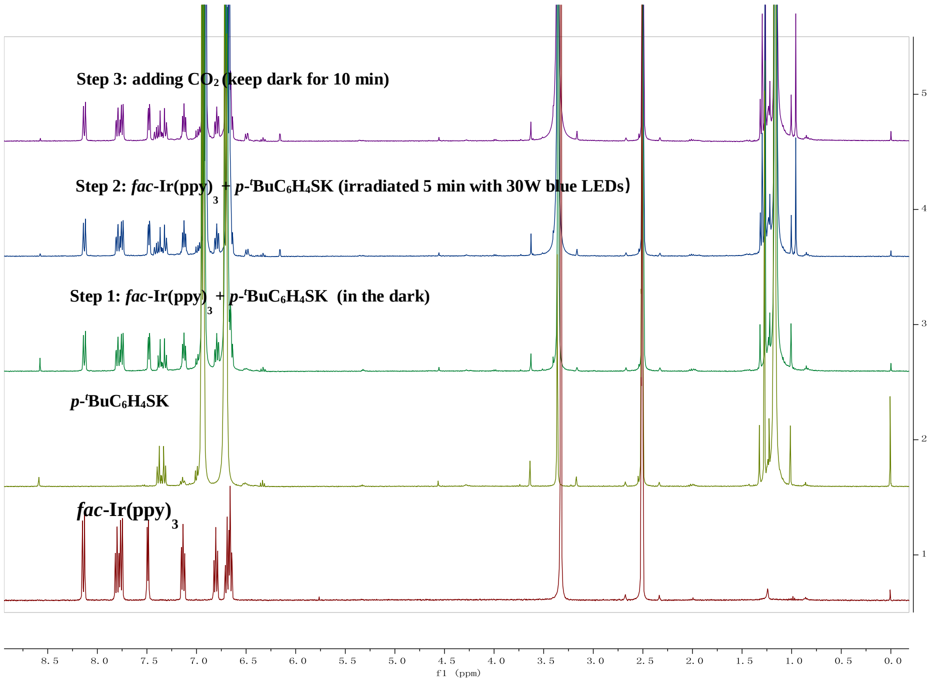

Supplementary Figure 13. **^{1}H NMR investigation for the possible ConPET process**

According to the previous work,[5] We conducted the ^{1}H NMR investigation and the characteristic NMR signals of *fac*-Ir(ppy)$_3$ were detected with a mixture of the photocatalyst and *p*-^{t}BuC$_6$H$_4$SK in DMSO-*d*$_6$. After exposure of the solutions within the NMR tube to 30W blue LED, the signal was not disappeared. The addition of CO_2 showed no effect on the photocatalyst in the absence of light.

Overall, these results did not support the conPET process owing to the insufficient stability of Ir(II) species.

3.1.7 Density Functional Theory (DFT) calculations

Computational Details

Complete authorship of software packages used in this work: Frisch, M. J., Trucks, G. W., Schlegel, H. B., Scuseria, G. E., Robb, M. A., Cheeseman, J. R., Scalmani, G., Barone, V., Petersson, G. A., Nakatsuji, H., Li, X., Caricato, M., Marenich, A. V., Bloino, J., Janesko, B. G., Gomperts, R., Mennucci, B., Hratchian, H. P., Ortiz, J. V., Izmaylov, A. F., Sonnenberg, J. L., Williams, Ding, F., Lipparini, F., Egidi, F., Goings, J., Peng, B., Petrone, A., Henderson, T., Ranasinghe, D., Zakrzewski, V. G., Gao, J., Rega, N., Zheng, G., Liang, W., Hada, M., Ehara, M., Toyota, K., Fukuda, R., Hasegawa, J., Ishida, M., Nakajima, T., Honda, Y., Kitao, O., Nakai, H., Vreven, T.,

Throssell, K., Montgomery Jr., J. A., Peralta, J. E., Ogliaro, F., Bearpark, M. J., Heyd, J. J., Brothers, E. N., Kudin, K. N., Staroverov, V. N., Keith, T. A., Kobayashi, R., Normand, J., Raghavachari, K., Rendell, A. P., Burant, J. C., Iyengar, S. S., Tomasi, J., Cossi, M., Millam, J. M., Klene, M., Adamo, C., Cammi, R., Ochterski, J. W., Martin, R. L., Morokuma, K., Farkas, O., Foresman, J. B. & Fox, D. J. *Gaussian 16, Rev. A.03*. (Gaussian, Inc., **2016**)

Computational Methods

All the DFT calculations were carried out with the GAUSSIAN 16 series of programs. All the geometry optimizations and frequency calculations were performed with B3LYP functional[6,7,8] and D3 version of Grimme's dispersion corrections with Becke-Johnson damping[9] in DMSO, at the SDD basis set for Ir and 6-31+g(d) basis set for the other atoms by using the Solvation Model based on Density (SMD)[10] with keyword in the Gaussian code route section "SCRF = (SMD, Solvent = dimethylsulfoxide)". The vibrational frequencies were computed at the same level of theory as for the geometry optimizations to confirm whether each optimized structure is an energy minimum or a transition state, and to evaluate the zero-point vibrational energy (ZPVE) and thermal corrections. The thermal corrections to 6-31+g(d) Gibbs free energies were calculated at 298.15 K. The M06 functional[11] in combination with the SDD basis set for Ir and 6-31+g(d) basis set for the other atoms were used to calculate the solvation single point energies to give more accurate energy information. The solvent effects were considered with an SMD solvation model in the DMSO solvent. The 3D diagrams of molecules were generated using CYLView.[12]

Discussion on the generation of CO_2 radical anion ($CO_2^{\bullet-}$) as the possible intermediate to facilitate the radical addition pathway to form the final arylcarboxylated product.

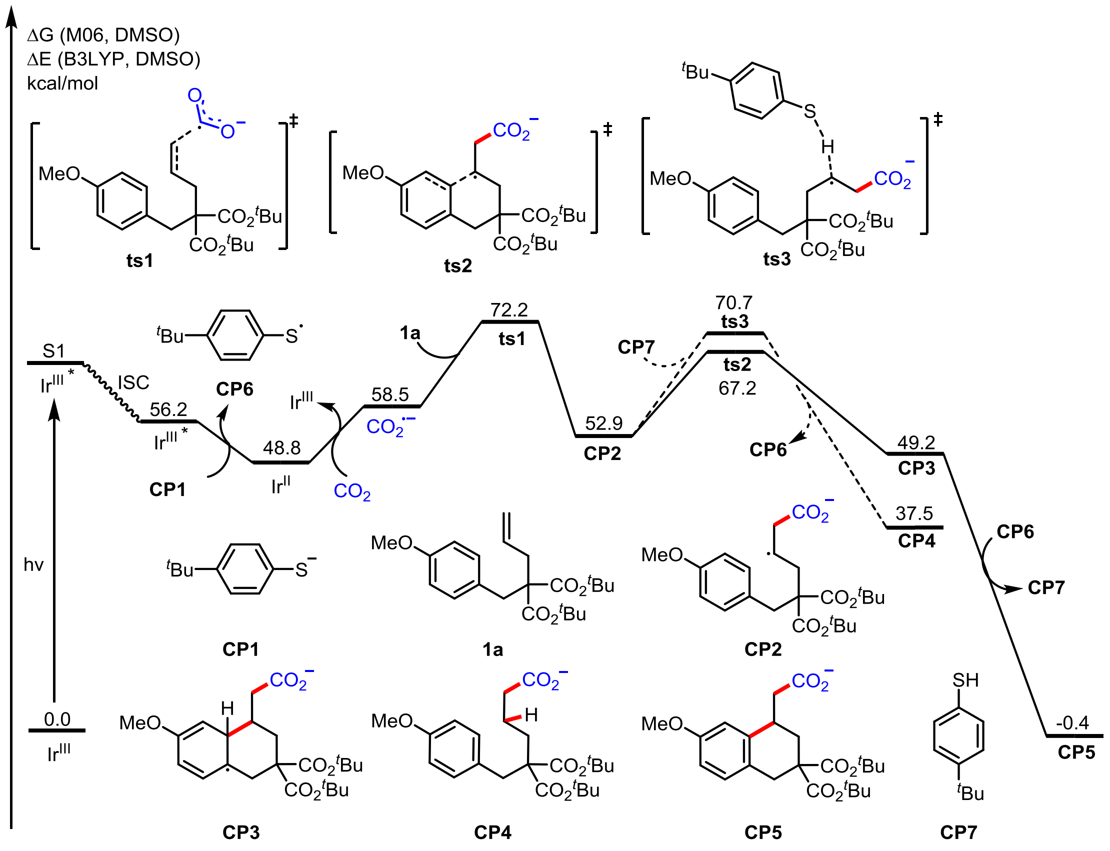

Supplementary Figure 14. The DFT calculated free energy profiles for the arylcarboxylation of unactivated alkenes with CO_2 via visible-light photoredox catalysis. The favored pathway is labeled by solid lines. The values given in kcal/mol are the relative free energies calculated by the M06/6-311+G(d,p)//B3LYP/6-31+G(d) method in DMSO solvent.

Photocatalyst *fac*-Ir^{III}(ppy)$_3$ in ground state was set as the relative zero to calculate free energy profiles. The reduction of excited *fac*-Ir^{III}(ppy)$_3$ at triplet state by thiolate could deliver *fac*-Ir^{II}(ppy)$_3$ and a thiyl radical. This step is 5.4 kcal/mol exergonic. The redox potentials of *fac*-Ir^{II}(ppy)$_3$ ($E_{1/2}^{III/II}$ = -2.19 V vs SCE) and CO_2 ($CO_2/CO_2^{\bullet-}$) = -2.21 V vs SCE] are very near, thus the SET reduction between *fac*-Ir^{II}(ppy)$_3$ and CO_2 might be difficult. Our DFT calculations show that this step is 9.7 kcal/mol endergonic, leading to the formation of corresponding CO_2 radical anion with the generation of the *fac*-Ir^{III}(ppy)$_3$. Subsequently, CO_2 radical anion can undergo radical addition to alkene **1a** via transition state **ts1** with an energy barrier of 13.7 kcal/mol. This step is exergonic 5.6 kcal/mol releasing radical **CP2**. The overall activation free energy is 23.4 kcal/mol, which could be considered to be the rate-limit for the whole transformation. Further 6-endo-trig radical addition can occur via **ts2** with an energy barrier of 14.3 kcal/mol to

form **CP3** by 3.7 kcal/mol exergonic. Driven by the aromatization in the intermediate **CP3**, an intermolecular HAT process could take place to deliver final product by 49.6 kcal/mol exergonic. Thus, the pathway we proposed in Fig8 in the main text involving SET reduction between Ir(II) and CO_2 is reasonable.

3.1.8 Proposed mechanism in the absence of silane

Path I

Based on the experimental results and previous reports, a possible mechanism is proposed. Upon absorption of visible light, the iridium photocatalyst complex *fac*-$Ir^{III}(ppy)_3$ was excited to *fac*-$^{*}Ir^{III}(ppy)_3$, which can function as an oxidant ($E_{1/2}{}^{*III/II}$ = +0.31 V vs SCE) and can be quenched by the thiolate to produce *fac*-$Ir^{II}(ppy)_3$, as well as thiyl radical. In order to close the photoredox cycle, Ir^{II} species ($E_{1/2}{}^{III/II}$ = -2.19 V vs SCE) or $^{*}Ir^{II}$ might reduce the CO_2 [$E_{1/2}$ ($CO_2/CO_2^{\bullet-}$) = -2.21 V vs SCE] via single electron transfer event to deliver $CO_2^{\bullet-}$ along with regeneration of *fac*-$Ir^{III}(ppy)_3$ to close the photoredox catalytic cycle. The generated $CO_2^{\bullet-}$ then undergoes radical addition to the C=C bond of unactivated alkenes **1** to provide a carbon radical **A**, which quickly undergoes cyclization to form the radical intermediate **B**. However, the intermolecular hydrogen atom transfer (HAT) would take place if a higher concentration of hydrogen atom donor exists in the reaction, resulting in anti-Markovnikov hydrocarboxylation byproduct **C**. Finally, the product **2** could be obtained by a HAT process of radical intermediate **B** with the thiyl radical along with regeneration of thiol catalyst.

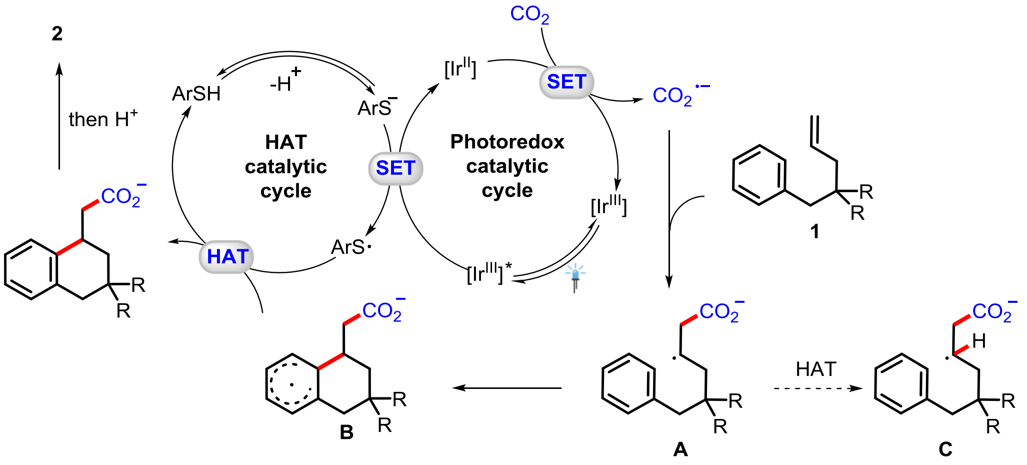

Supplementary Figure 15. Proposed path I for the arylcarboxylation reaction in the absence of silane

Path II

As intermediate **B** might be oxidized by *fac*-*$Ir^{III}(ppy)_3$ to form the carbocation intermediate and regenerate Ir^{II} species, we also proposed the path II as following.

The irradiation of photocatalyst *fac*-$Ir^{III}(ppy)_3$ generates excited *fac*-*$Ir^{III}(ppy)_3$, which can be reductively quenched by a thiolate to furnish *fac*-$Ir^{II}(ppy)_3$ and a thiyl radical. Then, the Ir^{II} species engage in reducing CO_2 via SET event to deliver $CO_2^{\bullet-}$ along with regeneration of *fac*-$Ir^{III}(ppy)_3$ to close the first photoredox catalytic cycle. The *in situ* generated $CO_2^{\bullet-}$ is trapped by unactivated alkene **1** to form an alkyl carbon radical **A**, which is supposed to be quickly captured via cyclization to form the radical intermediate **B**. In the second photoredox catalytic cycle, the intermediate **B** is oxidized by *fac*-*$Ir^{III}(ppy)_3$ to form the intermediate carbocation **C** and Ir^{II} species. Finally, the carbocation **C** is deprotonated to give the product **2**.

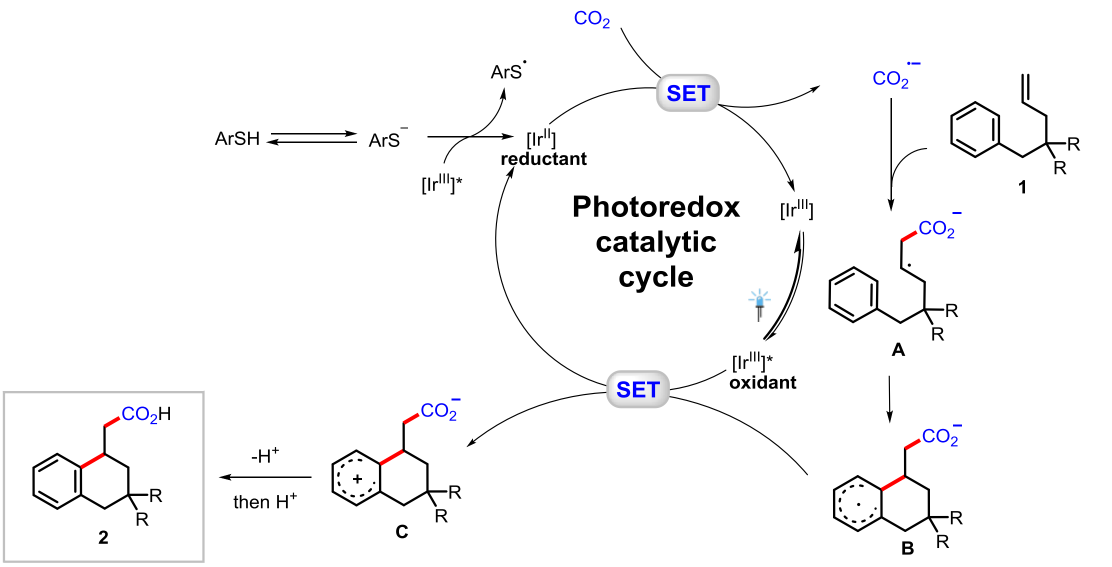

Supplementary Figure 16. Proposed path II for the arylcarboxylation reaction in the absence of silane

In this pathway, thiolate acts as the reductive quencher to initiate the reaction. As Hantzsch ester might also act the similar role, we investigated the action of Hantzsch ester, which is reported to reductively quench the same Ir-photocatalyst and should act similarly in this reaction.[4]

All fluorescence quenching experiments were measured by using RF-5301PC Spectrofluorophotometer. Anhydrous DMSO was degassed by N_2 bubbling for 30 min before use. The photocatalyst *fac*-$Ir(ppy)_3$ was excited at 395 nm and the emission

spectrum λ_{max} = 518 nm was recorded. In a typical experiment, the amount of HE was added to a 3 mL (1.0 × 10^{-4} M) solution of *fac*-Ir(ppy)$_3$ in DMSO in the 3.5 mL quartz cuvette (d = 1 cm) and covered with Teflon cap in the glovebox. Then the emission spectrum of the solution was collected at each addition. The concentration of HE is 0.005 M.

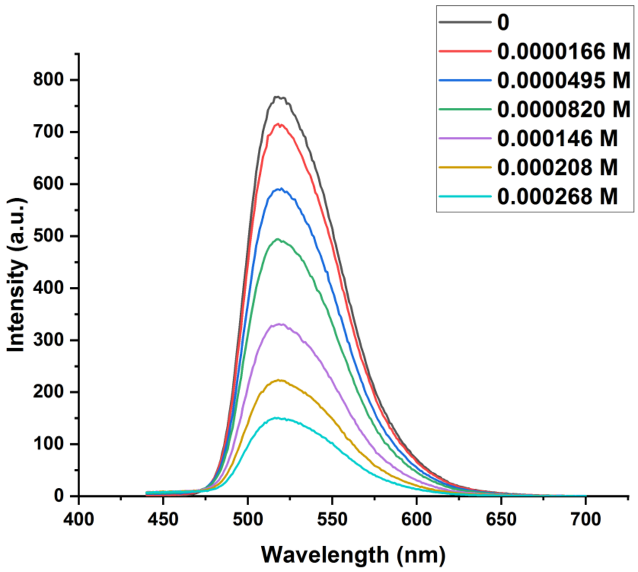

(a) *fac*-Ir(ppy)$_3$ (1.0 × 10^{-4} M) with HE (0-0.000268 M) in DMSO

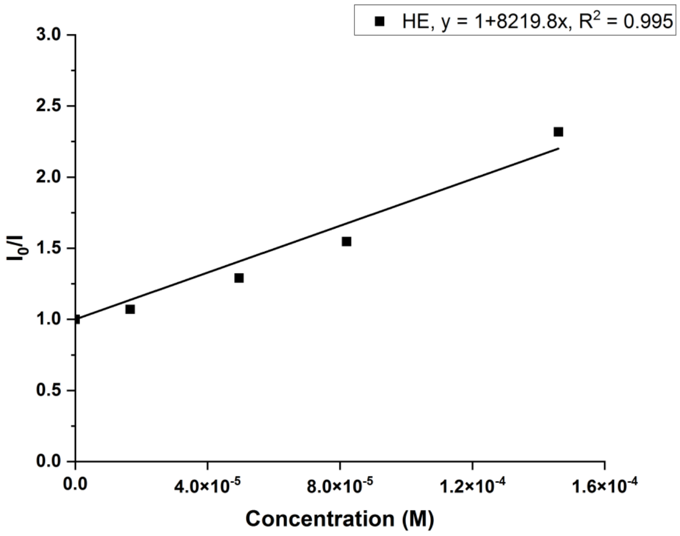

(b) Stern-Volmer quenching plots

Supplementary Figure 17. **Luminescence quenching experiments**: (a) *fac*-Ir(ppy)$_3$ (1.0 × 10^{-4} M) with HE (0-0.000268 M) in DMSO. (b) Stern-Volmer quenching plots of the HE.

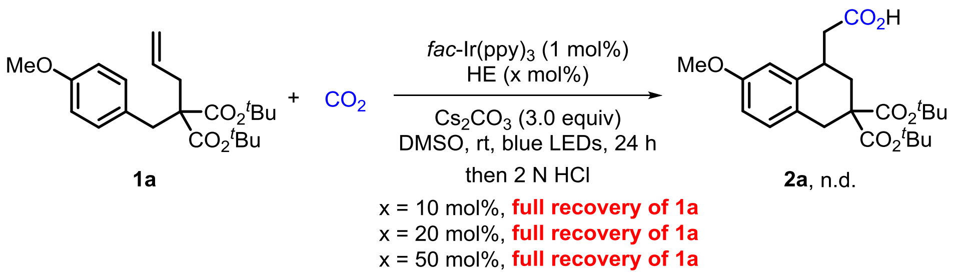

According to fluorescence quenching experiments and previous reports,[2] we find that *fac*-$^{*}Ir^{III}(ppy)_3$ can be reductively quenched by the Hantzsch ester to deliver Ir^{II} and we speculated that Ir^{II} could undergo a similar process in the later step. However, a different amount of HE instead of *p*-$^{t}BuC_6H_4SH$ was added to the reaction. The desired product **2a** was not detected by ESI-MS along with full recovery of **1a**, which suggested that path II is not reliable. Overall, the above experimental results indicated that intermediate **B** might be engaged in the HAT process rather than oxidative aromatization by *fac*-$^{*}Ir^{III}(ppy)_3$ or oxygen. We are more inclined to believe that the HAT process is the main pathway (Path I) of this transformation.

3.1.9 Proposed mechanism in the presence of silane

Upon absorption of visible light, the iridium photocatalyst complex *fac*-$Ir^{III}(ppy)_3$ is excited to *fac*-$^{*}Ir^{III}(ppy)_3$, which can function as an oxidant ($E_{1/2}{}^{*III/II}$ = +0.31 V vs SCE) and can be quenched by the thiolate to produce *fac*-$Ir^{II}(ppy)_3$, as well as thiyl radical. In order to close the photoredox cycle, Ir^{II} species ($E_{1/2}{}^{III/II}$ = -2.19 V vs SCE) might reduce the CO_2 [$E_{1/2}$ ($CO_2/CO_2^{\bullet-}$) = -2.21 V vs SCE] via single electron transfer event to deliver $CO_2^{\bullet-}$ along with regeneration of *fac*-$Ir^{III}(ppy)_3$ to close the photoredox catalytic cycle. The thiyl radical, known as the HAT catalyst, would abstract the hydrogen atom from the formate, which was formed *in situ* between silane and CO_2 to close the HAT catalytic cycle and produce $CO_2^{\bullet-}$. The generated $CO_2^{\bullet-}$ then undergoes radical addition to the C=C bond of unactivated alkenes **1** to provide a carbon radical **A**, which quickly undergoes cyclization to form the radical intermediate **B**. Finally, product **2** could be obtained by a HAT process of radical intermediate **B** with the CO_2 radical anion along with regeneration of formate.

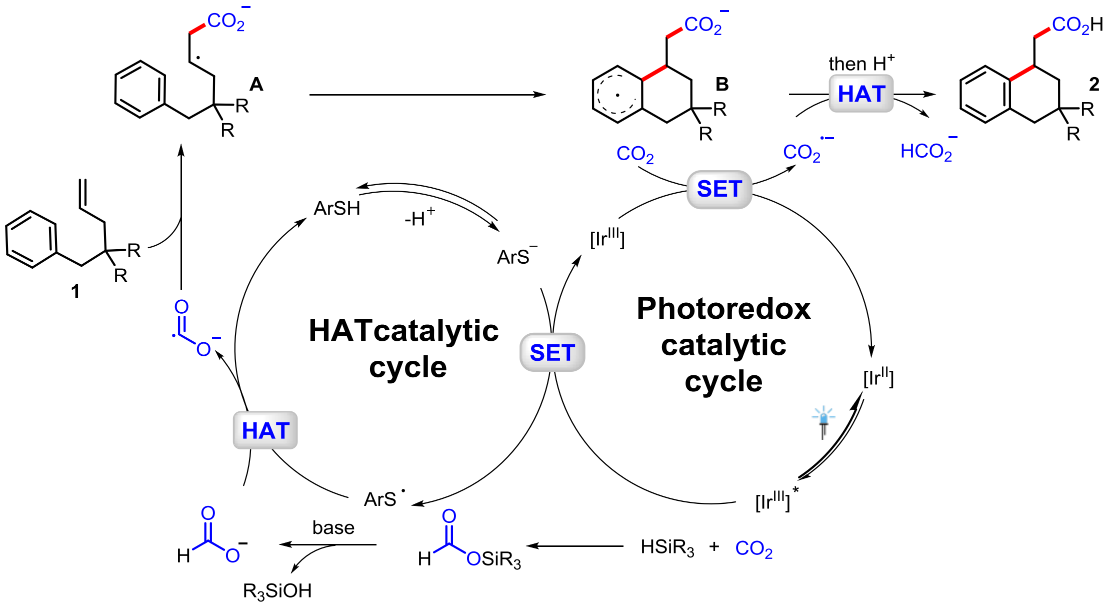

Supplementary Figure 18. **Proposed mechanism for the arylcarboxylation reaction in the presence of silane**

3.2 NMR spectra

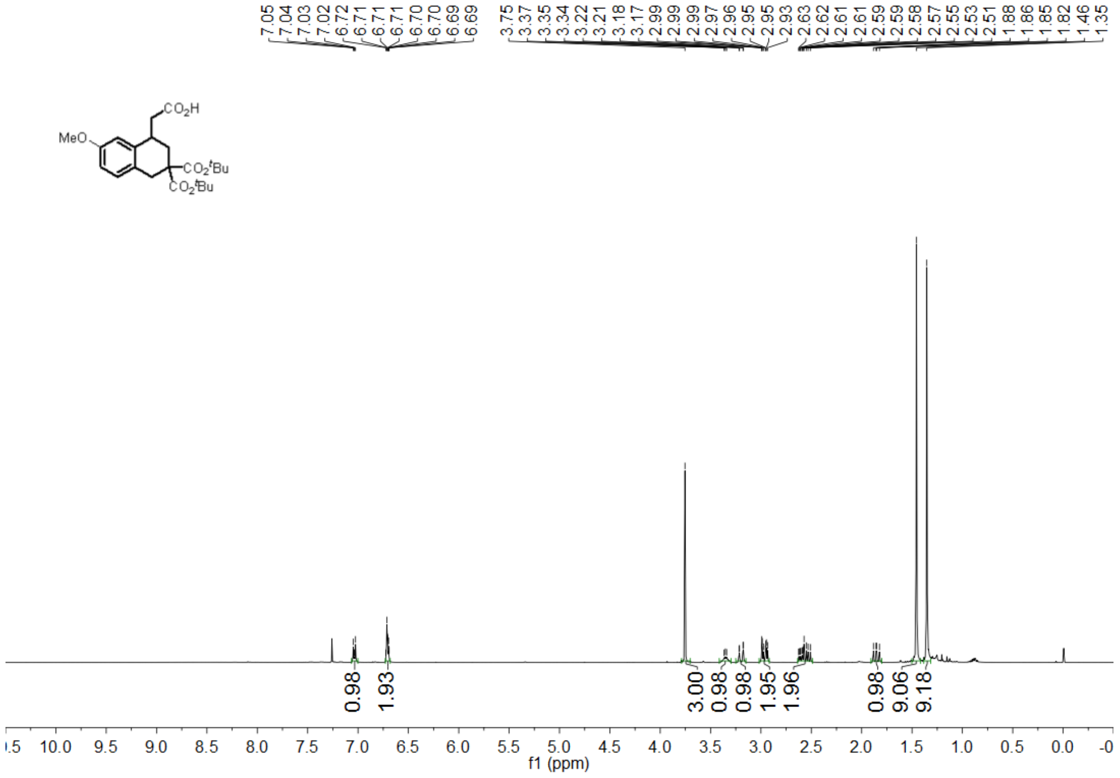

Supplementary Figure 19 ^{1}H NMR spectrum of **2a**

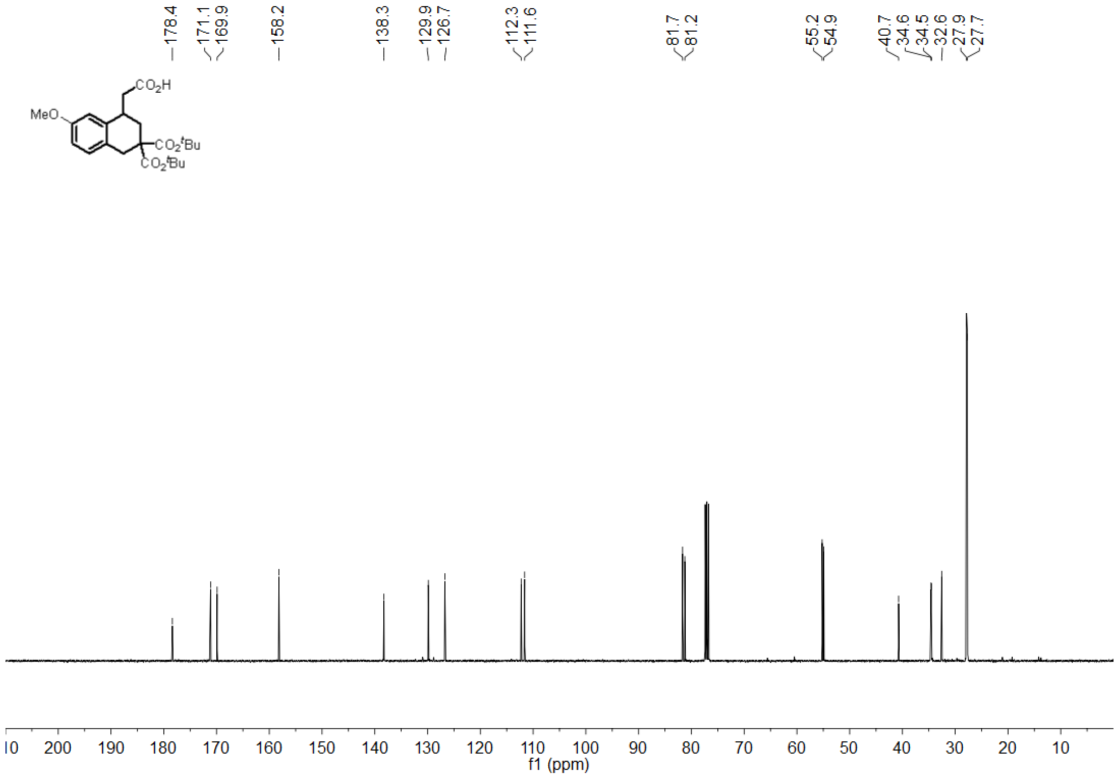

Supplementary Figure 20 ^{13}C NMR spectrum of **2a**

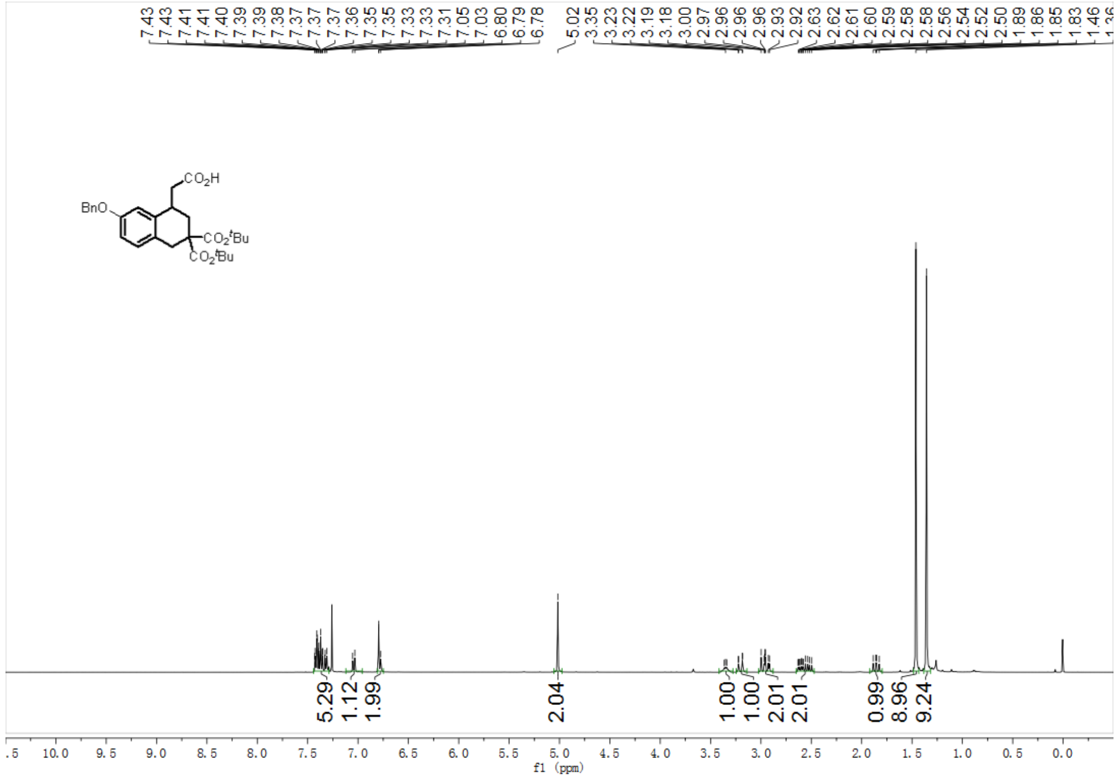

Supplementary Figure 21 ^{1}H NMR spectrum of **2b**

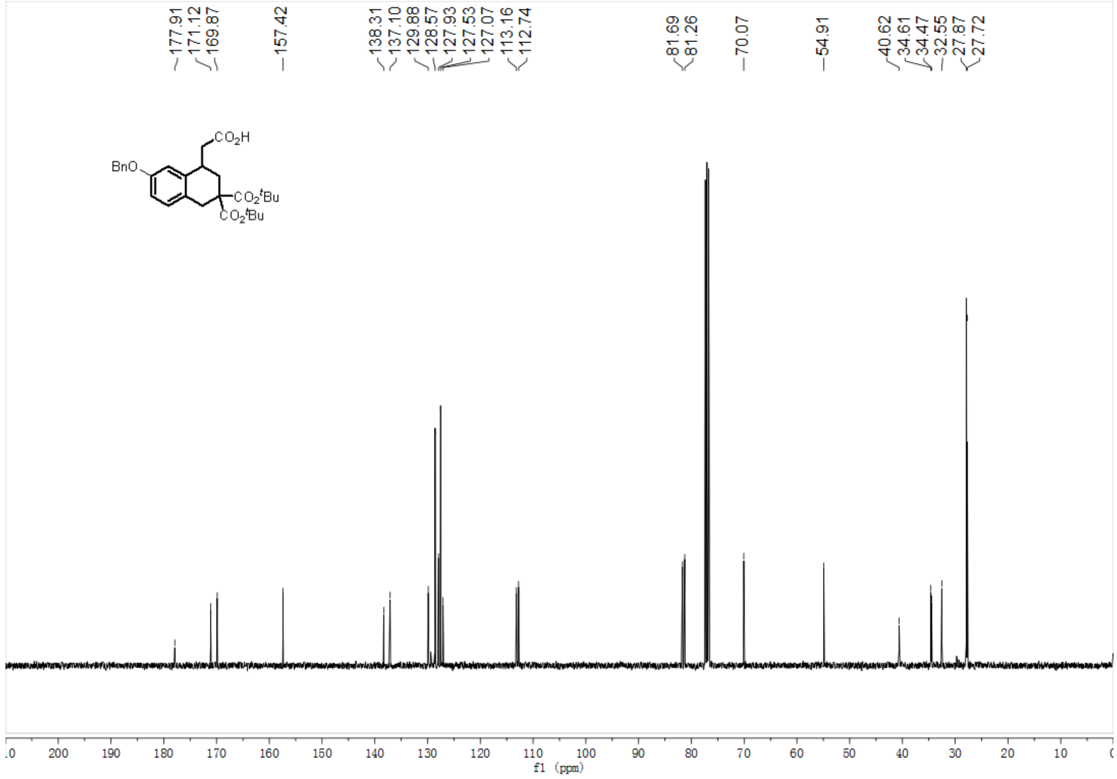

Supplementary Figure 22 ^{13}C NMR spectrum of **2b**

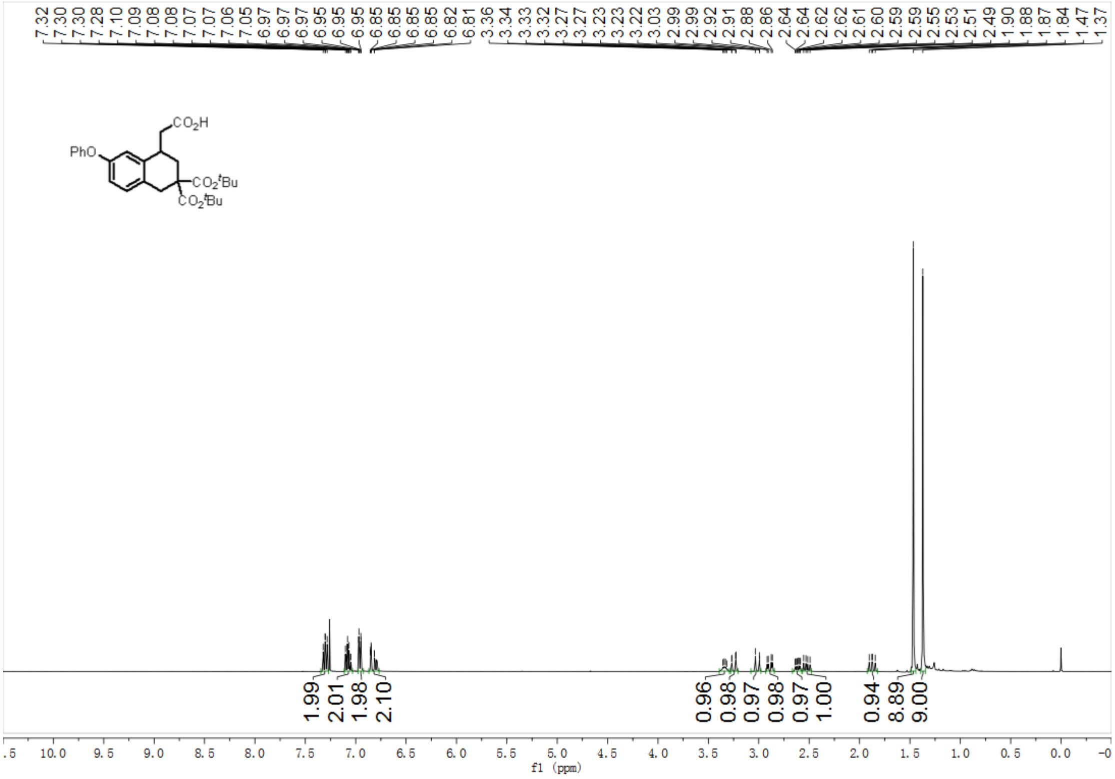

Supplementary Figure 23 ^{1}H NMR spectrum of **2c**

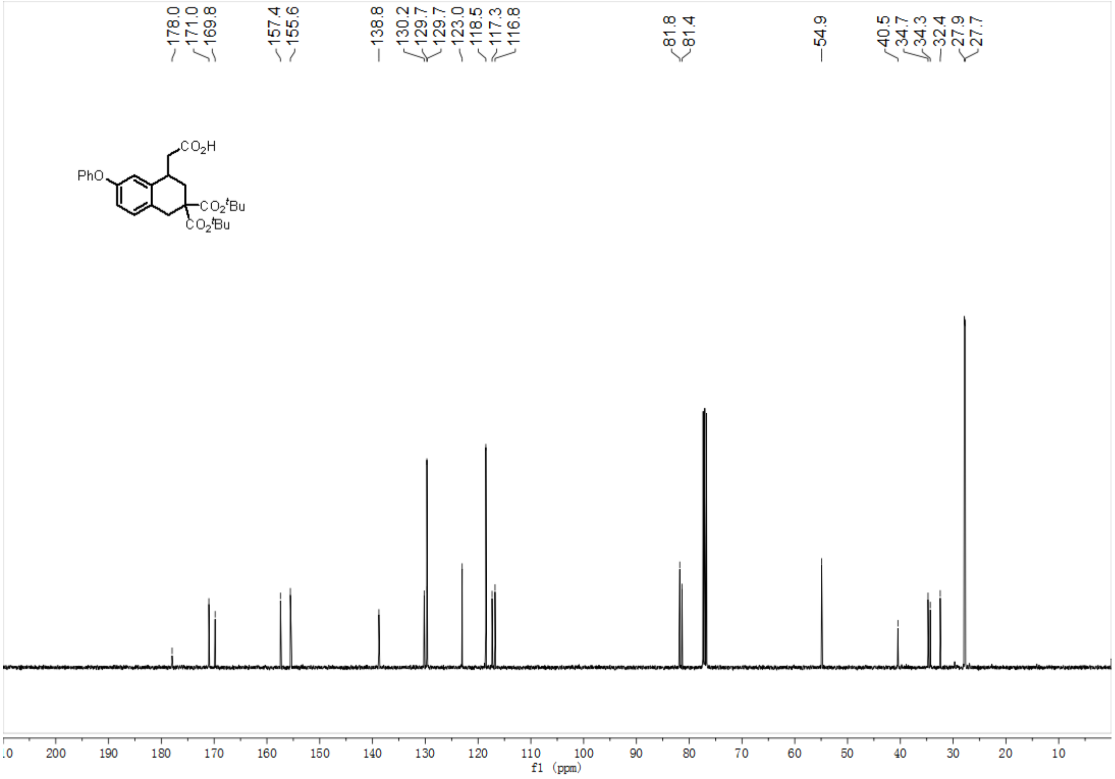

Supplementary Figure 24 ^{13}C NMR spectrum of **2c**

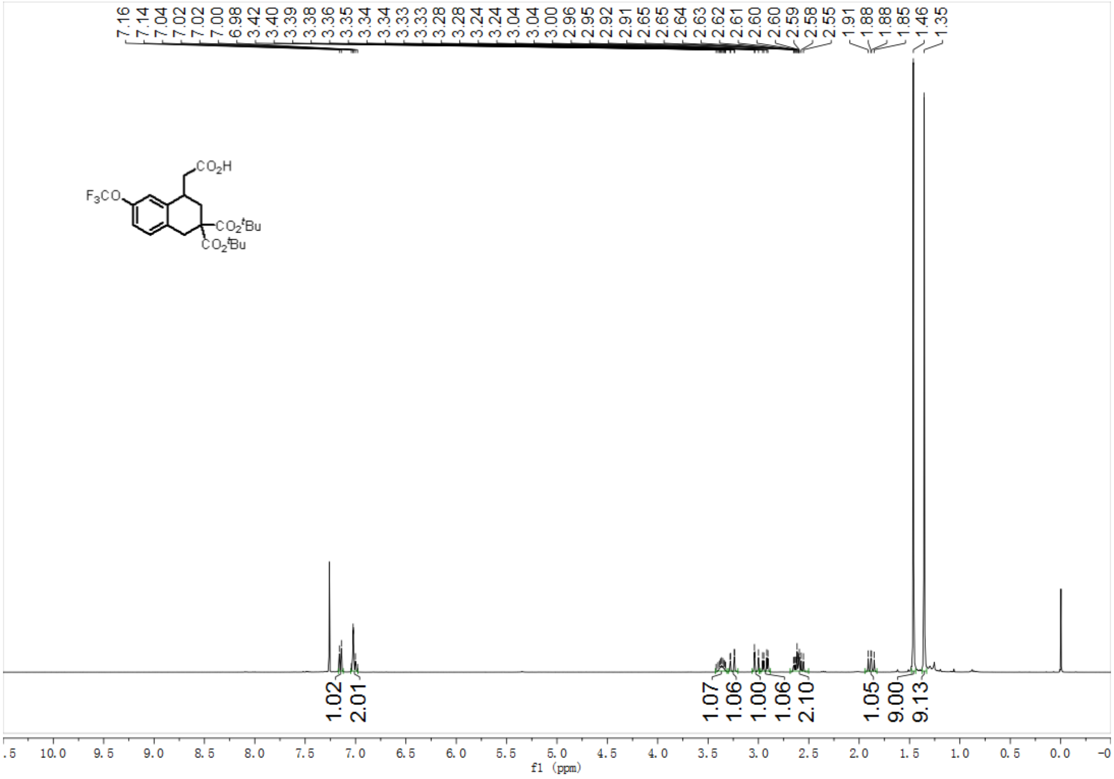

Supplementary Figure 25 ^{1}H NMR spectrum of **2d**

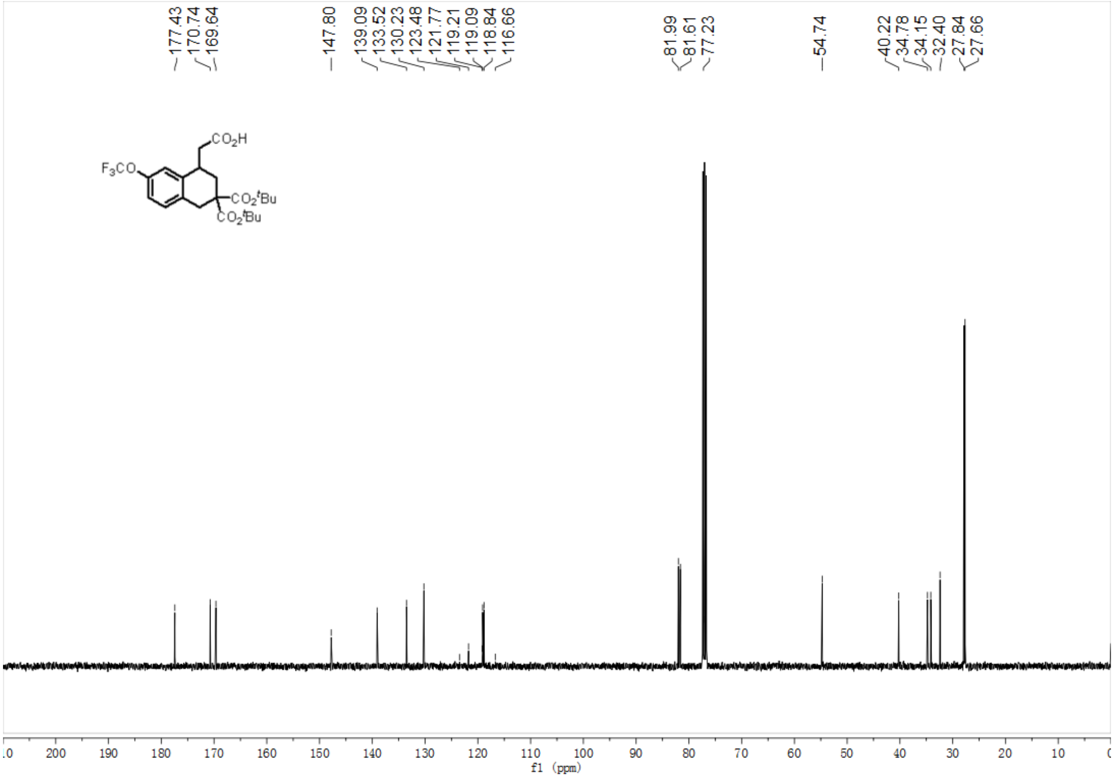

Supplementary Figure 26 ^{13}C NMR spectrum of **2d**

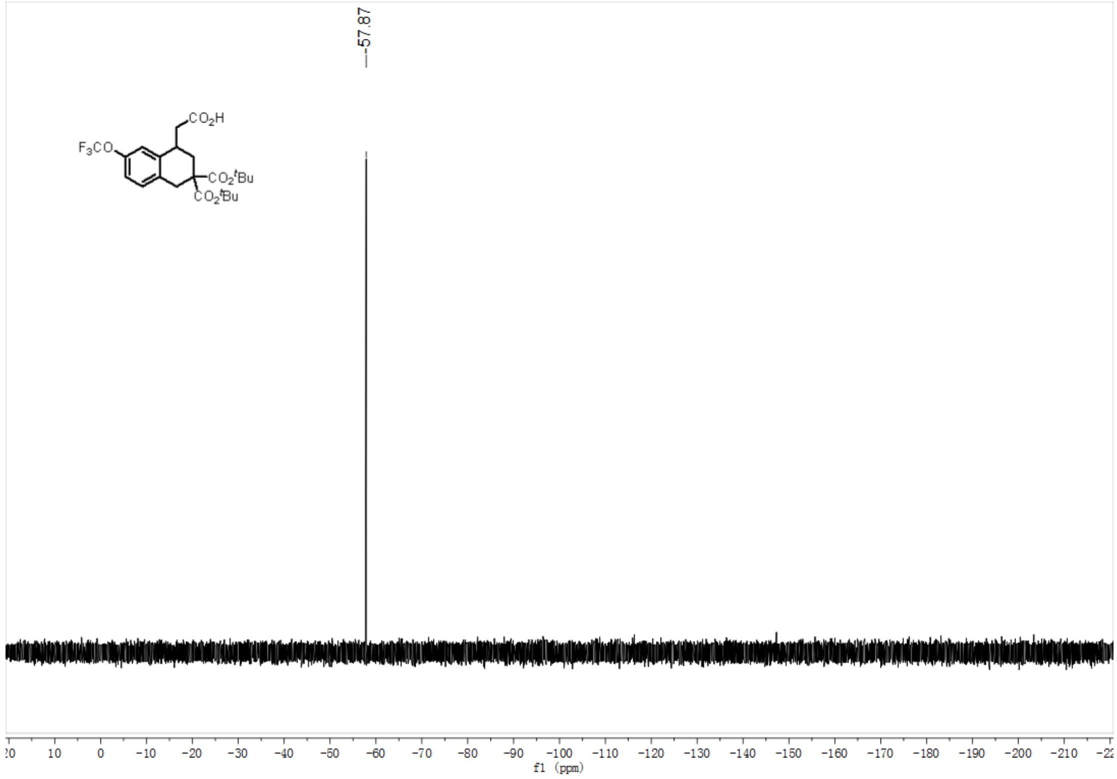

Supplementary Figure 27 ^{19}F NMR spectrum of **2d**

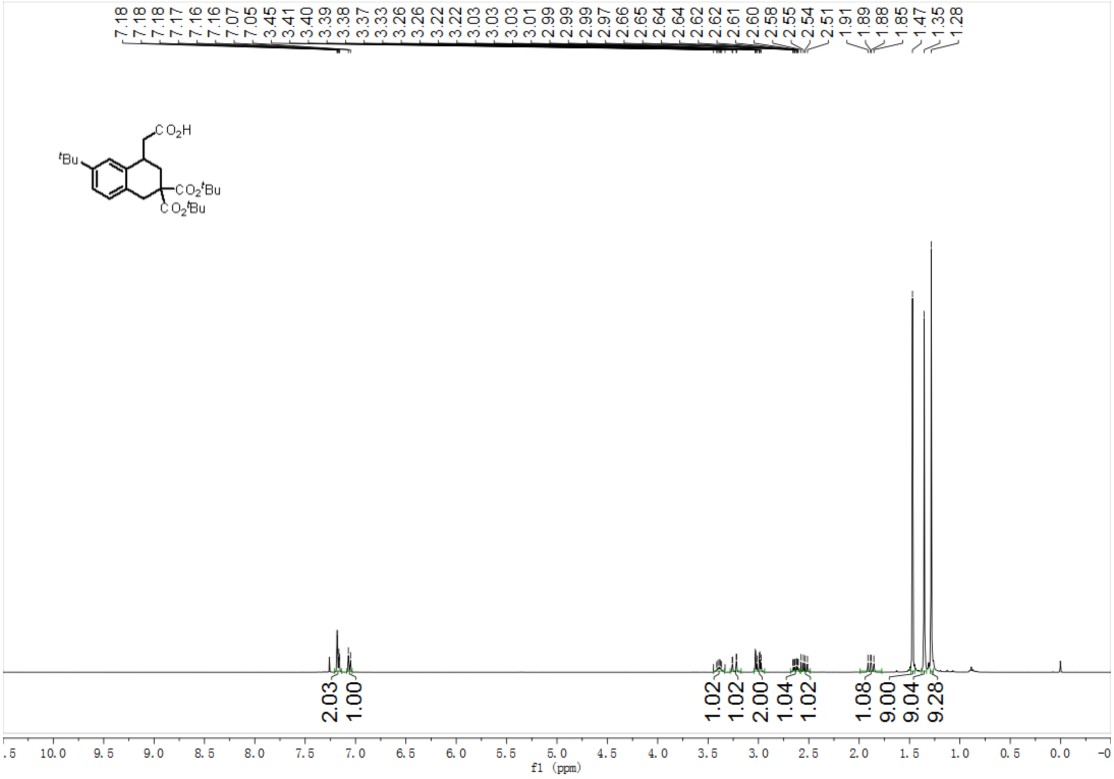

Supplementary Figure 28 ^{1}H NMR spectrum of **2e**

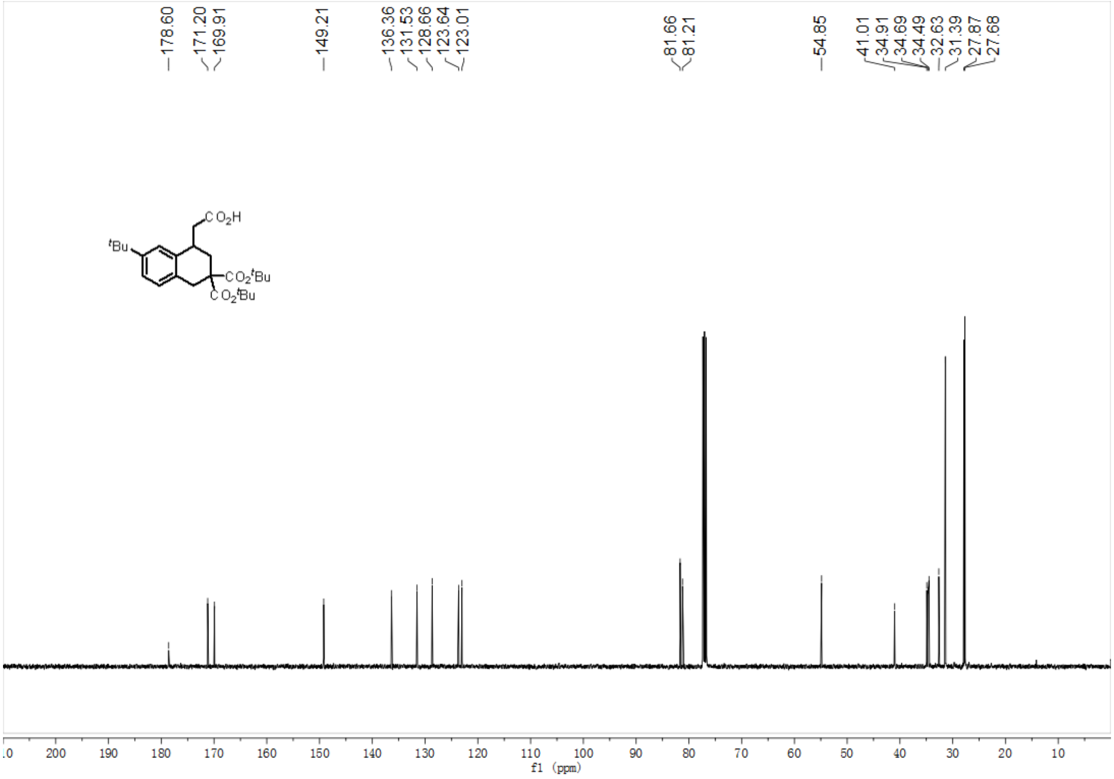

Supplementary Figure 29 ^{13}C NMR spectrum of **2e**

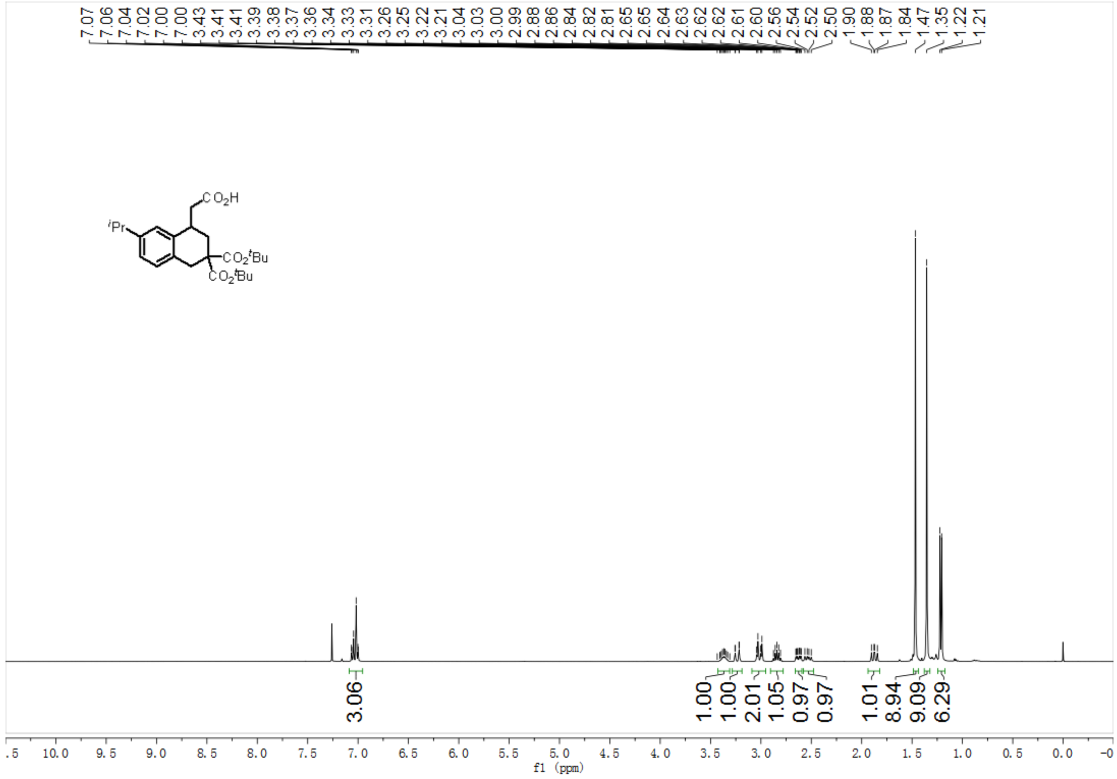

Supplementary Figure 30 ^{1}H NMR spectrum of **2f**

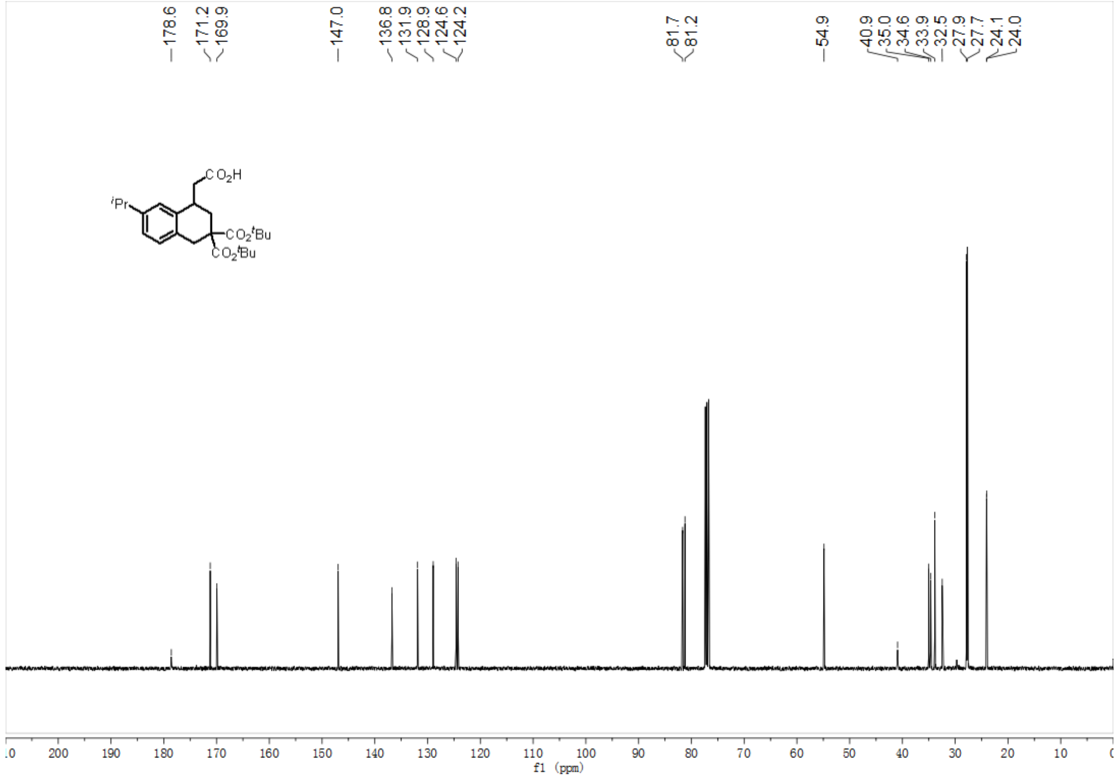

Supplementary Figure 31 ^{13}C NMR spectrum of **2f**

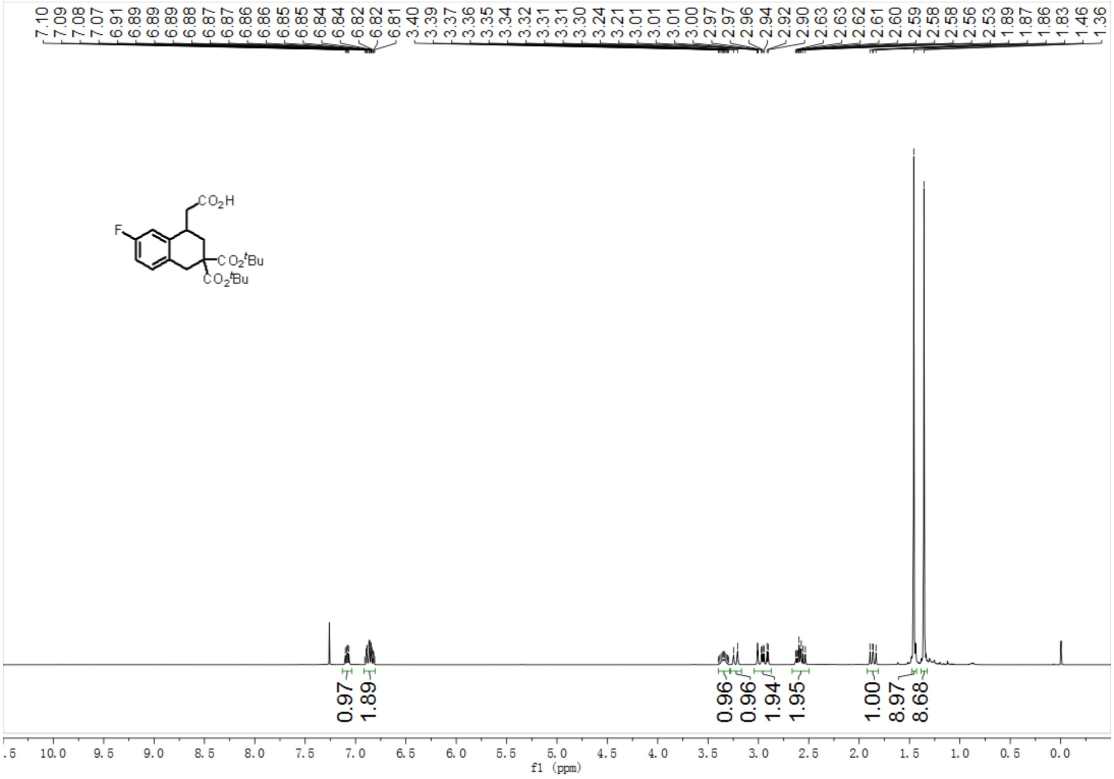

Supplementary Figure 32 ^{1}H NMR spectrum of **2g**

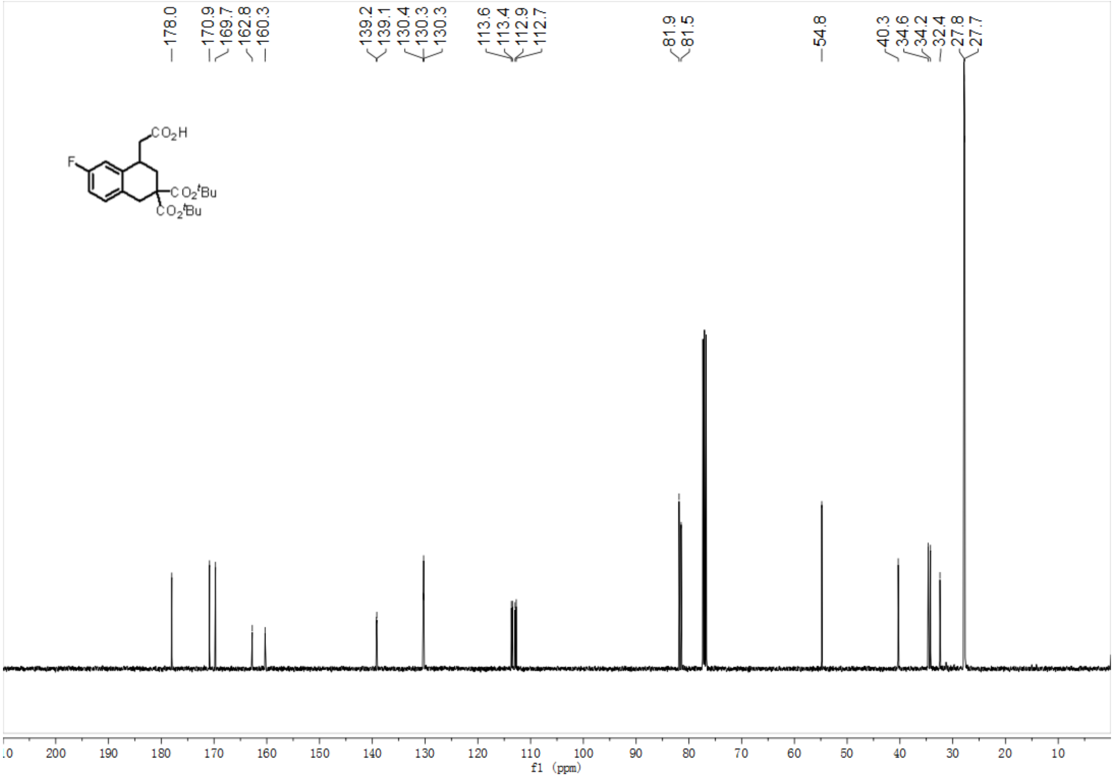

Supplementary Figure 33 ^{13}C NMR spectrum of **2g**

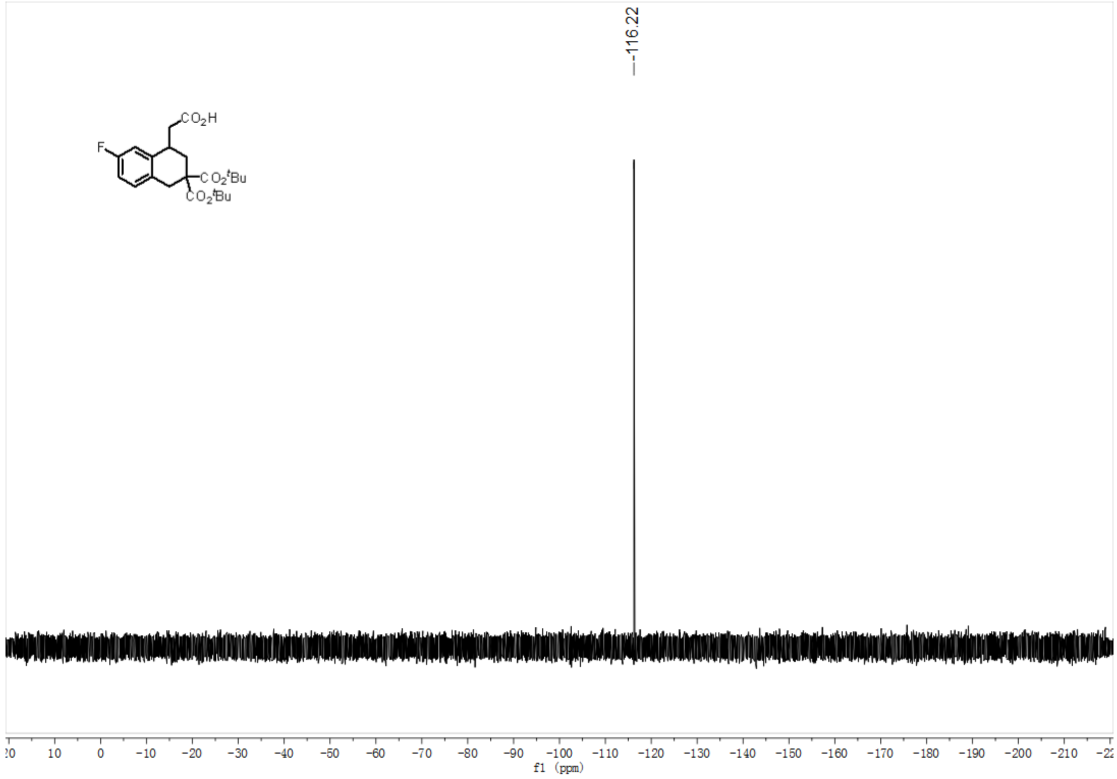

Supplementary Figure 34 ^{19}F NMR spectrum of **2g**

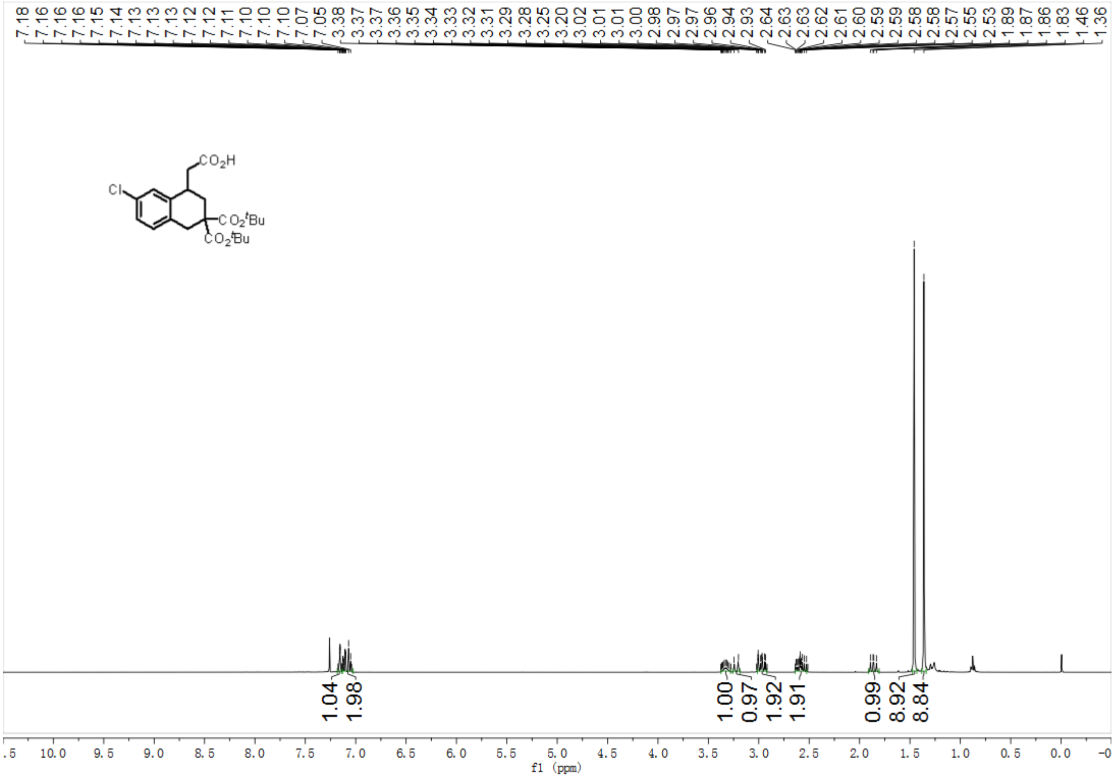

Supplementary Figure 35 ^{1}H NMR spectrum of **2h**

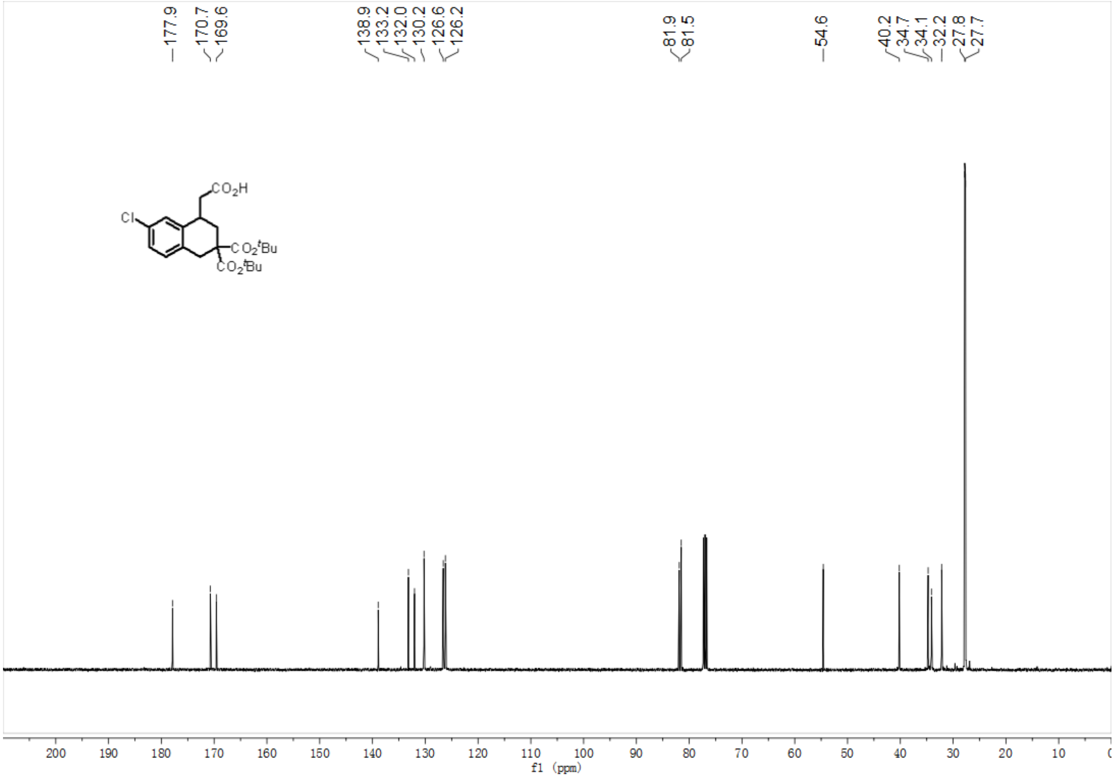

Supplementary Figure 36 ^{13}C NMR spectrum of **2h**

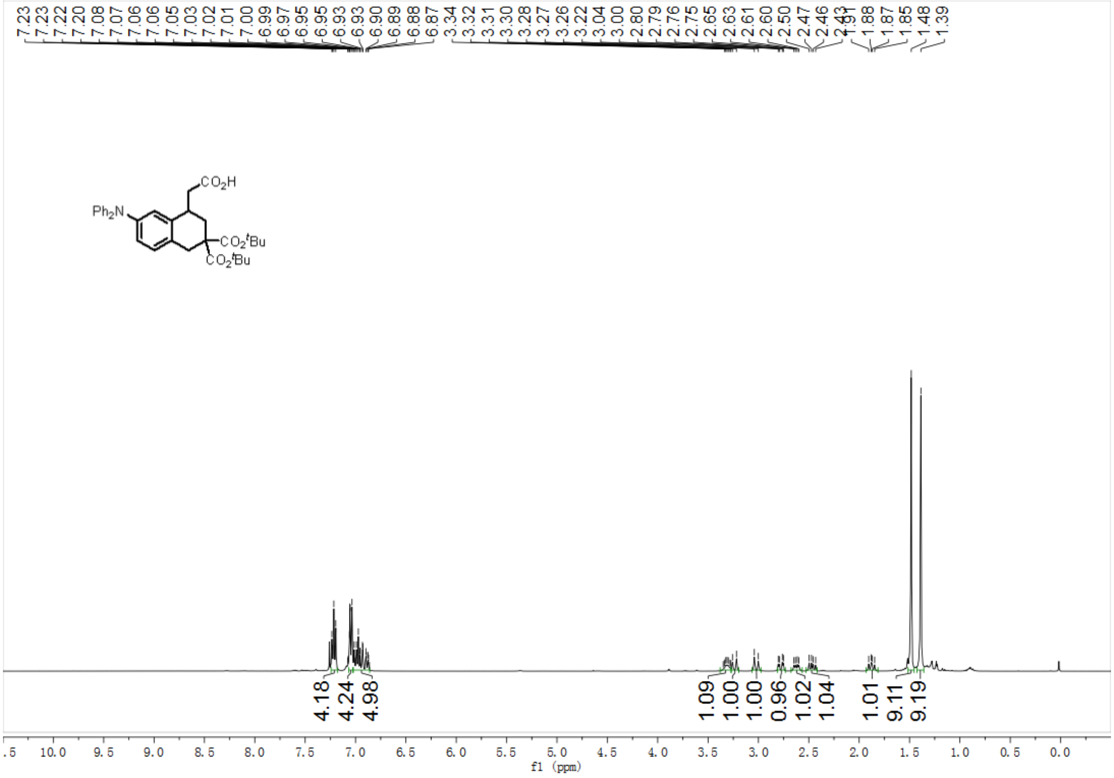

Supplementary Figure 37 ^{1}H NMR spectrum of **2i**

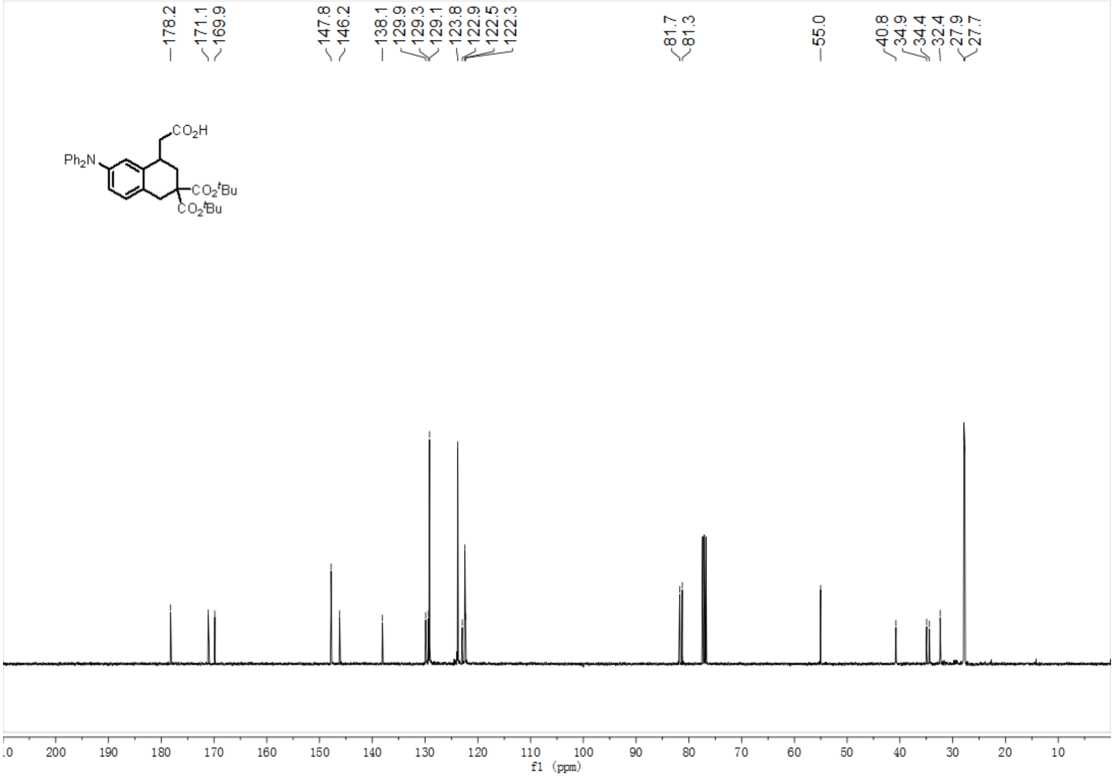

Supplementary Figure 38 ^{13}C NMR spectrum of **2i**

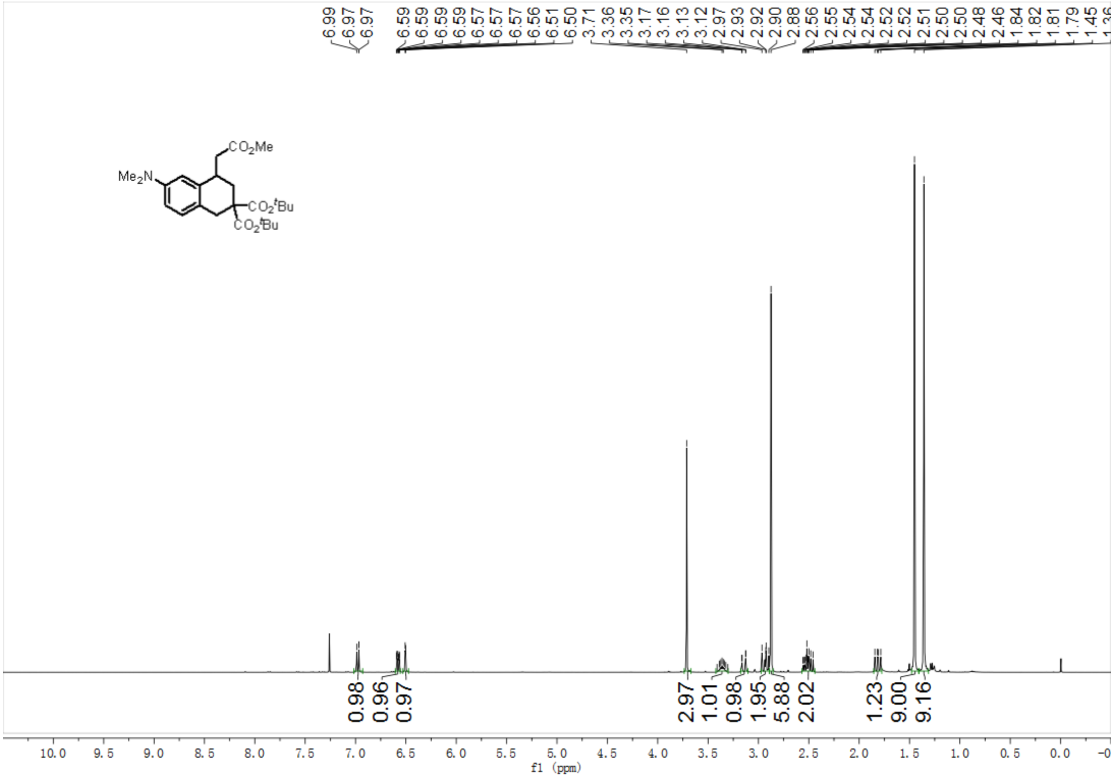

Supplementary Figure 39 ^{1}H NMR spectrum of **2j**

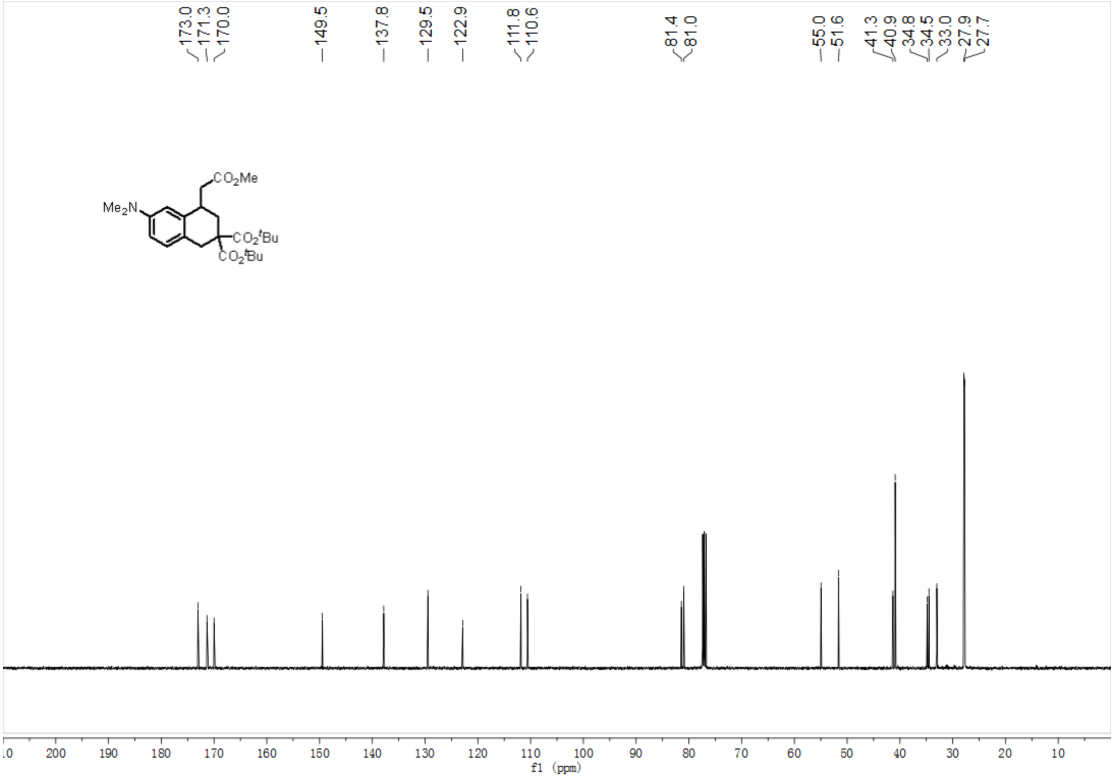

Supplementary Figure 40 ^{13}C NMR spectrum of **2j**

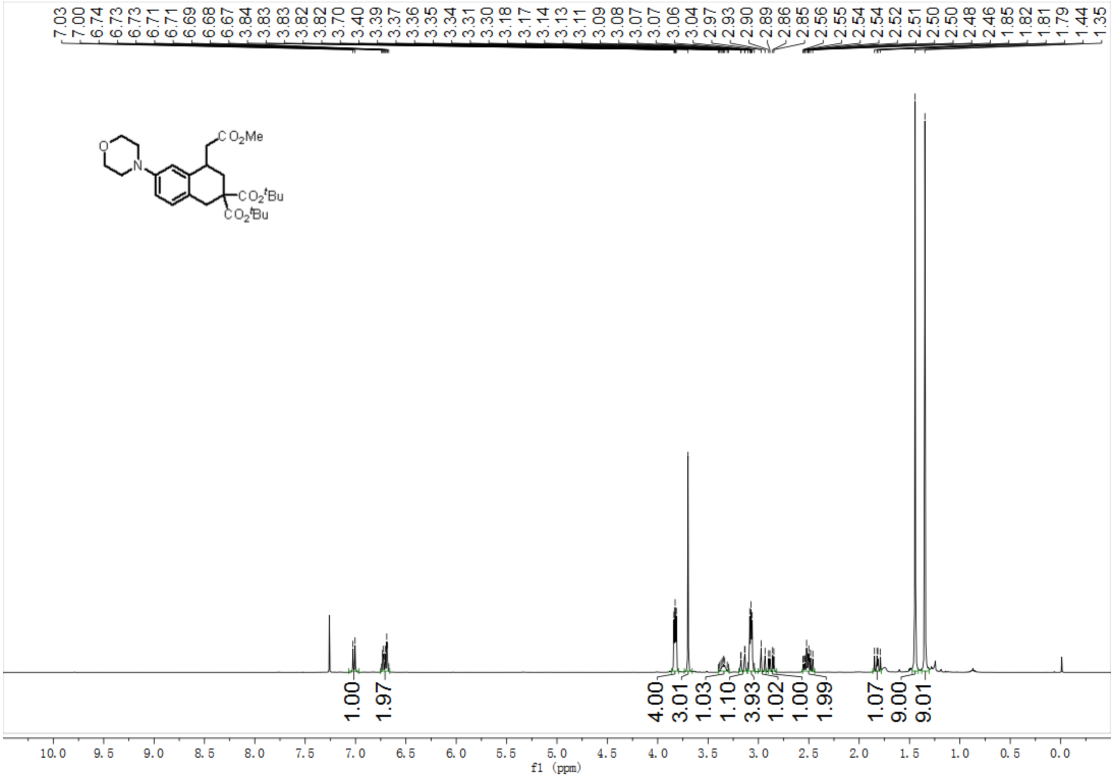

Supplementary Figure 41 ^{1}H NMR spectrum of **2k**

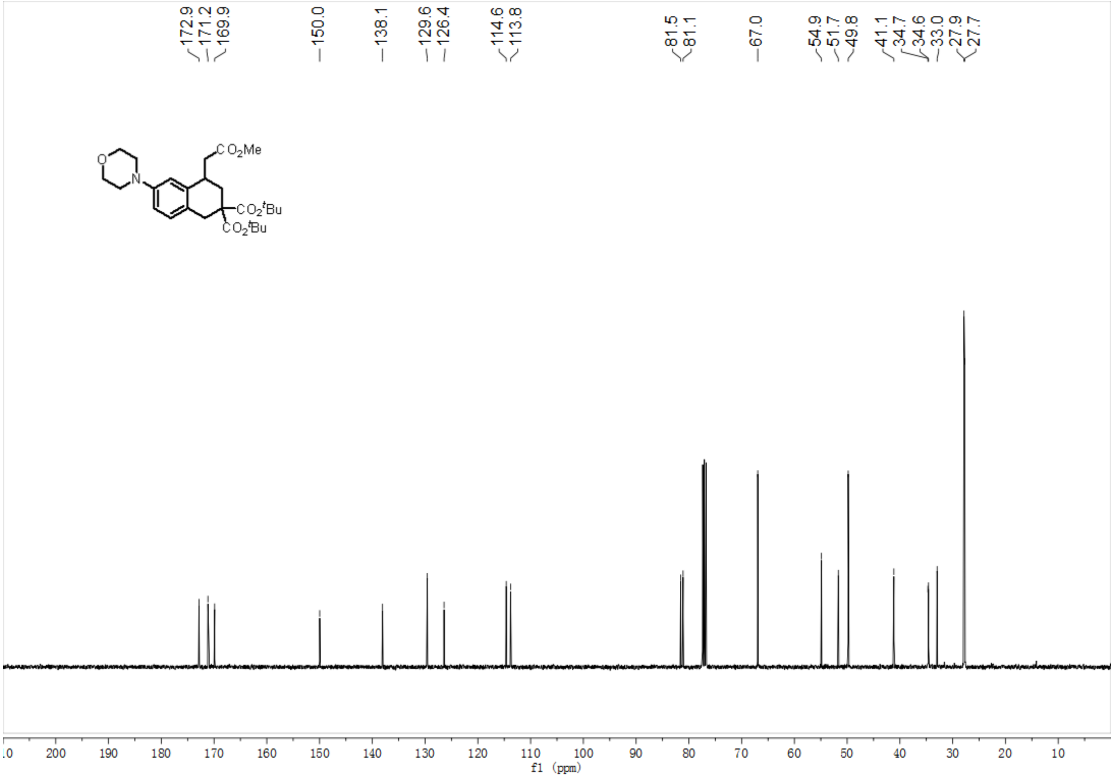

Supplementary Figure 42 ^{13}C NMR spectrum of **2k**

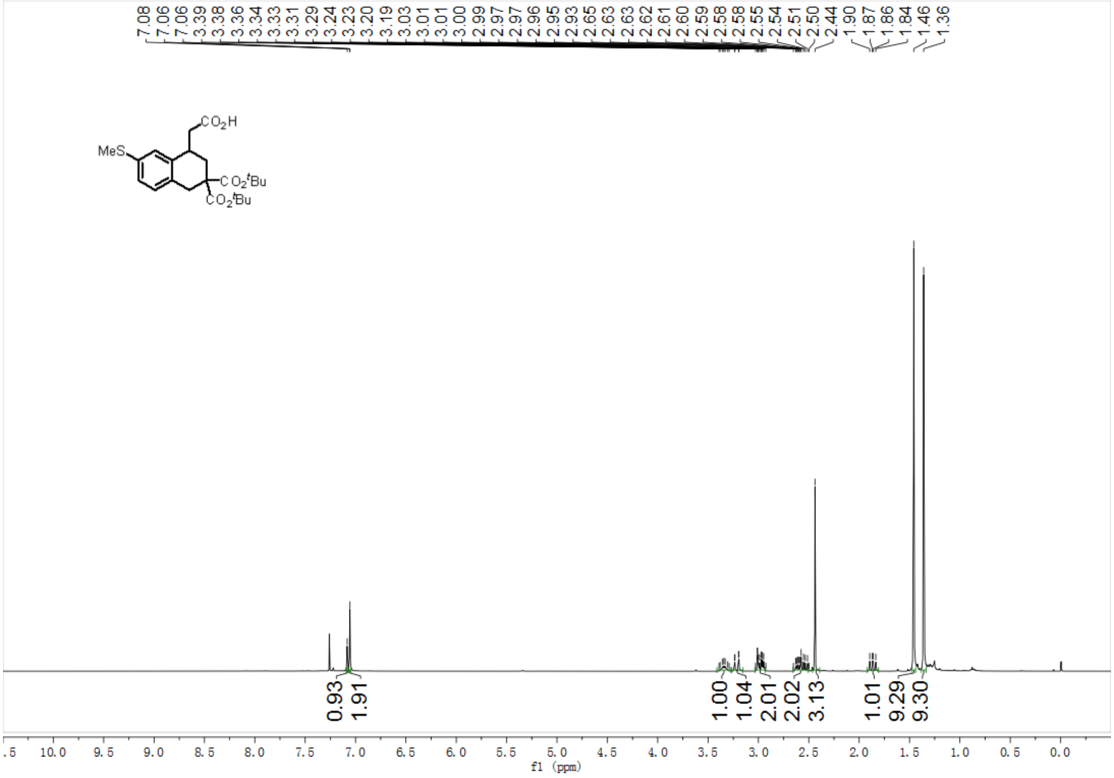

Supplementary Figure 43 ^{1}H NMR spectrum of **2l**

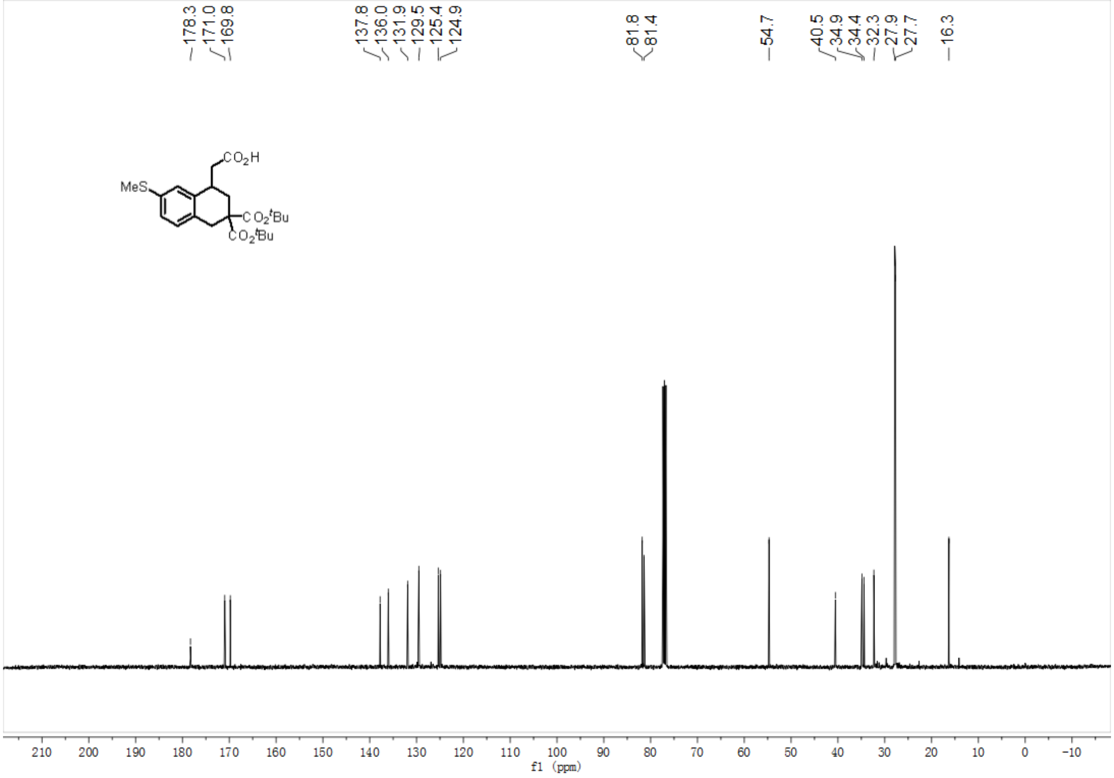

Supplementary Figure 44 ^{13}C NMR spectrum of **2l**

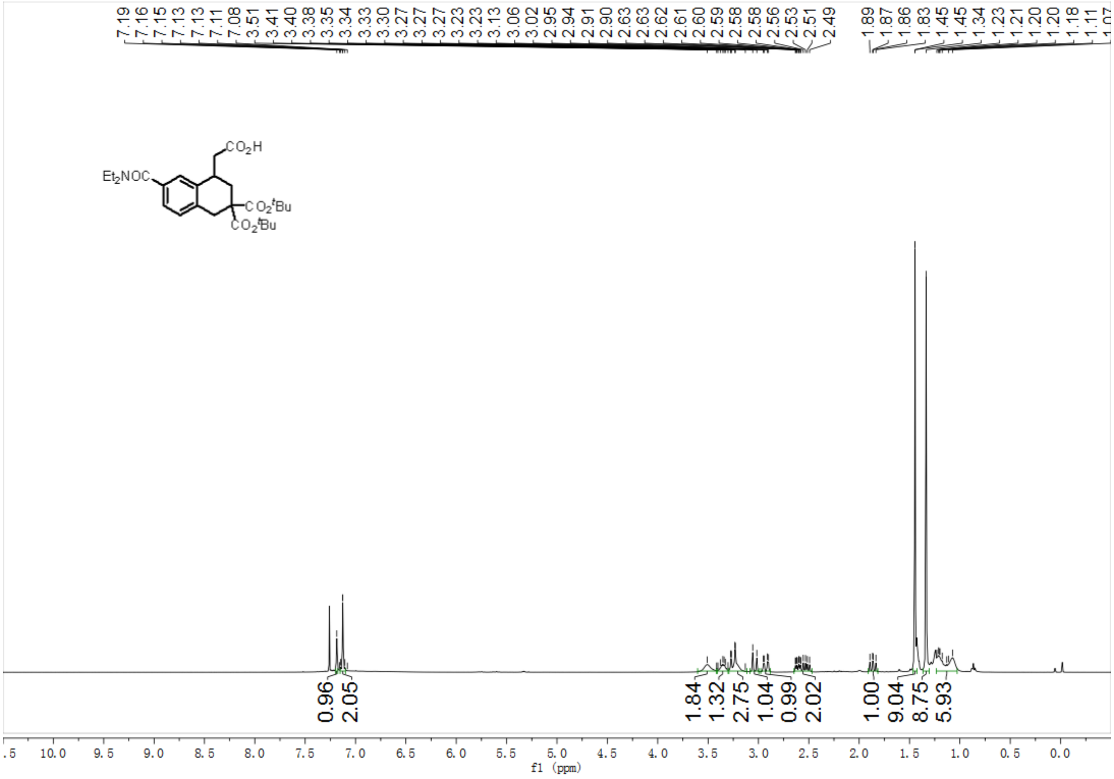

Supplementary Figure 45 ^{1}H NMR spectrum of **2m**

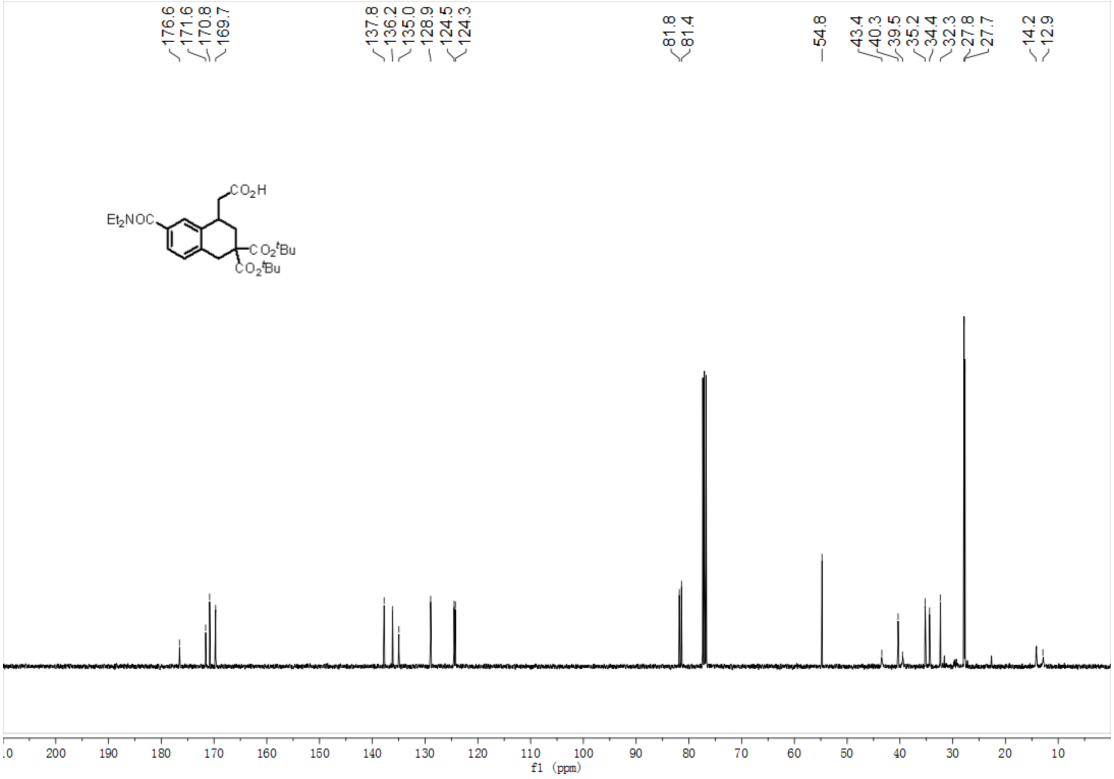

Supplementary Figure 46 ^{13}C NMR spectrum of **2m**

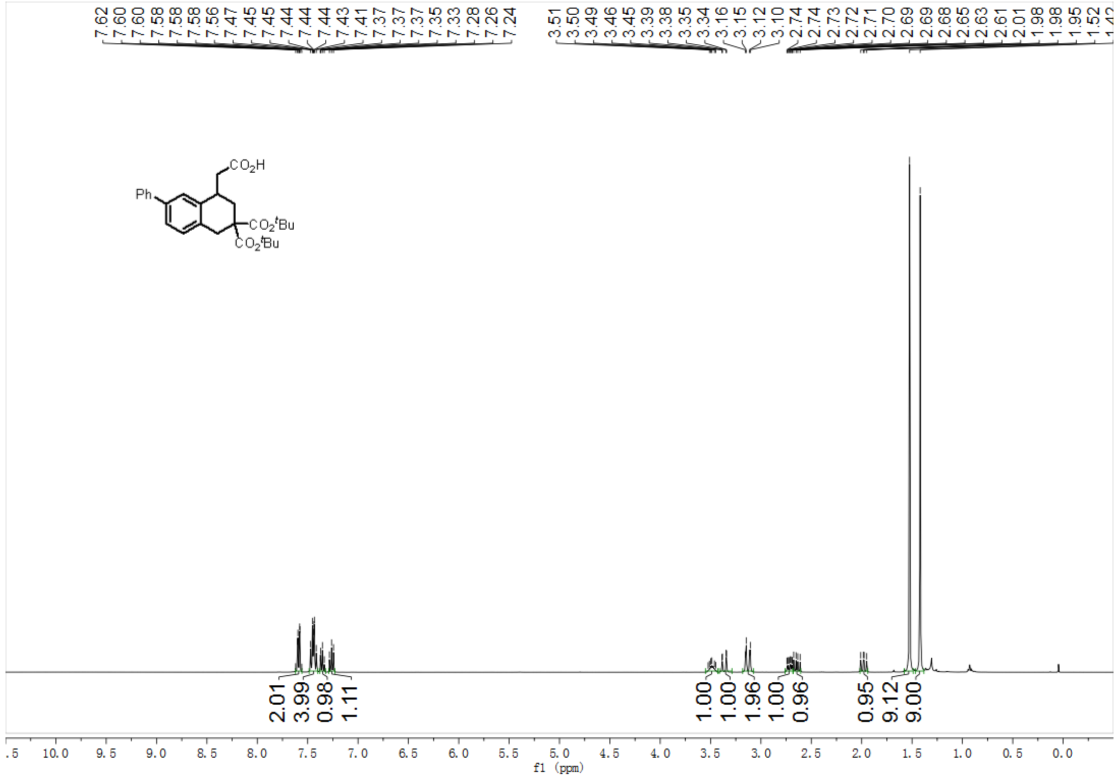

Supplementary Figure 47 ^{1}H NMR spectrum of **2n**

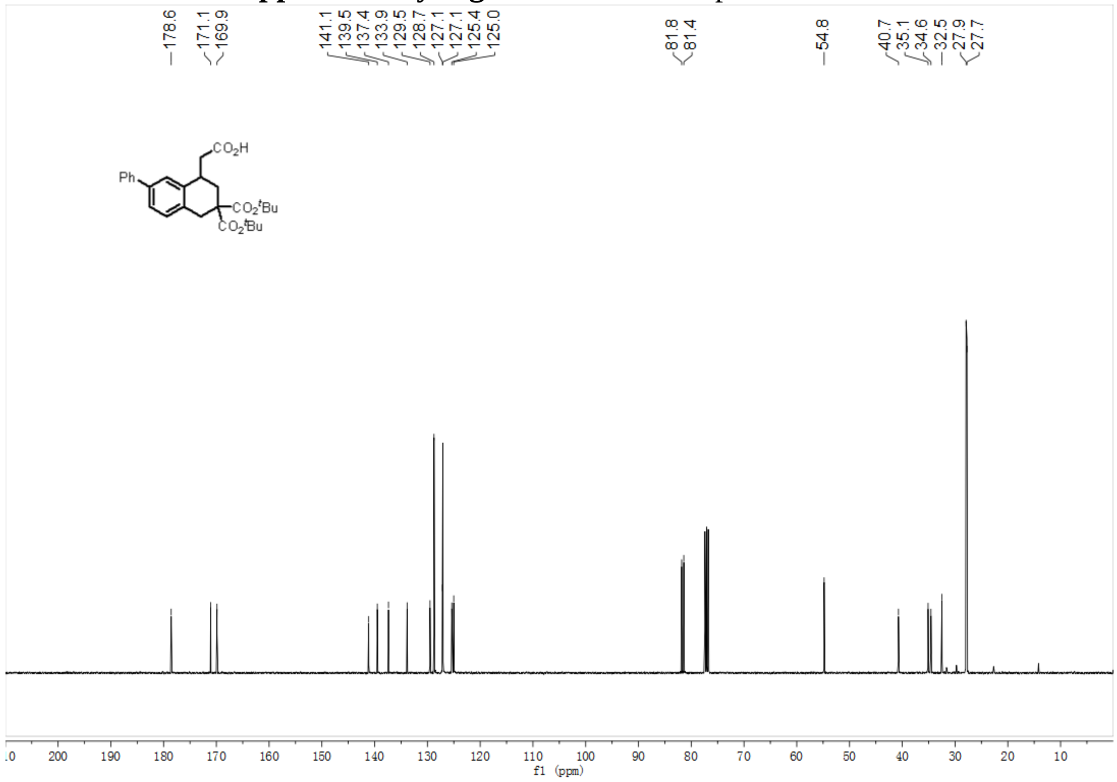

Supplementary Figure 48 ^{13}C NMR spectrum of **2n**

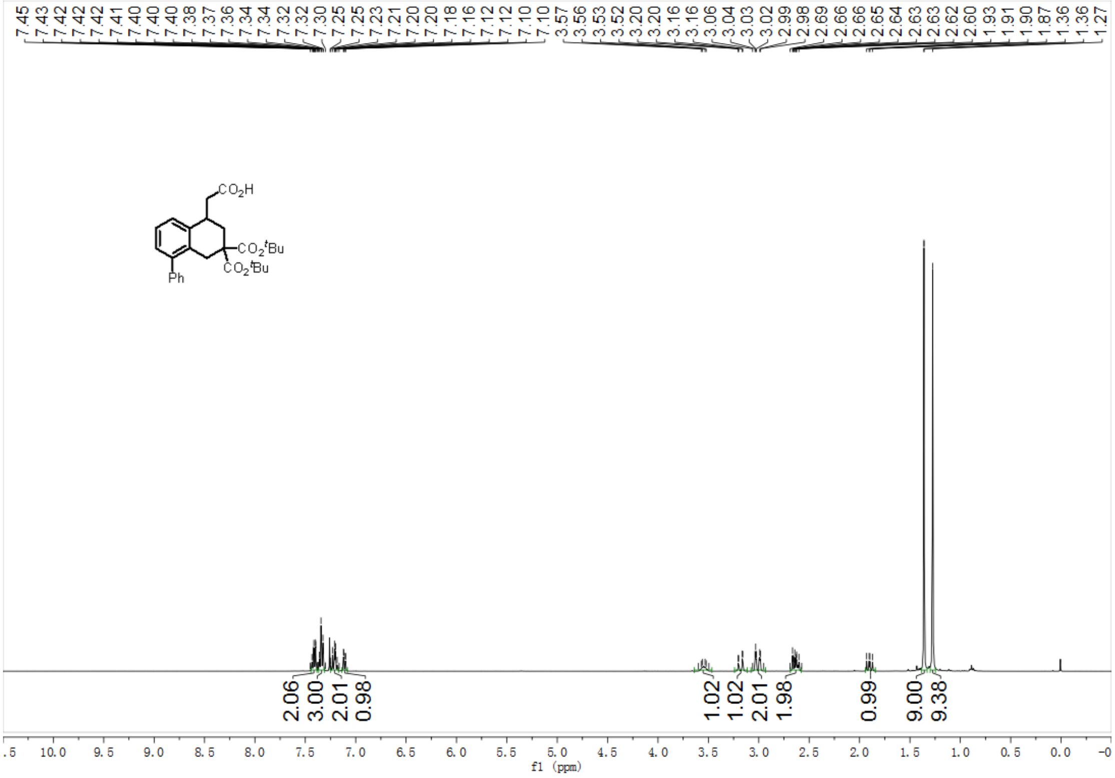

Supplementary Figure 49 ^{1}H NMR spectrum of **2o**

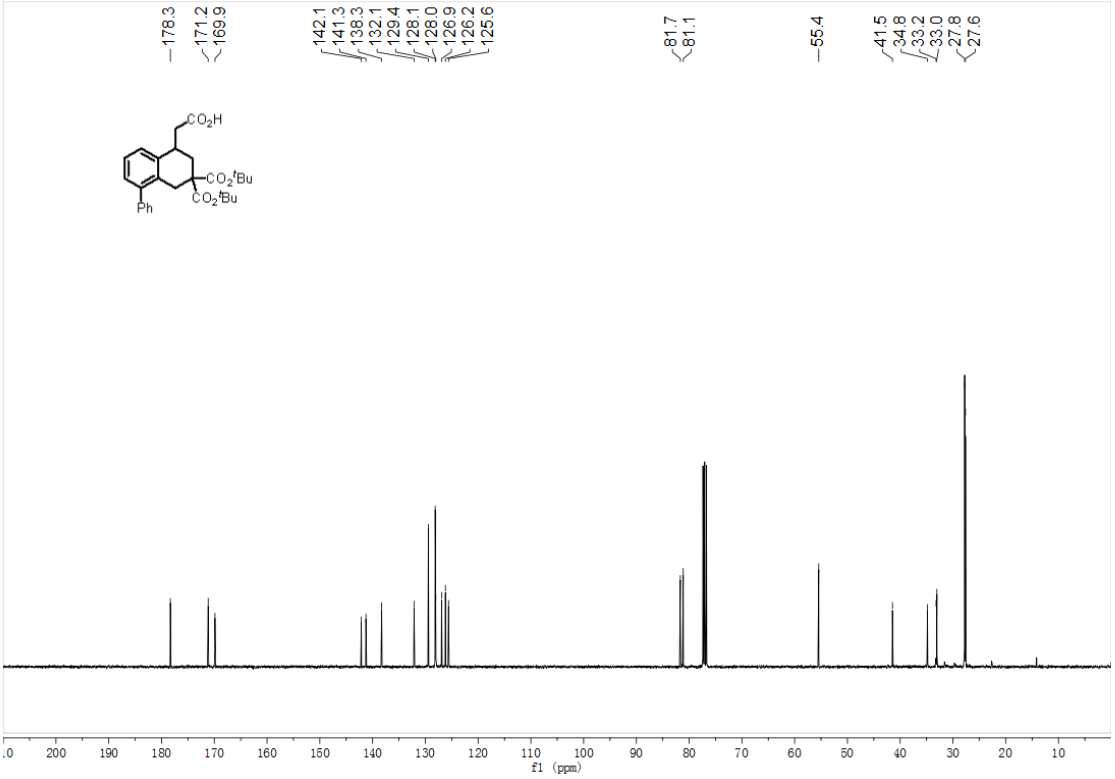

Supplementary Figure 50 ^{13}C NMR spectrum of **2o**

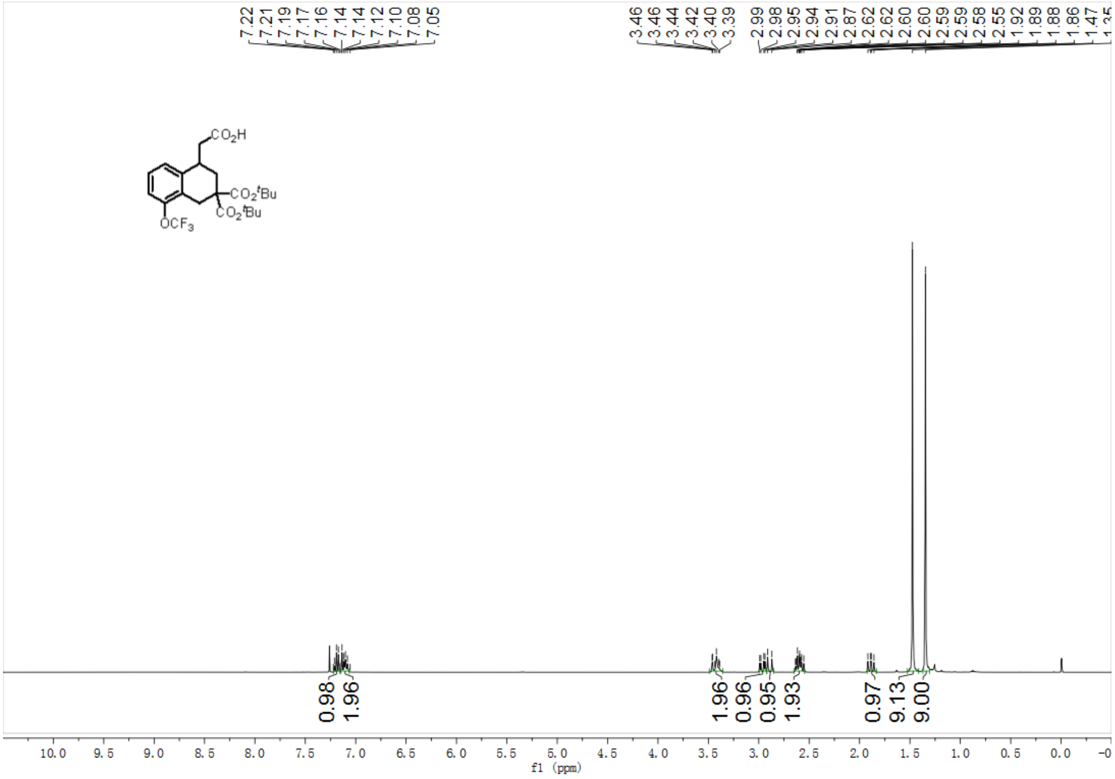

Supplementary Figure 51 ^{1}H NMR spectrum of **2p**

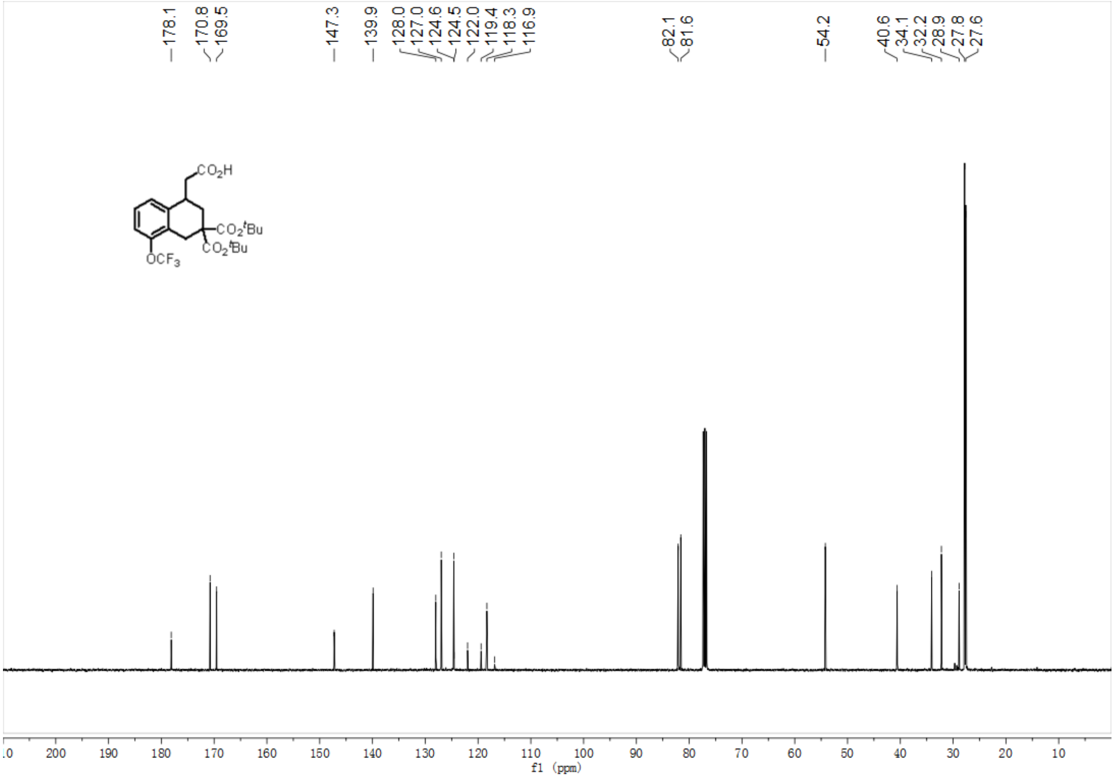

Supplementary Figure 52 ^{13}C NMR spectrum of **2p**

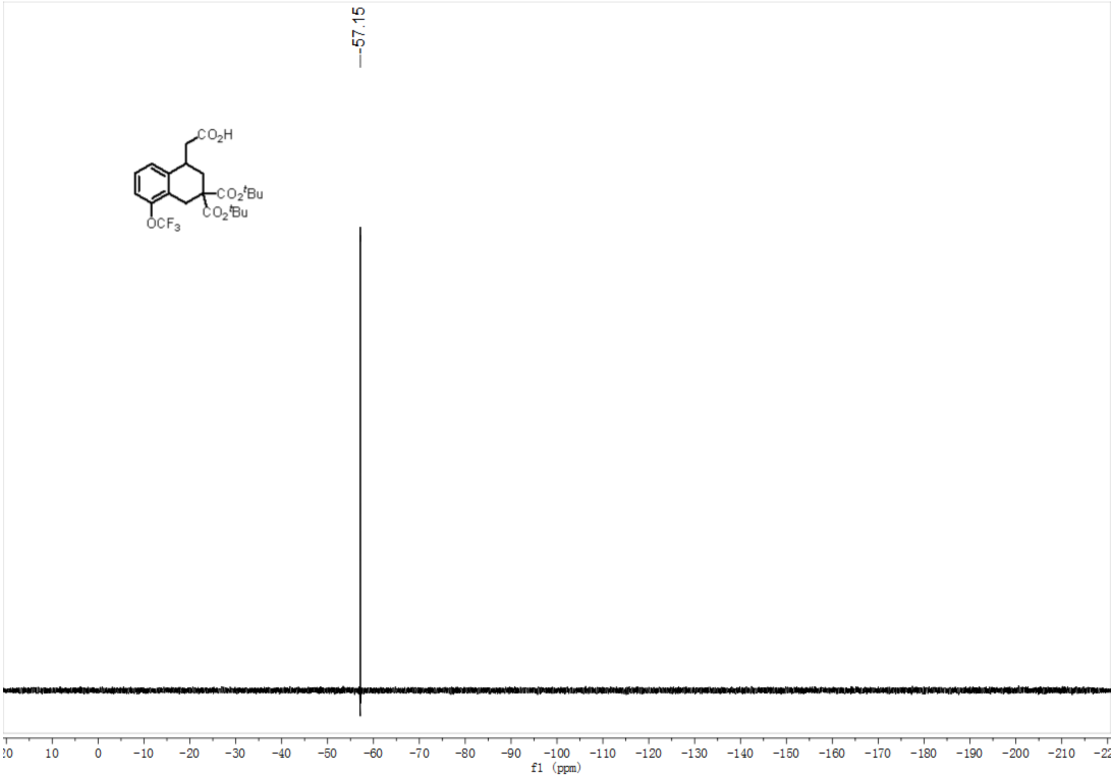

Supplementary Figure 53 ^{19}F NMR spectrum of **2p**

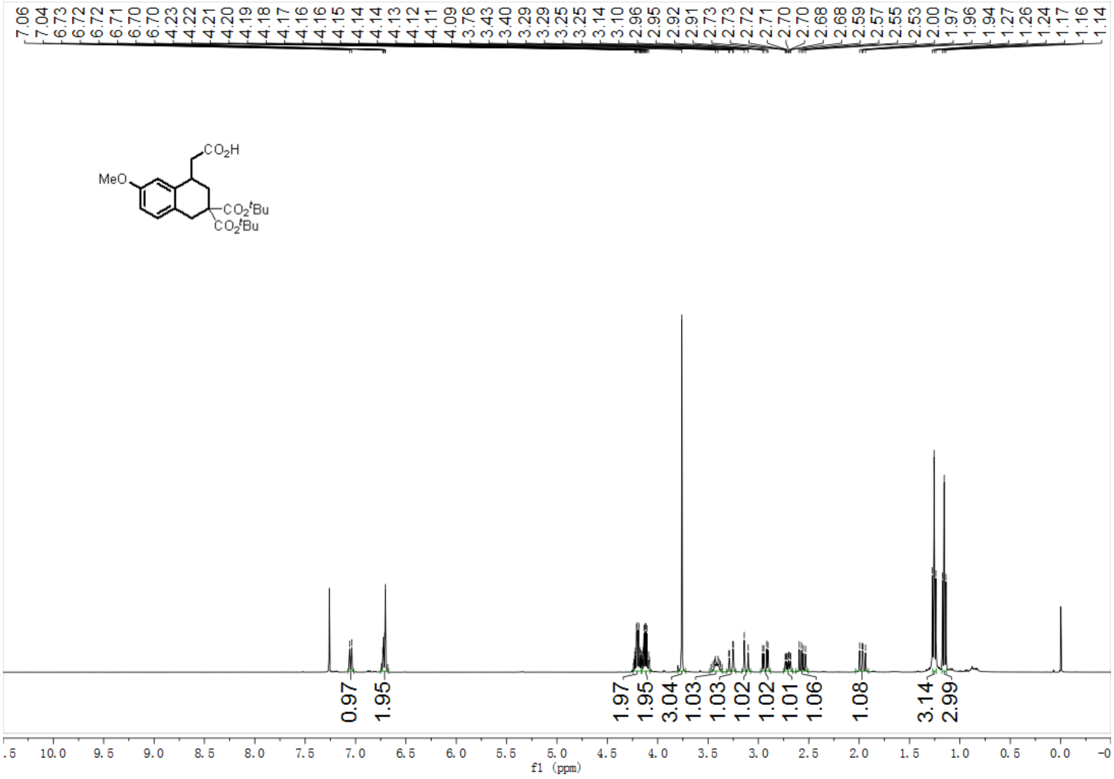

Supplementary Figure 54 ^{1}H NMR spectrum of **2q**

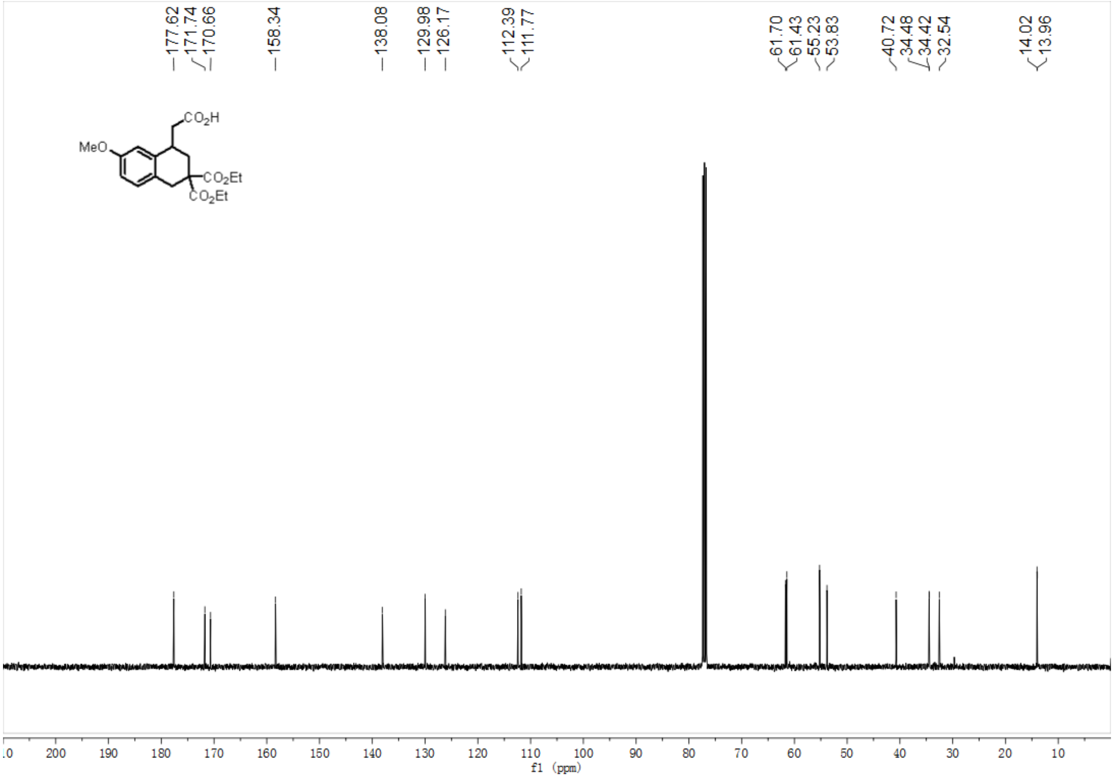

Supplementary Figure 55 ^{13}C NMR spectrum of **2q**

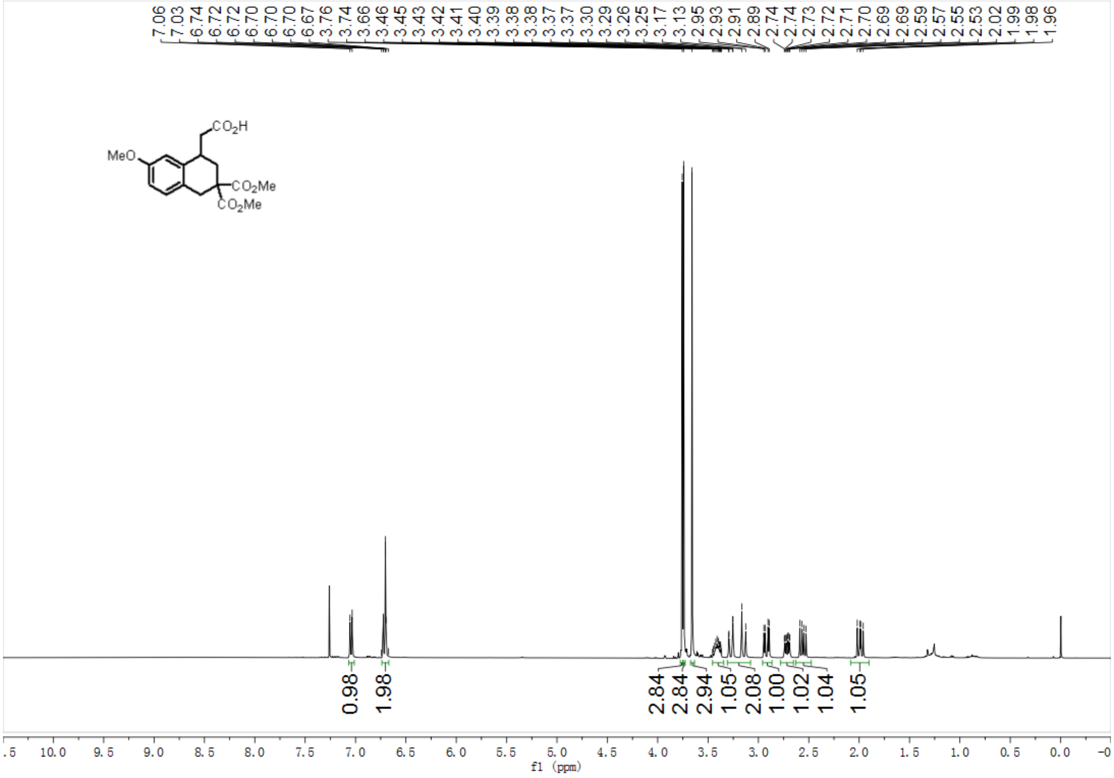

Supplementary Figure 56 ^{1}H NMR spectrum of **2r**

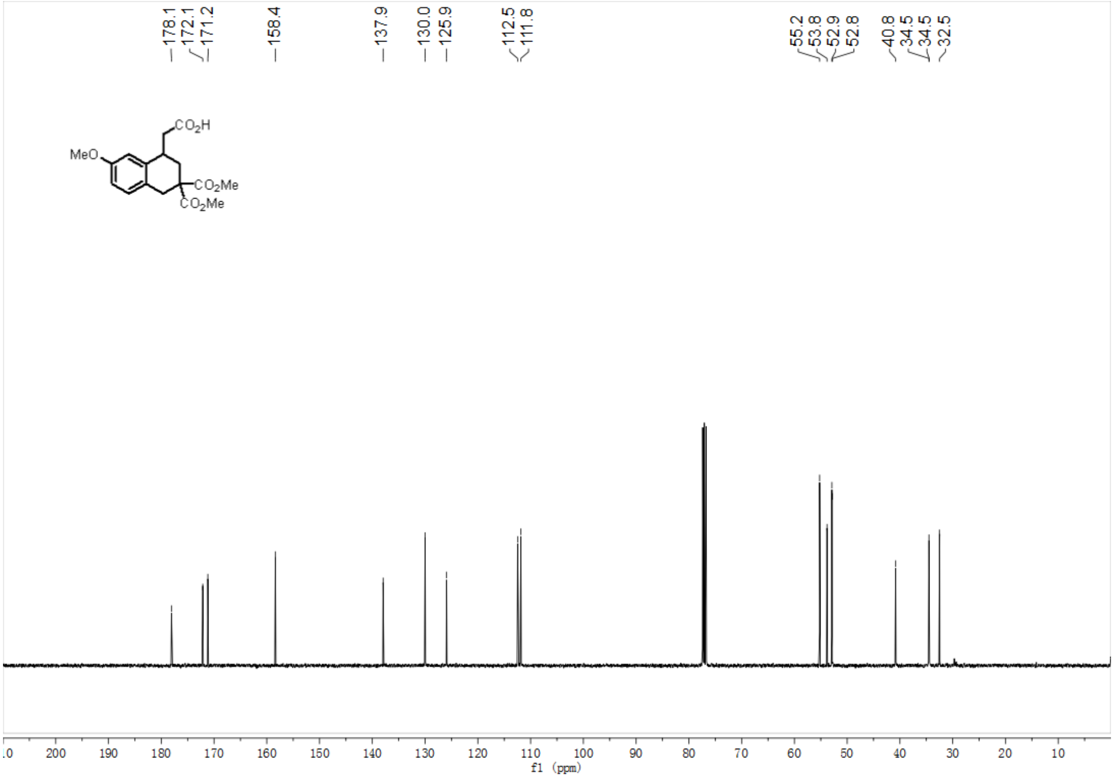

Supplementary Figure 57 ^{13}C NMR spectrum of **2r**

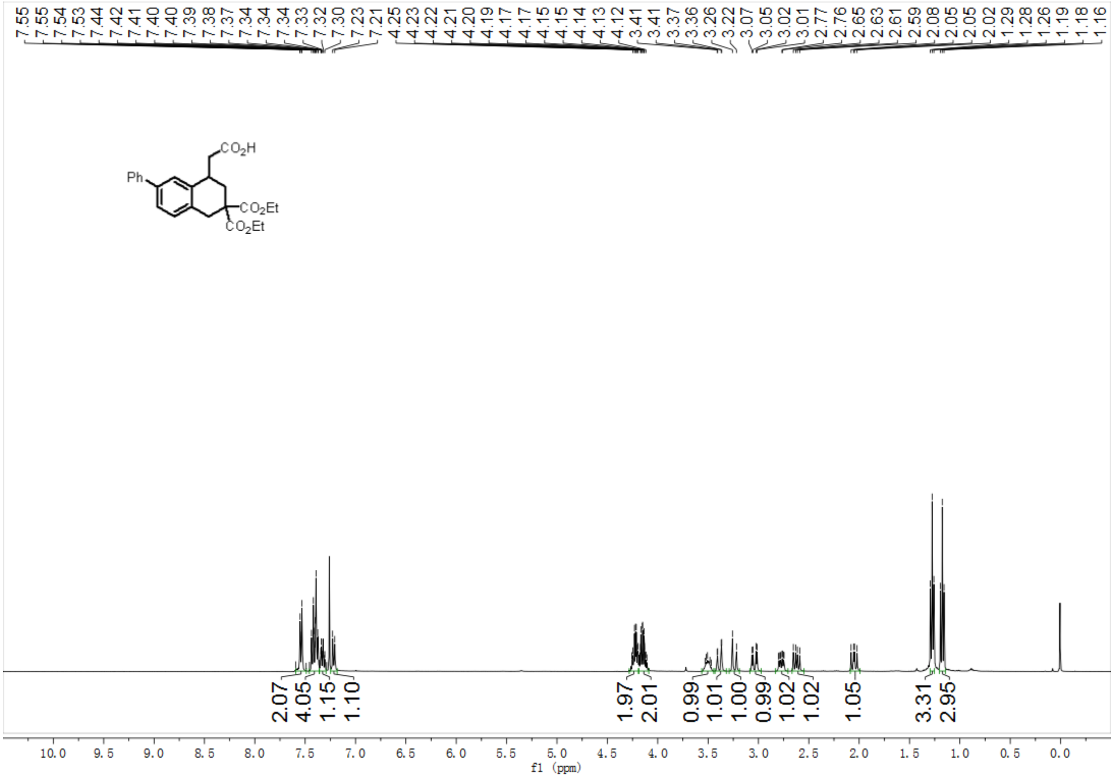

Supplementary Figure 58 ^{1}H NMR spectrum of **2s**

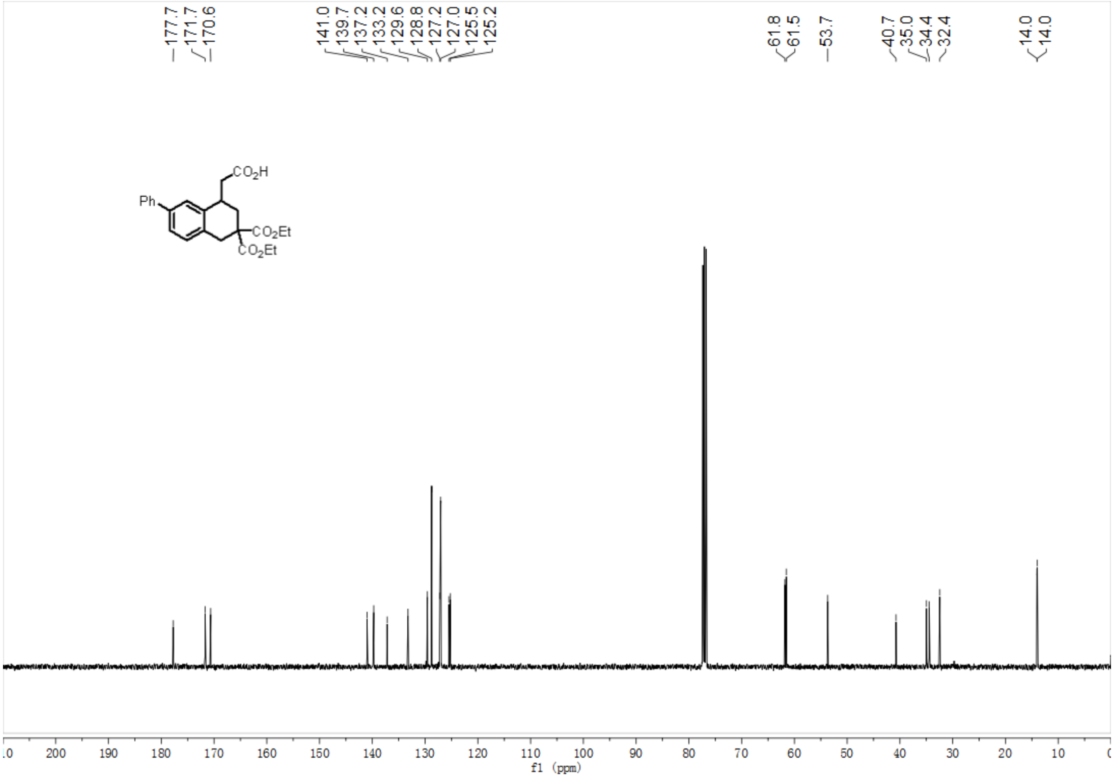

Supplementary Figure 59 ^{13}C NMR spectrum of **2s**

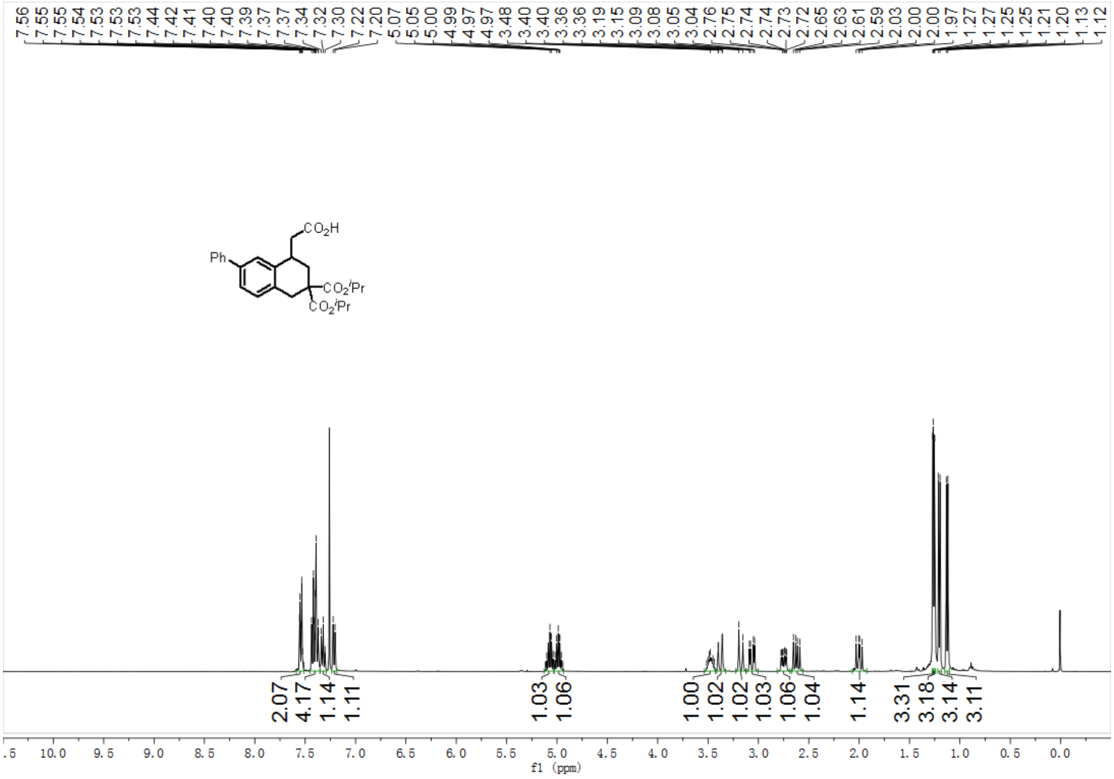

Supplementary Figure 60 ^{1}H NMR spectrum of **2t**

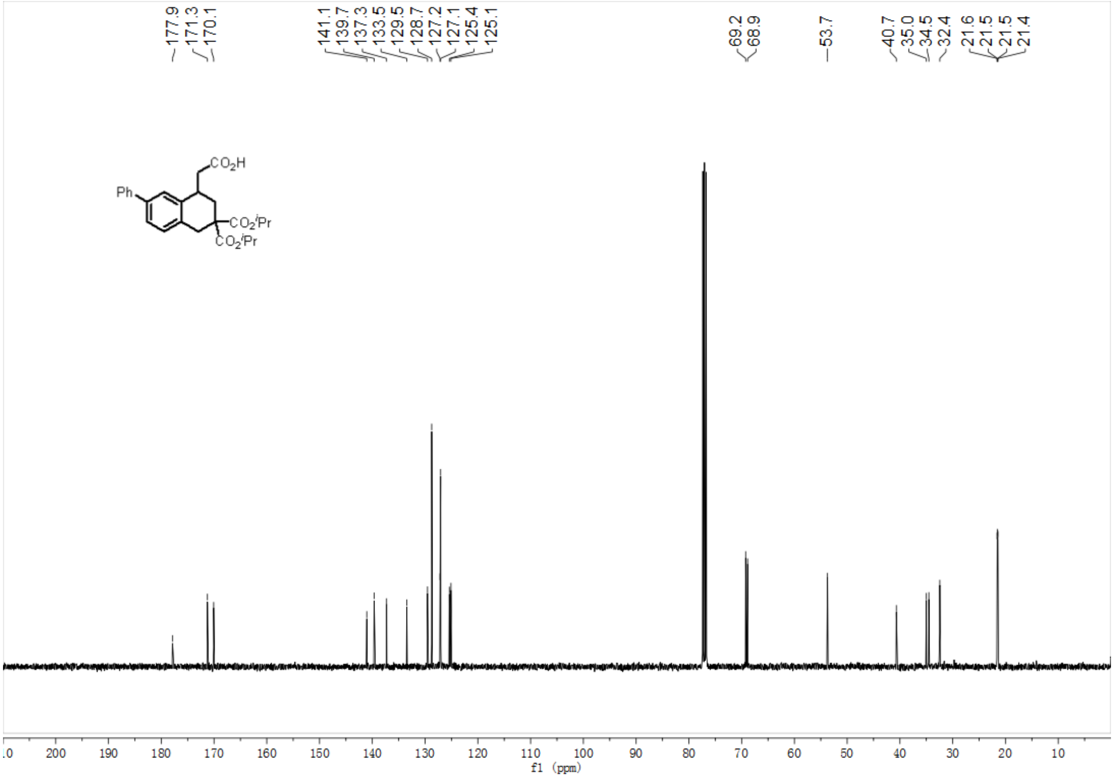

Supplementary Figure 61 ^{13}C NMR spectrum of **2t**

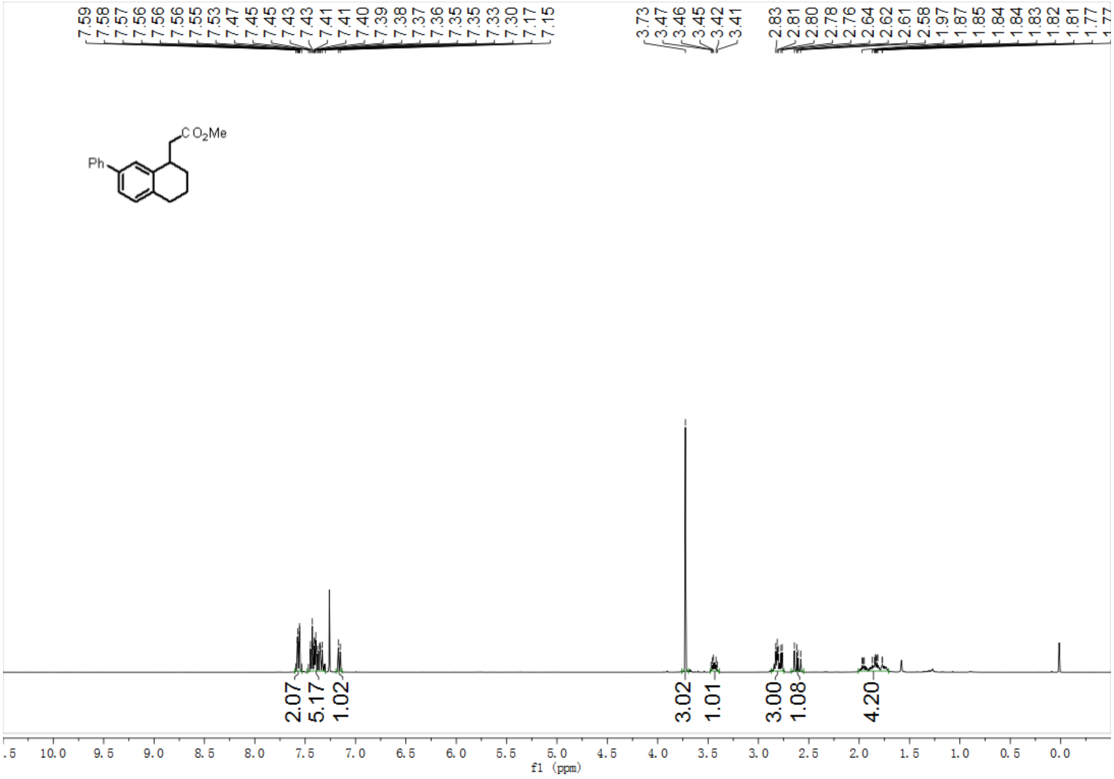

Supplementary Figure 62 ^{1}H NMR spectrum of **2u**

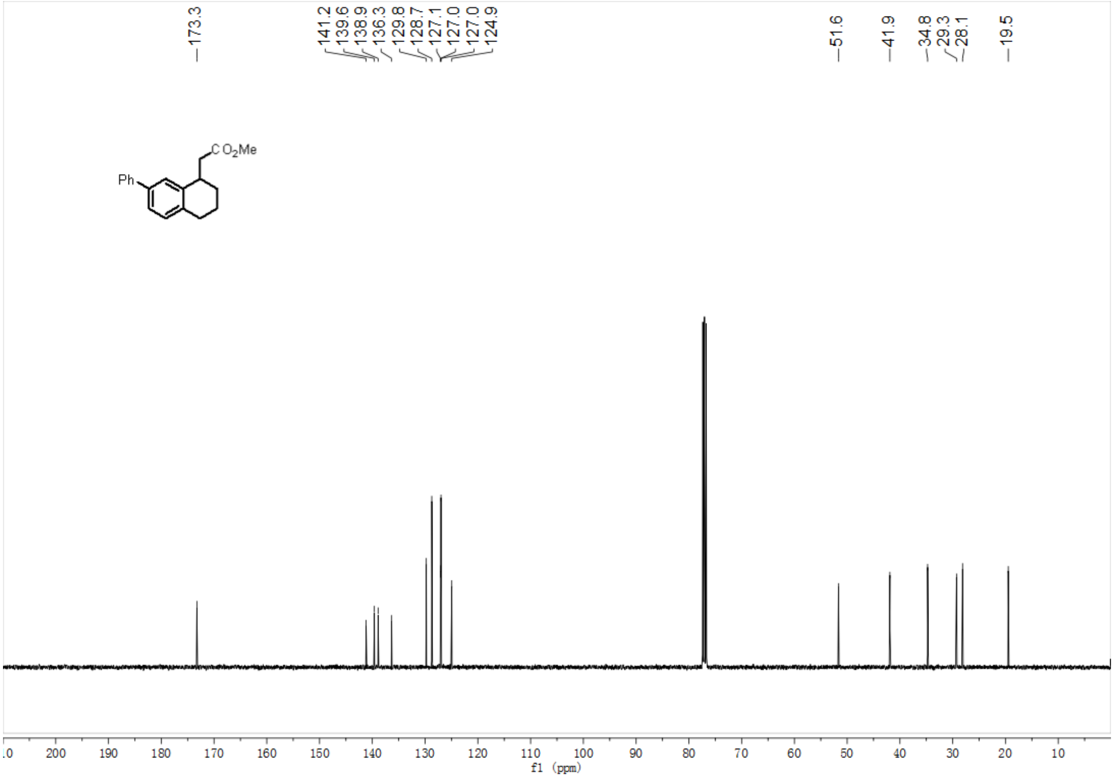

Supplementary Figure 63 ^{13}C NMR spectrum of **2u**

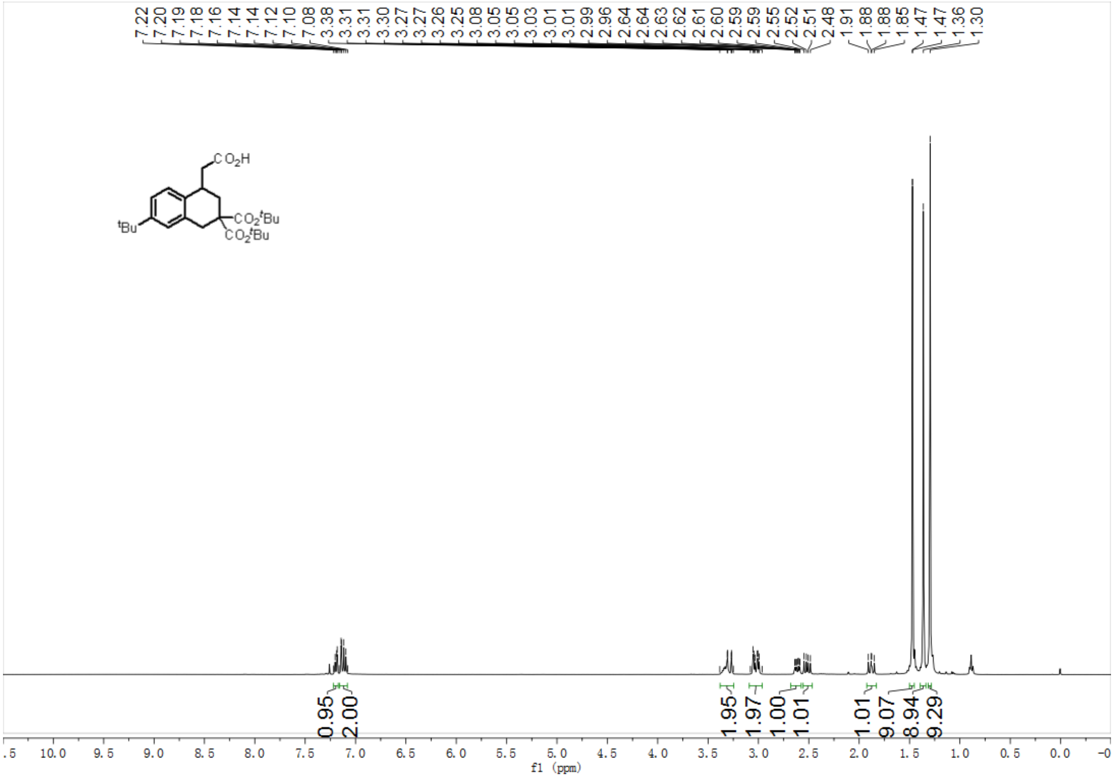

Supplementary Figure 64 ^{1}H NMR spectrum of **2v**

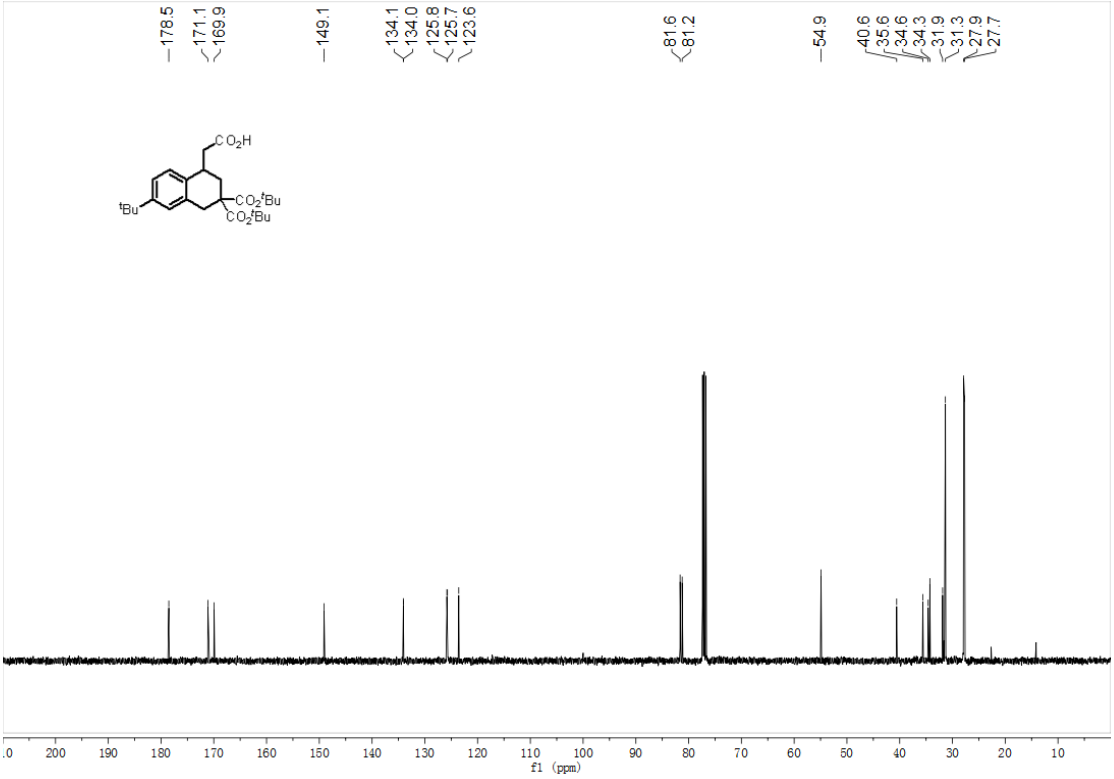

Supplementary Figure 65 ^{13}C NMR spectrum of **2v**

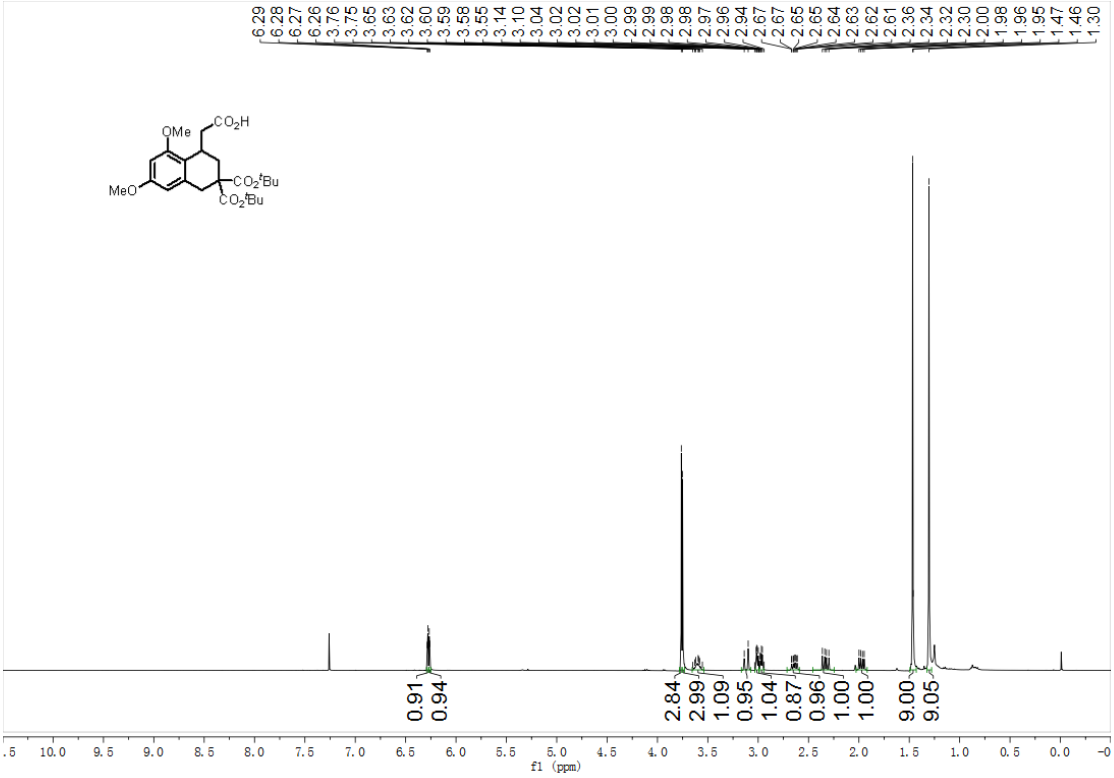

Supplementary Figure 66 ^{1}H NMR spectrum of **2w**

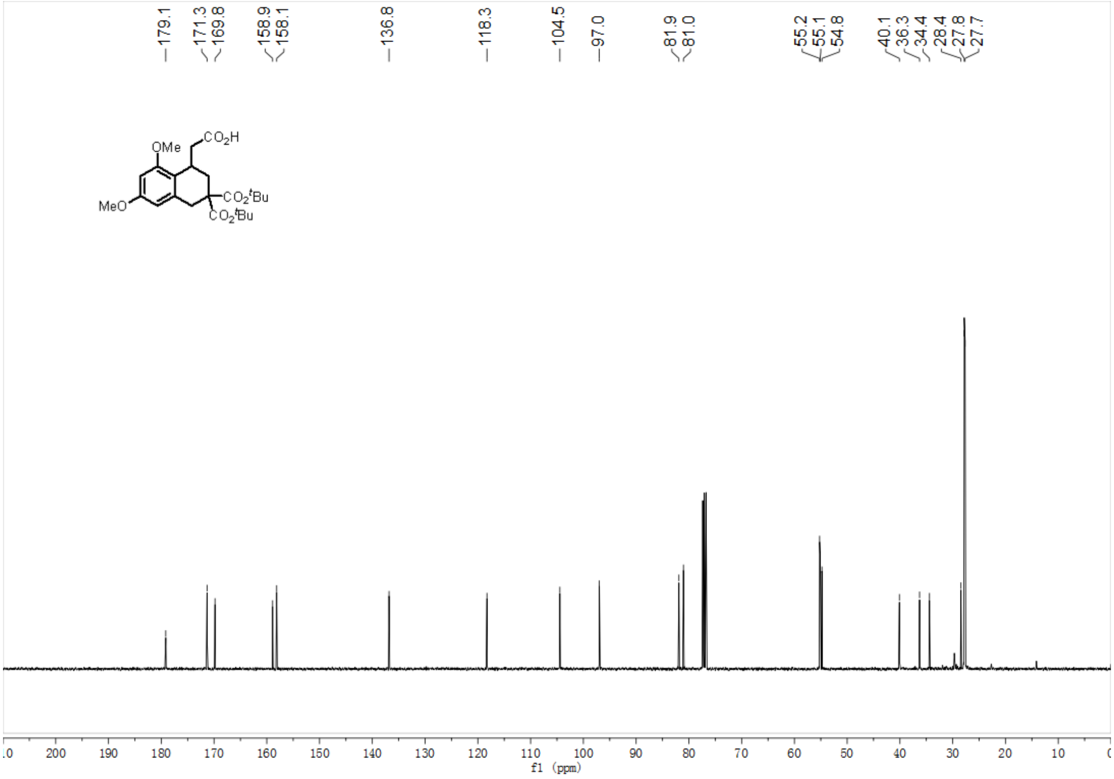

Supplementary Figure 67 ^{13}C NMR spectrum of **2w**

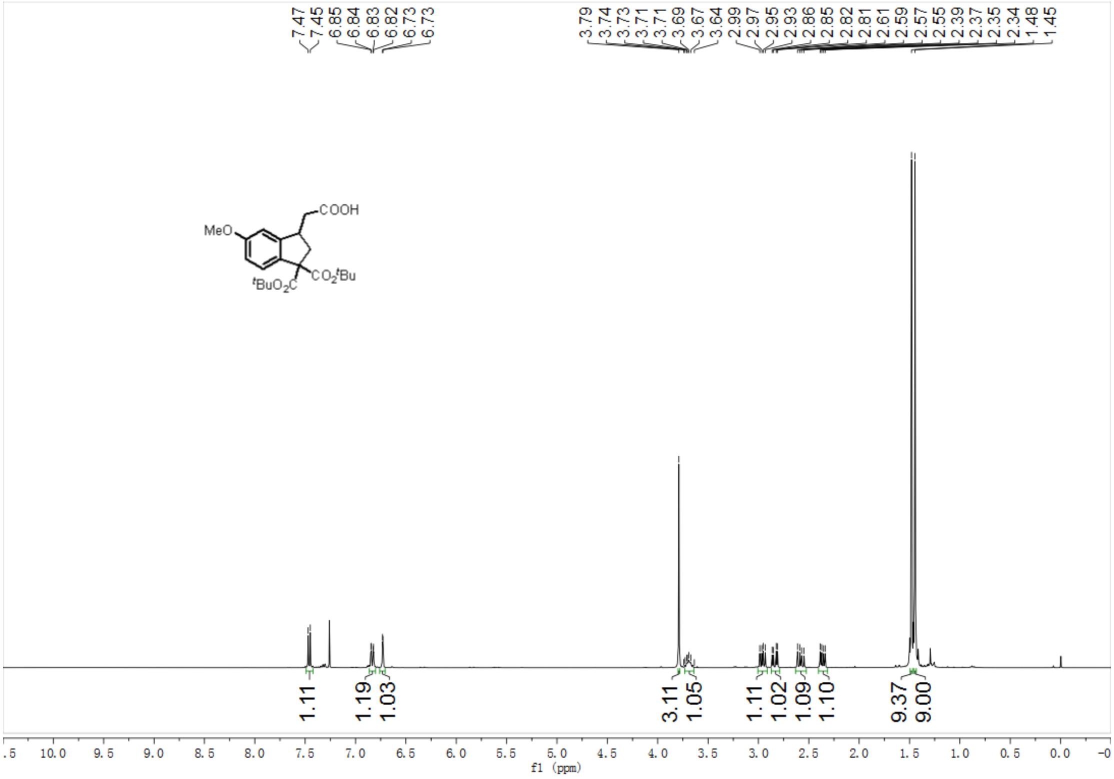

Supplementary Figure 68 ^{1}H NMR spectrum of **2x**

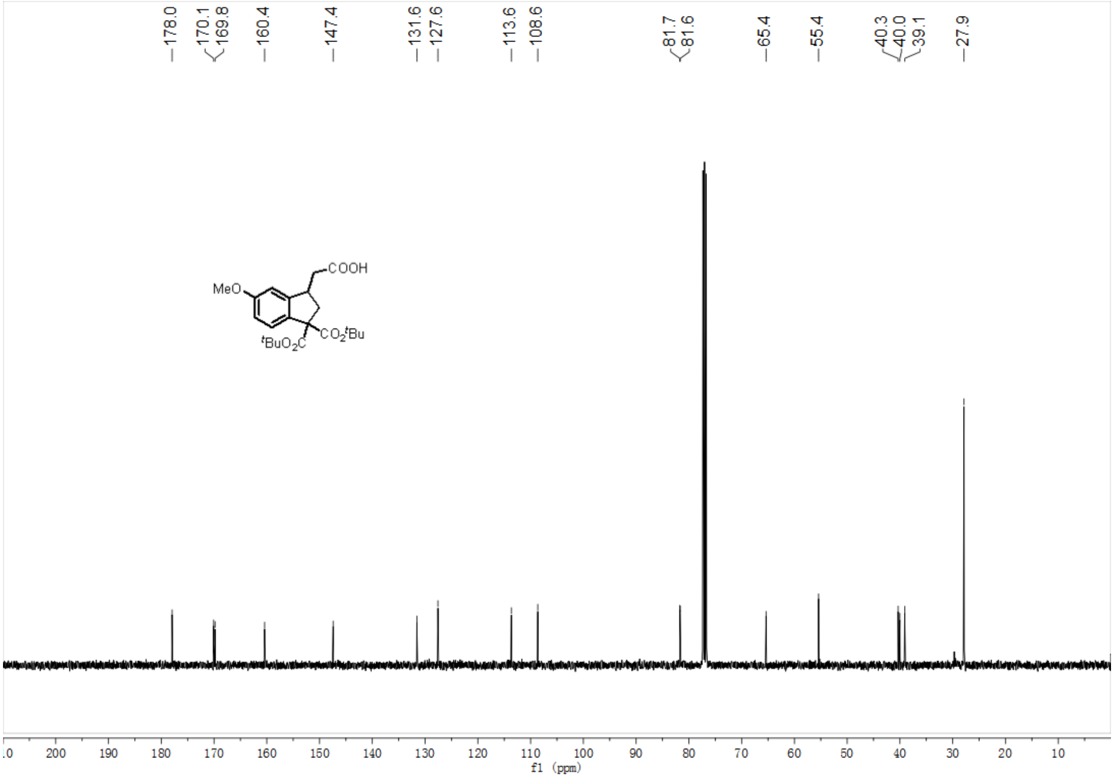

Supplementary Figure 69 ^{13}C NMR spectrum of **2x**

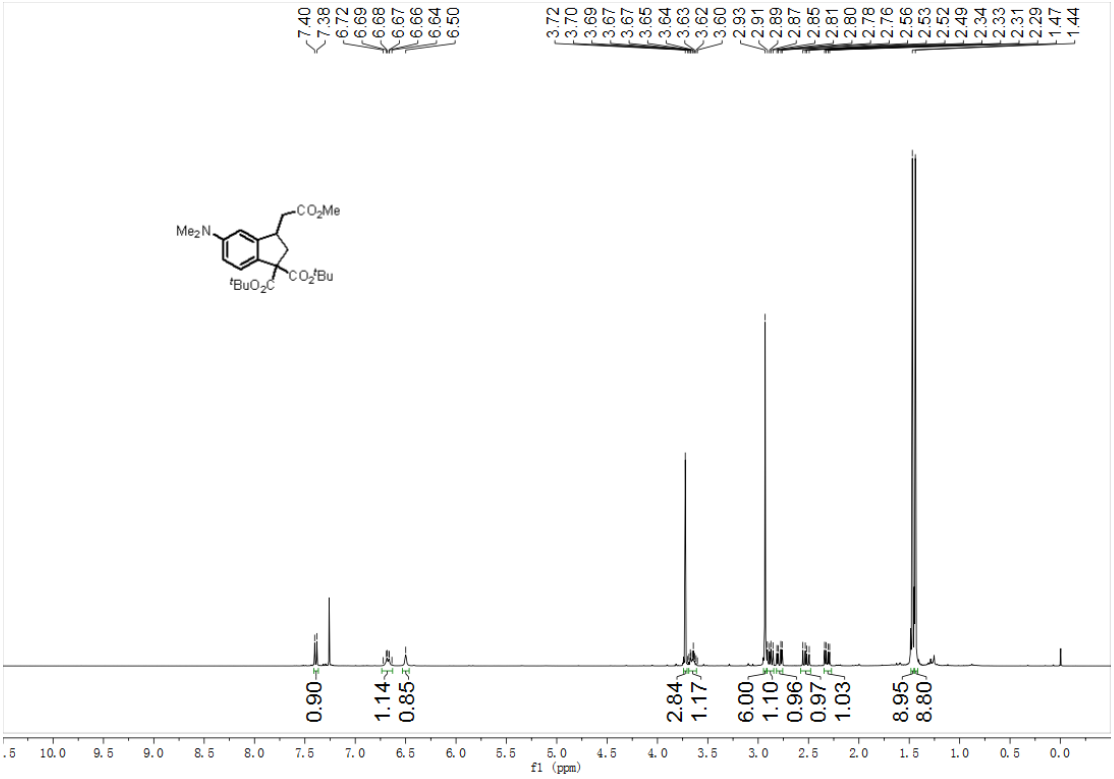

Supplementary Figure 70 ^{1}H NMR spectrum of **2y**

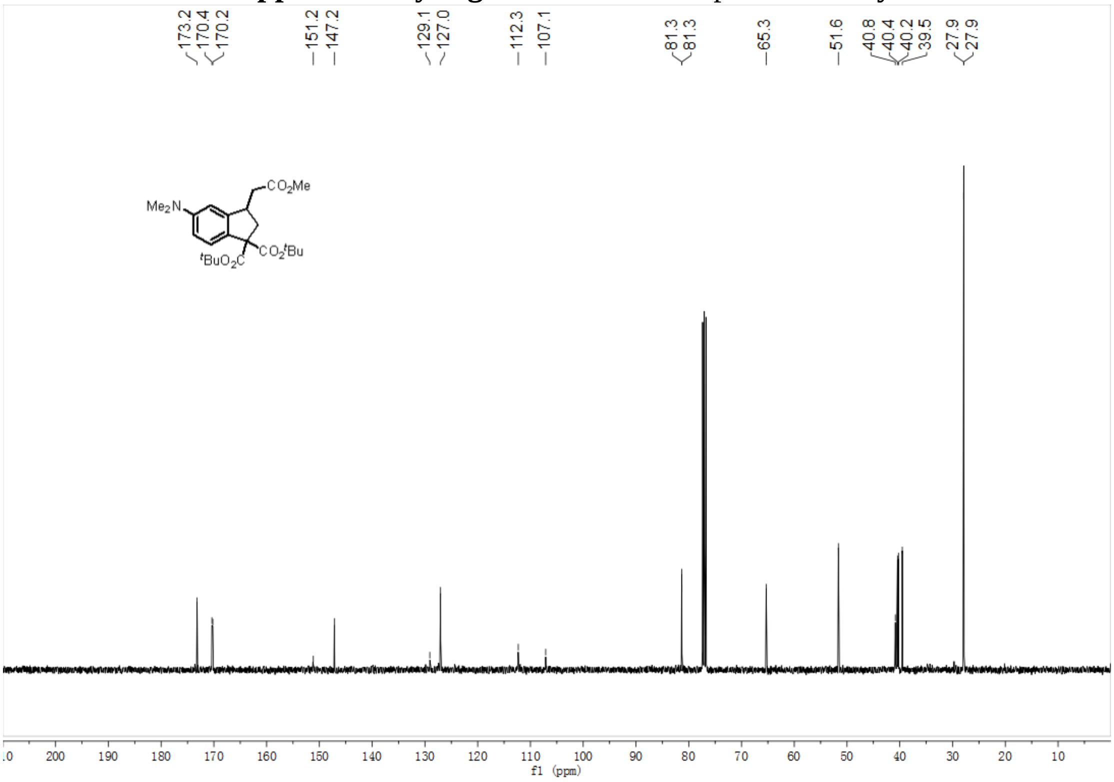

Supplementary Figure 71 ^{13}C NMR spectrum of **2y**

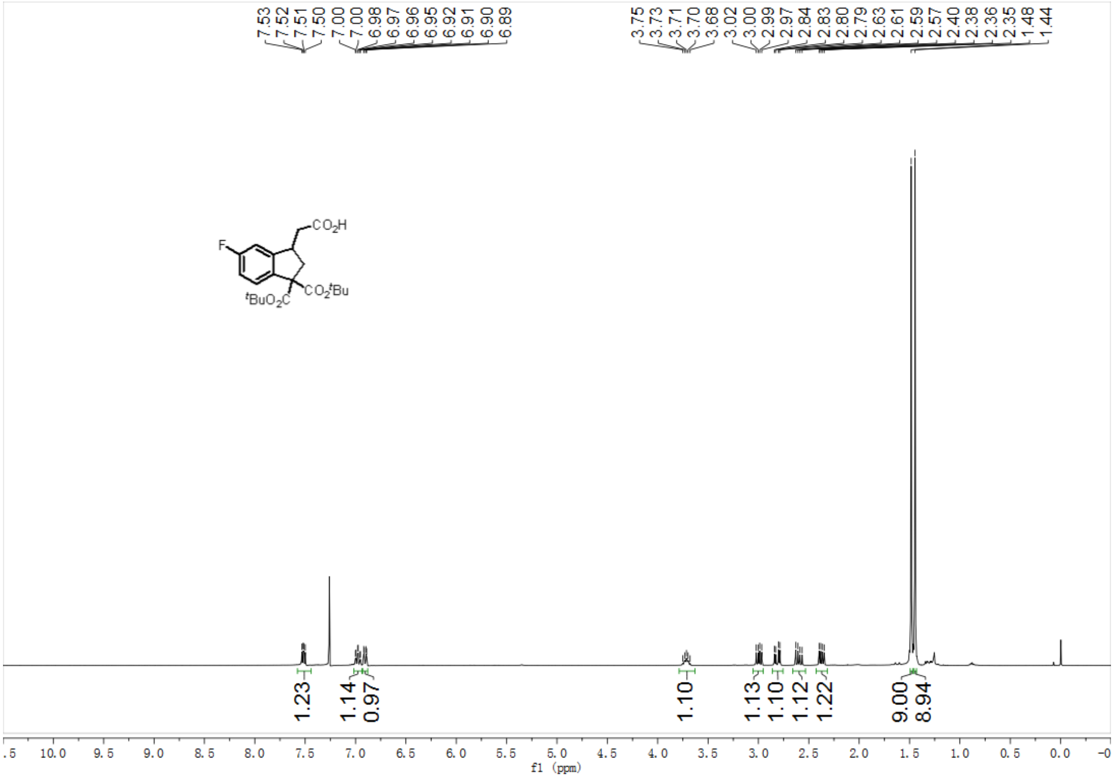

Supplementary Figure 72 ^{1}H NMR spectrum of **2z**

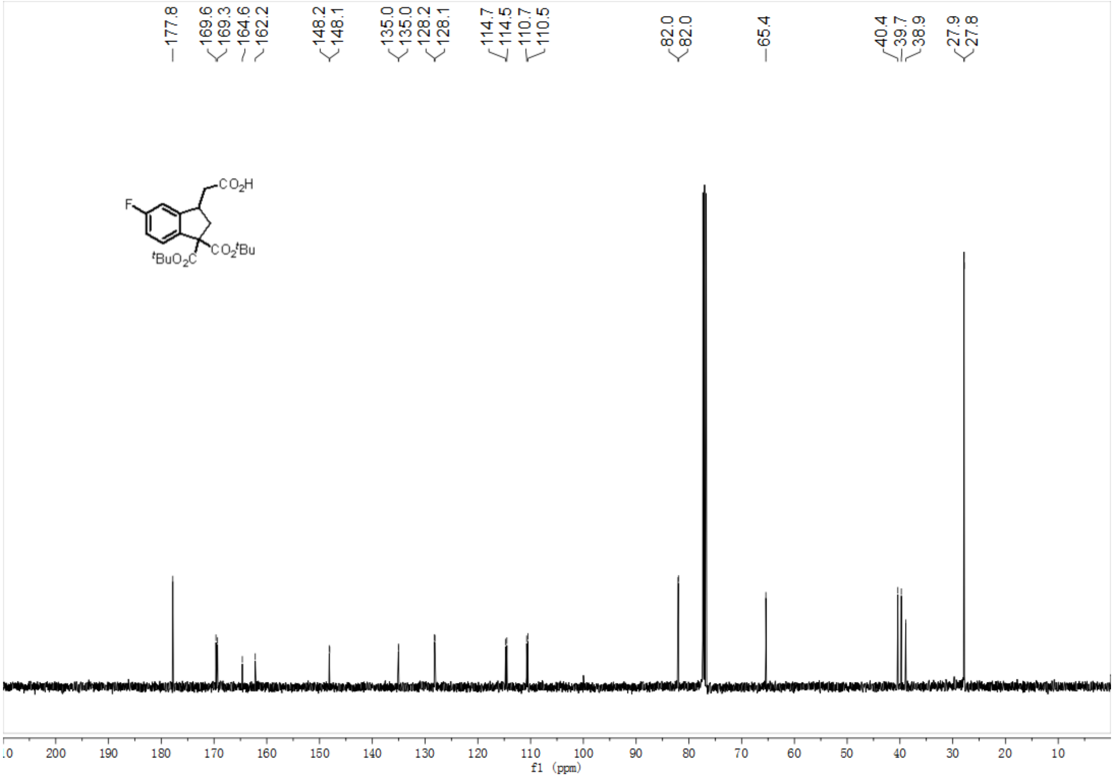

Supplementary Figure 73 ^{13}C NMR spectrum of **2z**

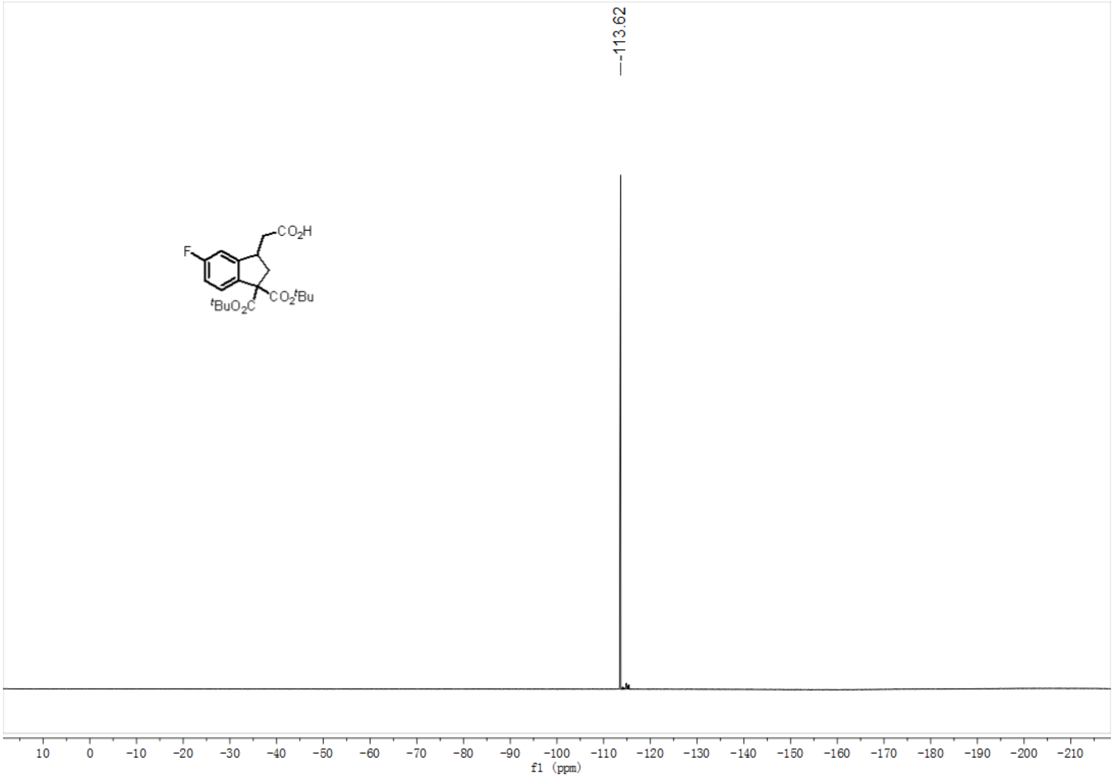

Supplementary Figure 74 ^{19}F NMR spectrum of **2z**

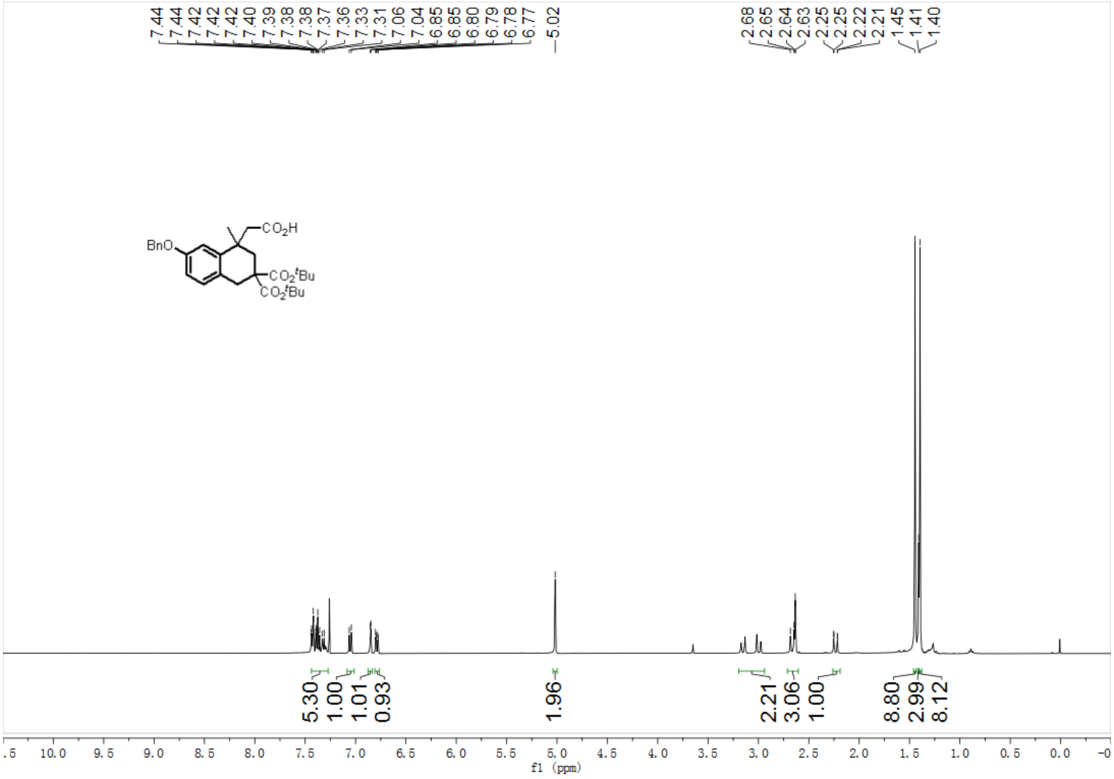

Supplementary Figure 75 ^{1}H NMR spectrum of **2aa**

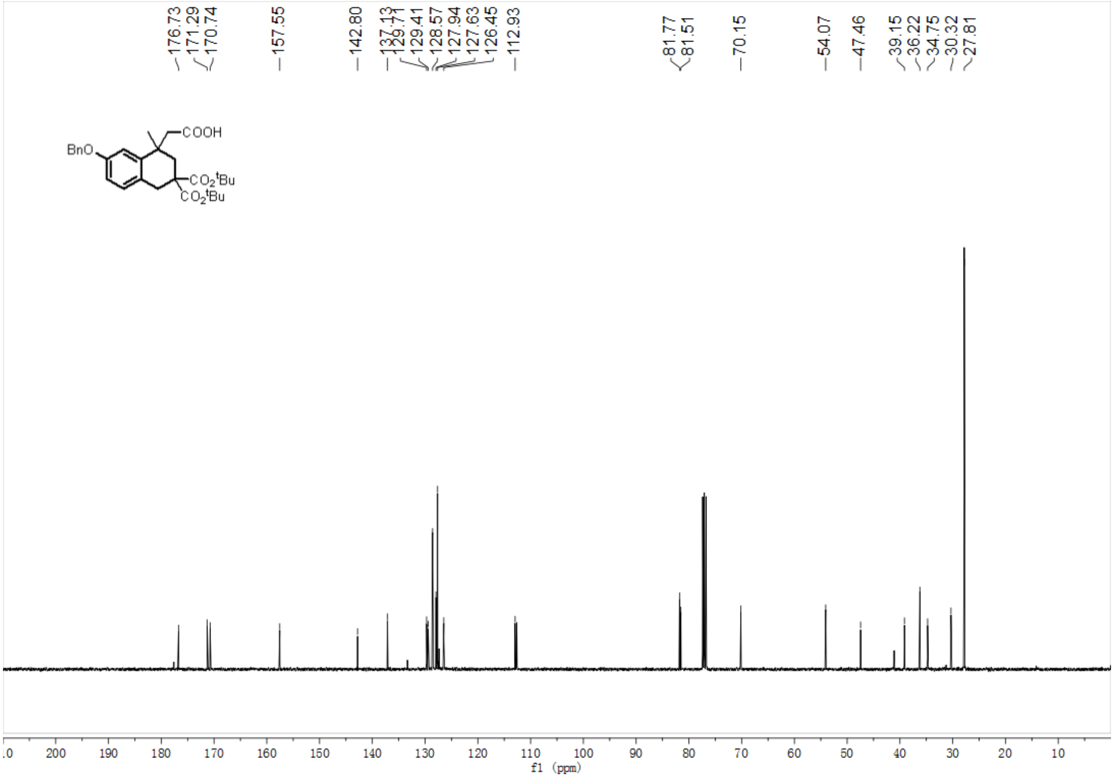

Supplementary Figure 76 ^{13}C NMR spectrum of **2aa**

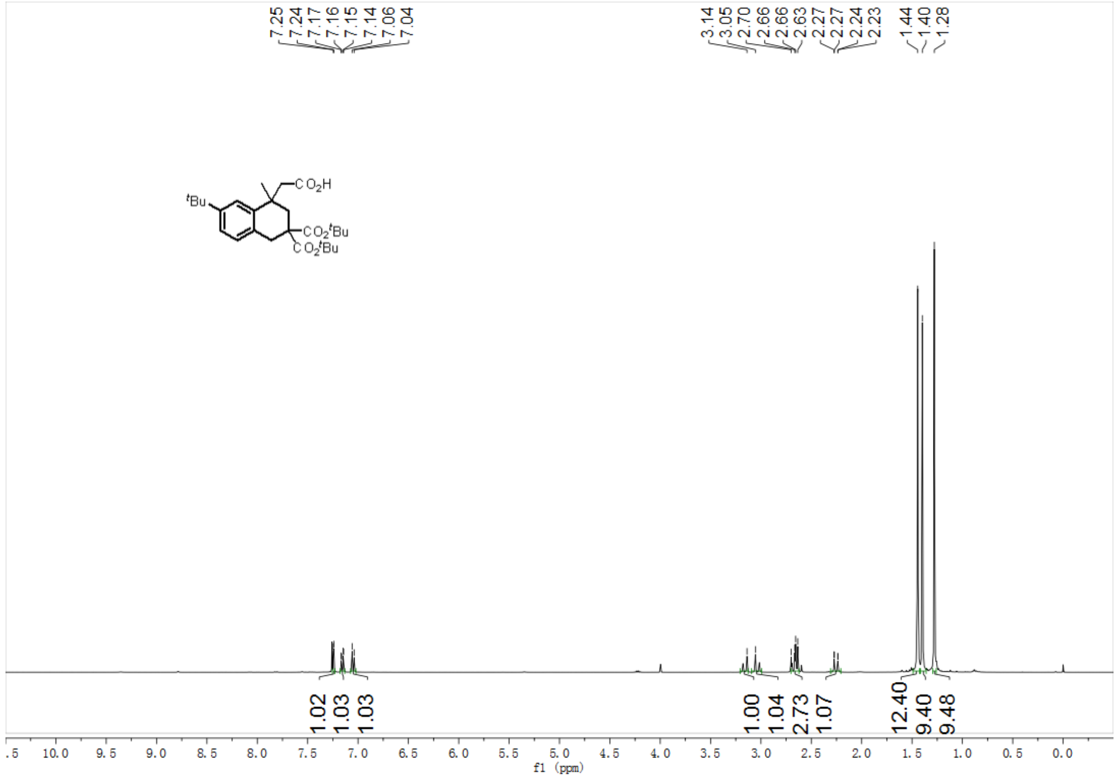

Supplementary Figure 77 ^{1}H NMR spectrum of **2ab**

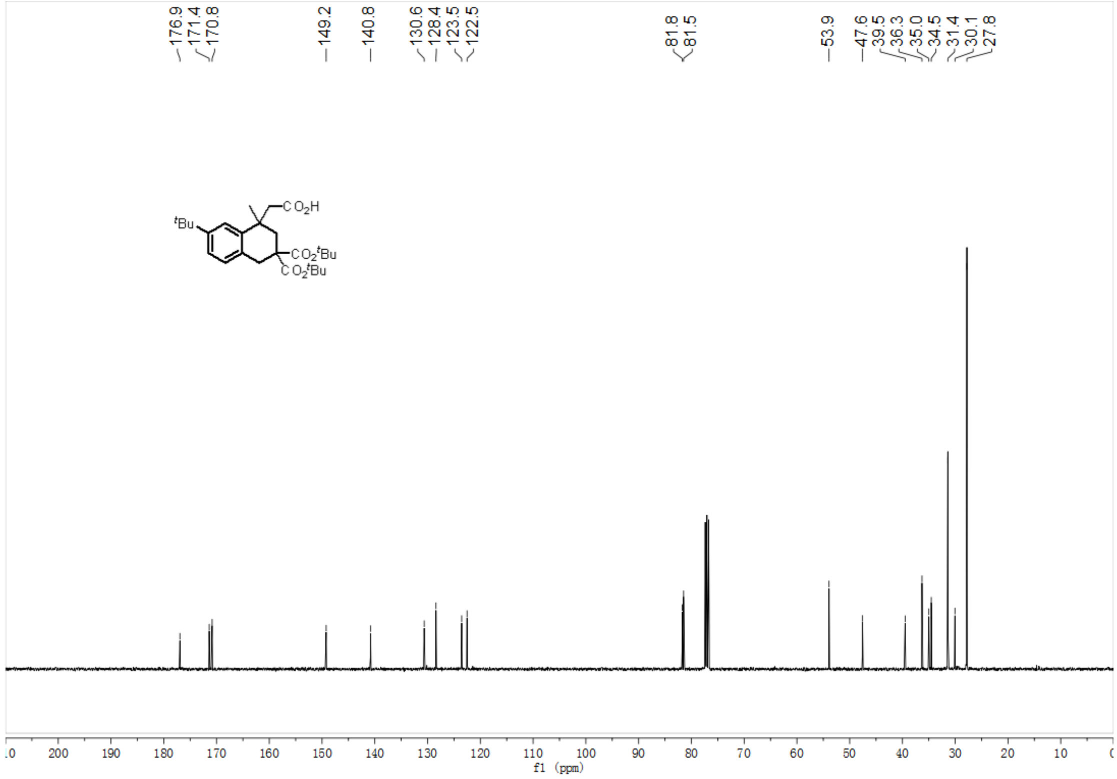

Supplementary Figure 78 ^{13}C NMR spectrum of **2ab**

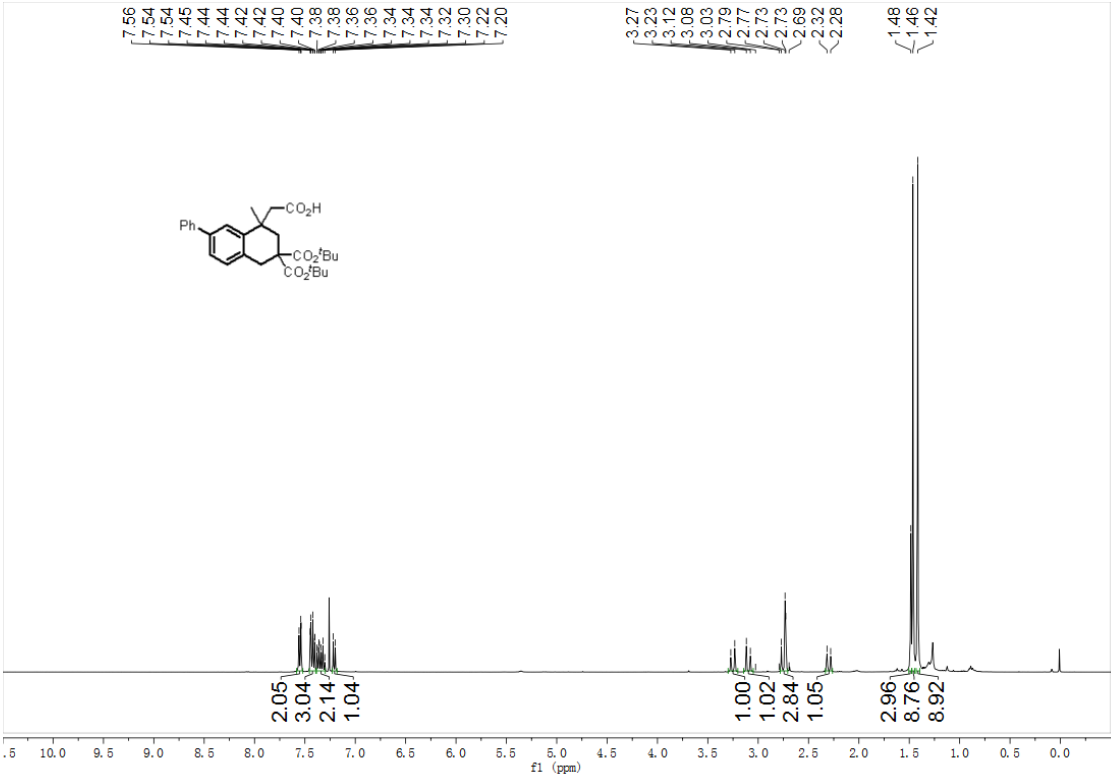

Supplementary Figure 79 ^{1}H NMR spectrum of **2ac**

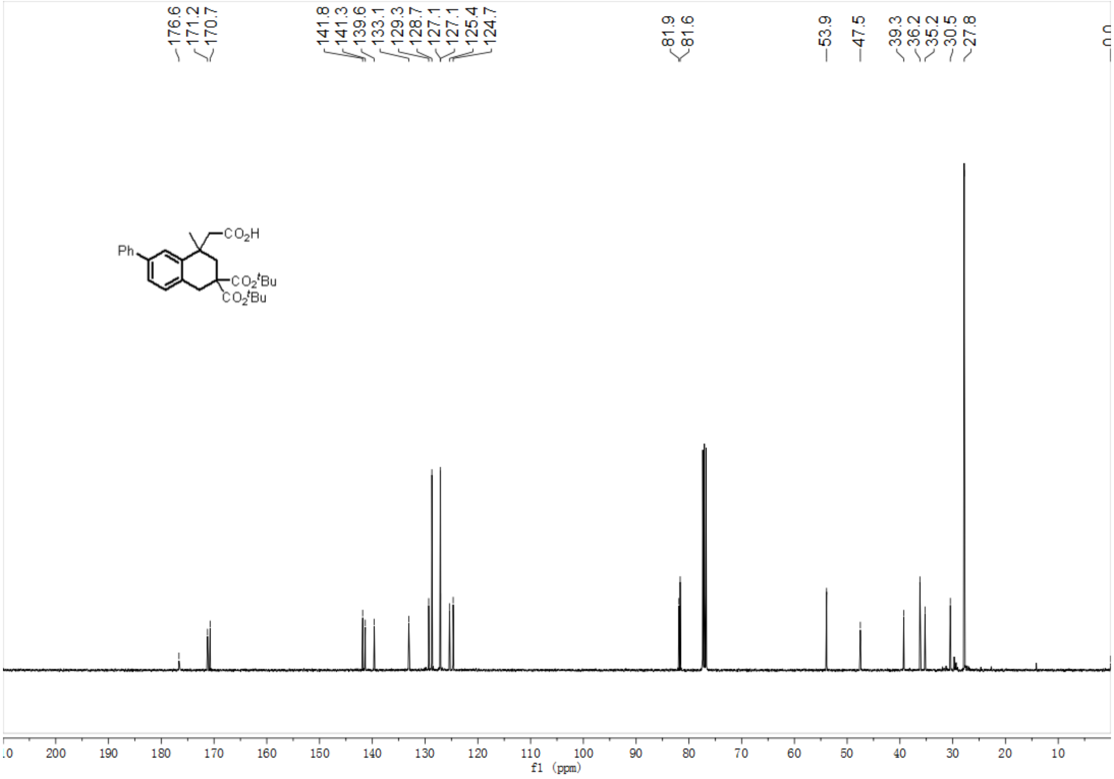

Supplementary Figure 80 ^{1}H NMR spectrum of **2ac**

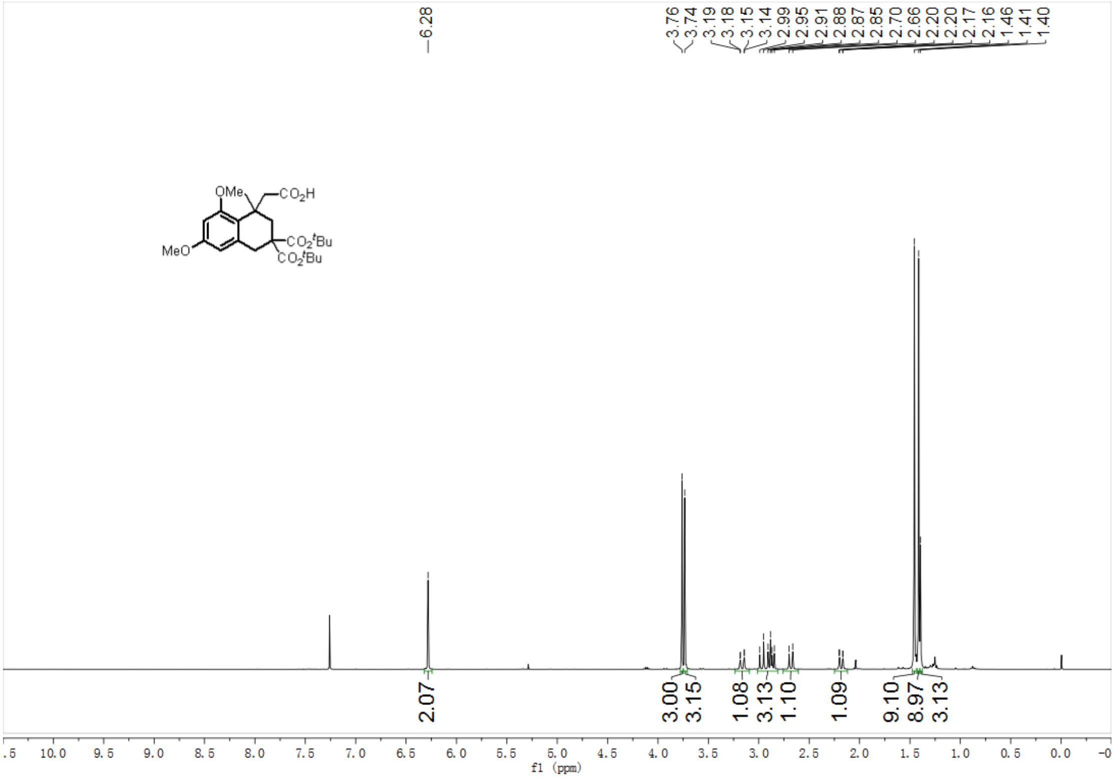

Supplementary Figure 81 ^{1}H NMR spectrum of **2ad**

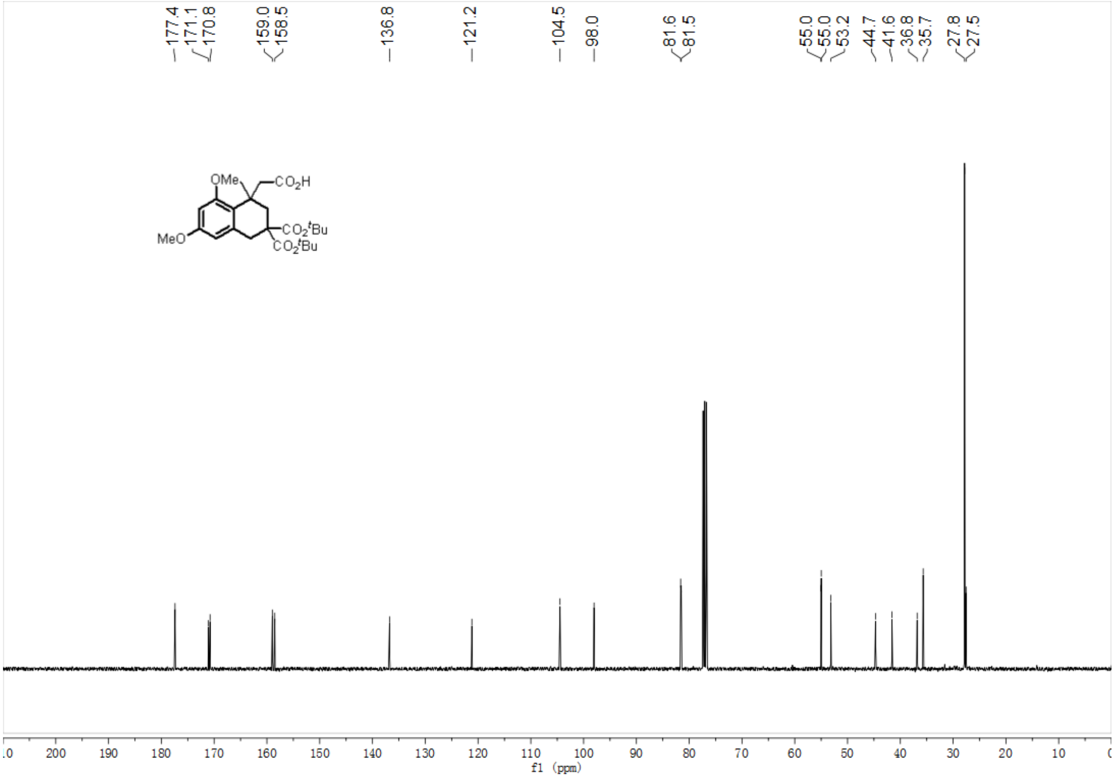

Supplementary Figure 82 ^{13}C NMR spectrum of **2ad**

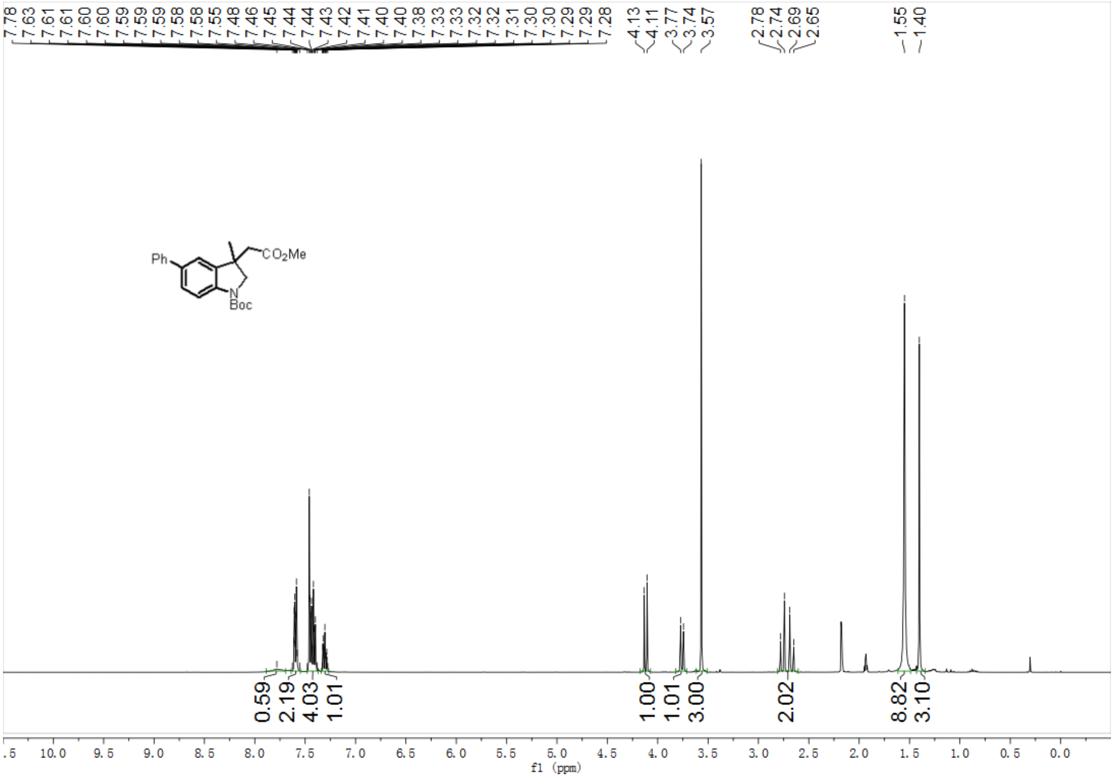

Supplementary Figure 83 ^{1}H NMR spectrum of **4a**

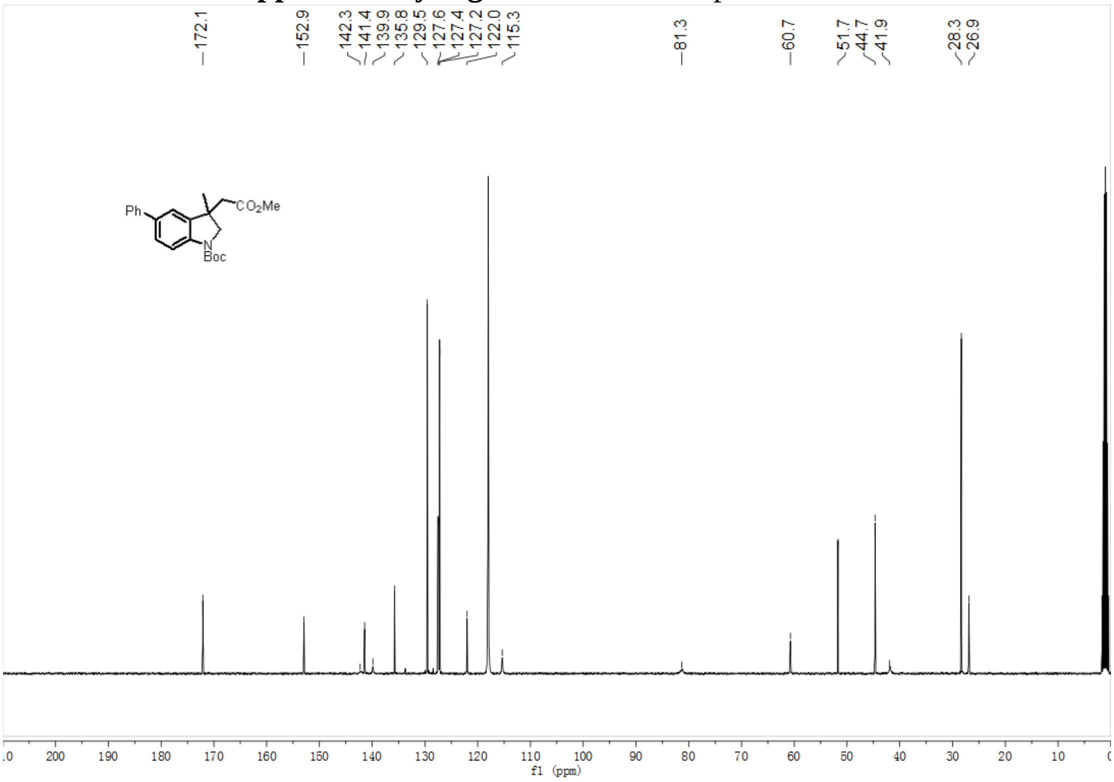

Supplementary Figure 84 ^{13}C NMR spectrum of **4a**

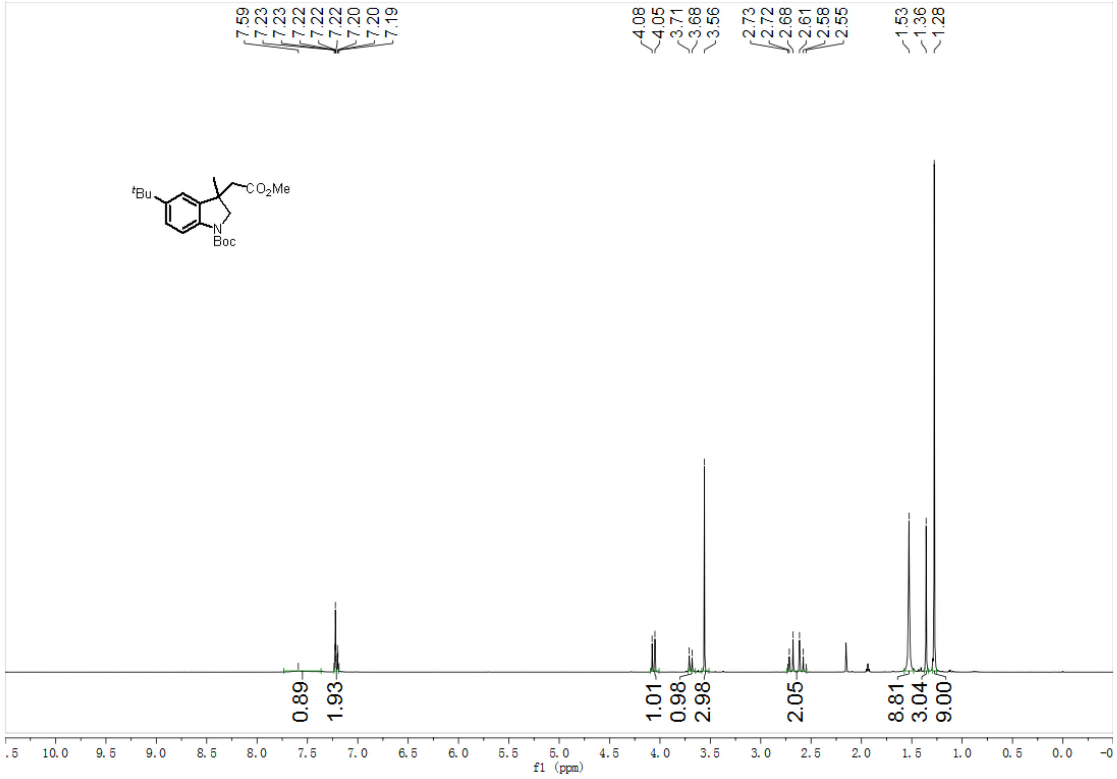

Supplementary Figure 85 ^{1}H NMR spectrum of **4b**

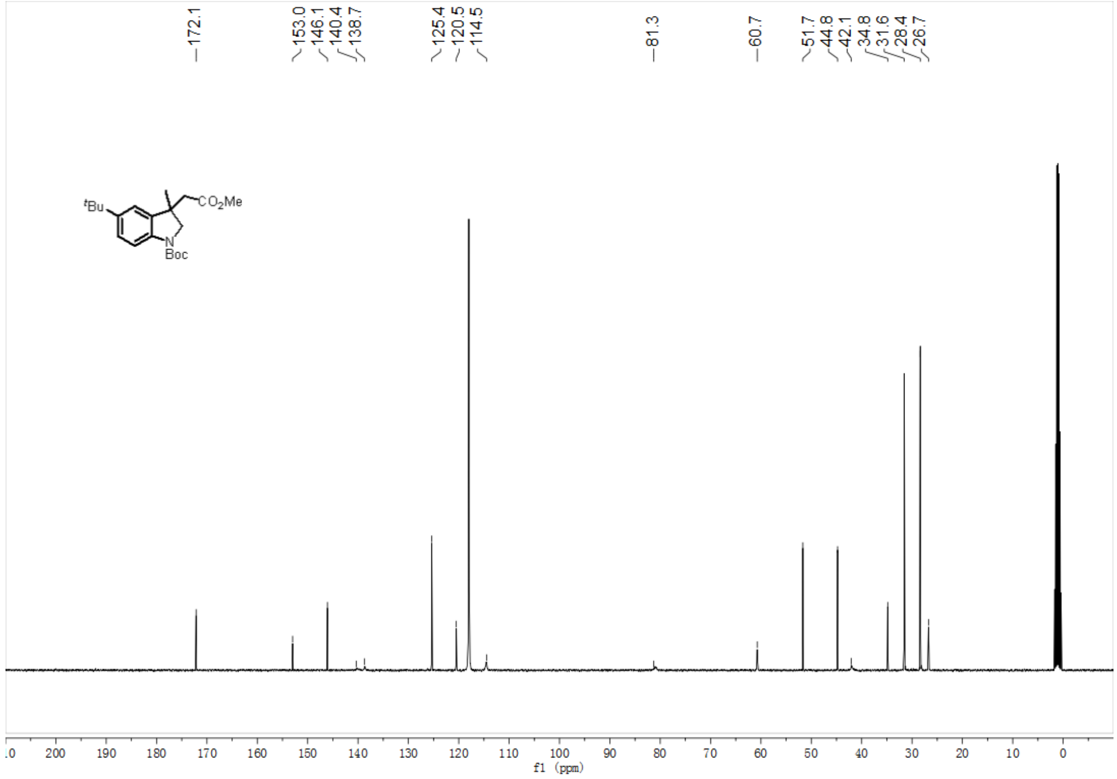

Supplementary Figure 86 ^{13}C NMR spectrum of **4b**

Tert-butyl 3-(2-methoxy-2-oxoethyl)-3-methylindoline-1-carboxylate (4c)

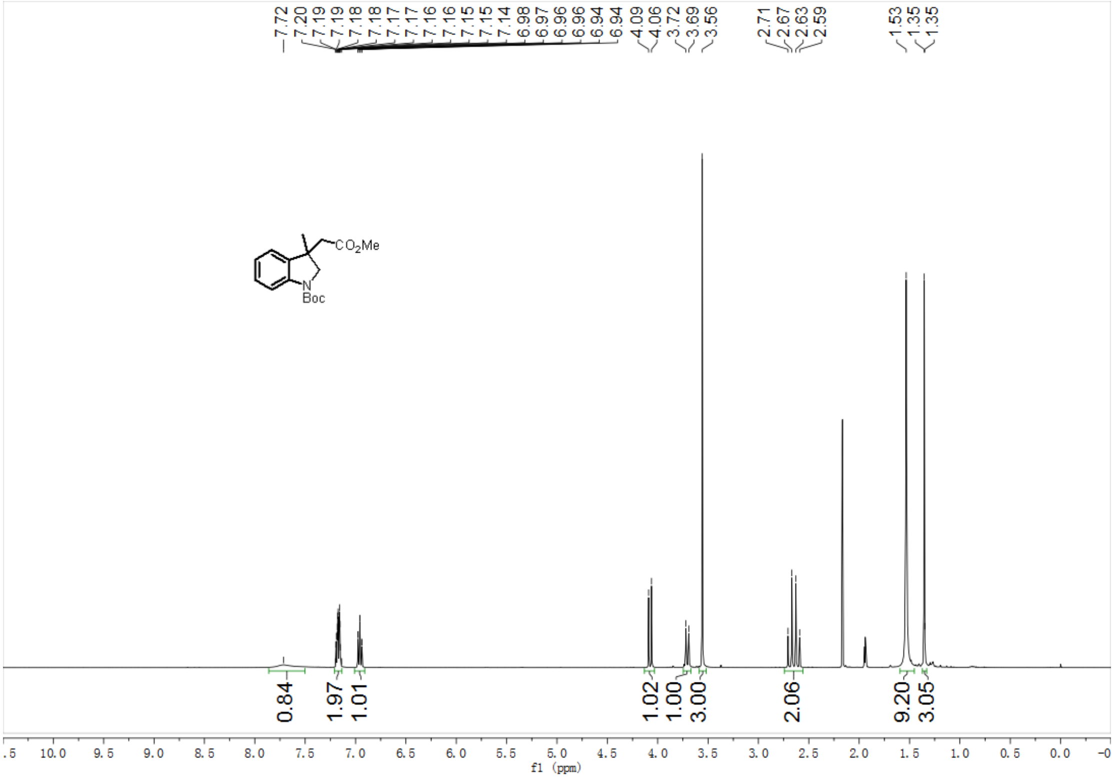

Supplementary Figure 87 ^{1}H NMR spectrum of **4c**

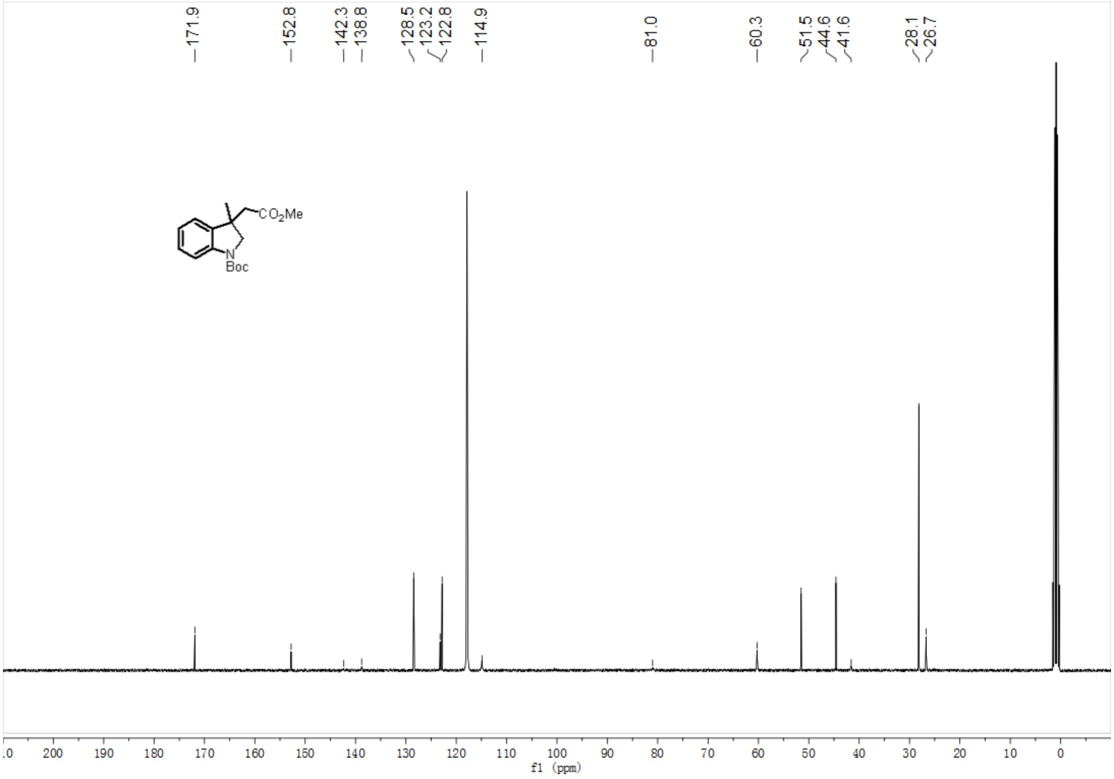

Supplementary Figure 88 ^{13}C NMR spectrum of **4c**

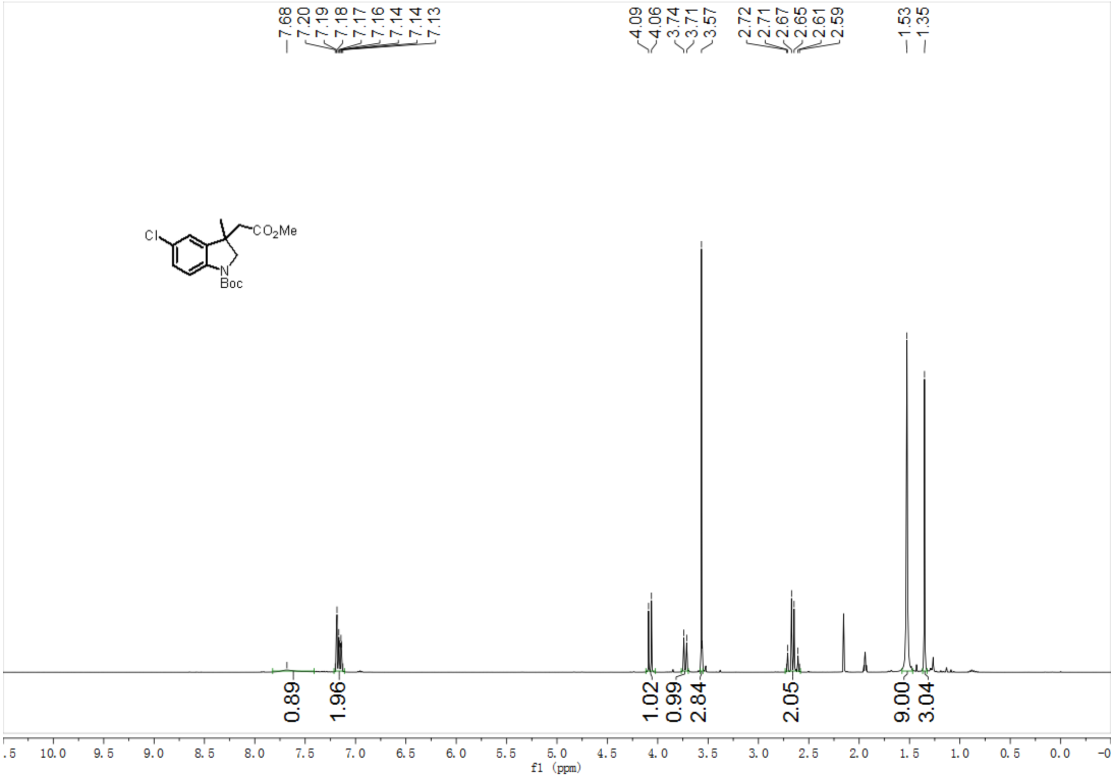

Supplementary Figure 89 ^{1}H NMR spectrum of **4d**

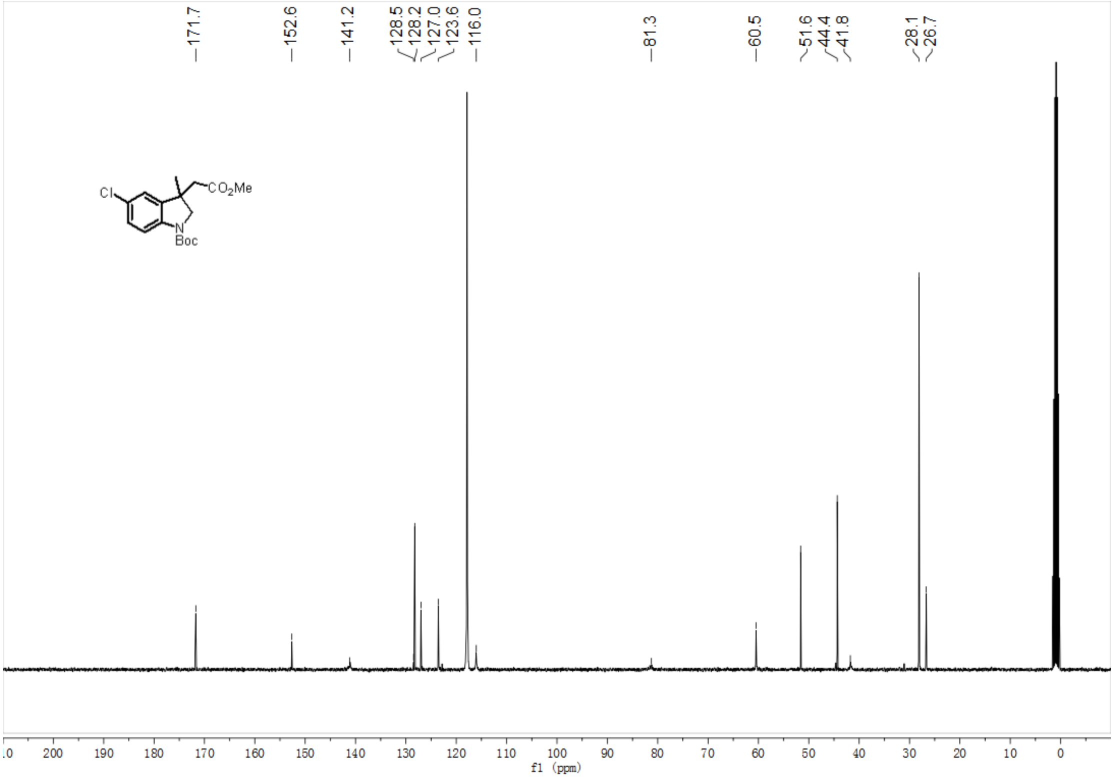

Supplementary Figure 90 ^{13}C NMR spectrum of **4d**

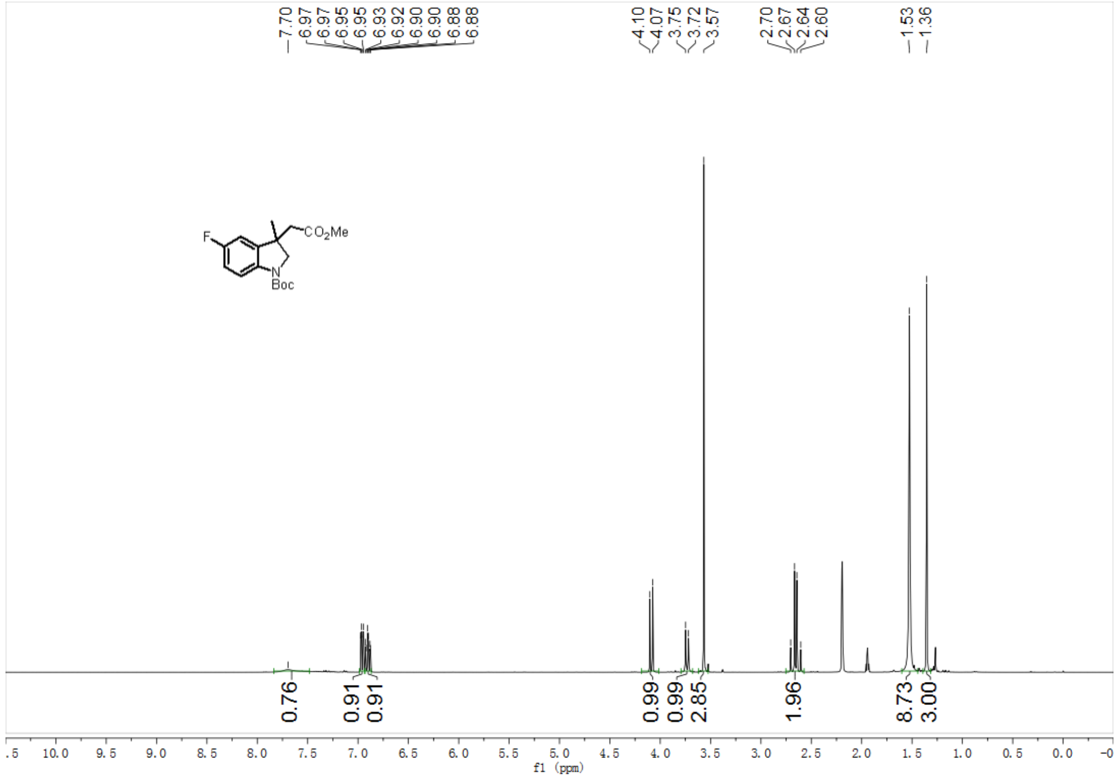

Supplementary Figure 91 ^{1}H NMR spectrum of **4e**

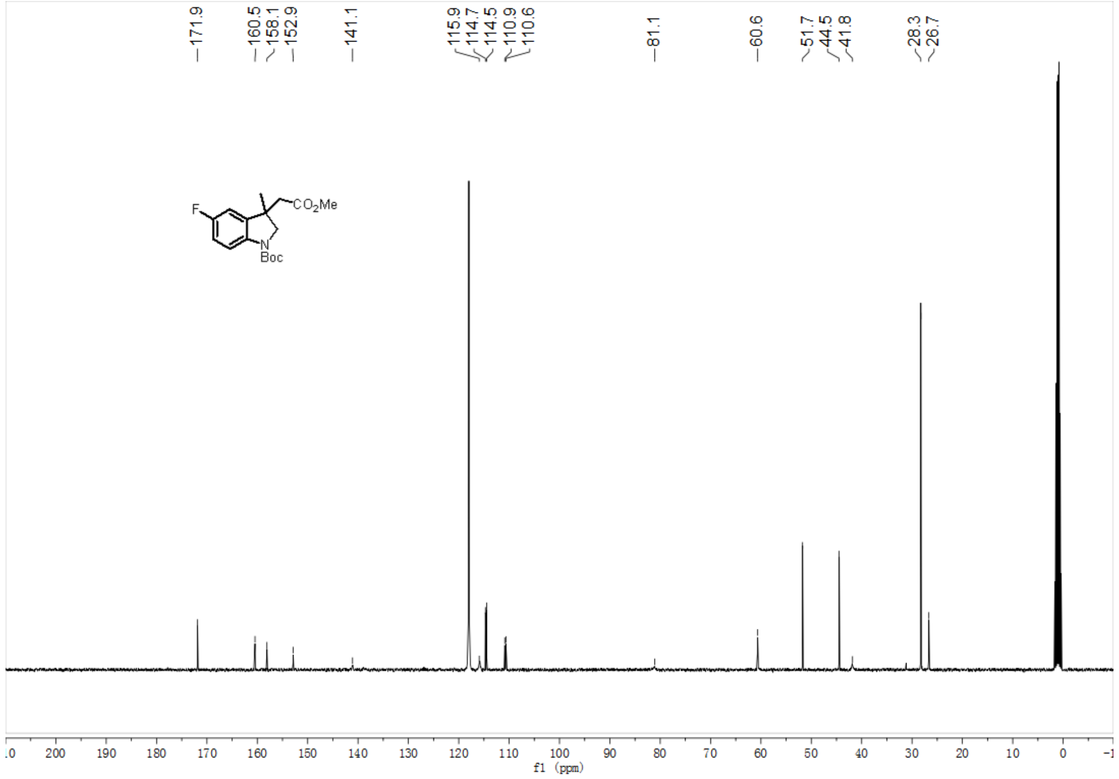

Supplementary Figure 92 ^{13}C NMR spectrum of **4e**

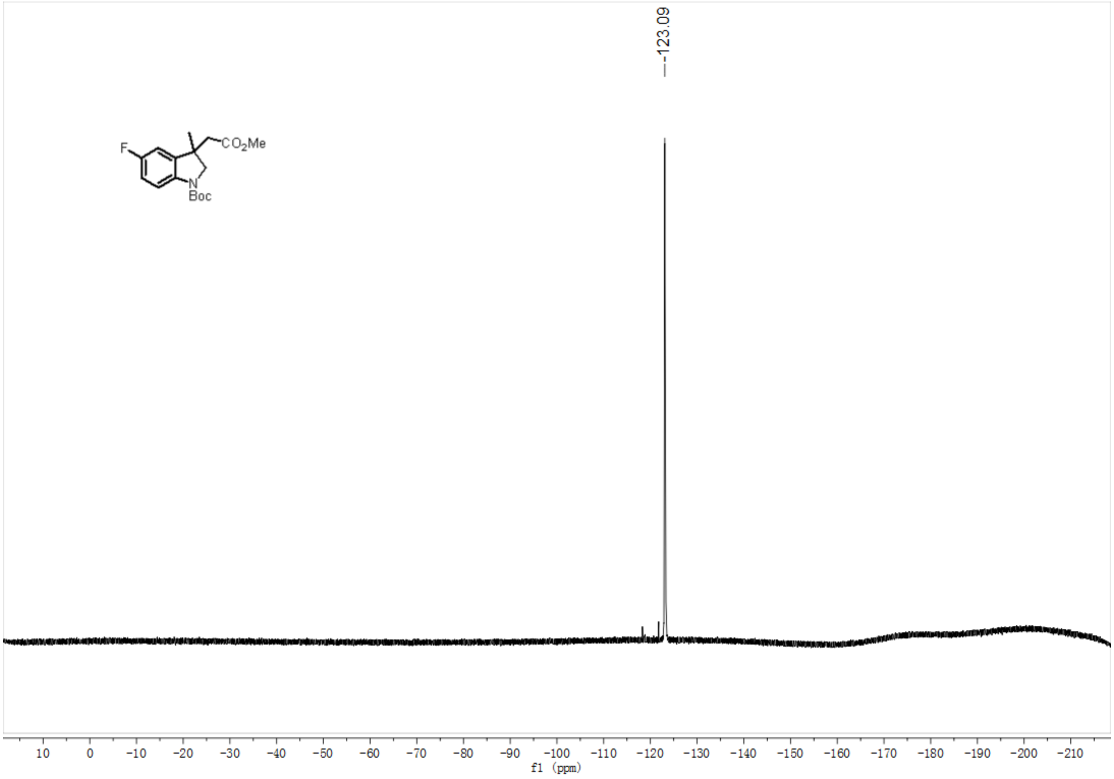

Supplementary Figure 93 ^{19}F NMR spectrum of **4e**

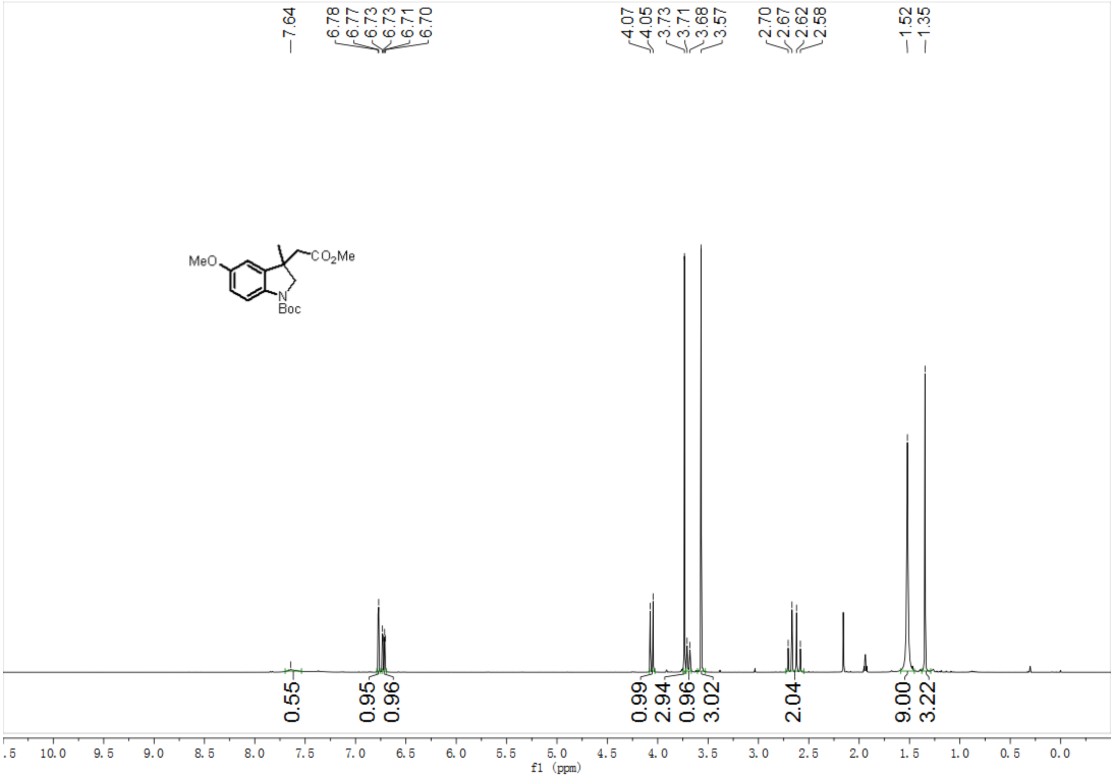

Supplementary Figure 94 ^{1}H NMR spectrum of **4f**

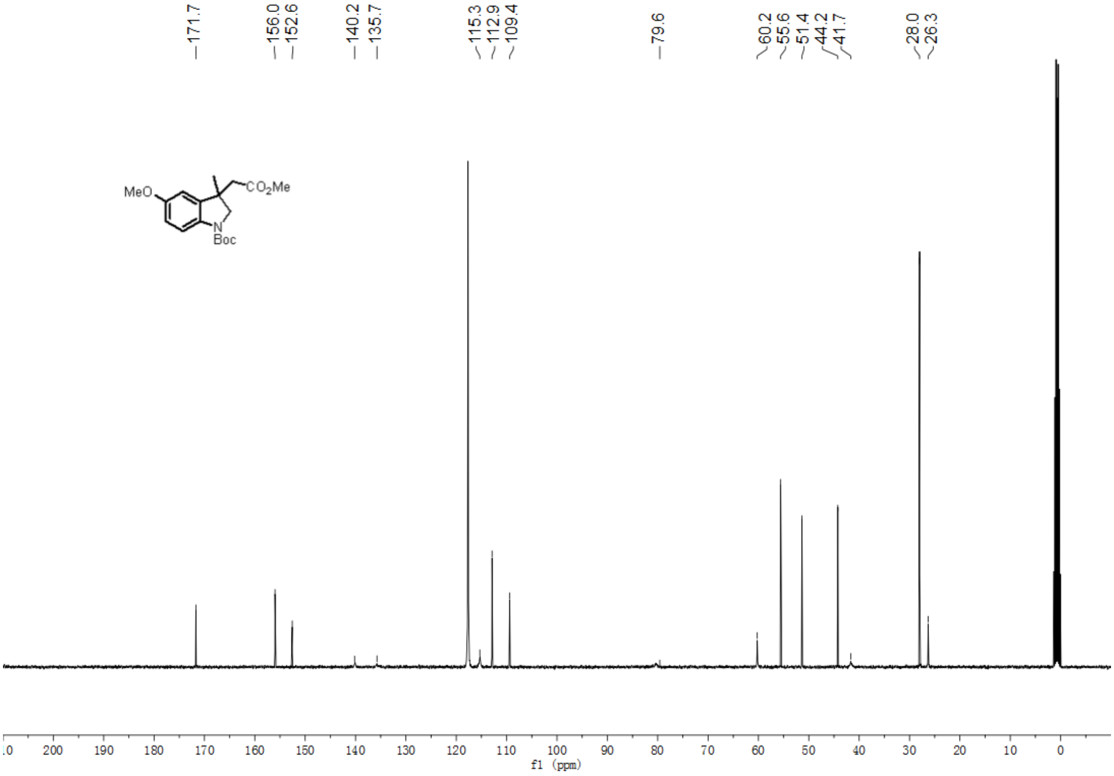

Supplementary Figure 95 ^{13}C NMR spectrum of **4f**

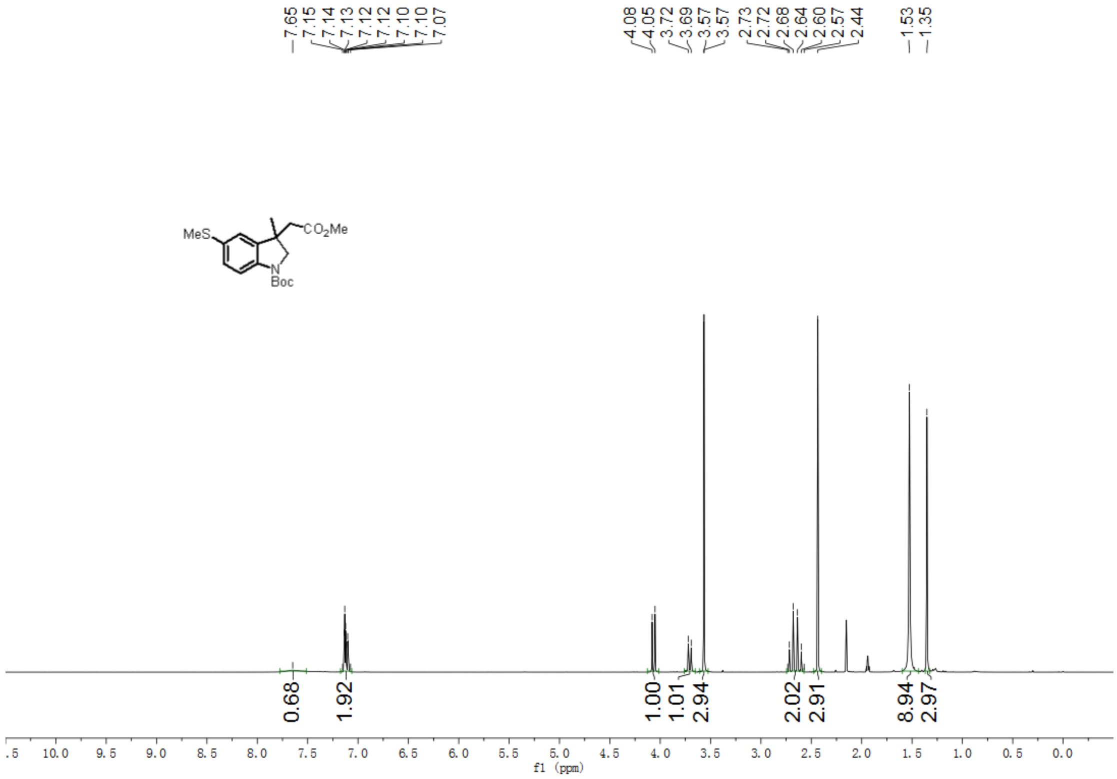

Supplementary Figure 96 ^{1}H NMR spectrum of **4g**

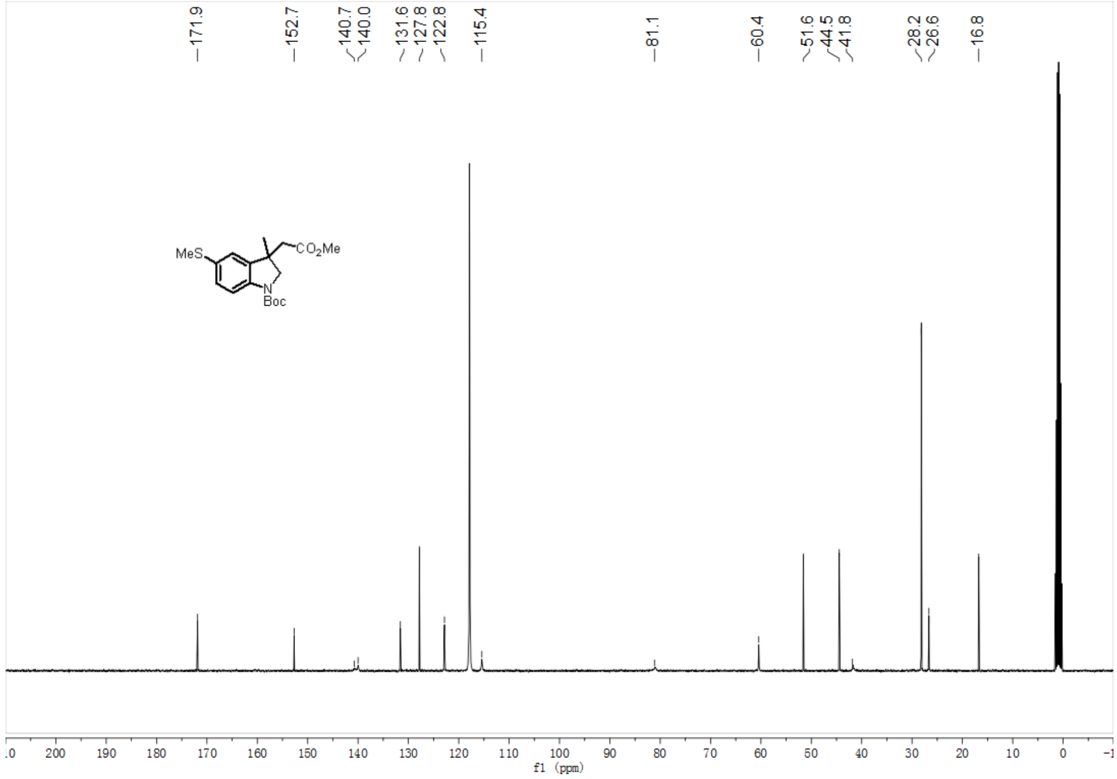

Supplementary Figure 97 ^{13}C NMR spectrum of **4g**

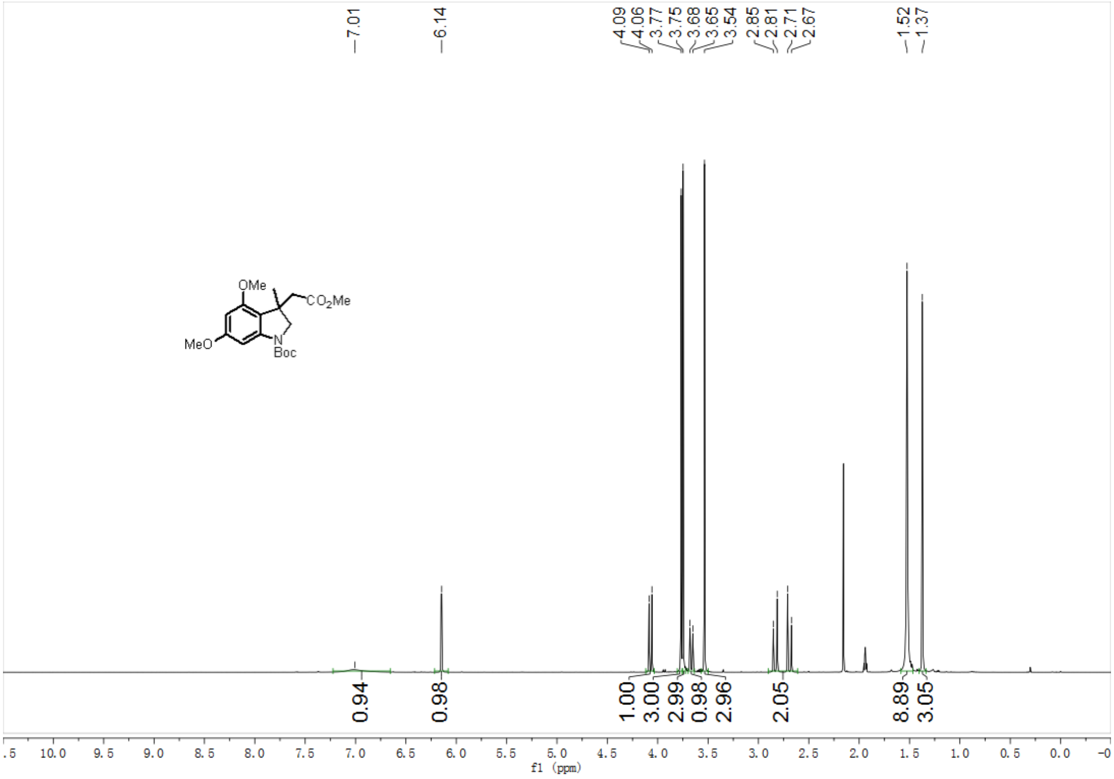

Supplementary Figure 98 ^{1}H NMR spectrum of **4h**

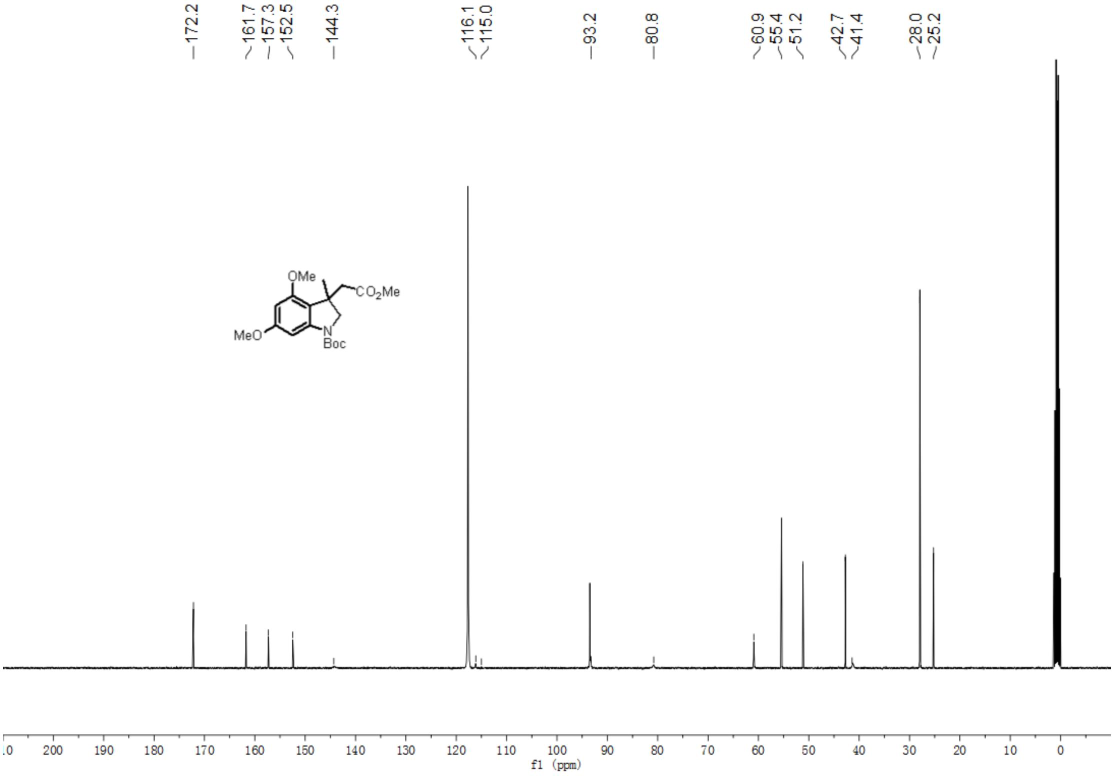

Supplementary Figure 99 ^{13}C NMR spectrum of **4h**

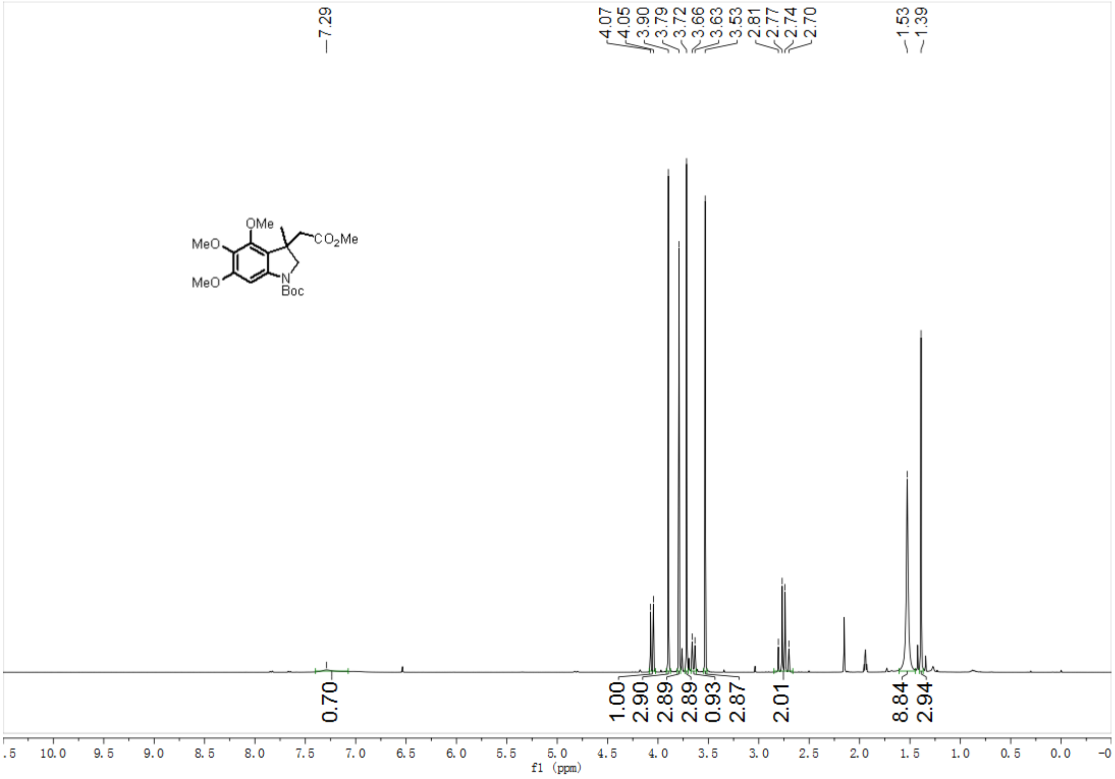

Supplementary Figure 100 ^{1}H NMR spectrum of **4i**

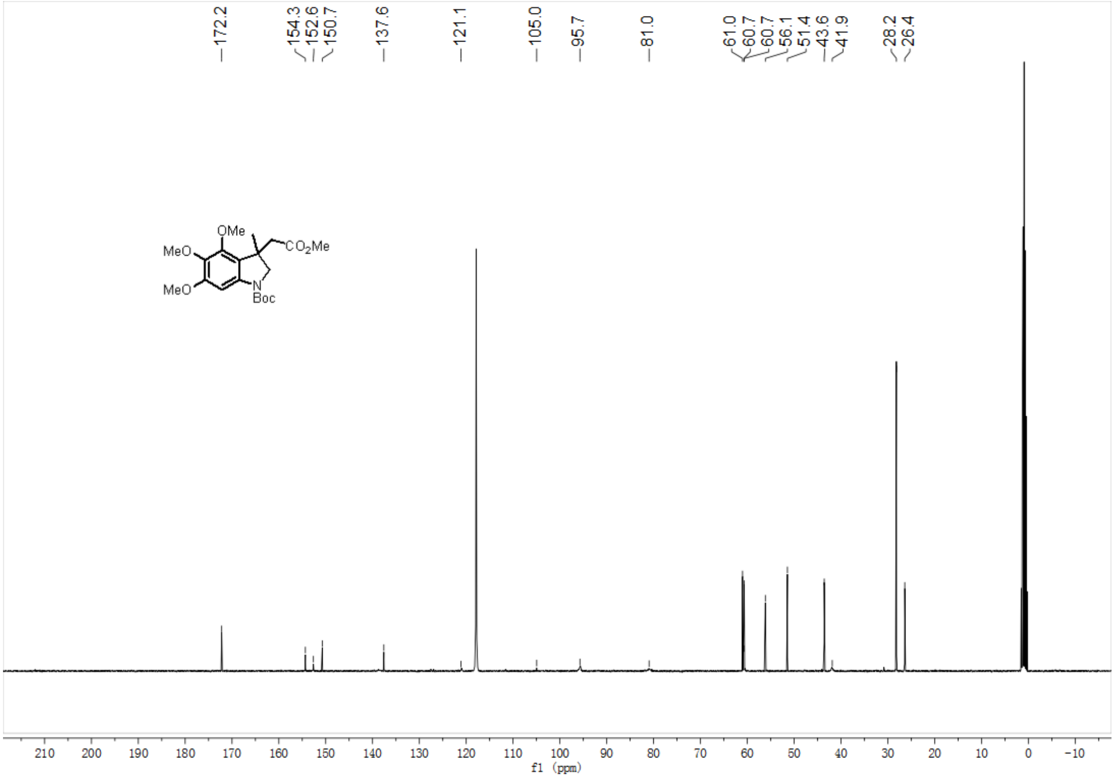

Supplementary Figure 101 ^{13}C NMR spectrum of **4i**

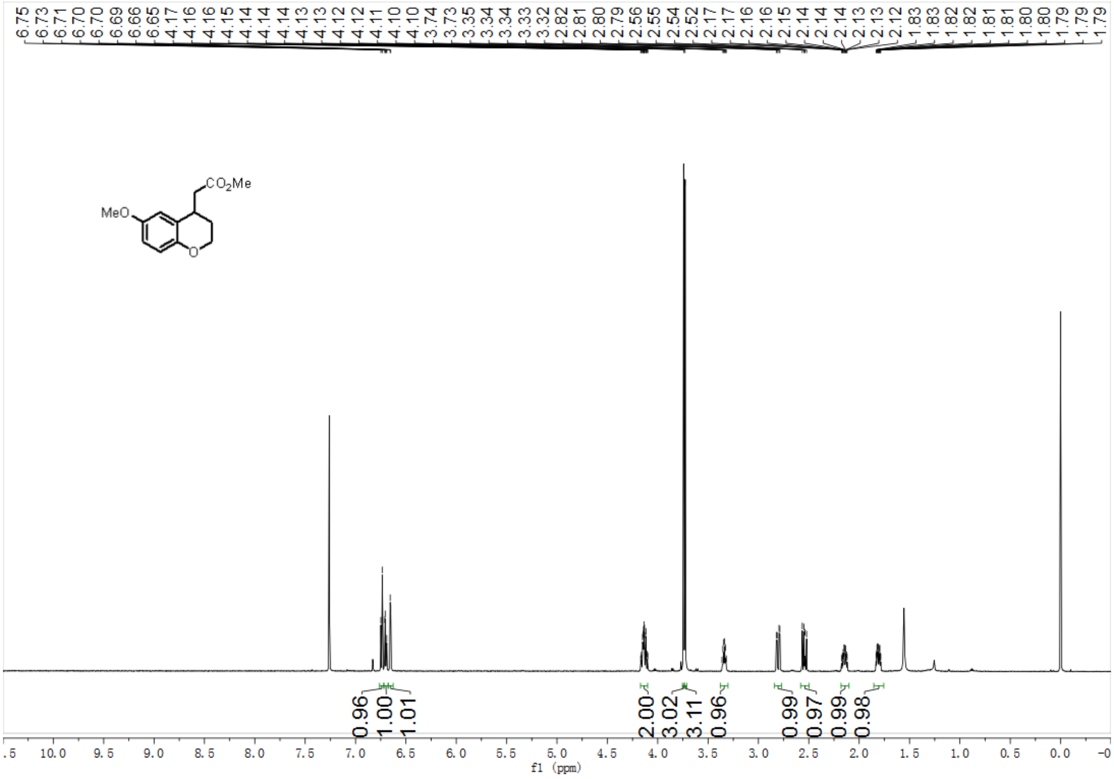

Supplementary Figure 102 ^{1}H NMR spectrum of **6a**

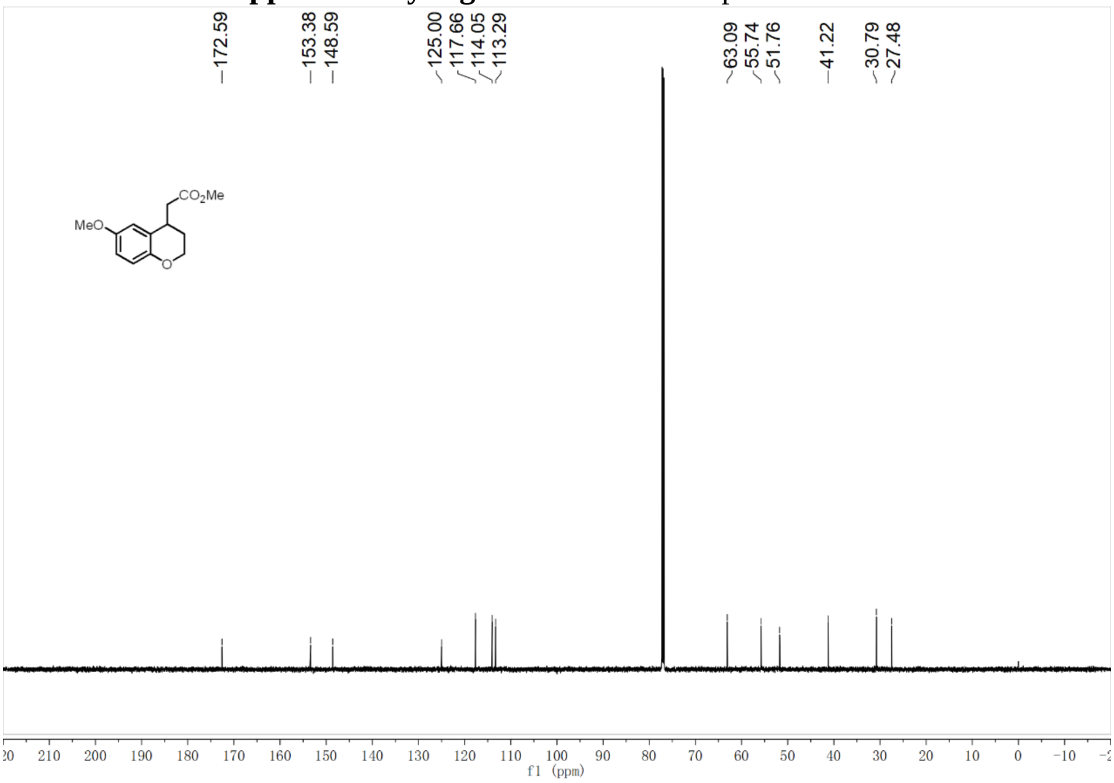

Supplementary Figure 103 ^{13}C NMR spectrum of **6a**

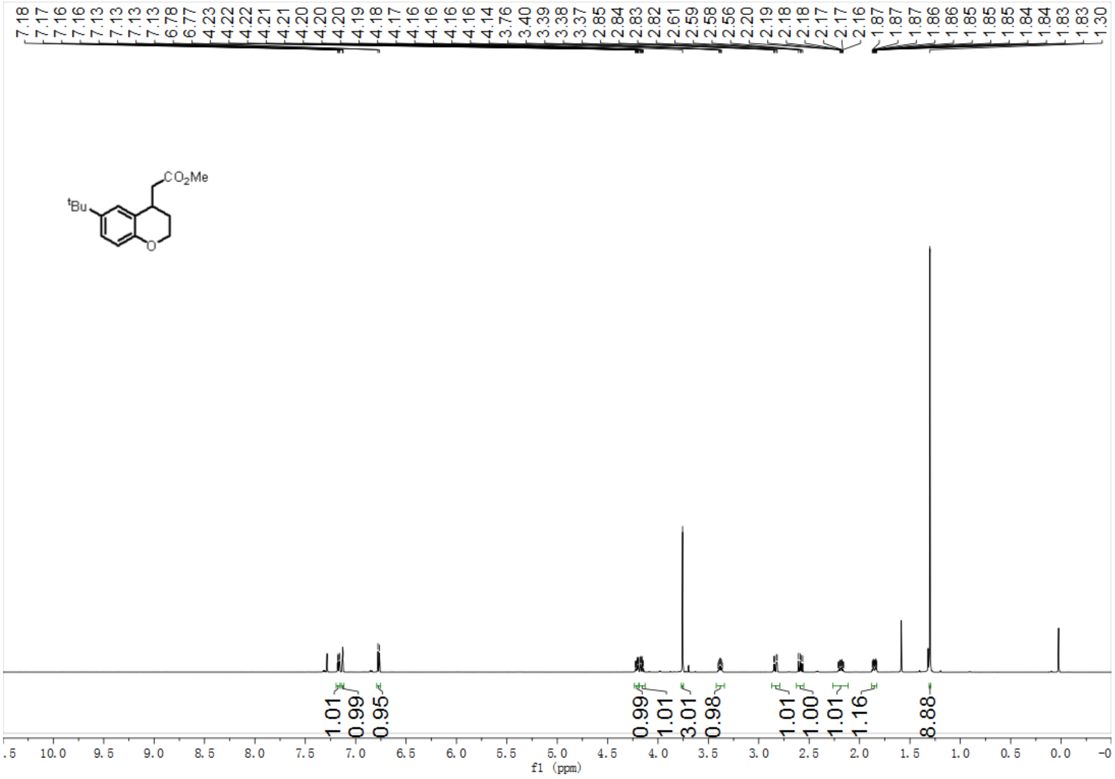

Supplementary Figure 104 ^{1}H NMR spectrum of **6b**

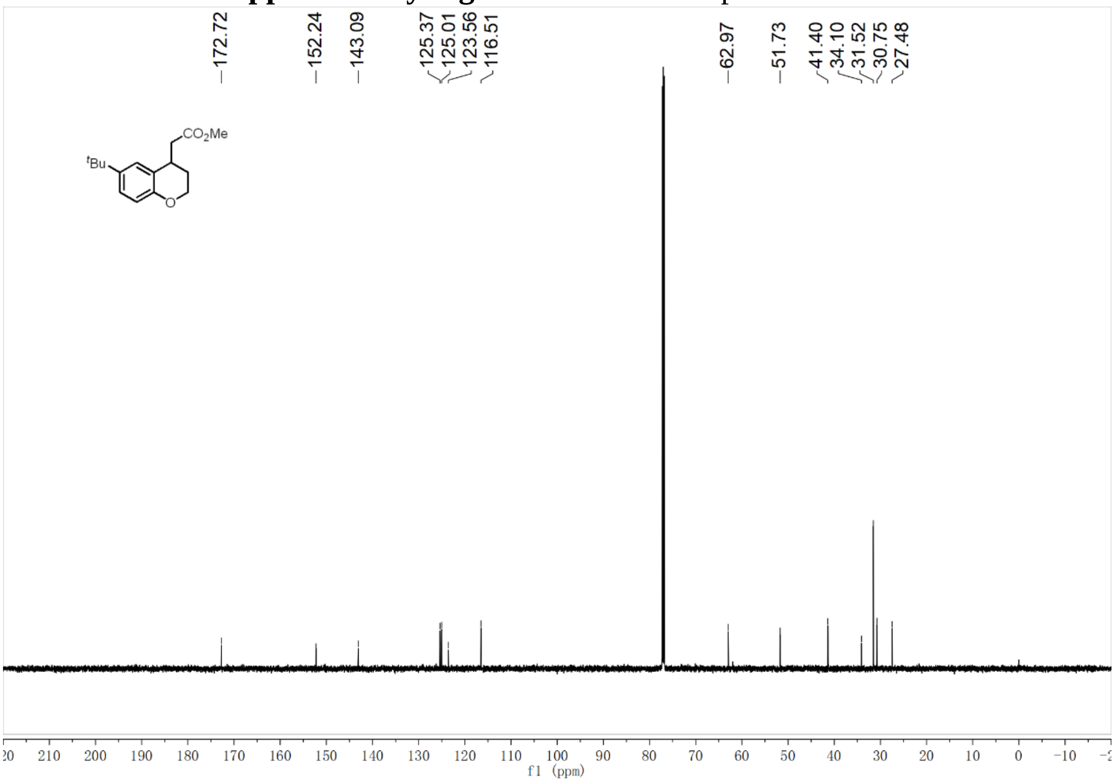

Supplementary Figure 105 ^{13}C NMR spectrum of **6b**

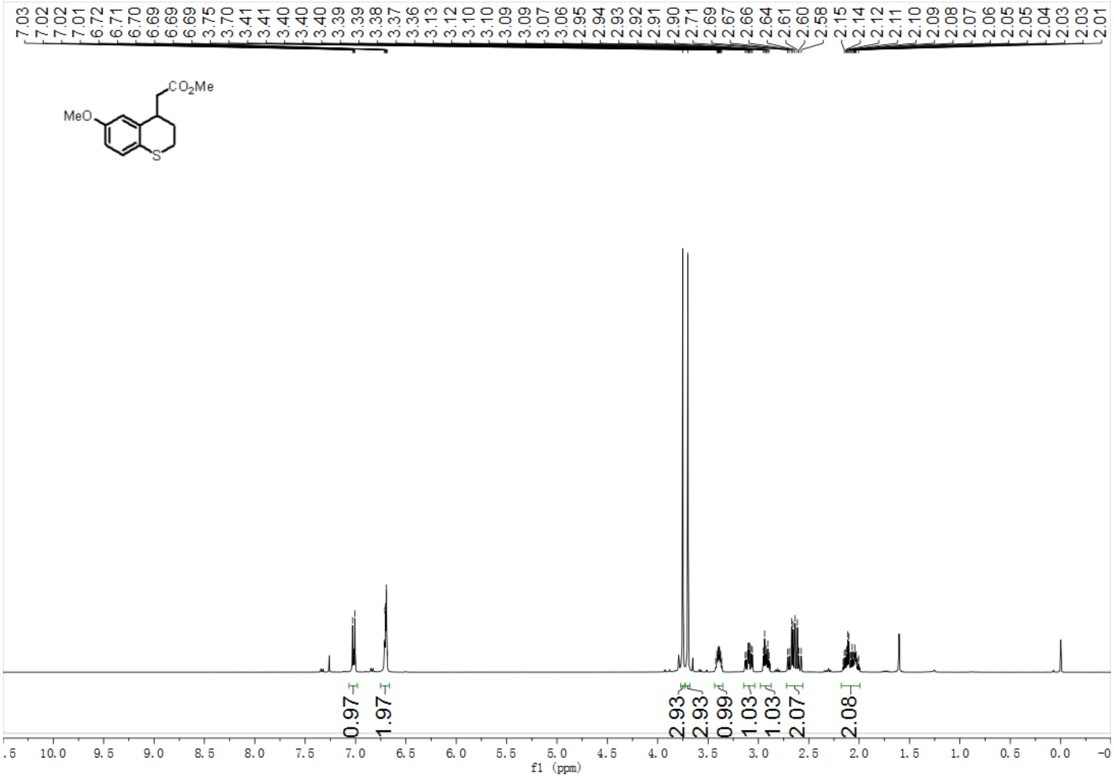

Supplementary Figure 106 ^{1}H NMR spectrum of **6c**

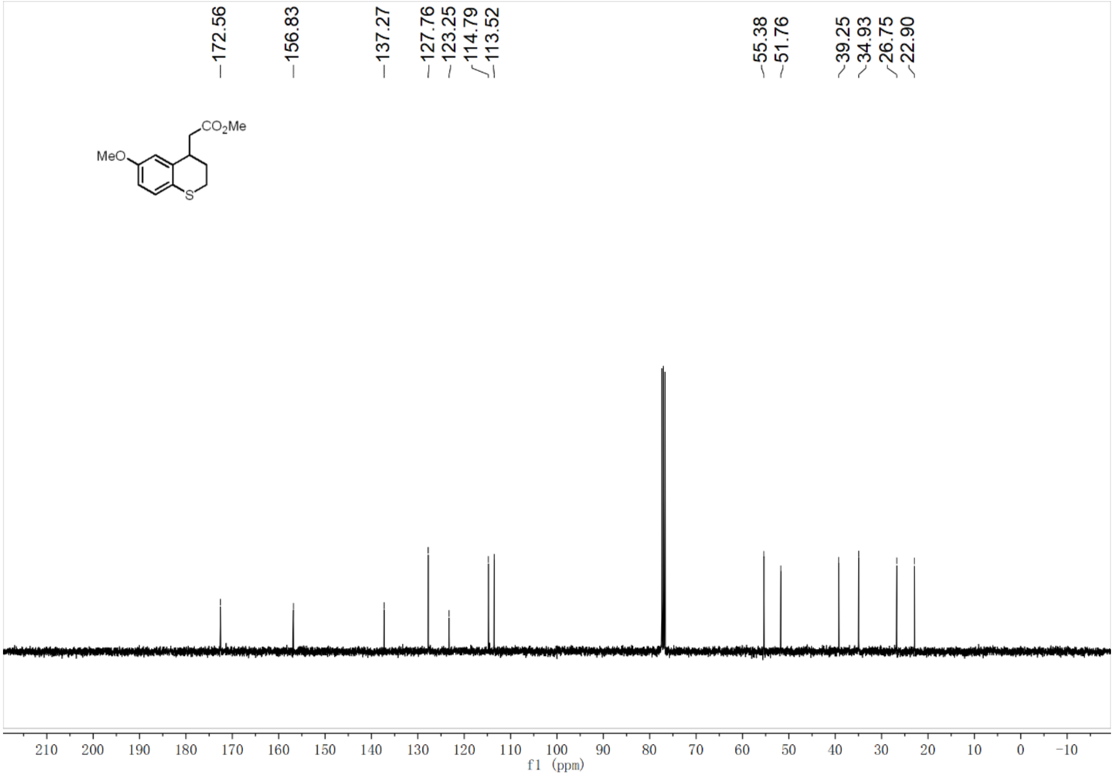

Supplementary Figure 107 ^{13}C NMR spectrum of **6c**

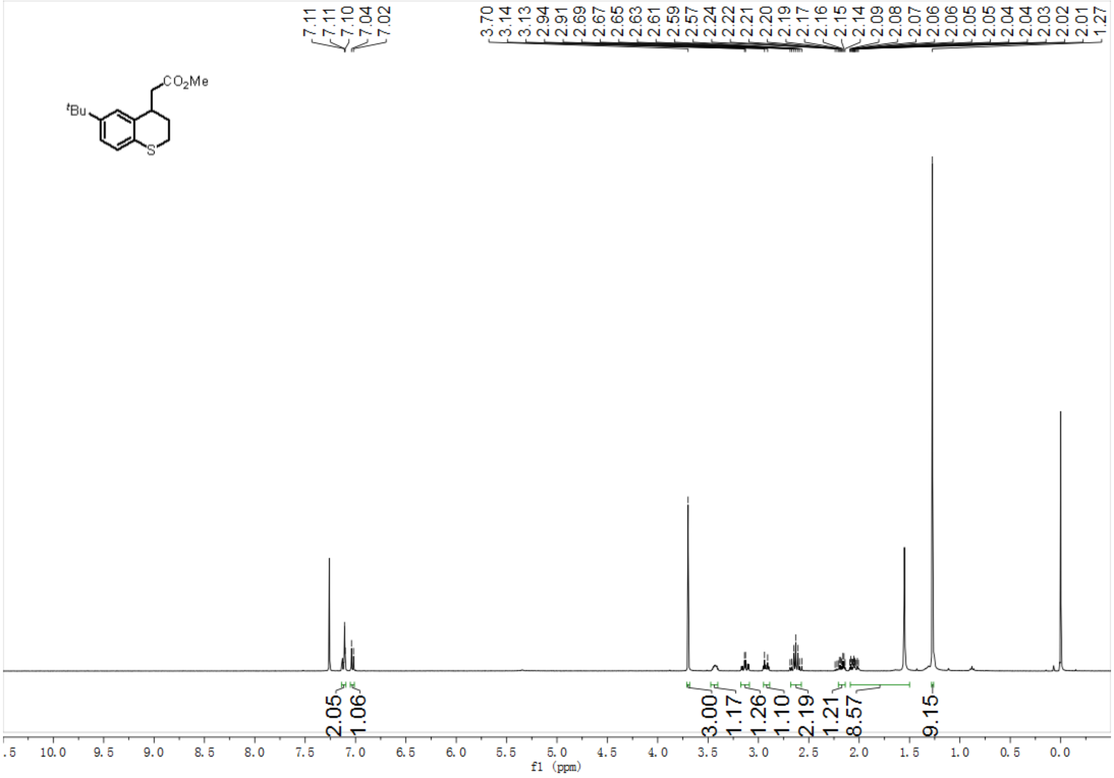

Supplementary Figure 108 ^{1}H NMR spectrum of **6d**

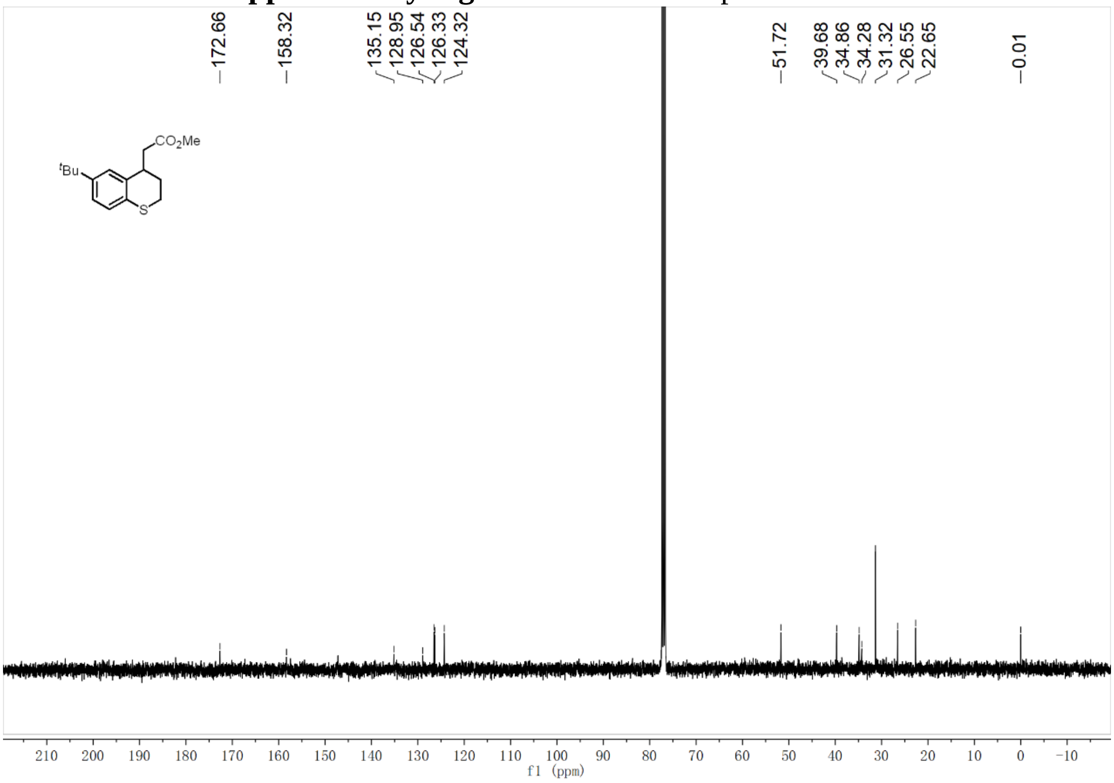

Supplementary Figure 109 ^{13}C NMR spectrum of **6d**

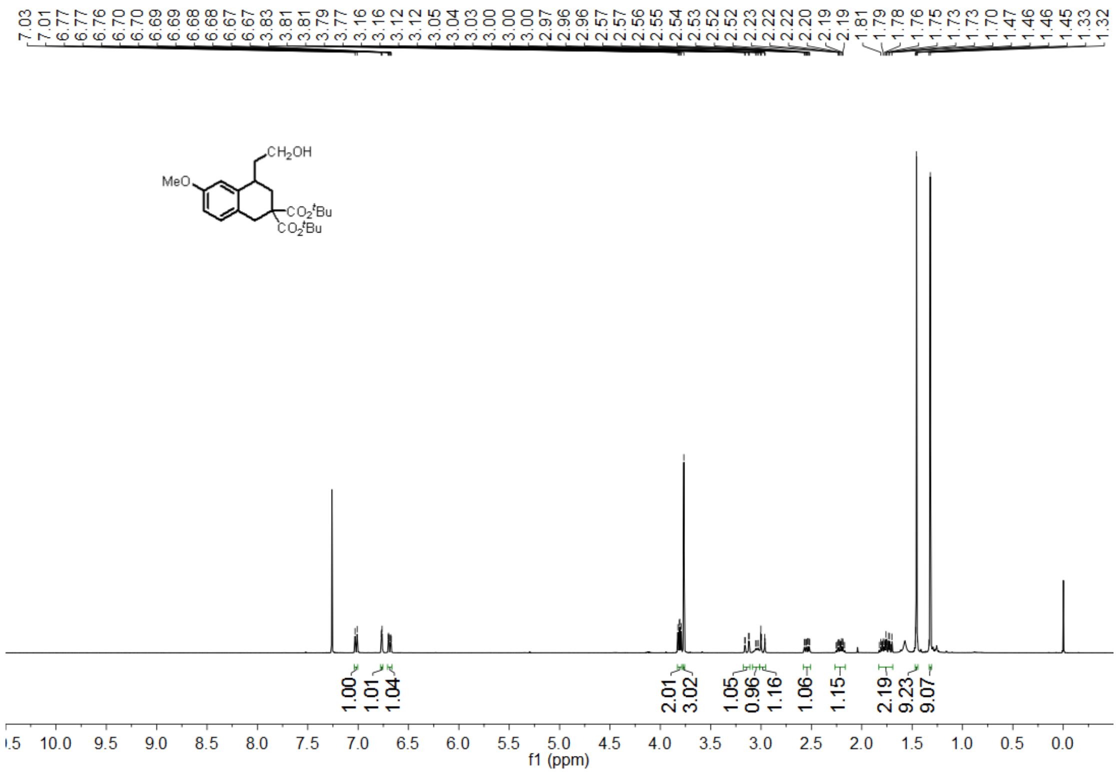

Supplementary Figure 110 ^{1}H NMR spectrum of **7**

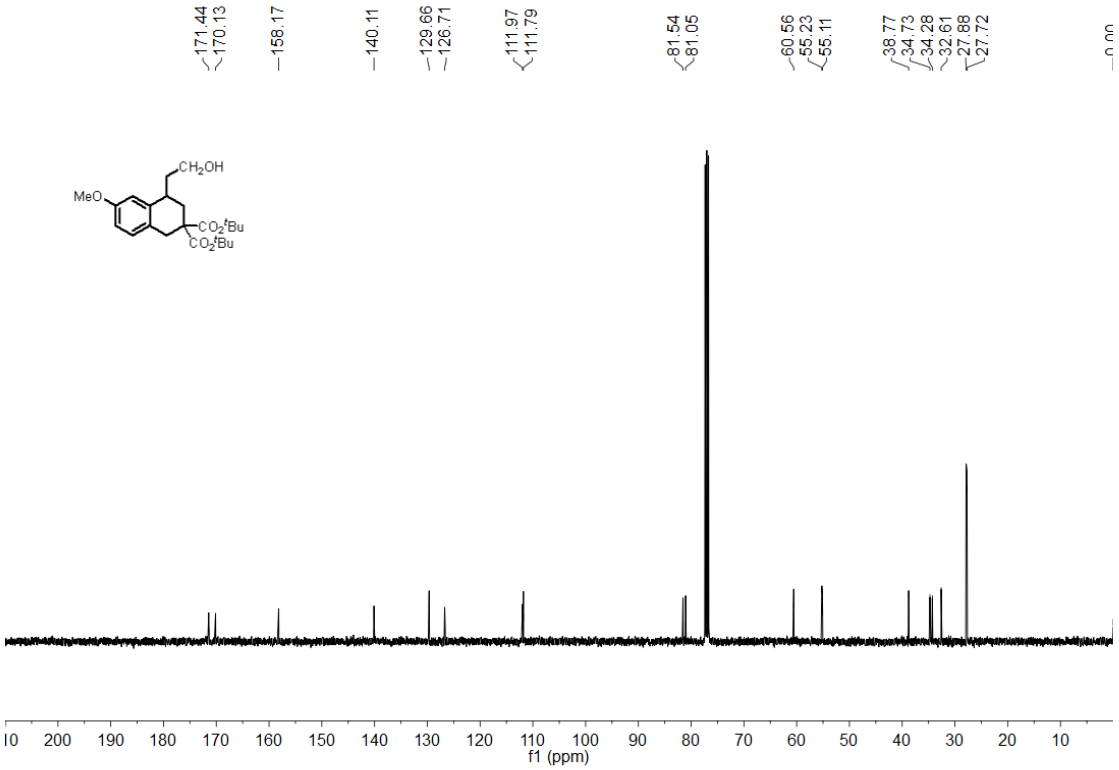

Supplementary Figure 111 ^{13}C NMR spectrum of **7**

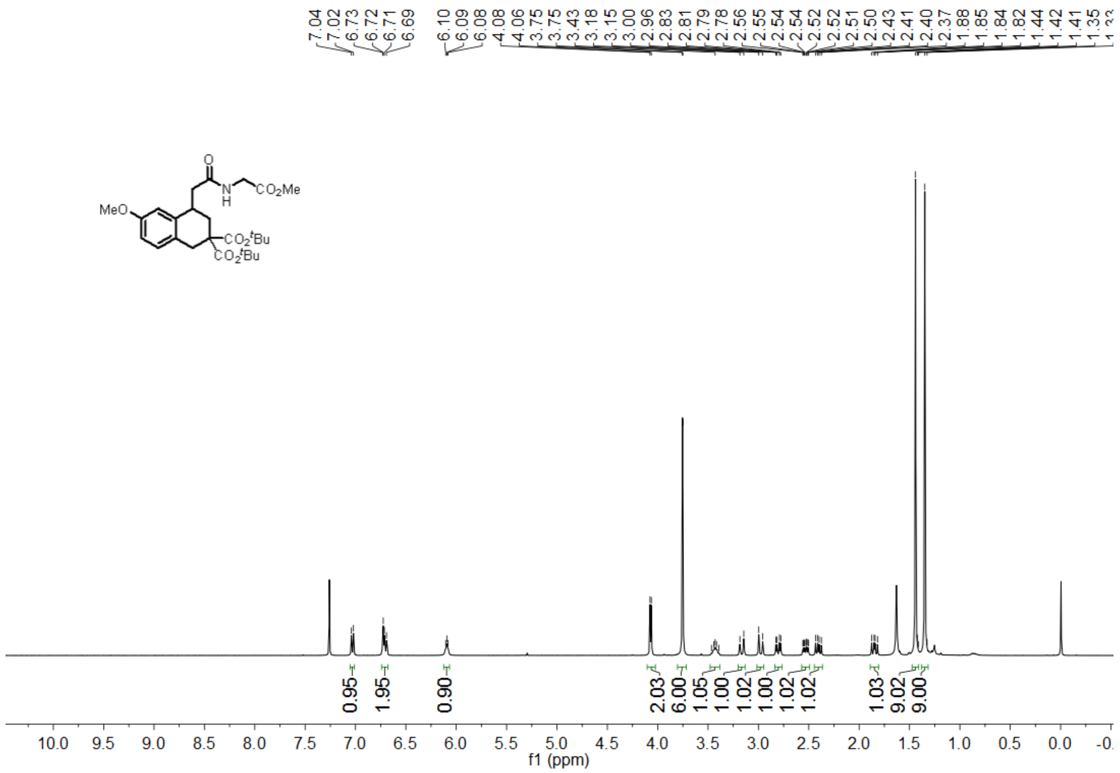

Supplementary Figure 112 ^{1}H NMR spectrum of **8**

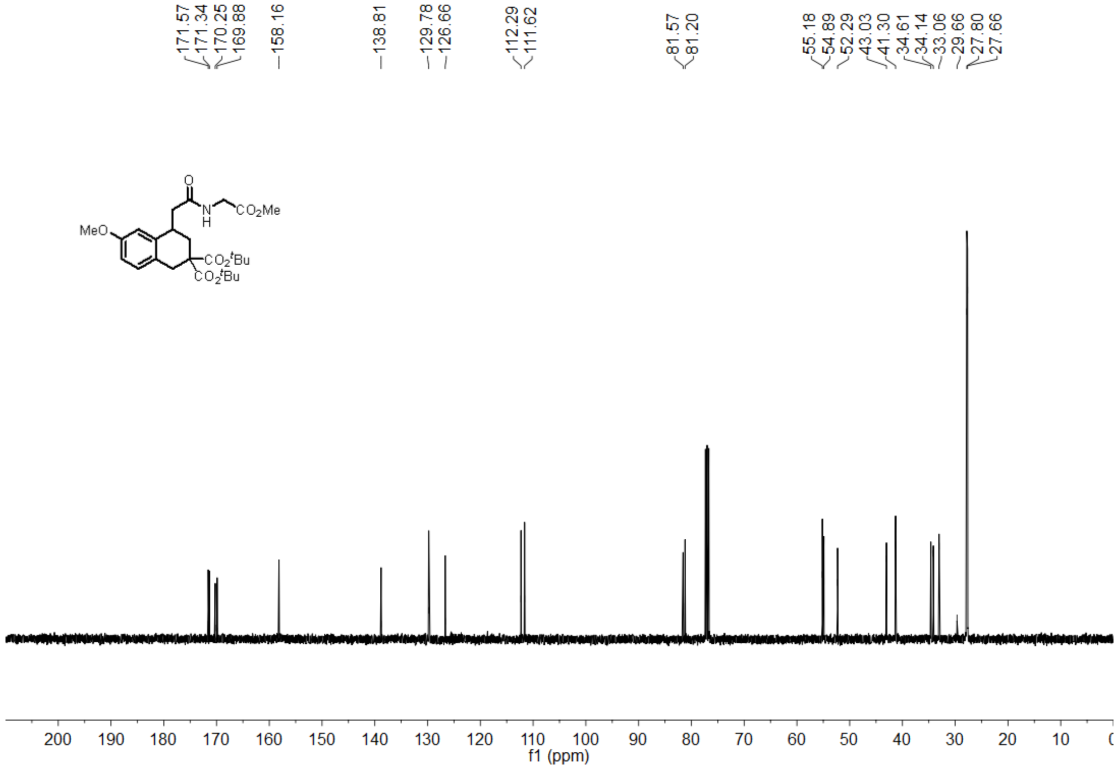

Supplementary Figure 113 ^{13}C NMR spectrum of **8**

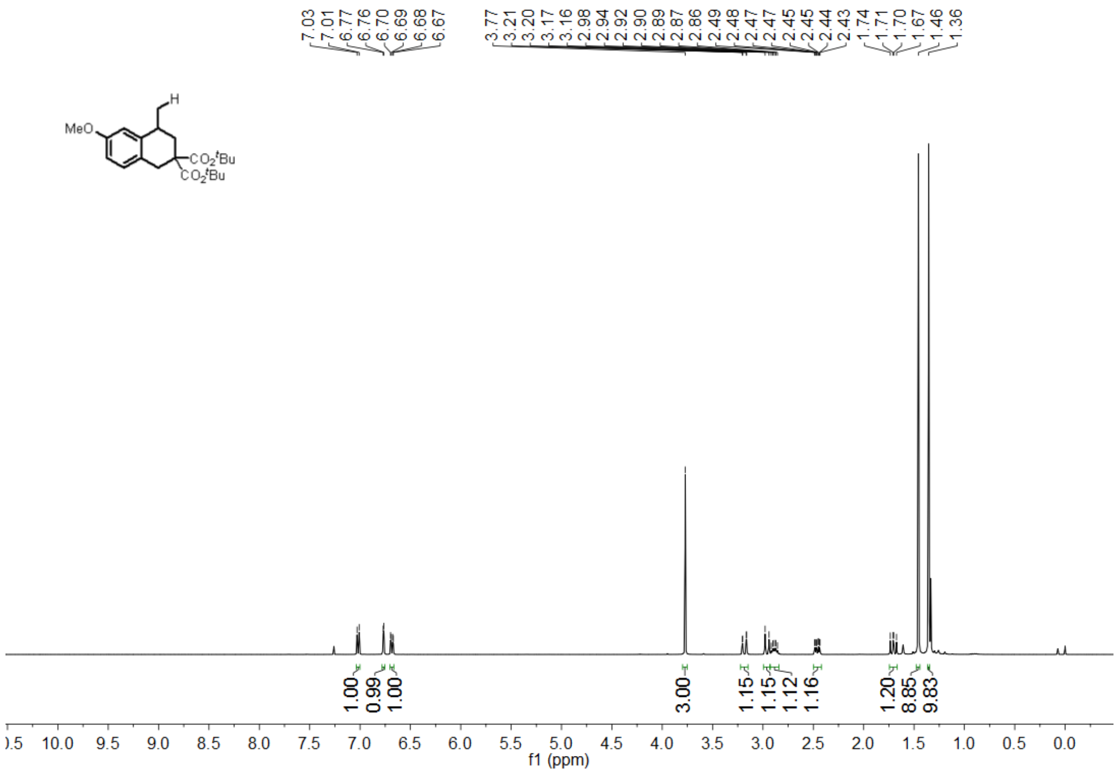

Supplementary Figure 114 ^{1}H NMR spectrum of **9**

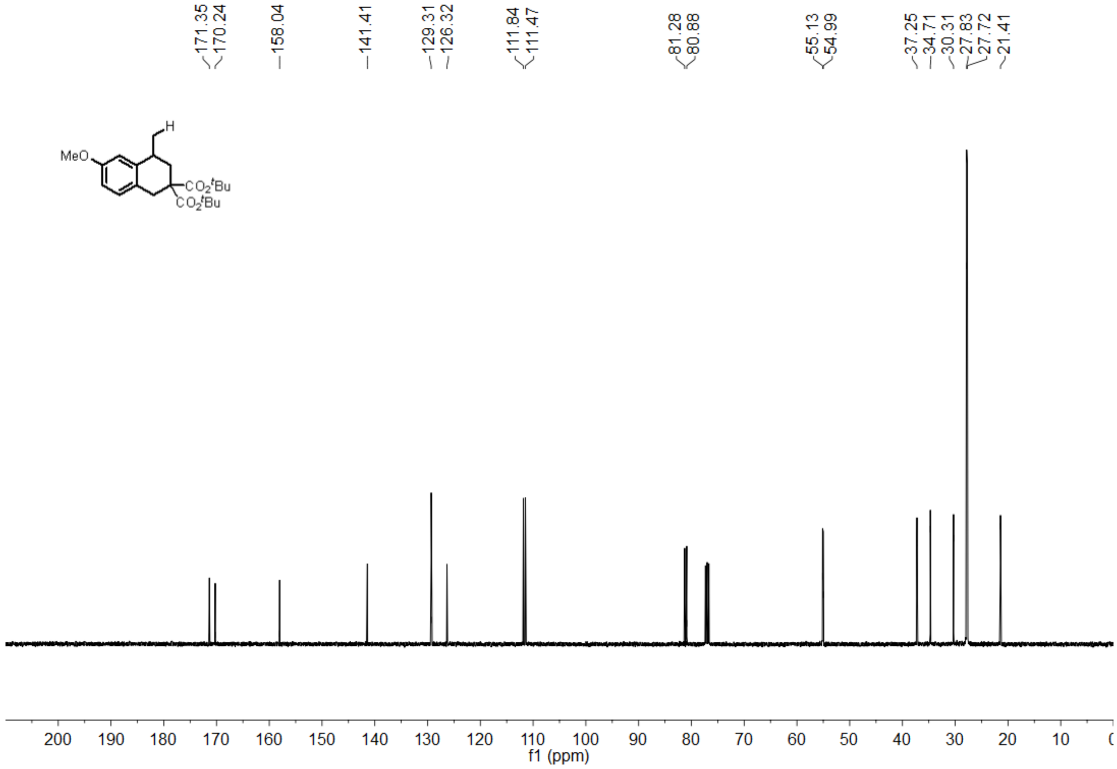

Supplementary Figure 115 ^{13}C NMR spectrum of **9**

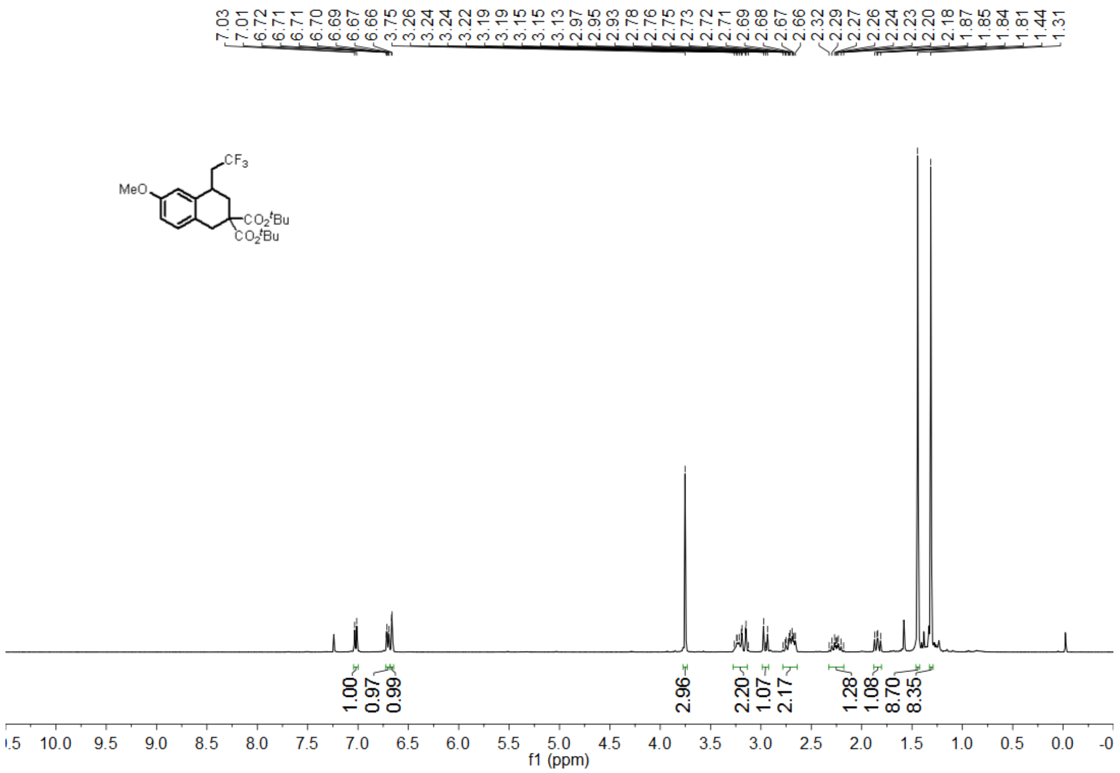

Supplementary Figure 116 ^{1}H NMR spectrum of **10**

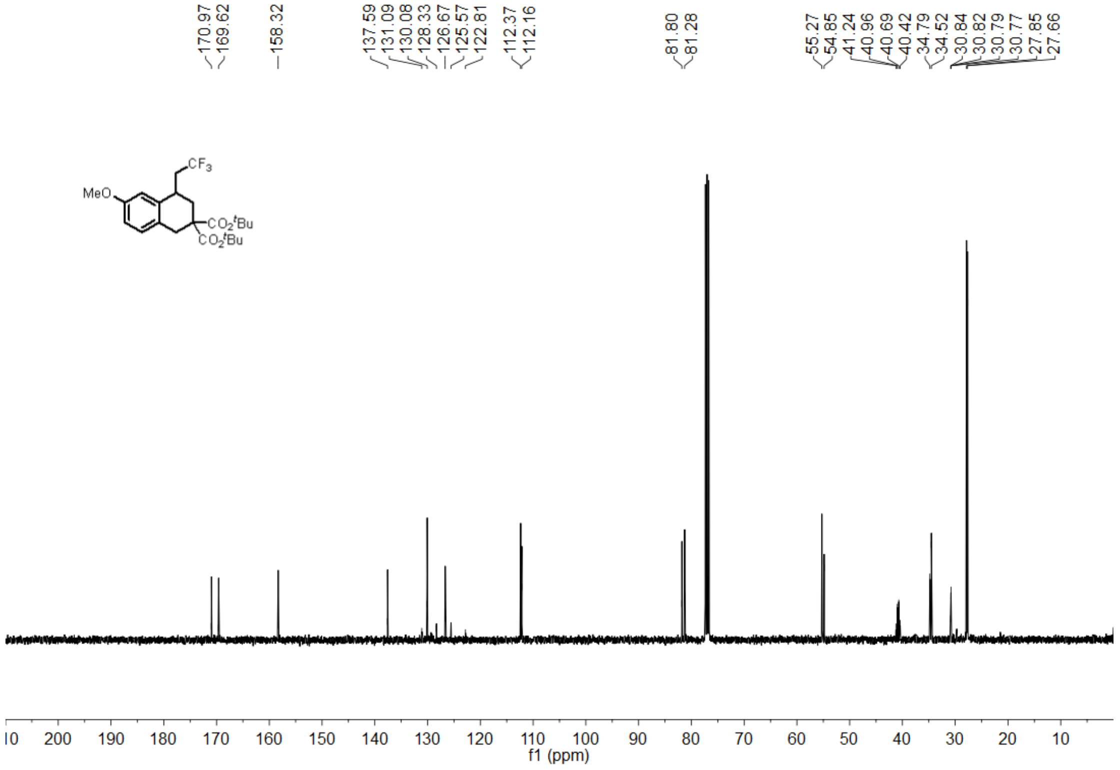

Supplementary Figure 117 ^{13}C NMR spectrum of **10**

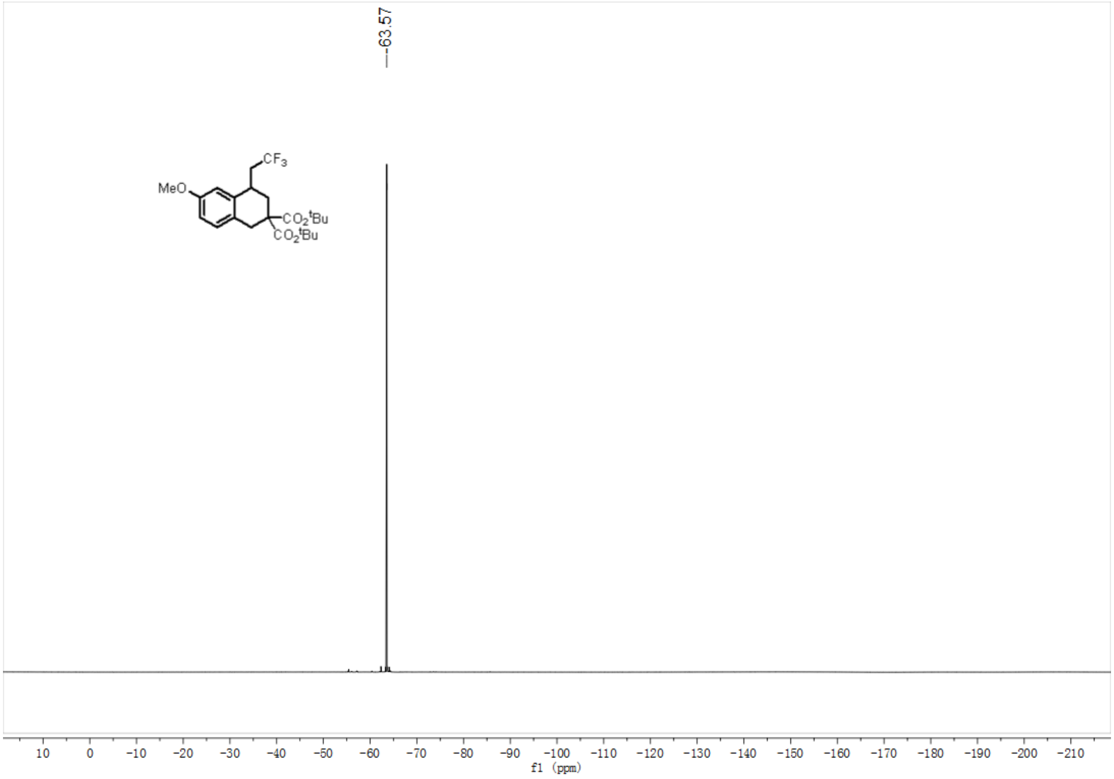

Supplementary Figure 118 ^{19}F NMR spectrum of **10**

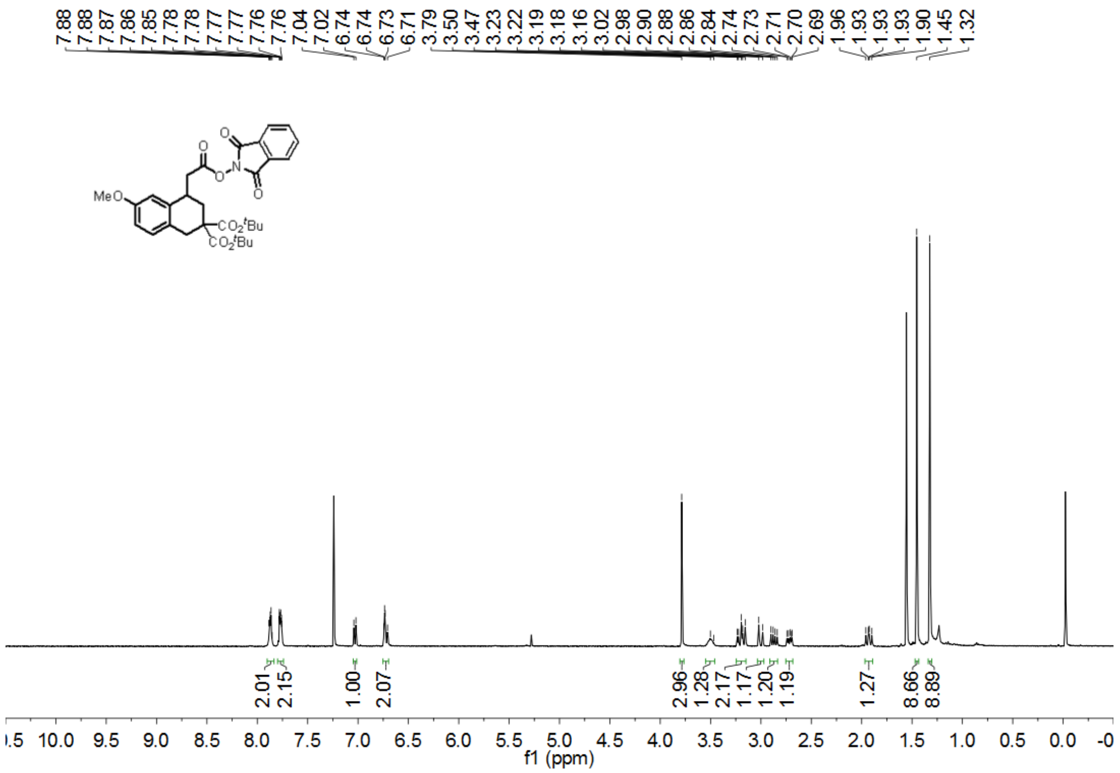

Supplementary Figure 119 ^{1}H NMR spectrum of **11**

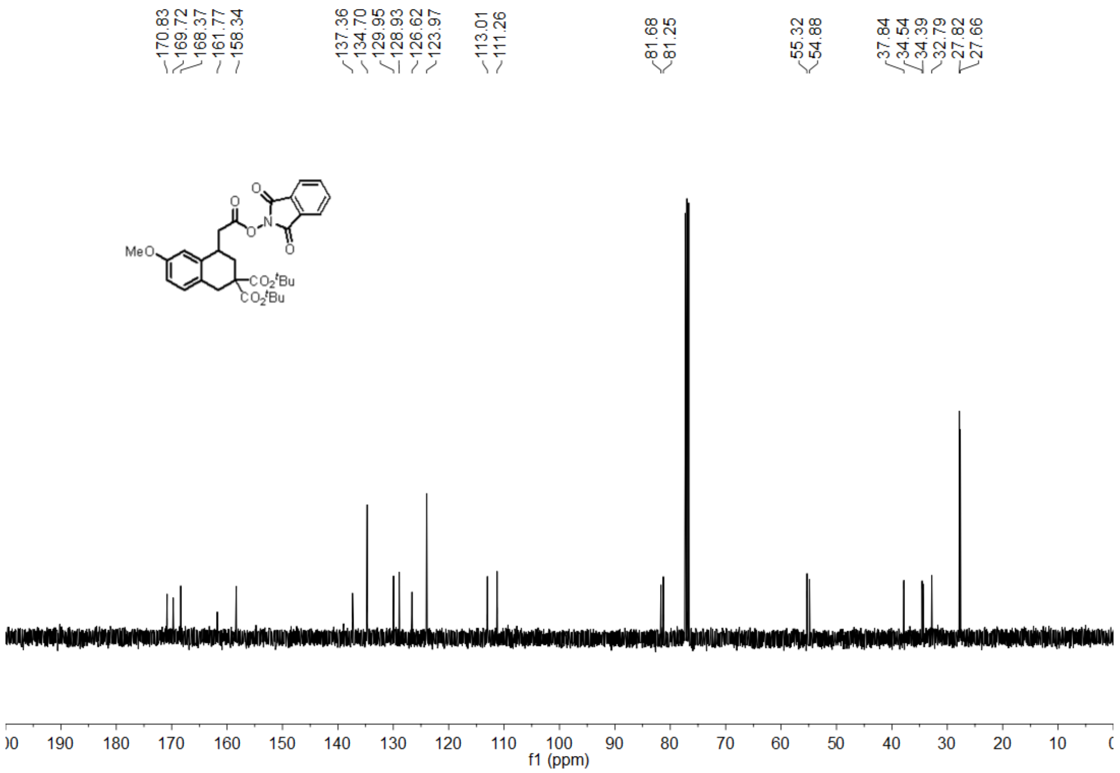

Supplementary Figure 120 ^{13}C NMR spectrum of **11**

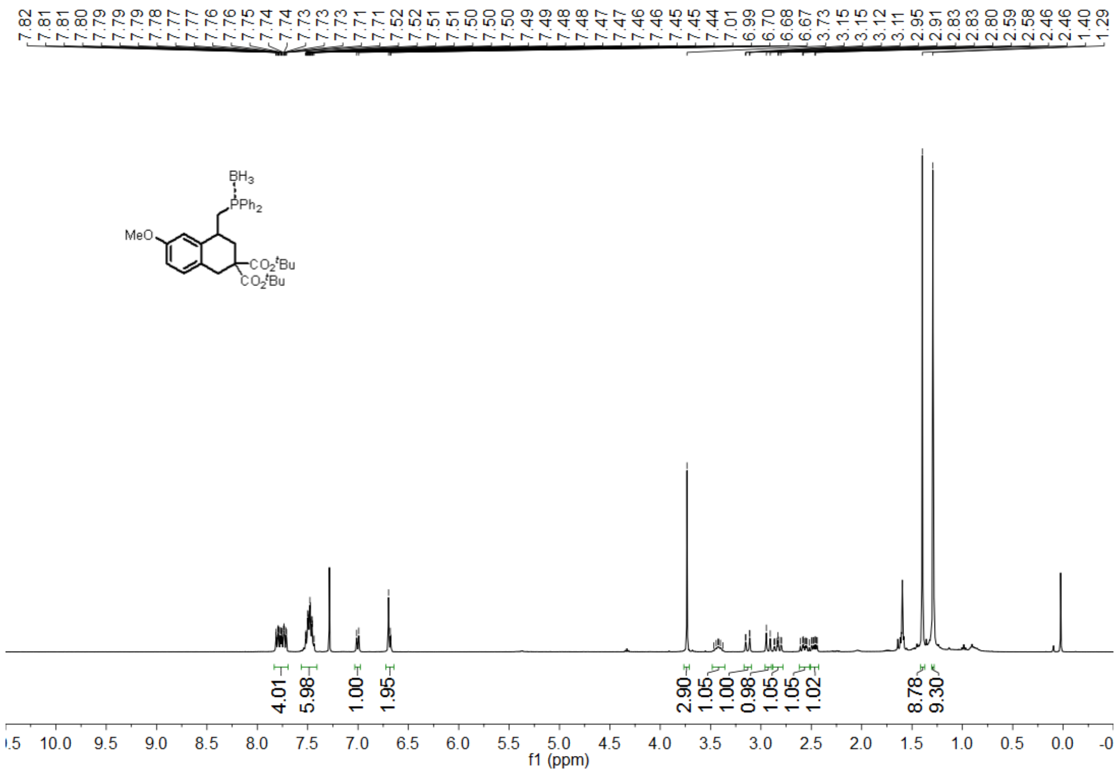

Supplementary Figure 121 ^{1}H NMR spectrum of **12**

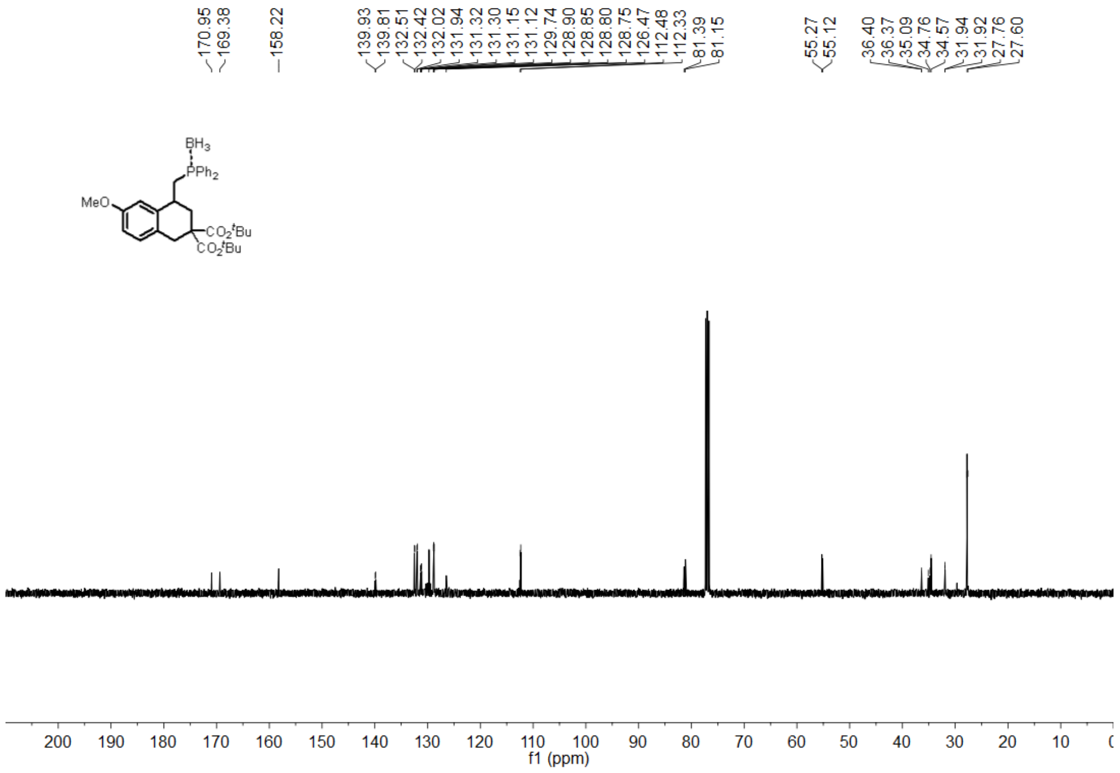

Supplementary Figure 122 ^{13}C NMR spectrum of **12**

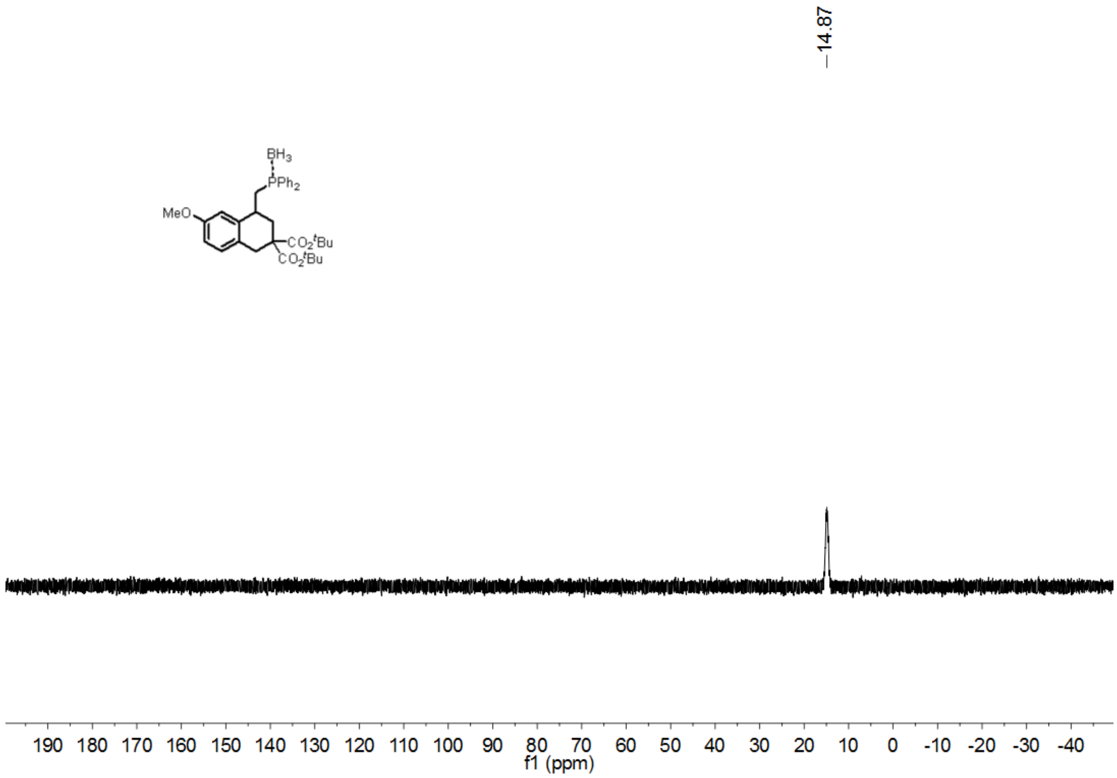

Supplementary Figure 123 ^{31}P NMR spectrum of **12**

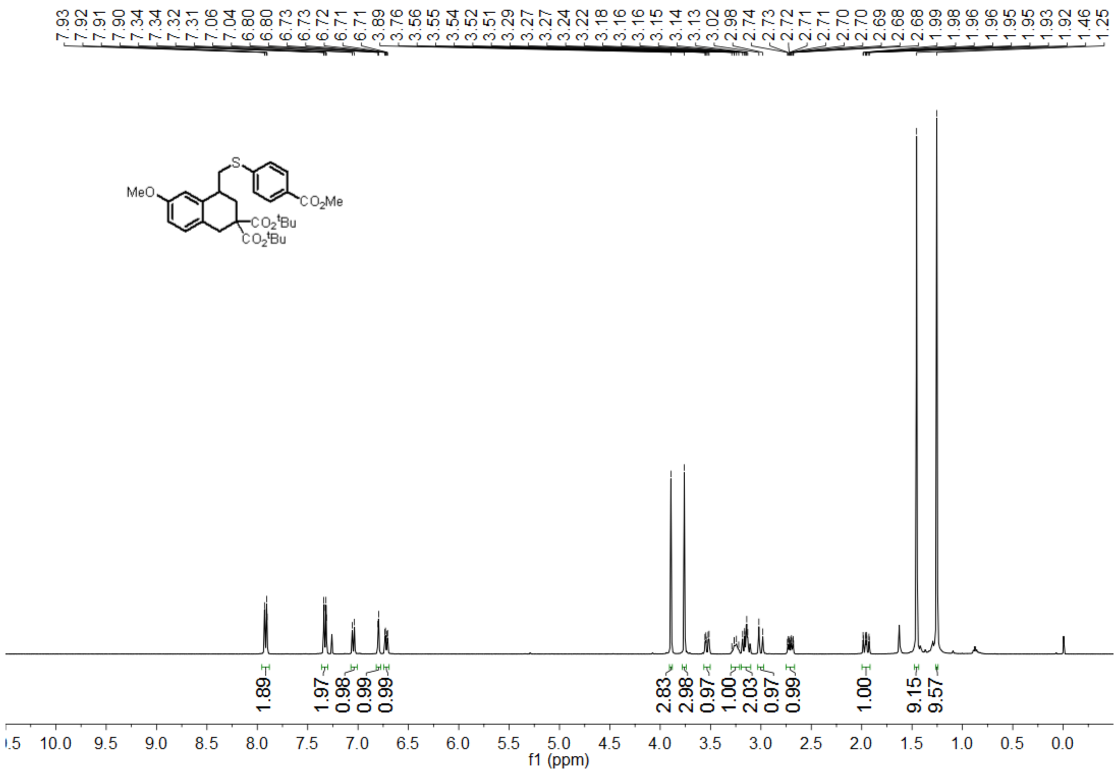

Supplementary Figure 124 ^{1}H NMR spectrum of **13**

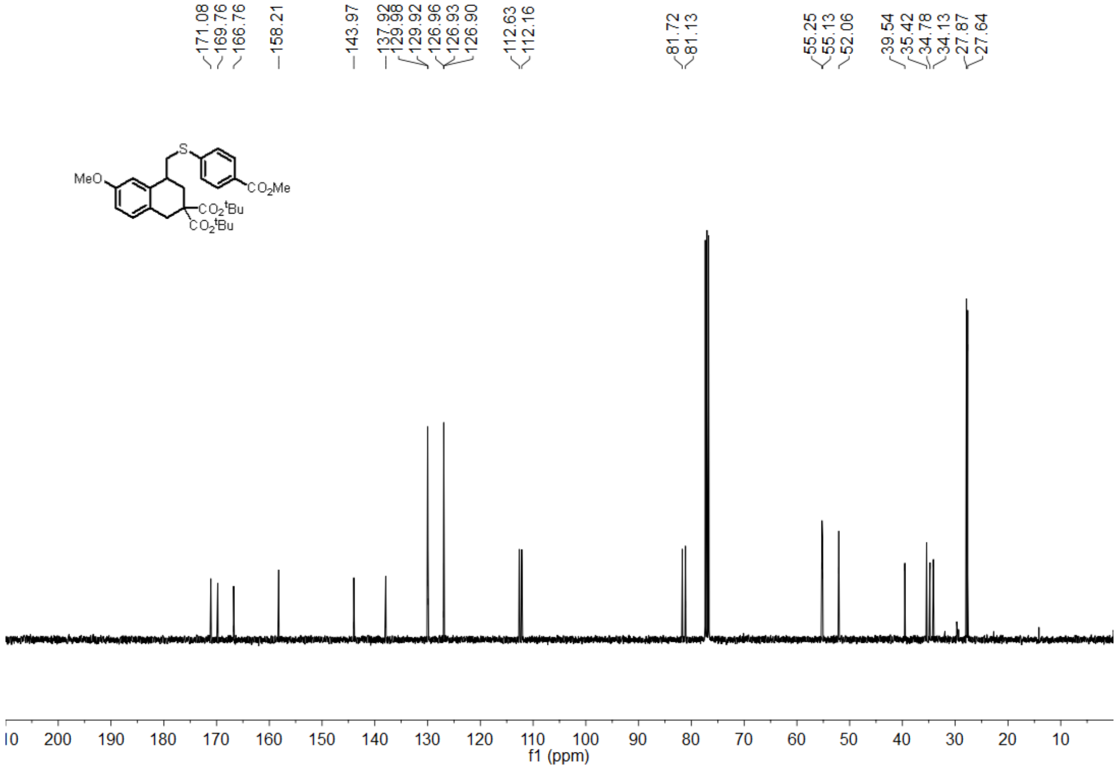

Supplementary Figure 125 ^{13}C NMR spectrum of **13**

4 Supplementary References

1. Xue, Y. et al. *Org. Biomol. Chem.* **20**, 989-994 (2022).
2. Bodkin, J. A., Bacskay, G. B. & Mcleod, M. D. *Org. Biomol. Chem.* **6**, 2544-2553 (2008).
3. Cao, Y. et al. *Green Chem*. **24**, 4789-4793 (2022).
4. Zhang, J.; Li, Y.; Zhang, F. Y.; Hu, C. C. & Chen, Y. Y. *Angew. Chem. Int. Ed.* **55**, 1872-1875 (2016).
5. Xu, J. et al. *J. Am. Chem. Soc.* **143**, 13266-13273 (2021).
6. Becke, A. D. Density-functional thermochemistry. III. The role of exact exchange. *J. Chem. Phys*. **98**, 5648-5652 (1993).
7. Lee, C., Yang, W. & Parr, R. G. Development of the Colle-Salvetti correlation-energy formula into a functional of the electron density. *Phys. Rev. B* **37**, 785-789 (1988).
8. Grimme, S., Antony, J., Ehrlich, S. & Krieg, H. A consistent and accurate ab initio parametrization of density functional dispersion correction (DFT-D) for the 94 elements H-Pu. *J. Chem. Phys.* **132**, 154104 (2010).
9. Grimme, S. Ehrlich, S. & Goerigk, L. Effect of the damping function in dispersion corrected density functional theory. *J. Comput. Chem.* **32**, 1456-1465 (2011).
10. Marenich, A. V., Cramer, C. J. & Truhlar, D. G. Universal solvation model based on solute electron density and on a continuum model of the solvent defined by the bulk dielectric constant and atomic surface tensions. *J. Phys. Chem. B* **113**, 6378-6396 (2009).
11. Zhao, Y. & Truhlar, D. G. The M06 suite of density functionals for main group thermochemistry, thermochemical kinetics, noncovalent interactions, excited states, and transition elements: two new functionals and systematic testing of four M06-class functionals and 12 other functionals. *Theor. Chem. Acc.* **120**, 215-241 (2007).
12. Legault, C.Y. CYLview, 1.0 b, Université de Sherbrooke, Quebec (Canada), 2009, (http://www.cylview.org)
