# Supplementary material for: Arylcarboxylation of unactivated alkenes with CO2 via visible-light photoredox catalysis
Source: Nat Commun. 2023 Jun 14;14:3529. doi: 10.1038/s41467-023-39240-8 (PMC10267151; doi:10.1038/s41467-023-39240-8)
Supplement: Supplementary file 3 — Description of Additional Supplementary Files [file 41467_2023_39240_MOESM3_ESM.docx]

**Description of Additional Supplementary Files**

**File Name:** Supplementary Data 1

**Description:** Energies of Stationary Points and Coordinates
