# Supplementary material for: Arylcarboxylation of unactivated alkenes with CO2 via visible-light photoredox catalysis
Source: Nat Commun. 2023 Jun 14;14:3529. doi: 10.1038/s41467-023-39240-8 (PMC10267151; doi:10.1038/s41467-023-39240-8)
Supplement: Supplementary file 4 — Supplementary Data 1 [file 41467_2023_39240_MOESM4_ESM.pdf]

Supplementary Data 1

Absolute Calculation Energies, Enthalpies, and Free Energies

Geometry	Thermal Correction to Free Energy	Thermal Correction to Enthalpy	Electronic Energy at basis set 6-311+g(d,p)	IF
**$[Ir]^{III}$**	0.421544	0.511303	-1539.956510	-
**$^{*}[Ir]^{III}$**	0.416603	0.508185	-1539.861989	-
**$[Ir]^{II}$**	0.418787	0.511773	-1539.766054	-
**CO_2**	-0.009243	0.015069	-188.560479	-
**$CO_2^{\cdot-}$**	-0.014913	0.012325	-188.621821	-
**1a**	0.443725	0.536341	-1233.263061	-
**CP1**	0.166532	0.214948	-787.006125	-
**CP2**	0.446975	0.550954	-1421.912050	-
**CP3**	0.457088	0.552154	-1421.928063	-
**CP4**	0.464521	0.565554	-1422.571926	-
**CP5**	0.446992	0.542678	-1421.379301	-
**CP6**	0.166242	0.215663	-786.847204	-
**CP7**	0.175368	0.225010	-787.474076	-
**ts1**	0.445927	0.548783	-1421.880176	232.77*i*
**ts2**	0.454686	0.549931	-1421.896789	334.85*i*
**ts3**	0.64999	0.776353	-2209.385332	1436.05*i*

B3LYP Geometries for all the optimized compounds and transition states

fac-$Ir^{III}(ppy)_3$

Ir	-0.00104300	-0.00042500	0.03629900
C	0.01342300	2.88772800	0.58228200
C	-1.35907500	1.74588300	2.20658100
C	-1.64080300	2.96532100	2.82877000
C	-1.09627200	4.15812500	2.33159100
C	-0.27121600	4.11459500	1.20894600
C	0.88213500	2.79028300	-0.59951400
C	1.82074700	1.30593600	-2.15636400
C	2.47281100	2.33265000	-2.82818700
C	2.31641400	3.63897600	-2.35330000
C	1.51938300	3.86676200	-1.23609100

H	-1.79122900	0.83656900	2.61642400
H	-2.28653300	2.98885800	3.70471800
H	0.14791500	5.04074500	0.82495100
H	1.90692300	0.27330800	-2.47663600
H	3.08584600	2.11004000	-3.69538700
H	1.39213300	4.87451200	-0.85732100
N	1.04846800	1.52570700	-1.07901500
C	-2.51366600	-1.42500100	0.58364800
C	-0.84760000	-2.02758500	2.22289200
C	-1.76825500	-2.87097600	2.85118700
C	-3.07209400	-2.99692900	2.35049700
C	-3.43983100	-2.27371300	1.21705400
C	-2.85504500	-0.63916400	-0.61057300
C	-2.02939800	0.89792200	-2.18031700
C	-3.23942200	0.93815400	-2.86223200
C	-4.29428600	0.15233200	-2.38656600
C	-4.10005500	-0.63767400	-1.25818900
H	0.15443600	-1.94554700	2.63615200
H	-1.17649900	1.48567900	-2.50201700
H	-3.34778300	1.56911300	-3.73829500
H	-4.90909200	-1.25180400	-0.87959000
N	-1.84088100	0.13376100	-1.09118800
H	-3.79065500	-3.65075200	2.83795300
C	-1.18379300	-1.27500000	1.07991500
C	1.98403100	-2.15400000	-0.59117500
C	0.24557000	-2.22002700	-2.16660800
C	0.82019800	-3.29101400	-2.83999500
C	2.02717800	-3.80703700	-2.35669700
C	2.60923100	-3.23635600	-1.22950300
C	2.48888900	-1.45690200	0.60031800
C	2.16873900	0.29409900	2.23022100
C	3.35859200	-0.07539200	2.86371900
C	4.12415700	-1.14145800	2.36982900

C	3.68641500	-1.82788200	1.23837000
H	-0.69012600	-1.77961300	-2.49358200
H	1.59333800	1.12144900	2.63769600
H	3.69250600	0.46754500	3.74605700
H	4.28161500	-2.65302100	0.85647100
H	2.50829400	-4.64484400	-2.85325300
H	5.04935200	-1.43166000	2.86099500
H	3.54422600	-3.62716200	-0.84420900
H	-5.25674600	0.15567000	-2.89018500
H	0.33161000	-3.70687000	-3.71502900
H	-4.45104700	-2.37351000	0.83118500
H	-1.47154700	-3.43260200	3.73512800
H	2.81097900	4.46989600	-2.84821200
H	-1.31320100	5.10789300	2.81353900
N	0.80838400	-1.66710600	-1.07886600
C	1.69021100	-0.37960100	1.08888600
C	-0.52501400	1.66066500	1.07408900

* *fac*-Ir^{III}(ppy)$_3$

Ir	0.01135900	-0.02699400	-0.05961100
C	-2.73681800	-0.88146200	-0.66967300
C	-1.98600100	0.71486000	-2.31992200
C	-3.21670700	0.61651400	-2.97655000
C	-4.21548300	-0.23517000	-2.48464200
C	-3.97412900	-0.98043700	-1.33166700
C	-2.43825800	-1.63699900	0.55659400
C	-0.80379500	-2.05444500	2.19602600
C	-1.63452200	-2.93178300	2.88032900
C	-2.91713300	-3.16193500	2.37081000
C	-3.31953700	-2.51307100	1.20820000
H	-1.22445400	1.37690600	-2.72449600
H	-3.39909000	1.20216800	-3.87540900

H	-4.75193200	-1.63790800	-0.95327200
H	0.20112700	-1.83141200	2.53741200
H	-1.28359300	-3.41970800	3.78351000
H	-4.31262400	-2.68184500	0.80792300
N	-1.19377000	-1.43001100	1.07232800
C	0.66433700	2.79859400	-0.56201400
C	1.86044700	1.36641900	-2.10033200
C	2.45598000	2.48814400	-2.68294400
C	2.16123900	3.77368500	-2.20875900
C	1.26851100	3.92461700	-1.14939500
C	-0.28051800	2.90541500	0.55876200
C	-1.63670500	1.68117300	2.03132800
C	-2.08885800	2.83379700	2.66150600
C	-1.60875100	4.06744100	2.21056500
C	-0.70304400	4.10278800	1.15559900
H	2.09712000	0.38002200	-2.48950300
H	-1.97418800	0.69780300	2.33899300
H	-2.79646400	2.76167800	3.48080100
H	-0.32599800	5.05252100	0.79444600
N	-0.75778300	1.71609200	1.01636500
H	2.62339000	4.64752300	-2.66021800
C	0.94870200	1.48535800	-1.03476200
C	2.62575200	-1.24866900	0.68194000
C	2.07545300	0.44694200	2.25590600
C	3.24910400	0.23833600	2.97368600
C	4.14833400	-0.77861500	2.52579800
C	3.83678500	-1.50276300	1.40081200
C	2.20372300	-1.90049200	-0.49553500
C	0.42564500	-2.17472100	-2.19864400
C	1.16767400	-3.18265400	-2.81992600
C	2.43793400	-3.54483300	-2.28284000
C	2.94889600	-2.93051000	-1.15799500
H	1.36156600	1.20546100	2.56435600

H	-0.53524200	-1.89009400	-2.61854700
H	0.79183500	-3.68345900	-3.70802300
H	3.91980900	-3.23319300	-0.77650300
H	5.06785100	-0.97514500	3.07015200
H	3.01764600	-4.32503000	-2.77182200
H	4.50768200	-2.27924700	1.04714300
H	-1.93819900	4.99176300	2.67630000
H	3.46105700	0.83896400	3.85178300
H	1.04695100	4.92272500	-0.78220100
H	3.15222600	2.36112400	-3.50937200
H	-3.59748600	-3.84080700	2.87694400
H	-5.17163600	-0.31593700	-2.99496100
N	1.74472300	-0.25709600	1.16838200
C	0.89348300	-1.49683500	-1.06000200
C	-1.70577300	-0.03022800	-1.16141400

fac-$Ir^{II}(ppy)_3$

Ir	0.00544100	-0.01062900	-0.07787200
C	0.68984000	2.80497400	-0.56712800
C	1.91292300	1.33910600	-2.06833600
C	2.50768800	2.45280100	-2.66375500
C	2.20324100	3.74502900	-2.21187900
C	1.30262500	3.91958100	-1.16249000
C	-0.24035600	2.91297200	0.56201200
C	-1.58512800	1.68661600	2.04883200
C	-2.02170500	2.84036300	2.68577500
C	-1.54138300	4.07338300	2.23263400
C	-0.64836800	4.10970400	1.16763600
H	2.15676800	0.34612500	-2.43323900
H	3.21017400	2.31665900	-3.48258700
H	1.08487300	4.92360100	-0.81090400
H	-1.92481000	0.70451300	2.35672900

H	-2.71961200	2.76999800	3.51320000
H	-0.26903000	5.05793200	0.80563200
N	-0.71785600	1.72332600	1.02325400
C	2.11033000	-1.99299600	-0.53801600
C	0.27455100	-2.28479600	-2.10905200
C	0.96488800	-3.33497600	-2.71241200
C	2.22521600	-3.72266000	-2.23136200
C	2.79222600	-3.05877900	-1.14404400
C	2.62853900	-1.26991900	0.62805500
C	2.18468000	0.47720400	2.13657400
C	3.38130600	0.26654300	2.80922600
C	4.22447700	-0.75724900	2.36478100
C	3.84701800	-1.52869800	1.27120100
H	-0.69655700	-1.99230300	-2.49563200
H	1.49177000	1.25597200	2.43442700
H	3.64200000	0.89064800	3.65722200
H	4.49017200	-2.32563800	0.91668800
N	1.82006900	-0.27251300	1.08377600
H	2.75949700	-4.54369800	-2.70106700
C	0.83634700	-1.57463800	-1.03001800
C	-2.43163800	-1.61728300	0.58160000
C	-0.74534300	-2.09414400	2.15221500
C	-1.55875700	-2.99260300	2.82828400
C	-2.85767600	-3.20304300	2.35227700
C	-3.29524700	-2.51382800	1.22715400
C	-2.76092500	-0.82563600	-0.60856300
C	-2.04098700	0.86216700	-2.19837800
C	-3.28545200	0.79334000	-2.82774500
C	-4.27375400	-0.07997500	-2.35033200
C	-4.01403400	-0.88211800	-1.24069800
H	0.27004800	-1.89121900	2.47294300
H	-1.28568500	1.54115000	-2.58279200
H	-3.48909200	1.41968900	-3.69309600

H	-4.78894300	-1.54721400	-0.87167800
H	-3.52229500	-3.89899100	2.85555300
H	-5.24248300	-0.13013500	-2.83961700
H	-4.30047900	-2.66519300	0.85126300
H	5.16769500	-0.95084600	2.86696100
H	-1.18102900	-3.51278700	3.70193400
H	3.76153900	-3.37801500	-0.77336300
H	0.52591500	-3.85522200	-3.56021200
H	-1.86145400	4.99696600	2.70579300
H	2.67089000	4.61044100	-2.67308700
N	-1.17129900	-1.42663300	1.06606100
C	-1.74385500	0.04486700	-1.09469500
C	0.97784700	1.48914700	-1.02877200

CO_2

C	1.18736400	-0.18518500	0.00000000
O	2.35696700	-0.18518500	0.00000000
O	0.01776100	-0.18518500	0.00000000

CO_2 radical anion

C	1.18736400	-0.18518500	-0.13799700
O	2.33778300	-0.18518500	0.32659900
O	0.03694500	-0.18518500	0.32659900

1a

C	-0.64901600	3.92894000	-1.86770200
C	0.56117800	3.46651800	-1.32634400
C	1.76779100	4.03217700	-1.72851700
C	1.81121300	5.06792900	-2.67799500
C	0.59333400	5.52821700	-3.19072200

C	-0.63122000	4.97339100	-2.80081900
C	3.12283800	5.65746900	-3.14408200
C	3.75718500	4.91586600	-4.35616300
C	4.24073100	3.48685200	-3.98277900
C	5.38228700	3.47454300	-3.00307500
C	5.38574900	2.80451200	-1.84578200
O	-1.78276500	3.31120500	-1.42326000
C	-3.03554700	3.75143400	-1.95151900
C	2.73497400	4.91865900	-5.50735300
O	2.38623100	5.95752900	-6.04557900
O	2.29205100	3.70130000	-5.80384300
C	1.32247600	3.44279900	-6.90245300
C	1.92543500	3.89509000	-8.23090800
C	-0.00877000	4.12352800	-6.59338200
C	1.17685600	1.92426700	-6.86036800
C	4.96387500	5.72581800	-4.87009200
O	5.42411700	6.70602900	-4.31526000
O	5.43315900	5.16683100	-5.98955300
C	6.59936900	5.70661700	-6.73523400
C	6.70201200	4.75393700	-7.92313000
C	7.84830600	5.62289700	-5.86027600
C	6.29848700	7.12862600	-7.20434600
H	0.53975800	2.66650100	-0.59131800
H	2.69356500	3.66682400	-1.29408500
H	0.59048400	6.33612800	-3.91732600
H	-1.54899500	5.35976200	-3.22893900
H	2.97661900	6.70210300	-3.42949700
H	3.85461100	5.64284900	-2.33121600
H	4.55703700	2.98714100	-4.90444500
H	3.39643100	2.92011800	-3.58283400
H	6.27591400	4.03378200	-3.28554100
H	6.25493900	2.80648100	-1.19107700
H	4.52468700	2.22465200	-1.51737800

H	-3.79442900	3.12949100	-1.47312900
H	-3.21972900	4.80511000	-1.70862400
H	-3.07832800	3.61076300	-3.03849400
H	2.91849200	3.45339200	-8.37184400
H	1.28059800	3.55211800	-9.04798600
H	2.01009500	4.98213400	-8.28924300
H	0.07913700	5.21170800	-6.60843900
H	-0.38357900	3.81090000	-5.61338900
H	-0.74116800	3.82325400	-7.35166900
H	0.79820900	1.59760200	-5.88553600
H	0.47061200	1.60147500	-7.63268900
H	2.13988500	1.43528500	-7.04456100
H	5.78730400	4.78361000	-8.52542000
H	7.54497000	5.04728400	-8.55777800
H	6.86486100	3.72501100	-7.58369300
H	7.78944800	6.29911800	-5.00459500
H	8.72228000	5.89948200	-6.46100400
H	7.99555000	4.59959200	-5.49682200
H	7.09330800	7.45270600	-7.88578900
H	5.34828900	7.15974900	-7.74933900
H	6.25139900	7.83055000	-6.36911800

CP1

C	-2.23504800	-0.92959600	0.31374100
C	-0.86530500	-1.09560300	0.11130000
C	-0.05332600	-0.06393300	-0.39777800
C	-0.70038000	1.14623800	-0.69177300
C	-2.07480500	1.31909500	-0.49079900
C	-2.89502600	0.28866700	0.01939500
H	-2.81629700	-1.75958500	0.70965500
H	-0.42190700	-2.05767700	0.35850500
H	-0.13527400	1.98471000	-1.08754100

H	-2.52588000	2.27836900	-0.73556400
S	-4.63561200	0.50117100	0.27611000
C	1.45044300	-0.29133400	-0.60452800
C	2.15683300	0.95322200	-1.16683000
H	2.06784900	1.81294000	-0.49178200
H	3.22507100	0.74096000	-1.29619200
H	1.75459400	1.24389900	-2.14481400
C	2.11101700	-0.65089100	0.74466300
H	1.68049500	-1.56193700	1.17521200
H	3.18765000	-0.81915100	0.61041200
H	1.98145600	0.16081200	1.47155600
C	1.66743900	-1.45635800	-1.59528700
H	1.21542000	-1.23017600	-2.56921600
H	2.74015300	-1.63170000	-1.75022300
H	1.22536100	-2.38848300	-1.22590800

CP2

C	-0.69031800	4.08428100	-1.85364500
C	0.54541900	3.78463100	-1.25731700
C	1.70702600	4.40363300	-1.70927300
C	1.67889300	5.33429900	-2.76290200
C	0.43687000	5.62983500	-3.33430200
C	-0.74412300	5.01834100	-2.89589200
C	2.94720200	5.97300200	-3.27850200
C	3.66019600	5.14172700	-4.38369900
C	4.13604300	3.76438700	-3.85901500
C	5.13429800	3.84324500	-2.75014900
C	5.51728300	2.61379500	-2.00392700
O	-1.77619000	3.42591300	-1.35085500
C	-3.05245400	3.69633500	-1.93370200
C	2.70642300	5.03568200	-5.58669200
O	2.36497900	6.02275500	-6.22024800

O	2.30459500	3.78949200	-5.81684300
C	1.39958400	3.43025000	-6.93974400
C	2.06227900	3.79510400	-8.26681400
C	0.04221100	4.10381400	-6.74998500
C	1.27787800	1.91599400	-6.79180600
C	4.89041800	5.91890900	-4.88729900
O	5.31267600	6.94550800	-4.38731800
O	5.42997500	5.27773200	-5.92877300
C	6.66887200	5.73568300	-6.60490300
C	6.86071600	4.68258100	-7.69311400
C	7.83028800	5.70987300	-5.61303900
C	6.44319400	7.11602800	-7.21869500
H	0.57817400	3.06637500	-0.44256100
H	2.65653300	4.16052400	-1.23868600
H	0.37981400	6.35095200	-4.14542400
H	-1.68380600	5.27716600	-3.37029000
H	2.72734000	6.96332200	-3.68600700
H	3.66028500	6.10724900	-2.46067300
H	4.55379100	3.19877300	-4.70539000
H	3.26119300	3.19005200	-3.53326200
H	5.81847400	4.69004800	-2.71481000
H	4.63422000	1.98797100	-1.82027900
H	-3.76373900	3.06719300	-1.39534100
H	-3.32931900	4.75053600	-1.80987100
H	-3.06531900	3.43469500	-2.99891900
H	3.06799500	3.36345300	-8.32469300
H	1.46540300	3.38079100	-9.08734100
H	2.13307300	4.87644700	-8.40112500
H	0.11110300	5.18891600	-6.84958800
H	-0.37274900	3.86284000	-5.76573000
H	-0.64796100	3.72732100	-7.51385900
H	0.85229800	1.65441300	-5.81668600
H	0.62070800	1.52159100	-7.57419500

H	2.25793100	1.43545200	-6.88639100
H	6.00369000	4.66853900	-8.37562400
H	7.76183700	4.91148500	-8.27201400
H	6.97466200	3.68570700	-7.25311700
H	7.71696300	6.46982300	-4.83681000
H	8.76442000	5.90200500	-6.15316200
H	7.90694000	4.72552300	-5.13761400
H	7.30782800	7.37483000	-7.84059500
H	5.55292600	7.11045300	-7.85792100
H	6.32453300	7.88547200	-6.45286900
H	5.97969000	2.87307300	-1.04409900
C	6.55555600	1.76712300	-2.83513100
O	6.06949900	0.91982400	-3.63393500
O	7.77390300	2.03758300	-2.64503300

CP3

C	3.53998000	-1.76677400	-1.78659200
C	2.15364800	-1.94183500	-1.58897800
C	1.55964000	-1.70931300	-0.37215300
C	2.35632600	-1.20188900	0.79555000
C	3.83136400	-1.09389100	0.53654900
C	4.36497900	-1.35058400	-0.69598700
C	0.09991000	-1.93041000	-0.11392400
C	-0.59474700	-0.66659400	0.47180800
C	0.24571800	-0.07477900	1.62830900
C	1.73548900	0.14491700	1.31919900
C	2.02021500	1.33677700	0.40087800
C	-1.96839600	-1.10703100	1.01812600
O	-2.23060400	-1.24362800	2.19914200
O	-2.80285200	-1.35719700	0.00394900
C	-4.20503600	-1.80157900	0.20310900
C	-4.72981700	-1.89069700	-1.22754500

C	-4.97534400	-0.74808900	0.99738400
C	-4.21624400	-3.17300200	0.87510900
C	-0.88832100	0.34957700	-0.64680100
O	-0.65497000	0.16864700	-1.82776300
O	-1.47580700	1.43258500	-0.12756800
C	-2.00877400	2.53532600	-0.96639700
C	-2.51636400	3.52551500	0.07902900
C	-3.16010400	2.00142200	-1.81643200
C	-0.89449200	3.16023400	-1.80420100
C	1.79530500	2.73787200	1.02653900
O	1.81907500	3.70653500	0.20932900
O	1.65486300	2.81856900	2.27987300
O	5.69624000	-1.24516100	-1.01464000
C	6.58668600	-0.81306300	0.01157700
H	3.99796000	-1.95428700	-2.75275700
H	1.54260800	-2.27392100	-2.42618500
H	4.44928400	-0.76797600	1.36675900
H	-0.01127300	-2.72911500	0.63604200
H	-0.41558400	-2.25172200	-1.02125300
H	0.16527000	-0.78114100	2.46148200
H	-0.19941800	0.86512500	1.96142600
H	2.22084500	0.36339400	2.27707200
H	1.45462100	1.27948900	-0.53431900
H	3.07660500	1.30537100	0.09979100
H	-4.14621300	-2.61089100	-1.81175700
H	-5.77456600	-2.21907100	-1.21622400
H	-4.67686300	-0.91458600	-1.72151000
H	-4.63545200	-0.69152400	2.03356400
H	-6.03950300	-1.01027800	0.99467700
H	-4.86615400	0.23829000	0.53340700
H	-5.24330300	-3.55539600	0.88903400
H	-3.59661200	-3.88120800	0.31308600
H	-3.85209200	-3.12179000	1.90363100

H	-1.69293100	3.87770700	0.71003100
H	-2.96677100	4.39062900	-0.41953400
H	-3.27456500	3.06074100	0.71926200
H	-2.81297300	1.26241400	-2.54253000
H	-3.61675500	2.83487100	-2.36284800
H	-3.92877000	1.54456100	-1.18435100
H	-1.27082200	4.09190600	-2.24447800
H	-0.02586700	3.39638100	-1.17866300
H	-0.58148700	2.49881100	-2.61502200
H	7.57882400	-0.78474600	-0.44373500
H	6.31992100	0.18836400	0.37258200
H	6.59104600	-1.51514400	0.85525200
H	2.19995700	-1.90609900	1.63614600

CP4

C	-0.61177700	4.08190300	-1.78746300
C	0.63009500	3.77041000	-1.21069100
C	1.79229500	4.36706900	-1.69061900
C	1.75885100	5.28756100	-2.75312100
C	0.51039700	5.59681700	-3.30326300
C	-0.67102300	5.00668600	-2.83773600
C	3.02663100	5.90297000	-3.29825500
C	3.68986200	5.07206900	-4.43659900
C	4.12152600	3.65909400	-3.97185700
C	5.23859700	3.64399100	-2.92288900
C	5.57083700	2.21356000	-2.46895500
O	-1.69733900	3.44399900	-1.25852700
C	-2.97948200	3.72046300	-1.82542600
C	2.70679400	5.04455100	-5.62216900
O	2.39377200	6.06496400	-6.21638700
O	2.24769500	3.82446100	-5.88437800
C	1.30932700	3.54256300	-7.00199900

C	1.96619100	3.92171500	-8.32801900
C	-0.01437600	4.26767600	-6.76842900
C	1.12280400	2.03099800	-6.90128800
C	4.93526800	5.82153400	-4.94464800
O	5.41339400	6.80821000	-4.41618100
O	5.41914700	5.20353500	-6.02744800
C	6.63423400	5.66314500	-6.74446600
C	6.74275900	4.65941200	-7.88942200
C	7.84429300	5.56292300	-5.81783300
C	6.41473700	7.07627700	-7.28151900
H	0.66750000	3.06070500	-0.38871600
H	2.74463200	4.11390900	-1.23207900
H	0.44795600	6.31212400	-4.11909700
H	-1.61543400	5.27541200	-3.29694200
H	2.81620900	6.90097700	-3.69209000
H	3.76108500	6.02323700	-2.49878200
H	4.44656800	3.08960000	-4.84712600
H	3.24035100	3.14710900	-3.57406600
H	6.14641400	4.10672300	-3.33332600
H	5.86525600	1.61789100	-3.34236100
H	-3.68878200	3.10497800	-1.26894800
H	-3.24433700	4.77868400	-1.71028000
H	-3.01067000	3.44617200	-2.88709200
H	2.95091300	3.44896600	-8.41719700
H	1.33881600	3.56119900	-9.15117200
H	2.08248400	5.00277000	-8.42835900
H	0.10048200	5.35142600	-6.83414900
H	-0.42480100	4.01259300	-5.78590900
H	-0.73163600	3.94670600	-7.53271500
H	0.70294400	1.75604700	-5.92736900
H	0.43520400	1.69225400	-7.68358600
H	2.07858300	1.51110400	-7.03027200
H	5.85422900	4.70163700	-8.52909500

H	7.62161000	4.89160300	-8.50036100
H	6.84836300	3.63955100	-7.50322500
H	7.78206000	6.27475800	-4.99183000
H	8.75208600	5.77778800	-6.39327400
H	7.93225000	4.55034300	-5.40831900
H	7.24825000	7.33890900	-7.94295700
H	5.48801800	7.12494000	-7.86453300
H	6.36590500	7.81290500	-6.47684800
H	4.67011300	1.76103300	-2.03387000
C	6.70951200	2.20806900	-1.42493600
O	7.88788900	2.05273800	-1.86372200
O	6.37349400	2.39042900	-0.21697000
H	4.95035600	4.23567400	-2.04667500

CP5

C	-4.54111400	0.38043900	-1.93413500
C	-3.35887600	-0.34980800	-1.91614200
C	-2.13310000	0.23044700	-1.54407100
C	-2.11333500	1.58363400	-1.17626300
C	-3.30796100	2.32991500	-1.19945000
C	-4.51518100	1.73760700	-1.57388600
C	-0.88205700	-0.62114100	-1.60568600
C	0.30563200	-0.04792500	-0.80826800
C	0.41540600	1.46244300	-1.09510900
C	-0.83656800	2.26852600	-0.71223100
C	-0.90609000	2.61725200	0.79245600
C	1.58394200	-0.76245400	-1.28147700
O	2.45508400	-0.24556600	-1.95669500
O	1.56827000	-2.03475900	-0.86948100
C	2.67288400	-2.98678700	-1.14658400
C	2.20175900	-4.25499800	-0.43955500
C	3.96827600	-2.46908100	-0.52400200

C	2.78982200	-3.21308800	-2.65244200
C	0.16536500	-0.35665100	0.69229300
O	-0.78287000	-0.92735100	1.19849000
O	1.25622200	0.06993600	1.33974600
C	1.49652300	-0.20206000	2.77858900
C	2.86685500	0.42914300	3.01097500
C	1.55024100	-1.71128400	3.01030500
C	0.43212900	0.48616600	3.63064200
C	-1.74894200	3.87212000	1.14030700
O	-2.38197300	3.83529000	2.23535500
O	-1.69951900	4.84448500	0.33187400
O	-5.71364200	2.39684200	-1.61887400
C	-5.73123800	3.77293300	-1.23676800
H	-5.48245500	-0.08026100	-2.22105800
H	-3.38141600	-1.40111100	-2.19727500
H	-3.26263500	3.37484800	-0.91721700
H	-0.56416300	-0.70746200	-2.65465600
H	-1.10386000	-1.63474800	-1.26292500
H	0.58783200	1.55945600	-2.17275700
H	1.29295200	1.87779100	-0.59251400
H	-0.76367000	3.23251700	-1.22934500
H	0.11364000	2.82694400	1.14555600
H	-1.27781900	1.77305700	1.37728700
H	1.24957400	-4.60179300	-0.85630500
H	2.94647600	-5.04735300	-0.57015800
H	2.06985200	-4.07486700	0.63284600
H	4.33165800	-1.57195300	-1.02970800
H	4.73592800	-3.24727800	-0.60330100
H	3.81992100	-2.24307100	0.53753900
H	3.50270900	-4.02485600	-2.83706200
H	1.82141100	-3.50813700	-3.07260900
H	3.14276800	-2.31808900	-3.16952000
H	2.84114200	1.50321800	2.79607300

H	3.16322800	0.29195600	4.05646700
H	3.62366200	-0.03826700	2.37134100
H	0.57734200	-2.18097600	2.84976300
H	1.86020300	-1.90095800	4.04433300
H	2.28360800	-2.17642800	2.34315600
H	0.69470000	0.36479700	4.68797700
H	0.39322200	1.55772100	3.41004200
H	-0.55771100	0.05495600	3.46787000
H	-6.77005500	4.09502500	-1.33277100
H	-5.40215300	3.90073300	-0.19831200
H	-5.09844400	4.37729100	-1.89856900

CP6

C	-2.21756700	-0.95315900	0.31871600
C	-0.85889300	-1.11187700	0.11605800
C	-0.05742700	-0.06354300	-0.39698800
C	-0.69317700	1.15855900	-0.69761800
C	-2.05552800	1.33348600	-0.49984900
C	-2.86068700	0.28114100	0.01523500
H	-2.81011500	-1.77303700	0.71345100
H	-0.40444800	-2.06718400	0.35913900
H	-0.11823200	1.98792300	-1.09224300
H	-2.52099700	2.28484200	-0.73947000
S	-4.55138900	0.49134300	0.26300500
C	1.44053500	-0.28631900	-0.60424000
C	2.14821500	0.95690900	-1.16619300
H	2.06276200	1.81605500	-0.49052200
H	3.21446500	0.73751800	-1.29276400
H	1.74917200	1.24660800	-2.14538000
C	2.09004900	-0.64711900	0.75162600
H	1.66779300	-1.56392000	1.17631500
H	3.16680400	-0.80418600	0.61367000

H	1.95282400	0.16223200	1.47882300
C	1.64477800	-1.45485200	-1.59585400
H	1.18546100	-1.22959600	-2.56590200
H	2.71749900	-1.61915100	-1.75516500
H	1.21302200	-2.38901400	-1.22153400

CP7

C	-2.23247300	-0.93213200	0.31439100
C	-0.86345900	-1.09635800	0.11142700
C	-0.05684500	-0.06186800	-0.39772700
C	-0.69224200	1.15178400	-0.69519400
C	-2.06556800	1.33440300	-0.49774400
C	-2.84662600	0.29163100	0.00939600
H	-2.81852700	-1.75814900	0.70993900
H	-0.42103600	-2.05712800	0.35842100
H	-0.12346900	1.98618200	-1.09032700
H	-2.51592700	2.29295900	-0.74245300
S	-4.60657700	0.44977200	0.28856300
H	-4.71992600	1.72833900	-0.13029000
C	1.44653300	-0.29062200	-0.60401300
C	2.15340500	0.95338400	-1.16614500
H	2.06618300	1.81293600	-0.49079300
H	3.22071100	0.73790000	-1.29430100
H	1.75267500	1.24373500	-2.14474300
C	2.10117200	-0.64996300	0.74788400
H	1.67360100	-1.56339200	1.17585400
H	3.17797500	-0.81376600	0.61385500
H	1.96758900	0.16083100	1.47476500
C	1.65687600	-1.45629300	-1.59511400
H	1.20108800	-1.23019900	-2.56708700
H	2.72914100	-1.62830000	-1.75309600
H	1.21864500	-2.38911900	-1.22347200

ts1

C	-4.68972900	1.23662100	-0.15525500
C	-3.73620600	2.24870200	0.04183900
C	-2.45733700	2.11665700	-0.49127100
C	-2.08661600	0.98080900	-1.23265700
C	-3.05481800	-0.00897100	-1.43062700
C	-4.34689700	0.10291800	-0.90265000
C	-0.68609900	0.82037600	-1.77555100
C	0.30854200	0.17808500	-0.76526600
C	0.53720800	1.06248000	0.48177300
C	1.21727200	2.37533600	0.17827000
C	1.59905700	3.23875200	1.16833200
O	-5.91551600	1.44678400	0.40929900
C	-6.91041600	0.43406700	0.24681000
C	-0.21069800	-1.22953100	-0.42306900
O	-0.24983200	-2.11862000	-1.25999900
O	-0.61305600	-1.33659800	0.83980500
C	-1.12316400	-2.60820600	1.41566300
C	-0.03384000	-3.67652700	1.33891700
C	-2.41046400	-3.02315500	0.70655500
C	-1.40451100	-2.22297300	2.86557700
C	1.66714200	-0.03775100	-1.46107300
O	1.92363500	0.29942000	-2.60264600
O	2.51186800	-0.63096700	-0.61263100
C	3.92799500	-0.91816200	-0.94881000
C	4.46022600	-1.51011300	0.35391100
C	4.65512200	0.38266400	-1.28446800
C	3.98395600	-1.94173800	-2.08053600
H	-4.01260400	3.13031600	0.61383900
H	-1.73032700	2.90892600	-0.32979300
H	-2.80248400	-0.89596300	-2.00589500

H	-5.06310500	-0.69126000	-1.07915600
H	-0.70447200	0.19841500	-2.67419200
H	-0.28103300	1.79386100	-2.06497600
H	1.14373600	0.49031300	1.19423300
H	-0.42912800	1.22333200	0.97342600
H	1.56967800	2.55216200	-0.83747500
H	1.17046200	3.15691800	2.16486000
H	-7.79225000	0.79655400	0.77843700
H	-7.15690900	0.28587900	-0.81177900
H	-6.58334000	-0.51651400	0.68566200
H	0.89955700	-3.30458700	1.77681600
H	-0.35454900	-4.55302100	1.91343100
H	0.15742600	-3.98760600	0.30984400
H	-2.22930000	-3.29449400	-0.33538800
H	-3.14654900	-2.21331400	0.74009500
H	-2.83354800	-3.89197400	1.22408700
H	-2.14456200	-1.41661200	2.91624600
H	-1.79854900	-3.08998300	3.40649400
H	-0.48806900	-1.88938000	3.36485000
H	3.90053800	-2.41096200	0.62963600
H	5.51492300	-1.77984200	0.23309900
H	4.37857900	-0.78228500	1.16897500
H	4.31355900	0.80431400	-2.23233400
H	5.72870700	0.17491500	-1.36539200
H	4.50941200	1.12120100	-0.48833100
H	5.02469700	-2.24955300	-2.23409800
H	3.39884000	-2.83207100	-1.82315000
H	3.60434600	-1.52711200	-3.01731700
H	2.04513600	4.19787300	0.91798900
C	3.62863000	2.51599400	2.16643300
O	3.41195500	1.89835700	3.21547700
O	4.61493900	2.78003600	1.47010400

ts2

C	2.06639900	-1.61885700	-1.64296800
C	1.54587800	-1.82311300	-0.36823300
C	2.42275900	-1.71268000	0.75269100
C	3.82400900	-1.55877300	0.54253200
C	4.30917300	-1.34845200	-0.74512400
C	0.07364200	-1.99473800	-0.11139900
C	-0.59790500	-0.71651900	0.48512200
C	0.18027900	-0.17610300	1.71394700
C	1.63222200	0.17642800	1.52197400
C	2.06768300	1.26535600	0.59678400
C	-2.00633900	-1.14060200	0.95444000
O	-2.30910200	-1.35528100	2.11411600
O	-2.81715700	-1.28239200	-0.09816500
C	-4.23920500	-1.68994600	0.02665100
C	-4.72044900	-1.65763100	-1.42165500
C	-4.99771900	-0.66827100	0.87207000
C	-4.31789200	-3.10468700	0.59648400
C	-0.81660200	0.34581200	-0.60693000
O	-0.58823200	0.18236200	-1.79112600
O	-1.34034500	1.44934700	-0.06184200
C	-1.80256000	2.60341900	-0.87221200
C	-2.24569600	3.59766500	0.19835700
C	-2.98532800	2.16245500	-1.73224700
C	-0.65365200	3.17944700	-1.69827600
C	1.92821800	2.72243900	1.15354900
O	2.12954700	3.63833100	0.30495700
O	1.66862200	2.86907000	2.37951500
O	5.62770200	-1.13310000	-1.04606100
C	6.55651900	-1.06606700	0.03554200
H	3.82997400	-1.22582400	-2.84775900
H	1.39632500	-1.63660900	-2.49785000

H	4.48561000	-1.56447300	1.40112900
H	-0.08073600	-2.80295300	0.61542900
H	-0.44974000	-2.26758600	-1.03028400
H	0.10376300	-0.94315400	2.49118700
H	-0.35685800	0.70309400	2.09609200
H	2.19344900	0.14665900	2.45501900
H	1.54955600	1.22477100	-0.36567800
H	3.13547800	1.13689500	0.36584500
H	-4.14278700	-2.35437000	-2.03921200
H	-5.77543700	-1.94850900	-1.46513000
H	-4.61928500	-0.65124300	-1.84164000
H	-4.68668200	-0.69573400	1.91846900
H	-6.06933800	-0.89263000	0.82225600
H	-4.84285500	0.34367000	0.48161700
H	-5.35704800	-3.45022300	0.55161400
H	-3.70442400	-3.79232100	0.00300100
H	-3.98569400	-3.14076600	1.63635100
H	-1.40091500	3.87934600	0.83653500
H	-2.63907500	4.50236600	-0.27768000
H	-3.03247600	3.16680700	0.82782100
H	-2.68476200	1.42176900	-2.47711400
H	-3.39132600	3.03528300	-2.25671800
H	-3.77921500	1.73748800	-1.10911000
H	-0.97949700	4.13543100	-2.12640300
H	0.22386300	3.36450600	-1.06871300
H	-0.37235000	2.51366400	-2.51688400
H	7.52840700	-0.86155700	-0.41822000
H	6.29932500	-0.25667800	0.73014300
H	6.60179500	-2.01702400	0.58091400
H	2.10303200	-2.13965500	1.70061800

ts3

C	5.50015000	0.92816200	1.13847700
C	4.88715100	-0.26783600	1.54435100
C	3.94403700	-0.88276600	0.72574000
C	3.57768800	-0.33064100	-0.51418900
C	4.21564100	0.85057600	-0.90931100
C	5.16714000	1.48529000	-0.10263100
C	2.52472700	-0.96894200	-1.39194600
C	1.06742900	-0.52969700	-1.06242700
C	0.54713800	-1.08227500	0.30612200
C	0.18492300	-2.54367300	0.30269200
C	1.18497400	-3.63357600	0.49105200
O	6.40563300	1.46645700	2.00733000
C	7.04784700	2.68682400	1.63294400
C	1.02260100	1.00810700	-1.12815400
O	1.16627600	1.60431600	-2.18383900
O	0.84995300	1.56704700	0.06496200
C	0.70557500	3.03502900	0.25254700
C	-0.52713900	3.51402700	-0.51101500
C	1.98644500	3.74939000	-0.17072800
C	0.49189600	3.15489300	1.75896700
C	0.11799900	-1.04289100	-2.15887100
O	0.41574300	-1.88334100	-2.98737900
O	-1.07784000	-0.46407200	-2.02293800
C	-2.23845200	-0.78779400	-2.89139400
C	-3.29173900	0.20766100	-2.41490900
C	-2.67703900	-2.22401300	-2.61508000
C	-1.89014200	-0.54392500	-4.35863300
H	5.16118700	-0.70446600	2.50095200
H	3.48832000	-1.81157500	1.05444300
H	3.96445300	1.29754600	-1.86739800
H	5.63069800	2.40154400	-0.44966800
H	2.71827500	-0.70883300	-2.43621400
H	2.57537500	-2.05685500	-1.31931400

H	-0.34230300	-0.50429500	0.56793200
H	1.30285200	-0.87687700	1.06861000
H	-0.69940500	-2.82197500	-0.27018800
H	1.97497200	-3.32318500	1.18114600
H	7.71221400	2.93883500	2.46160900
H	7.63765300	2.55973500	0.71686500
H	6.31660000	3.49211300	1.49201200
H	-1.39394300	2.89626400	-0.25705600
H	-0.74842200	4.54792200	-0.22269100
H	-0.37313600	3.48077900	-1.59185800
H	2.16300100	3.66292000	-1.24457900
H	2.85026900	3.34238400	0.36485600
H	1.89570200	4.81196100	0.08324400
H	1.34906500	2.74608300	2.30547900
H	0.37835300	4.20990400	2.03054000
H	-0.40965700	2.61700600	2.06789400
H	-2.96489900	1.23659400	-2.60005400
H	-4.22957300	0.03842200	-2.95461600
H	-3.47906000	0.08724800	-1.34495900
H	-1.91079200	-2.94364400	-2.91393000
H	-3.59008600	-2.43587800	-3.18332000
H	-2.89807000	-2.36019700	-1.55071900
H	-2.81069100	-0.59532000	-4.95140800
H	-1.45799300	0.45486100	-4.48867300
H	-1.18781900	-1.28605400	-4.74251200
H	1.66829300	-3.86105400	-0.47619100
C	0.54508000	-4.97584400	0.96544300
O	1.06637800	-5.51139800	1.98209800
O	-0.41852300	-5.40599800	0.27417100
C	-2.26120800	0.38514100	2.75853000
C	-3.12305100	1.35115400	2.24137200
C	-4.21731700	1.00644000	1.42719700
C	-4.40393300	-0.35626200	1.14804100

C	-3.55280900	-1.33417200	1.67015700
C	-2.47144700	-0.97743300	2.48778200
H	-1.41904600	0.68731400	3.37441600
H	-2.93176000	2.39361300	2.47844500
H	-5.22495300	-0.67815200	0.51742400
H	-3.73349400	-2.38078200	1.44129900
S	-1.40847500	-2.22049600	3.20358500
H	-0.60392800	-2.56571800	1.81090700
C	-5.15612800	2.10014800	0.90061000
C	-4.34860900	3.13644700	0.08955000
H	-5.01909100	3.91871000	-0.28846300
H	-3.58003600	3.62132900	0.70002300
H	-3.85563300	2.66640500	-0.76840100
C	-5.82379200	2.80815500	2.10028100
H	-6.50256100	3.59375900	1.74462500
H	-6.40765800	2.09774500	2.69863800
H	-5.08136400	3.27559300	2.75676200
C	-6.26219700	1.53515600	-0.00542800
H	-5.84903600	1.03410600	-0.88833100
H	-6.90248600	0.82168600	0.52682600
H	-6.89919600	2.35627300	-0.35473300
